# Chlamydomonas CHT7 is involved in repressing DNA replication and mitotic genes during synchronous growth

**DOI:** 10.1093/g3journal/jkac023

**Published:** 2022-02-07

**Authors:** Yang-Tsung Lin, Tomomi Takeuchi, Brian Youk, James Umen, Barbara B Sears, Christoph Benning

**Affiliations:** 1 Department of Biochemistry and Molecular Biology, Michigan State University, East Lansing, MI 48824, USA; 2 Department of Energy-Plant Research Laboratory, Michigan State University, East Lansing, MI 48824, USA; 3 Donald Danforth Plant Science Center, St. Louis, MO 63132, USA; 4 Department of Plant Biology, Michigan State University, East Lansing, MI 48824, USA

**Keywords:** cell cycle, cell division, quiescence, DNA replication, mitosis, algal cell wall, *Chlamydomonas reinhardtii*

## Abstract

In the green alga *Chlamydomonas reinhardtii*, regulation of the cell cycle in response to external cues is critical for survival in a changing environment. The loss of the nuclear COMPROMISED HYDROLYSIS OF TRIACYLGLYCEROLS 7 (CHT7) protein affects the expression of many genes especially in response to nitrogen availability. Cells lacking CHT7 exhibit abnormal cell morphology following nitrogen deprivation and fail to resume normal cell division after N resupply. To investigate the function of CHT7 in the regulation of cell cycle-related pathways, cells were synchronized, and RNA-seq analysis was performed during various stages of the cell cycle. In the *cht7* mutant following nitrogen deprivation, the cells were not dividing, but a subset of cell cycle genes involved in DNA replication and mitosis were found to be derepressed, suggesting that the CHT7 protein plays a role in cell cycle regulation that is opposite to that of the mitotic cyclin-dependent kinases. Furthermore, genes for cell wall synthesis and remodeling were found to be abnormally induced in nondividing *cht7* cells; this misregulation may deplete cellular resources and thus contribute to cell death following nitrogen deprivation. Lastly, 43 minimally characterized kinases were found to be highly misregulated in *cht7*. Further analysis suggested that some of these CHT7-regulated kinases may be related to the MAP3K and Aurora-like kinases, while others are unique. Together, these results suggest a role of CHT7 in transcriptional regulation of the cell cycle and reveal several pathways and genes whose expression appears to be subject to a CHT7-mediated regulatory network.

## Introduction

In the unicellular green alga Chlamydomonas (*Chlamydomonas reinhardtii*), orderly progression through the appropriate stages of the cell cycle is critical for survival in a changing environment. Different stages of the cell cycle exhibit specific metabolic activities and distinct cytological phases of cell division. Cellular metabolism and cell division are tightly coordinated to optimize the utilization of energy for cell growth and proliferation. The regulation of the cell cycle is modulated by the programming of gene expression. An RNA sequencing (RNA-seq) analysis by Zones *et al.* (2015) has documented the transcriptome dynamics throughout the life cycle of autotrophically growing wild-type (WT) Chlamydomonas cells under diel cycles. Briefly, genes of photosynthesis are highly expressed in the G1 stage during the daytime to ensure rapid cell growth driven by light energy. On the other hand, genes involved in cell division are not expressed until the end of the day, after the cell size-determined commitment point, when cells enter the S/M phase (S: DNA synthesis; M: mitosis). The peak expression of DNA replication, nuclear division, and chloroplast fission genes at this stage indicates rapid cycles of cell division. Cells that finish dividing then enter a short G0 period in the dark, with downregulation of cell division and cellular metabolism genes, while genes of flagellar assembly are actively expressed, after which the daughter cells will hatch out at dawn to begin a new life cycle (Zones *et al.* 2015). Together, the oscillating gene expression of cell division and metabolic pathways indicates the importance of transcriptional regulation during progression through the cell cycle.

In addition to the normal cell division cycle, transcriptional cell cycle control has also been observed during the transition into quiescence, a prolonged G0 phase, under nutrient-limited conditions (Gray *et al.* 2004). For example, Chlamydomonas following N deprivation has been reported to downregulate the transcription of genes encoding proteins that participate in photosynthesis, aerobic respiration, protein translation, and cell division. On the other hand, genes involved in triacylglycerol (TAG) and starch biosynthesis are upregulated in N-deprived cells, allowing the accumulation of carbon storage compounds (Miller *et al.* 2010; Schmollinger *et al.* 2014; Park *et al.* 2015). These transcriptional controls are required for the proper entry into quiescence and survival under stress.

The identification of COMPROMISED HYDROLYSIS OF TRIACYLGLYCEROLS 7 (CHT7, Cre11.g481800) has provided insight into the regulatory mechanism of quiescence transition in Chlamydomonas. The CHT7 loss-of-function mutant, *cht7*, was isolated during a screen for Chlamydomonas mutants that were slow or unable to degrade TAG when N is resupplied after N deprivation. Degradation of TAG allows the cell to reutilize the carbon and energy stored in that molecule. RNA-seq analysis of nonsynchronized, mixotrophic cells during the same study suggested that CHT7 is required for cells to transition between quiescence and the cell division cycle (Tsai *et al.* 2014). Subsequent work has shown that the expression of some cell cycle genes was strongly derepressed in *cht7* cells following N deprivation (Takeuchi *et al.* 2020b). A fraction of the N-deprived *cht7* cells were observed to have multiple nuclei and a swollen cell appearance, which was not seen in the *cht7* cells under N-replete (N+) conditions although the cell cycle genes were slightly misregulated (Takeuchi *et al.* 2020b). Most of the N-deprived *cht7* cells were unresponsive to N-refeeding, leading to the overall delayed TAG degradation mentioned above (Takeuchi *et al.* 2020b). Together, these results strongly suggested that CHT7 is required for viability during quiescence and for the proper transition from quiescence back into the normal cell division cycle.

The CHT7 protein has been localized to the nucleus and contains a CHC (CXC-Hinge-CXC) domain that is composed of 2 cysteine-rich CXC motifs (Tsai *et al.* 2014). Previous studies have shown that CHC proteins in plants and animals play an essential role in cell cycle regulation by forming complexes with various transcription factors and binding to DNA directly with their CHC domain (Beall *et al.* 2002; Thomas *et al.* 2003; Schmit *et al.* 2009; Sijacic *et al.* 2011; Matsuo *et al.* 2012; Sadasivam and DeCaprio 2013; Zheng *et al.* 2014; Marceau *et al.* 2016; Wang *et al.* 2018). However, it remains unclear whether CHT7 in Chlamydomonas functions through the same molecular mechanism. Our recent studies (Takeuchi *et al.* 2020b) suggested that the predicted DNA-binding domain of the CHT7 protein is not essential or is redundant as its deletion did not cause a detectable phenotype. In contrast, removal of the C-terminal disordered region resulted in a *cht7* null phenotype. This finding implicated a functional mechanism for CHT7 that may be distinct from other known CHC proteins and that does not require direct DNA binding by CHT7 through its CHC domain. Until now, most CHT7 studies have focused on the transition of quiescence following N deprivation and N resupply; the role of CHT7 during the normal cell division cycle has received less attention. One reason is that previous studies were done using asynchronous cell cultures (Tsai *et al.* 2014; Takeuchi *et al.* 2020b). To address this issue, we recently used cell cultures grown under conditions optimized for synchronization of cell division to investigate the impact of CHT7 on cell cycle progression, and we found that CHT7 proteins are more abundant during the S/M phase and may interact with the RB protein, suggesting that CHT7 might be a part of the cell cycle regulatory network (Takeuchi *et al.* 2020a). Here, to better understand the role of CHT7 in cell cycle regulation and to globally characterize the effect of CHT7 on gene expression under various conditions, we performed an RNA-seq of *cht7* and 2 control strains grown under light–dark conditions to achieve synchronized division. Our data show that genes involved in DNA replication, mitosis, and cell cycle regulation were inappropriately derepressed in *cht7* during quiescence and the G0 and G1 phases of the normal cell division cycle. We also found abnormally induced expression of genes encoding cell-wall components and potential regulators of cell wall remodeling in *cht7* following N deprivation. Lastly, we identified a previously uncharacterized group of protein kinases whose gene expression was subject to regulation by CHT7.

## Materials and methods

### Cell strains and growth conditions

Initial characterization of the 3 mating-type minus, cell-walled *C.reinhardtii* strains used in this study, 6145c (WT), *cht7*, and the *cht7* complementation line *CHT7-HA::cht7*, was reported previously (Takeuchi *et al.* 2020b). Synchronization of Chlamydomonas cells in HS medium with an environmental photobioreactor system (ePBR) using turbidostatic conditions (Lucker *et al.* 2014) has been described in detail by Takeuchi *et al.* (2020a). Three cell-walled, mating-type minus strains were used in this study: WT (6145c), the *cht7* mutant, and the *cht7* complemented line *CHT7-HA::cht7*. The complemented line was included here as an additional control to detect any possible effects caused by the deletion of genes other than CHT7 and other possible background mutations in the *cht7* mutant (Takeuchi *et al.* 2020b). For synchronization of Chlamydomonas cells in ePBRs (Lucker *et al.* 2014; Takeuchi *et al.* 2020a), cells were grown autotrophically in 330 ml of nitrogen containing high salt (HS+N) medium (Sueoka 1960) in ePBR under 12 h light:12 h dark cycles with a light intensity of 2,000 µmol m^−2^ s^−1^, and 5% CO_2_ was pumped into the cell culture regularly (30 s per 15 min). To maintain cell growth in mid-log phase, OD_940_ of the cell culture was monitored and diluted automatically by the ePBR using its turbidostat function. The target OD_940_ was determined according to the chlorophyll concentration (µg/ml) at ZT6, and all cultures were normalized to around 3 µg/ml of total chlorophyll. The cell cultures were grown under the condition above for 7–8 days to be synchronized prior to sample harvesting.

For N deprivation of the synchronized ePBR cell cultures, at ZT23 cells of each culture were pelleted at 3,000 g for 5 min and rinsed twice with 50 ml of N-free HS (HS−N) medium before being resuspended with 330 ml of HS−N medium. The N-deprived cells were then transferred to a clean ePBR vessel and grown under 2,000 µmol/m^2^ s of constant light with other growth conditions the same as the N+ cultures. For N-resupply of the nitrogen deprivation (ND) culture, aliquots of a sterile NH_4_Cl solution were added into N-deprived ePBR cultures at ND36 to bring the final concentration of NH_4_Cl to be the same as the HS+N medium.

### Flow cytometry analysis

Sample pretreatment for flow cytometry analysis was conducted according to a previous study (Fang *et al.* 2006). Five milliliters of cells harvested from ePBR culture were pelleted and resuspended in 5 ml of ethanol/acetic acid (3:1) solution for fixation for 2 h at room temperature. After fixation, cells were rinsed twice and resuspended with 1 ml of FACS buffer containing 0.2M Tris–HCl (pH 7.5) and 20 mM EDTA and stored at 4°C. Prior to flow cytometry analysis, the cell density of each sample was measured with a Z2 COULTER COUNTER Analyzer (Cat. 6605700, Beckman Coulter), and all samples were normalized to 1 × 10^6^ cells/ml in a total 900 µl of FACS buffer, mixed with 100 µl of FACS buffer containing 1 mg/ml of RNase A, and incubated at 37°C for 2 h. For DNA staining, the RNase A-treated samples were rinsed once and resuspended in 1 ml of PBS buffer. Four hundred and ninety-five microliters of sample were mixed with 5 µl of 100-fold diluted SYTOX Green (cat. S7020, Thermo Fisher Scientific) for 5 min. Flow cytometry was performed with the LSR II Flow Cytometer System (BD Biosciences) at the MSU Flow Cytometry Core (https://facs.iq.msu.edu/; accessed 2022 February 7), and data were analyzed with FACSDiva Software (BD Biosciences).

### RNA sequencing

Samples were harvested from ePBR cell cultures at various timepoints. Total RNA was extracted with Rneasy Plant Mini Kit (cat. 74903, Qiagen). The RNA concentration was measured with Qubit RNA BR Assay and Fluorometer (cat. Q10210 and cat. Q32866, Thermo Fisher Scientific). The quality of RNA was determined with Agilent 2100 Bioanalyzer and Agilent RNA 6000 Pico Assay (Cat. G2939BA and Cat. 5067-1514, Agilent Technologies) to ensure an RNA integrity number (RIN) value ≥8 before submitting an average of 4.36 µg of total RNA (220 ng/µl) per sample for RNA-seq. Lastly, RNA-seq was done in the Genomics Core at MSU RTSF (https://rtsf.natsci.msu.edu/genomics/). The cDNA libraries were prepared using TruSeq Stranded mRNA Library Preparation Kit (Illumina) and quantified using the Kapa Biosystems Illumina Library Quantification qPCR kit (Illumina). The pools of ZT sample and ND/NR sample libraries were loaded onto a total of 4 lanes of Illumina HiSeq 4000 flow cells and sequenced in a 1× 50 bp single-end mode using HiSeq 4000 SBS reagents. For each sample, around 25 million reads were generated, processed, and then mapped to Creinhardtii_281_v5.6.transcript downloaded from Phytozome v12.1 (https://phytozome-next.jgi.doe.gov/) using Salmon 0.11.3 (Patro *et al.* 2017) and R packages tximport 1.16.1 (Soneson *et al.* 2015) and DESeq2 1.28.1 (Love *et al.* 2014) to obtain the normalized read count of individual transcripts.

### Data processing and differential expression analysis

The raw sequence reads were mapped to Creinhardtii_281_v5.6 and calculated for transcript abundance with Salmon 0.11.3 (Patro *et al.* 2017). The quantified read counts were normalized through division by the size factor of each sample with the DESeq2 1.28.1 package in R v4.0.0 (Love *et al.* 2014). Principal component analysis (PCA) of all 81 samples was done using the same package with the variance stabilizing transformation function. The average read count of individual transcripts was calculated from 3 biological replicates of each group (e.g. WT_ZT6). Differential expression (DE) analysis was performed to obtain the log_2_-fold change of replicative average read counts of a transcript of any 2 strains at the same timepoint (e.g. Cre08.g372550.t1.1, *cht7* vs wild type at ND6). To estimate the statistical significance of the fold change, adjusted *P*-value (*P-*adj) was calculated from the original *P*-value using the Benjamini–Hochberg rule to correct for the false discovery rate (false positive). Differentially expressed genes (DEGs) were identified using *P-*adj < 0.05 and abs (log_2_-fold change) >1. A *cht7*-specific DEG is defined as a gene being differentially expressed in *cht7* compared with both WT and *CHT7-HA::cht7*, but not being differentially expressed between WT and *CHT7-HA::cht7*.

### Hierarchical clustering

Among the total 19,526 transcripts, we selected 14,456 transcripts with an average read count >20 that were differentially expressed [abs (log_2_-fold change) >1, *P-*adj < 0.05) between any timepoint and the mean of all timepoints. Read counts of these DEGs were then scaled to *Z*-score by the equation *x-x̄/σ*, where *x* is the read count of a timepoint and *x̄* is the mean of all timepoints, σ is the SD of the mean. Hierarchical clustering of the scaled data matrix was then performed in R using the Pearson correlation option to measure the similarity in *Z*-score across all timepoints between genes, and 14 gene clusters were generated by cutting the resulting hierarchical tree at height = 1.7. As a result, genes of each cluster have a unique expression profile in response to various genotypes and conditions.

### Gene ontology enrichment analysis

Two references of gene ontology (GO) annotation downloaded from EnsemblPlants (dataset: creinhardtii_eg_gene) and Phytozome v12.1 (dataset: Creinhardtii_281_v5.6) were combined into 1 GO annotation table and further analyzed in R. Among the total of 17,743 genes in the Chlamydomonas genome v5.6, 11,159 genes were annotated with at least 1 GO term. The input gene set for GO enrichment analysis was composed of the 14 gene clusters (Fig. 2c) or the *cht7*-specific upregulated DEGs (Fig. 3c). The analysis was performed using the R package topGO 2.40.0 (Alexa *et al.* 2006; Alexa and Rahnenfuhrer 2020) with the *nodeSize = 5* option to construct the GO tree prior to analysis. The significance of enriched GO terms was determined by Fisher’s exact test combining with the *weight01* algorithm to determine the most representative GO term by comparing the enrichment score of a GO term with its subsequent GO term. The threshold for a significantly enriched GO term is *P*-value <0.05.

### Phylogenetic analysis

Peptide sequences of the Chlamydomonas kinases including both the 46 *cht7*-induced kinases and reference kinases were obtained from Phytozome v12.1 (dataset: Creinhardtii_281) using R package biomaRt 2.44.0 (Durinck *et al.* 2009), and sequences of Arabidopsis kinases were downloaded from Phytozome v12.1 using PhytoMine web tools. Sequences of CcaMKs were obtained directly from Tirichine *et al.* (2006). Three *cht7*-induced kinases encoded by Cre01.g055457.t2.1, Cre12.g526150.t1.2, and Cre12.g526450.t1.2 were removed from the analysis to avoid duplicated sequences. Sequences of kinase domain were retrieved using an R script searching for IPR000719 Protein kinase domain defined by InterPro (https://www.ebi.ac.uk/interpro/). For the kinase encoded by Cre09.g404550.t1.2, only the IPR004166 MHCK/EF2 kinase domain was found and was used in this study. For the kinase encoded by Cre02.g108750.t1.2, 2 kinase domains were found, and they were both included in the analysis (labeled as Cre02.g108750a and Cre02.g108750b). Multiple sequence alignment of the total 166 kinase domains (122 reference sequences and 44 sequences of interest) above was performed using ClustalOmega in R package msa 1.19.0 (Bodenhofer *et al.* 2015). Maximum likelihood (ML) phylogeny was estimated using the Whelan and Goldman substitution model implemented in PhyML 3.3 software (Whelan and Goldman 2001; Guindon *et al.* 2010), and branch support was measured by a parametric, chi-square-based approximate likelihood-ratio (aLRT) test (Anisimova and Gascuel 2006). The resulting unrooted ML tree was furthered analyzed and rooted with FigTree v1.4.4 (http://tree.bio.ed.ac.uk/software/figtree/).

## Results

### Cell cycle analysis under synchronizing conditions

**Fig. 1.**
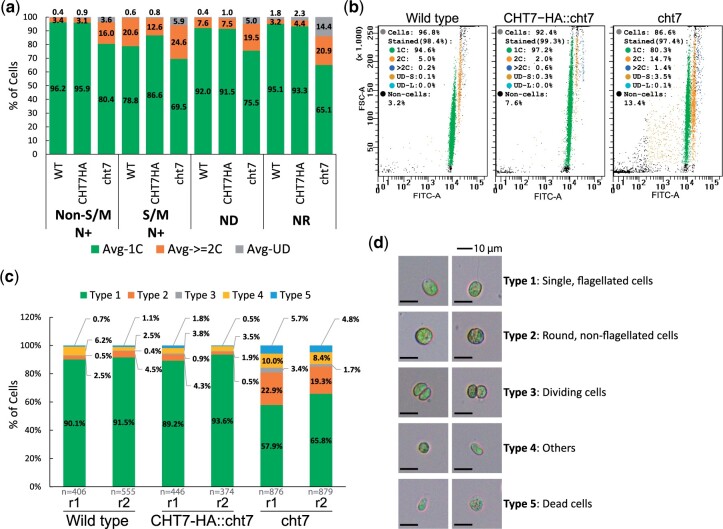
Assessment of cell cycle progression under synchronizing conditions. a, b) Flow cytometry analysis of DNA-stained cell cultures grown under N+, ND, and NR conditions. Samples of cells grown under synchronizing conditions were fixed and stained with SYTOX Green prior to flow cytometry analysis. Summary of DNA content (a) of cells at non-S/M (ZT0-10, ZT24) and S/M phases (ZT12-16) of N+ condition as well as cells under ND (ND0, 6, 12, and 36) and NR (NR6 and 12) conditions. *Y*-axis: average population (%) of cells carrying single (green), ≥2 (orange), or undetermined (gray) copies of chromosomes. *n* = 2 for each sample. Example plot (b) showing particle size (FSC-A; Forward scatter) vs DNA content (FITC-A) for samples at ZT6. Each dot represents noncell particle (black) or a cell carrying a single (green), 2 (orange), >2 (blue), or undetermined (brown and cyan) copies of the genome. One of the 2 replicates (*n* = 2) for each sample is presented. See also Supplementary Fig. 1. c, d) Cell morphology of wild type, CHT7-HA::cht7, and cht7 cells at ZT6. Two biological replicates (r1 and r2) for each strain were observed under the microscope and summarized in (c). *n*: numbers of cells observed. *Y*-axis: population (%) of cells of type 1 (green), 2 (orange), 3 (gray), 4 (yellow), and 5 (blue). The representative graphs and definition of each cell type are shown in (d).

Our previous observations showed largely synchronized growth of the *cht7* mutant and WT cells in photobioreactors in the presence of N while the cultures were bubbled with CO_2_ (Takeuchi *et al.* 2020a). To investigate the role of CHT7 in cell cycle regulation, 3 strains, wild type (WT), the *cht7* mutant, and the *cht7* complemented line *CHT7-HA::cht7*, were cultured under 3 sequential growth conditions determined by the availability of N: N-replete (N+), N-deprived (ND), and N resupplied (NR). N+ was defined as the standard growth condition, and samples harvested from cultures grown under this condition were collected at different time points ZT*n*, where *n* represents the time after the onset of light (ZT stands for Zeitgeber time, e.g. ZT6 was taken 6 h after illumination). Similarly, the ND*n* samples were taken *n* hours after N deprivation; these should represent cells in the quiescent state. The NR*n* samples were taken *n* hours following N resupply to investigate the transition from quiescence to the normal cell division cycle. We observed the typical progression of the cell cycle and the cellular response to N+, ND, and NR with cell size distribution analysis (Supplementary Fig. 1, left column). In all instances, the cells gained in size from ZT0 to ZT10 and underwent cell division between ZT12 and ZT16. Cells finished dividing and gave rise to single, small cells at ZT24 (ZT0) entering the next G1 phase. To obtain an independent quantitative measure for cell cycle progression and synchronization, we determined the DNA content of individual cells (Supplementary Fig. 1, right column). Our previous microscopy studies of DAPI-stained cells indicated that some N-deprived *cht7* cells are multinucleated (Tsai *et al.* 2014; Takeuchi *et al.* 2020b); this phenotype was not observed in *cht7* cells grown under standard N+ conditions. Here, we applied a more quantitative method using flow cytometry of cells stained with the DNA dye SYTOX Green (Supplementary Fig. 1, right column; Fig. 1, a and b) following established protocols (Fang *et al.* 2006; Tulin and Cross 2014). As shown in Fig. 1a, an average of ∼96% of WT and *CHT7-HA::cht7* cells stained with SYTOX Green and N+ grown were found to contain a haploid DNA content (1C) at ZT0-10, prior to DNA replication. In contrast only 80% of the stained *cht7* cells had a 1C DNA content with almost 20% carrying 2 or more genomes (≥2C) at the same timepoints (Fig. 1a; also see Fig. 1b for an example at ZT6). In addition, there appeared to be more cell debris (Fig. 1b, noncells) and cells with irregular DNA contents (Fig. 1b, UD-S and UD-L) across all N+, ND, and NR conditions for the *cht7* cultures. While the loss of viability of *cht7* cells under ND and NR has been previously reported using different methods (Takeuchi *et al.* 2020b), these more sensitive flow cytometry results point to some loss of viability under regular N+ growth conditions. Finally, at timepoints of cell division under N+ growth (S/M, Fig. 1a), the population of cells containing ≥2C DNA increased far more in *cht7* (30.5%) than in the WT and *CHT7-HA::cht7* line (21.2% and 13.4% respectively). Furthermore, under ND and NR culture conditions, *cht7* cells contained a ≥2C DNA content in a larger fraction of cells than did WT and *CHT7-HA::cht7* cultures (Fig. 1a; Supplementary Fig. 1). These findings were consistent with our previous observations of N-deprived *cht7* cells (Takeuchi *et al.* 2020b). The persistent presence of a significant fraction of *cht7* cells with >2C chromatin content throughout the sampling regime suggests that in the WT, the CHT7 protein may suppress the initiation of DNA replication. Impairment of this process in the *cht7* mutant could impede complete synchronization of *cht7* cultures, as discussed further below.

To observe the impact of the loss of CHT7 on the G1 phase under N+ conditions, we examined *cht7* cells collected at ZT6 by microscopy. As shown in Fig. 1, c and d, cells were classified based on their morphology and viability. The majority of WT and *CHT7-HA::cht7* cells were single and flagellated (type 1, 89.2–91.5%). In the *cht7* mutant, there were more cells with diverse morphological phenotypes such as round and nonflagellated cells (type 2, 19.3–22.9%), dividing cells (type 3, 1.7–3.4%), others (type 4, 8.4–10.0%), and dead cells (type 5, 4.8–5.7%). Together, these results indicated that *cht7* cells have a broader range of nuclear contents and cell types than do the 2 WT controls under conditions that synchronize the cell cultures, possibly due to an altered regulation of the cell cycle in the mutant.

### Transcriptome dynamics of cell cultures under various conditions

**Fig. 2.**
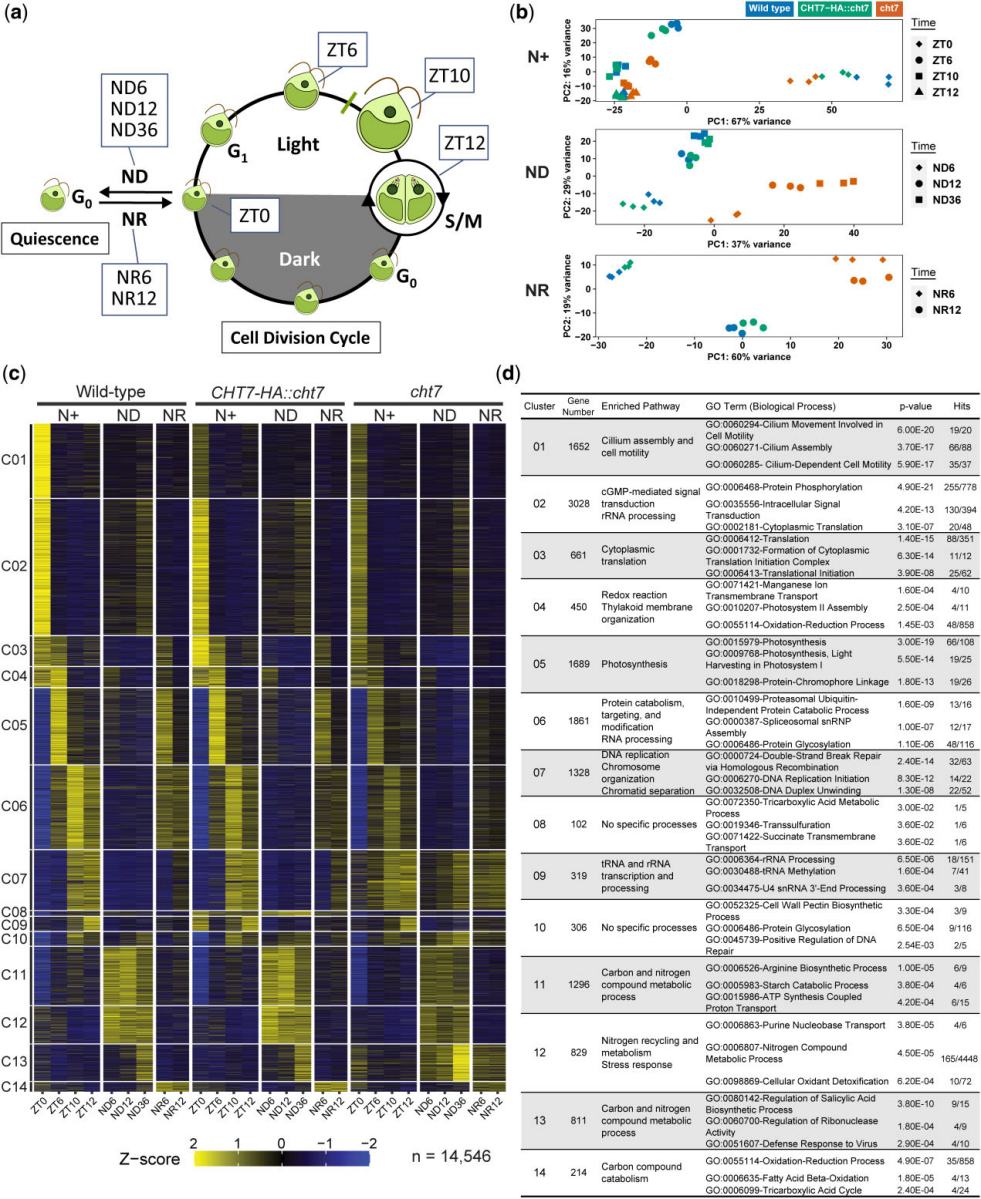
Transcriptome analysis of Chlamydomonas cell cultures under synchronizing conditions. a) Diagram of Chlamydomonas cell cycle and experimental design for RNA-sequencing analysis. Wild type, *CHT7-HA::cht7*, and *cht7* cells were grown photoautotrophically in an environmental photobioreactor (ePBR) under turbidostatic control. ZT(*n*) samples represent cells at different stages of the cell division cycle under normal N+ conditions, where *n* = hours after the onset of light. Quiescence was triggered by nitrogen deprivation (ND) in continuous light. ND(*n*) samples were harvested, where *n* = hours after N deprivation. The exit from quiescence was induced by N resupply (NR). NR(*n*) samples were harvested after *n* hours of N refeeding. Three independent biological samples (*n* = 3) were analyzed. b) PCA of transcriptomes of all 81 samples. Samples are labeled with different colors and shapes as shown on the top and right of the graph to indicate combinations of different genotypes and timepoints, respectively. c) *Z*-score heatmap and d) GO enrichment analysis of 14 gene clusters across all conditions. In total, 14,456 transcripts with an average normalized read count >20 (*n* = 3) differentially expressed in any sample and timepoint were grouped into 14 gene clusters by hierarchical clustering based on their *Z*-scores. Each cluster represents a unique expression pattern across all conditions. Hits (x/y): number of the input genes (x) over total genes (y) within a GO term. See also Supplementary Fig. 1 and Table 1.

Because the decreased synchrony and increased DNA content of *cht7* cultures point toward a possible role of CHT7 in cell cycle regulation of Chlamydomonas, we set out to better understand the scope of the transcriptional networks that could be affected by CHT7, and we performed RNA-seq analysis of WT and *CHT7-HA::cht7* and *cht7* cell cultures. Samples were collected in biologically independent triplicates at 9 representative timepoints under the N+, ND, and NR conditions corresponding to key points of the cell cycle and transitions in and out of quiescence (Fig. 2a).

After processing and mapping raw reads to the Chlamydomonas predicted transcriptome v5.6 (Creinhardtii_281_v5.6, Phytozome), we performed PCA to compare the transcriptomes of samples in the N+, ND, and NR conditions. As shown in Fig. 2b, samples with different transcriptomes form distinct groups across the dimensions of PC1 and PC2, the 2 principal components contributing most to the total variance between the samples in each condition. Samples with similar transcriptome compositions, on the other hand, were close to each other (e.g. wild type and *CHT7-HA::cht7*). The transcriptomes of *cht7* from the ND (middle plot; Fig. 2b), NR (lower plot; Fig. 2b), and N+ (ZT6) (circle dots; upper plot; Fig. 2b) samplings were most different from the respective 2 control samples, suggesting specific functions of CHT7 at these timepoints. Notably, under N+ conditions, the ZT0 samples also compose a unique group on the plot, because they represent the only time points at which cells were harvested in the dark (diamond dots; upper plot; Fig. 2b). As expected, light has a strong impact on the transcriptome of Chlamydomonas. It should be noted that *cht7* samples for ZT0 fell into the same group as WT and *CHT7-HA::cht7* suggesting that light responsive signaling is functional in the mutant, which is a prerequisite for synchronizing cell division.

Next, we performed hierarchical gene clustering to group the transcription profiles of 14,946 transcripts across all samples with an average read count >20 (*n* = 3), settling on 14 gene clusters with unique expression profiles (C01–14; Fig. 2c). GO enrichment analysis showed that each gene cluster was annotated with specific functions correlated to their expression pattern (Fig. 2d; Supplementary Table 1). For example, genes for cilium assembly and cytoplasmic translation were found in the C01 and C02 clusters, respectively, which were mainly transcribed in the dark in all strains, as reported previously (Zones *et al.* 2015). On the other hand, GO terms related to photosynthetic processes were highly enriched in group C05 genes, which were mainly transcribed at ZT6 in all strains, indicating that cells at this timepoint were highly active photosynthetically. Meanwhile, genes falling into the C11 and C12 clusters were highly expressed in ND samples of all strains, and GO terms enriched in these 2 clusters were mostly related to the metabolism of N-containing and other carbon compounds as well as stress responses, suggesting an adjustment of metabolic pathways in response to N deprivation (Fig. 2c and d). In summary, the transcriptional profiles of metabolic and cell cycle genes of the 2 control Chlamydomonas strains were generally consistent with previous transcriptomic studies of synchronized N+ cells (Zones *et al.* 2015) and ND cells (Miller *et al.* 2010; Schmollinger *et al.* 2014), allowing us to focus on the DEGs in the *cht7* mutant.

### A global analysis of upregulated genes in the cht7 mutant

**Fig. 3.**
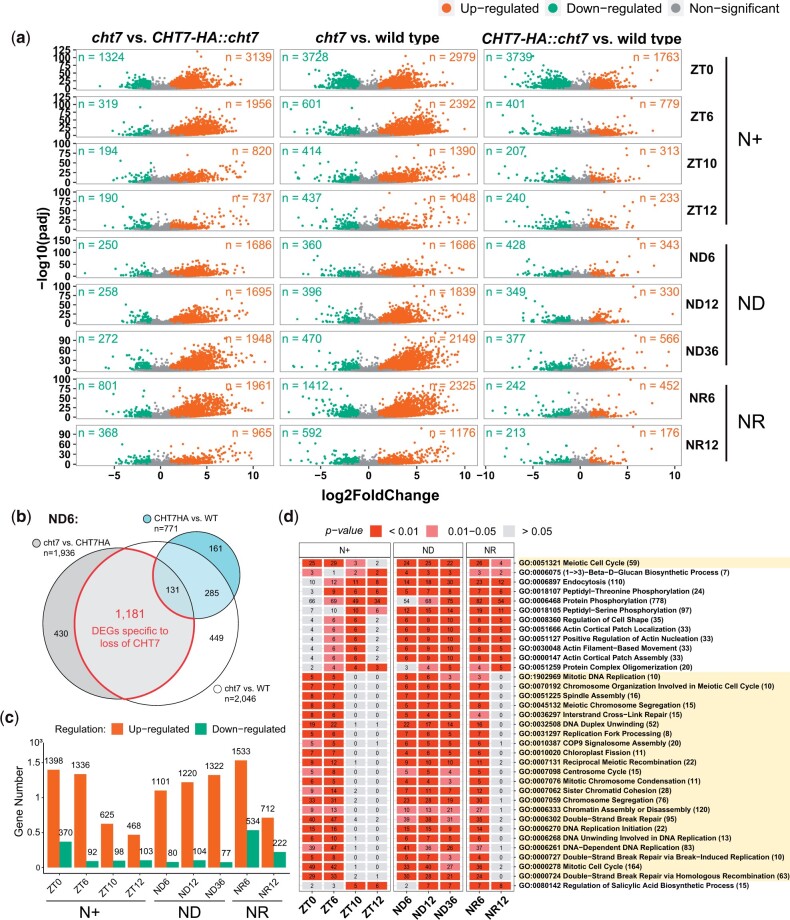
DE analysis of all 3 strains. a) Volcano plots showing the upregulated (orange; log_2_-fold change > 1, *P-*adj < 0.05), downregulated (green; log_2_-fold change < 1, *P-*adj < 0.05), and non (gray) DEGs in 3 groups of any 2 genotypes (e.g. *cht7* vs wild type) across all timepoints. *X*-axis: log_2_-fold change. *Y*-axis: -log_10_ adjusted *P*-value (*P-*adj). *n* = number of DEGs. b) Venn diagram example showing the selection of *cht7*-specific DEGs from the ND6 samples. DEGs between the WT and *CHT7HA::cht7* were excluded from DEGs between *cht7* and WT and *CHT7HA::cht7*, respectively. c) Numbers of up- (orange) and down- (green) regulated *cht7-*DEGs across all timepoints. d) GO enrichment analysis of upregulated *cht7*-DEGs across all timepoints. Twenty-three GO terms of biological processes related to the S/M phase are highlighted in light yellow. Number of upregulated DEGs are labeled in the boxes. Number of total annotated genes are indicated after GO terms. See also Supplementary Fig. 2.

We performed DE analysis to compare the read count of all individual transcripts (*n* = 19,923) of any 2 strains at the same timepoints (e.g. *cht7* vs WT at ZT6). The resulting DEGs (log_2_-fold change >1 [up] or <1 [down] and *P-*adj < 0.05) are displayed in Fig. 3a. Notably, when comparing *cht7* to the WT or the complemented line, the upregulated DEGs clearly outnumber the downregulated DEGs. This pattern was found at all time points except ZT0 (Fig. 3a), at which ∼3,700 genes were down-regulated in both *cht7* and the complemented line, *CHT7HA::cht7*, compared with WT. This difference might be due to the deletion of several other genes in the background of the *cht7* mutant (Tsai *et al.* 2014). GO enrichment analysis showed that many of the specifically downregulated genes in *cht7* and *CHT7HA::cht7* vs the WT participate in processes such as cilium assembly and cell motility (Supplementary Table 2). Because these downregulated DEGs at ZT0 are likely attributable to the original *cht7* mutant background, we will not discuss them further in this study, but focus on DEGs directly related to the loss of CHT7.

To identify DE that is specifically caused by the loss of CHT7 but not the other gene deletions in the *cht7* background, we compared the 3 groups at each timepoint (Fig. 3b). To be designated as a *cht7*-specific DEG, the gene must be differentially expressed in *cht7* compared with the controls and not be differentially expressed in *CHT7-HA::cht7* in comparison to WT. With this approach, we identified *cht7*-specific DEGs that were either up- or downregulated in the absence of CHT7 (Fig. 3c). It was quite striking that numbers of upregulated DEGs were 2.9- to 14.5-fold higher than downregulated DEGs across all timepoints, indicating that CHT7 might primarily be involved in transcriptional repression (Fig. 3c).

To gain insights into the function of the DEGs due to the loss of CHT7, we performed GO enrichment analysis of the total 3,551 DEGs that were specifically upregulated in *cht7* at more than 1 timepoint; 184 GO terms of biological processes were found to be highly enriched (*P <* 0.05) in these genes (Supplementary Fig. 2). Among these GO terms, 35 were enriched in at least 6 timepoints and were, therefore, chosen for further study (Fig. 3d).

### CHT7 suppresses transcription of genes involved in DNA replication and mitosis

A group of 23 GO terms (Fig. 3d; yellow) first caught our attention because they were all related to processes during the S/M phase of the cell cycle (e.g. DNA replication, nuclear division, chloroplast fission) and were highly enriched in *cht7*-specific upregulated DEGs (*P <* 0.01), suggesting that they are repressed in the presence of CHT7. Furthermore, this specific enrichment pattern supported the finding that genes corresponding to those cell cycle processes in the GO annotations were programmed to be transcriptionally activated during cell division ZT10, ZT12, and NR12 (Fig. 2c; C07).

Derepressed cell cycle genes in *cht7* under both N+ and ND conditions have also been observed in previous studies using nonsynchronized cell cultures (Tsai *et al.* 2014; Takeuchi *et al.* 2020b). To further specify the S/M processes potentially regulated by CHT7, we carefully investigated the annotated functions of the 512 genes under 23 GO terms mentioned above, among which 236 (46%) were upregulated in *cht7* at more than 1 timepoint. Information from multiple functional prediction databases and a previous study of cell cycle genes of Chlamydomonas (Zones *et al.* 2015) were included for this purpose (Supplementary Table 3).

**Fig. 4.**
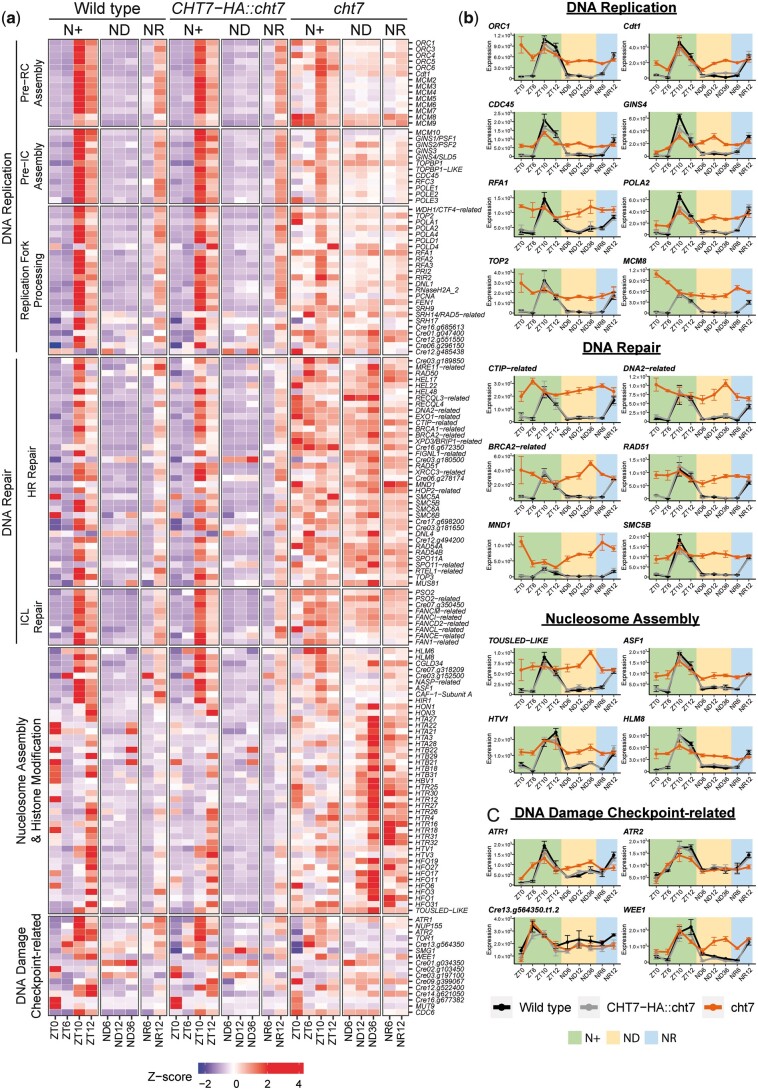
Derepressed DNA replication in the *cht7* mutant. a) *Z*-score heatmap of S-phase genes across all conditions. Genes that were differentially expressed (log_2_-fold change > 1, *P-*adj < 0.05) in *cht7* at ≥1 timepoints are presented. pre-RC, prereplicative complex; pre-IC, preinitiation complex; HR repair, homologous recombination repair; ICL, interstrand crosslink. Expression of representative S-phase genes (b) and DNA damage checkpoint-related genes (c) in *cht7* (orange), WT (black), and *CHT7-HA::cht7* (gray) across all timepoints. *Y*-axis: normalized read count. ORC1, origin recognition complex 1; Cdt1, Cdc10-dependent transcript 1; CDC45, cell division cycle 45; RFA1, replication factor A1; POLA2, DNA polymerase α2; TOP2, topoisomerase II; MCM8, minichromosome maintenance 8; CTIP, CtBP (C-terminal binding protein) interacting protein; BRCA2, breast cancer gene 2. MND1, meiotic nuclear divisions 1; SMC5B, structural maintenance of chromosomes 5; ASF1, anti-silencing factor 1; HTV1, histone H3 variant 1; HLM8, histone-lysine N-methyltransferase 8; ATR, Ataxia Telangiectasia mutated (ATM) and Rad3-related.

The results showed that 116 of the 236 DEGs were associated with DNA replication and DNA-replication-coupled homologous recombination (HR) repair, interstrand crosslink (ICL) repair, and chromatin assembly (Fig. 4a; Supplementary Table 3). These processes are referred to as S-phase events. During the normal cell division cycle, most genes that participate in S-phase events are transcribed at ZT10 and ZT12 (S/M phase) and turned off at ZT0 (G0 phase) and ZT6 (G1 phase). During ND-induced quiescence, these genes are repressed and not expressed until 12 h after N-resupply (Fig. 4a; see columns of WT and *CHT7-HA::cht7*). However, in the *cht7* mutant, many of these S-phase genes are not repressed, although the expression of these genes is not further increased at ZT10, 12, and NR12 either (Fig. 4, a and b). Figure 4b shows the fluctuation of transcript levels of genes known to be involved in specific events of the S-phase such as pre-replicative complex assembly (ORC1, Cdt1, and MCM8), preinitiation complex assembly (Cdc45 and GINS4), DNA synthesis (POLA2, RFA1, and TOP2), DNA repair (CTIP-related, DNA2-related, BRCA2-related, and RAD51), and nucleosome assembly (TOUSLED-LIKE, ASF1, HTV1, and HLM8) (Fabry *et al.* 1995; Vignard *et al.* 2007; MacNeill 2010; Nimonkar *et al.* 2011; De Benedetti 2012; Makharashvili and Paull 2015; Zones *et al.* 2015; Godin *et al.* 2016; Riera *et al.* 2017; Horard *et al.* 2018) in the WT lines, in contrast to the high background levels of expression in *cht7*. The constitutive expression of genes in *cht7* that are normally activated specifically in S-phase may provide an explanation for the presence of ≥2C DNA cells in *cht7* cultures (Fig. 1). To test the possibility that replicative stress in the *cht7* mutant caused the observed phenotypes, we examined the expression of homologs of genes that encode proteins known to be involved in sensing or repairing DNA damage in plants such as ATR kinases ATR1, ATR2, ATR-like Cre13.g564350, and the CDK inhibitory kinase WEE1 (Fig. 4c) (Bisova *et al.* 2005; Nisa *et al.* 2019). There was no significant difference in the expression of these genes between *cht7* and the control strains, except for the gene encoding WEE1 which was abnormally expressed in a pattern similar to other derepressed S-phase genes in *cht7*.

**Fig. 5.**
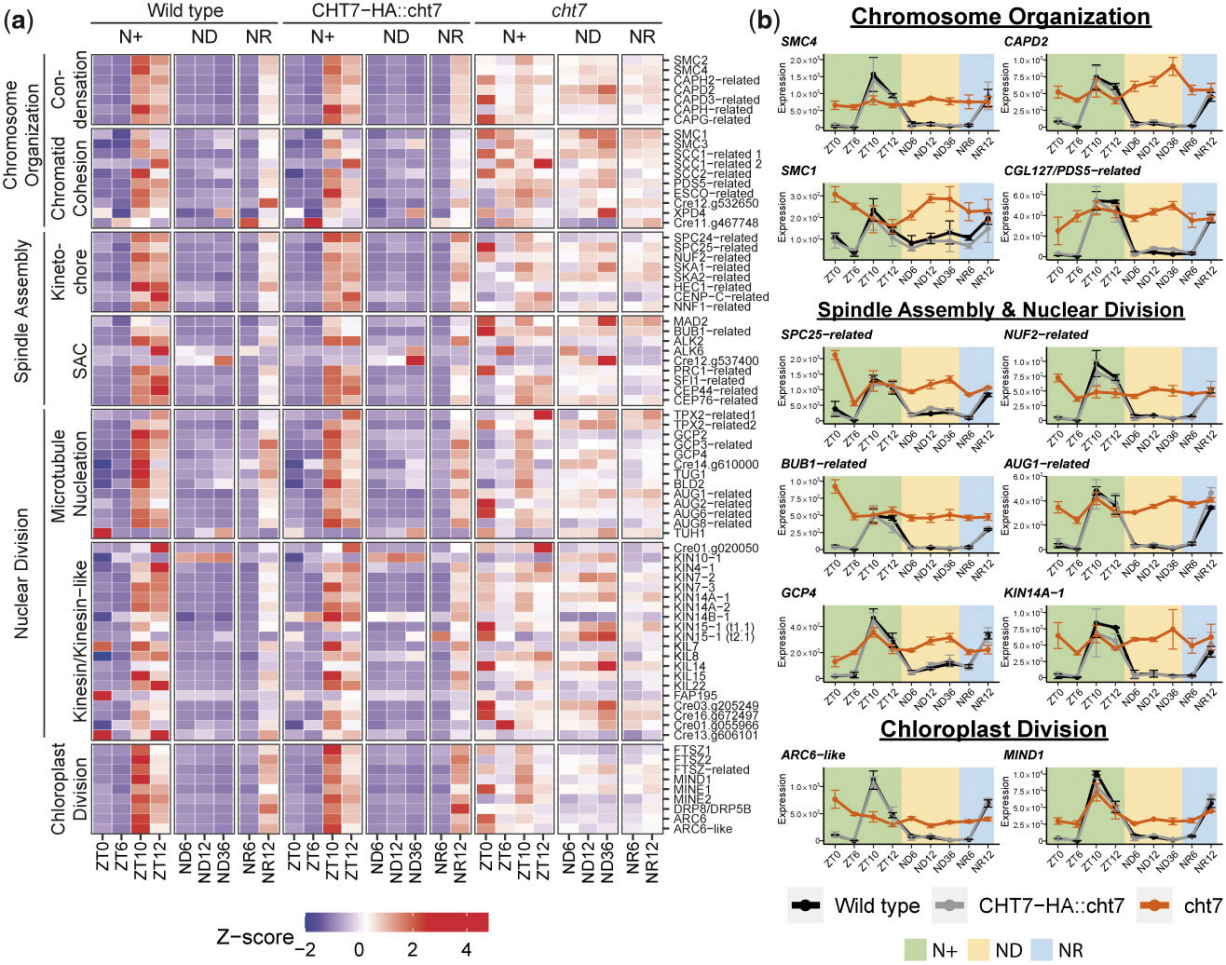
Derepressed mitotic genes in the *cht7* mutant. a) *Z*-score heatmap of mitotic genes. Genes that were differentially expressed (log_2_-fold change > 1, *P-*adj < 0.05) in *cht7* at ≥1 timepoints are presented. (SAC) Spindle assembly checkpoint (b) Expression of representative mitotic genes in *cht7* (orange), WT (black), and *CHT7-HA::cht7* (gray) across all timepoints. *Y*-axis: normalized read count. SMC4, structural maintenance of chromosomes 4; CAPD2, chromosome-associated protein D2; SPC25, spindle pole component 25; NUF2, nuclear filament-containing protein 2; AUG1, AUGMIN subunit 1; BUB1, budding uninhibited by benzimidazole 1; GCP4, γ-tubulin complex protein 4; KIN14A-1, kinesin-like protein 14A-1; ARC6-like, accumulation and replication of chloroplasts 6-like; MIND1, minicell mutant D1. See also Supplementary Table 3.

Lastly, we found that 68 of the 104 genes participating in mitotic processes (chromosome condensation, sister chromatid cohesion, spindle assembly, nuclear division) and chloroplast division seemed to be constitutively expressed in *cht7* (Fig. 5a; Supplementary Table 3). Some of these genes are known to play a crucial role in the mitotic events following DNA replication in vertebrates and yeast (Musacchio and Salmon 2007; Peters *et al.* 2008; Sundin *et al.* 2011; Hirano 2012; Chan *et al.* 2013; Cheeseman 2014; Tian and Kong 2019) and chloroplast fission in plants (Zones *et al.* 2015; Chen *et al.* 2018), and their transcript levels are shown in detail in Fig. 5b. However, it remains unclear whether mitosis of *cht7* cells was directly affected given that previous studies did not show obvious abnormal cell division phenotypes in the *cht7* mutant under N+ conditions (Takeuchi *et al.* 2020a,b). Together, these findings suggest that CHT7 is required for downregulating the transcription of genes involved in S/M phase-related processes during both the regular cell division cycle and quiescence, and that in *cht7* DNA replication seems to be activated across all conditions.

### How does CHT7 fit into the regulatory network of the cell cycle in Chlamydomonas?

**Fig. 6.**
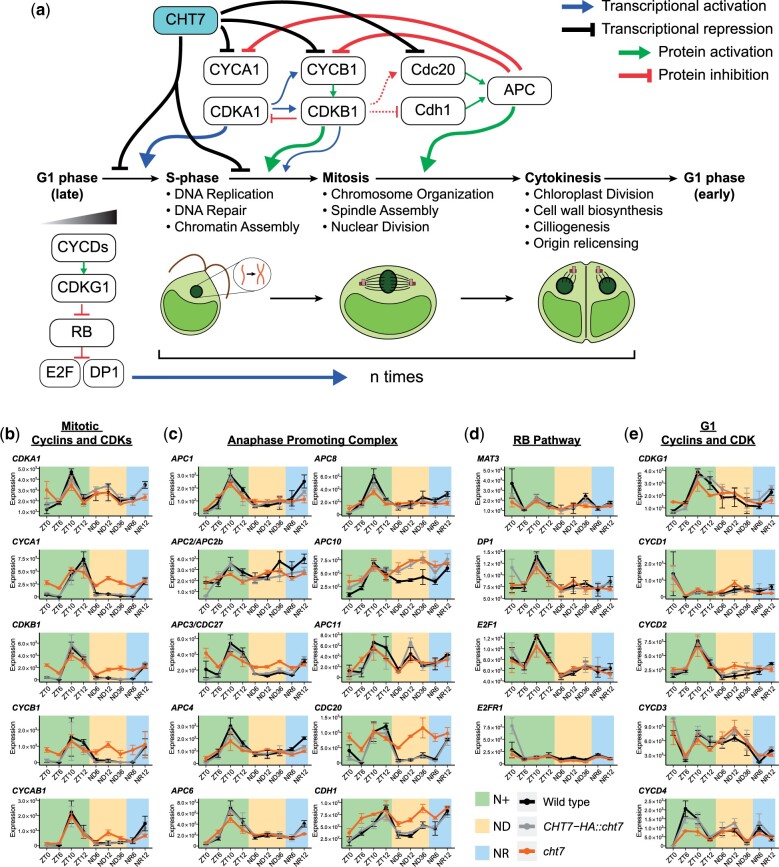
Impact of CHT7 on cell cycle regulation of Chlamydomonas. a) Regulatory network of the cell cycle of Chlamydomonas and proposed model of CHT7-mediated cell cycle regulation. Refer to text for details. b–e) Expression of the genes encoding cell cycle regulators in *cht7* (orange), WT (black), and *CHT7-HA::cht7* (gray) across all timepoints. *Y*-axis: normalized read count. RB, retinoblastoma protein; E2F, E2 factor; DP1, dimerization partner 1; CYC, cyclin; CDK, cyclin-dependent kinase; APC, anaphase-promoting complex; CDC20, cell division cycle protein 20; CDH1, Cdc20 homolog-1. See also Supplementary Table 3.

The finding of derepressed S/M phase genes in *cht7* during the normal cell division cycle in N+-grown cells suggested that CHT7 might have a function in the regulatory network of the cell cycle in Chlamydomonas. It also raised the possibility that CHT7 contributes to modulating the expression of cell cycle regulators, which in turn affect the transcription of downstream targets. To test this hypothesis, we examined the expression of genes encoding known cell cycle regulators of Chlamydomonas (Fig. 6; Supplementary Table 3). Figure 6a describes the current understanding of cell cycle regulation of Chlamydomonas. The timing of entry into the S/M phase is mainly controlled by a cyclin-dependent kinase, CDKA1, in a cell-size-dependent manner (Cross 2020). In the late-G1 phase, cells reaching the cell size threshold for cell division activate CDKA1, which induces the transcription of *CYCB1*, *CDKB1*, and other essential genes for DNA replication and mitosis (Tulin and Cross 2015; Atkins and Cross 2018; Cross 2020). The initiation of the S/M program by CDKA1 triggers the activation of CDKB1 by CYCB1, which then inactivates CDKA1 (negative feedback) and promotes the entry into mitosis (e.g. spindle formation), inducing the reinitiation of DNA replication after the first round of nuclear division (Tulin and Cross 2015; Atkins and Cross 2018). Another important regulator for the progression of the cell cycle is the anaphase-promoting complex (APC), a universally conserved complex required for chromosome segregation (Cross and Umen 2015). In Chlamydomonas, APC is required for both mitotic exit and the inactivation of CDKA1 and CYCB1–CDKB1 to ensure the transition into cytokinesis after the mitotic spindle is formed (Atkins and Cross 2018). In addition to the induction of cell division above, the control of cell division activity during the S/M phase is an important aspect of cell cycle regulation. This pathway is governed by the retinoblastoma complex (RB)–DP1–E2F which does not control timing of S/M but is required for appropriate coupling of cell division number and cell cycle transitions to cell size. The D-cyclin-dependent RB kinase CDKG1 is limiting for cell division number and is produced just prior to S/M phase with an abundance that positively correlates with mother cell-size. CDKG1-D cyclin along with other S/M kinases phosphorylate RB, inactivating its repressor function, and enabling the positive regulators of cell division, chromatin-bound heterodimeric DP and E2F subunits, to activate cell division (Olson *et al.* 2010; Li *et al.* 2016; Cross 2020). This mechanism ensures that larger mother cells will divide more times than smaller mother cells and allows size homeostasis to be maintained. Notably, unlike CDKA1, the RB–DP1–E2F complex does not control the timing of cell division nor the transcription of most S/M genes, but rather participates in cell size regulation (Fang *et al.* 2006; Cross 2020).

As reported previously, the expression of genes encoding CDKA1, CYCB1, CDKB1, CYCA1, and CYCAB1 peaks at ZT10-12 of WT cells (Zones *et al.* 2015). We confirmed this expression pattern for the WT lines, as well as for *cht7* (Fig. 6b). This result indicates that CHT7 does not act upstream of CDKA1 to regulate the S/M program, although some of the regulatory targets of both proteins seemed to overlap. However, our data also showed that genes for CYCA1, CYCB1, and CDKB1 were derepressed in *cht7* at noncell dividing timepoints such as ZT0, ND6-36, and NR6 (Fig. 6b). This pattern is similar to other CHT7-regulated S/M phase genes (Figs. 4 and 5), implying that the abnormal S/M program in *cht7* might be caused by the misregulated expression of *CYCB1* and *CDKB1* among other relevant genes. Interestingly, the expression of most of the genes encoding APC components was only slightly affected in *cht7* (Fig. 6c), indicating these genes may be subject to a different regulatory pathway than CYCB1 and CDKB1. Furthermore, although the APC activators CDC20 and CDC20 homolog 1 (CDH1) (Cross and Umen 2015) were highly induced in *cht7*, expression of genes encoding APC components were only slightly affected (Fig. 6c), indicating they are possibly subject to a different regulatory pathway than CYCB1 and CDKB1. Lastly, our results showed that there was no impact of the *cht7* mutation on the transcript levels of genes encoding the RB–DP–E2F complex- or CYCD–CDKG1 (Fig. 6, d and e). However, we cannot exclude the possibility that the protein activity of CHT7 is directly affected by the RB-mediated pathway given that a subfraction of CHT7 has been shown to associate with RB during the cell division cycle (Takeuchi *et al.* 2020a).

### Nitrogen starvation-induced cell wall synthesis in the cht7 mutant

**Fig. 7.**
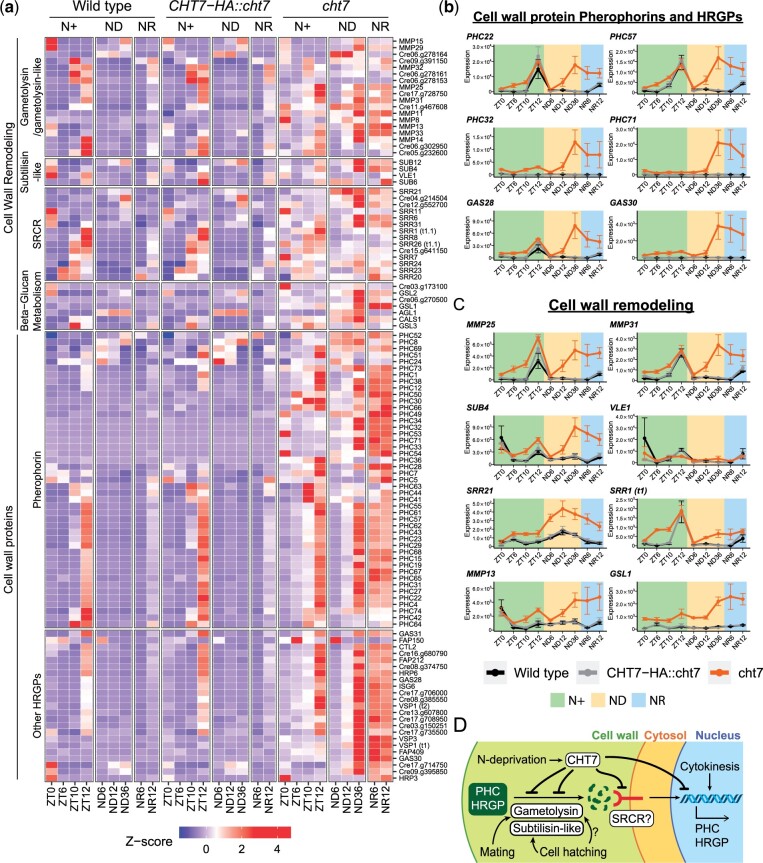
Misregulated cell wall-associated genes in the *cht7* mutant. a) *Z*-score heatmap of cell wall remodeling and biosynthetic genes. Genes that were differentially expressed (log_2_-fold change > 1, *P-*adj < 0.05) in *cht7* at ≥3 timepoints are presented. (SRCR) Scavenger receptor cysteine-rich. b, c) Expression of representative genes in *cht7* (orange), WT (black), and *CHT7-HA::cht7* (gray) across all timepoints. *Y*-axis: normalized read count. PHC, pherophorin; MMP, matrix metalloprotease; SUB, subtilisin-like protease; SRR, scavenger receptor cysteine-rich; GSL1, Gibberellin Stimulated-Like protein 1 (Callose synthase); VLE1, vegetative lytic enzyme 1; GAS28, Gamete-specific 28. d) Working model of CHT7-meidated cell wall regulation in Chlamydomonas. See also Supplementary Table 3.

In addition to cell division-related processes, we observed that the term GO:006075, defined as (1–3) beta-D-glucan biosynthetic process, was also enriched in the *cht7*-specific upregulated DEGs (Fig. 3d). Further analysis showed that 7 of the 11 annotated genes under this GO term were upregulated in *cht7* at more than 3 timepoints across all conditions (Supplementary Table 3). The GSL1 gene (Cre04.g214650; Callose synthase gene) was one of the DEGs that was highly transcribed in *cht7* at all timepoints, especially after ND12 and during N refeeding (Fig. 7c). Callose is a component of the cell wall in seed plants, and GSL8 in *Arabidopsis* was found to be essential for cell plate formation during cytokinesis (Chen *et al.* 2009; Chen and Kim 2009). Although cell walls are structurally different in Chlamydomonas and seed plants, callose is present at least in the primary wall of zygotes of *Chlamydomonas monoica* (Salanga and Van Winkle-Swift 2001). Hence the misregulation of the presumed GSL1 gene in *cht7* suggested that CHT7 may also affect aspects of cell wall biosynthesis in Chlamydomonas at certain stages of the cell cycle. To test this hypothesis, we examined the *cht7*-upregulated DEGs for other genes that might encode proteins with roles in cell wall-related processes. A group of genes encoding hydroxyproline-rich glycoproteins (HRGPs), the major cell wall component of Chlamydomonas (Cronmiller *et al.* 2019), fell into this category. Many of the *cht7*-induced HRGP genes belong to the pherophorin (PHC) subfamily of HRGPs that was originally named for its C-terminal similarity to the sex-inducing pheromone of *Volvox carteri* (Sumper *et al.* 1993; von der Heyde and Hallmann 2020). We identified 109 genes potentially encoding cell-wall-associated HRGPs, including 67 PHCs, in the Chlamydomonas genome v5.6, and 68 of the respective genes (including those for 45 pherophorins) were upregulated in *cht7* at more than 3 timepoints (Supplementary Table 3). The *Z*-score heatmap of these cell wall protein-encoding genes revealed that most of them were highly induced in *cht7* after 12 h of N deprivation (Fig. 7, a and b).

To better understand how CHT7 could modulate the expression of genes encoding PHCs and other HRGPs, we considered 2 instances of cell wall biosynthesis in Chlamydomonas that have been previously reported. The first one is the biosynthesis of new daughter cell walls following nuclear division. A transcriptomic study reported that the increase in expression of some PHC encoding genes was highly correlated with the S/M phase of the cell cycle and dependent on the kinases CDKA1 and CDKB1(Tulin and Cross 2015). For example, the gene for GAS28, a cell wall HRGP, was not transcribed in the temperature-arrested *cdkb1* mutant (Hoffmann and Beck 2005; Tulin and Cross 2015; Cronmiller *et al.* 2019). Our data are in agreement that some genes encoding PHCs and HRGPs, including GAS28, were expressed at ZT12 in all 3 cell strains when cell division takes place, and these genes were further induced in *cht7* following N deprivation (Fig. 7, a and b). Together, these findings imply that the expression of some cell wall genes might be suppressed by the same CHT7-mediated regulatory mechanism as other S/M phase genes above, and that CHT7 might play a role opposite to mitotic CDKs which positively regulate the expression of cell wall genes.

In addition to the necessity for cell wall synthesis after cytokinesis, cell wall biosynthesis can also be triggered by insults to the integrity of the cell wall (Cronmiller *et al.* 2019). Previous research has shown that exposure of cells to gametolysin, a cell wall peptidase normally produced during gametic fusion, induces the expression of cell wall genes necessary for vegetative growth, of which some are also active in the early zygote (Joo *et al.* 2017). To test if the abnormal induction of cell wall genes in *cht7* is correlated with a misregulated cell wall degradation signal, we examined the expression of cell-wall proteases that might be involved in cell wall remodeling. In the Chlamydomonas genome v5.6, cell wall peptidases such as gametolysin and subtilisin-like proteases are mostly encoded by the metalloproteases (MMP) and subtilisin (SUB) gene families, respectively (Luxmi *et al.* 2018). We also included genes encoding proteins containing the scavenger receptor cysteine rich (SRCR) domain in our analysis because it has been proposed that SRCR proteins might be involved in the peptidase-mediated cell-wall biosynthesis in Chlamydomonas (Wheeler *et al.* 2008; Cronmiller *et al.* 2019) (see the full list in Supplementary Table 3). Our results showed that 36 genes encoding MMPs, SUBs, and SRCR proteins were upregulated in *cht7* at more than 3 timepoints, including ND12, 36, and NR6 (Fig. 7, a and c). Notably, the expression patterns of these cell wall peptidases and SRCR protein-encoding genes were very similar to those of cell wall genes (Fig. 7b), implying that they belong to the same cell wall remodeling/biosynthetic pathway in Chlamydomonas that appears suppressed by CHT7, especially under long-term ND conditions (Fig. 7d). We also found that 30 of these *cht7*-induced genes encoding cell wall remodeling proteins, such as MMP25, MMP31, SUB4, and SRR21, were normally transcribed at ZT0, 10, or 12 in all 3 lines (Fig. 7c), suggesting that they have an additional function in cell wall reconfiguration during cell division other than gametic fusion. The aberrant transcription profiles in *cht7* are consistent with previous observations of misshapen *cht7* mutant cells under ND and NR conditions (Takeuchi *et al.* 2020b).

### Induction of genes encoding uncharacterized protein kinase families in cht7

In addition to the biological processes mentioned above, the GO term GO:0006468 Protein Phosphorylation, was also found to be enriched among proteins encoded by genes that were derepressed in the *cht7* mutant (Fig. 3d). In Chlamydomonas, 778 genes have been annotated to encode proteins with this GO term, and 162 of them were upregulated in *cht7* at more than 1 timepoint. To narrow the scope of our analysis to the most unexplored aspects, we focused on 46 previously uncharacterized, kinase-encoding genes that were induced in *cht7* at more than 5 timepoints (Supplementary Table 3). In order to categorize these kinases and infer possible involvement in signaling pathways, we performed a phylogenetic analysis of the kinase domain of 43 kinases of unknown function with 122 known kinases with characterized functions from Chlamydomonas and various plant species, including Mitogen-activated protein kinase (MAPK) cascade kinases (MAPKs, MAP2Ks, and MAP3Ks) (Krysan *et al.* 2002; Yu *et al.* 2007; Doczi *et al.* 2012; Fei *et al.* 2017), Calcium-dependent protein kinases (CDPKs, CPKs, CIPKs, CCaMKs) (Cheng *et al.* 2002; Tirichine *et al.* 2006; Yu *et al.* 2007), NEVER IN MITOSIS A (NIMA)-related kinases (CNKs, NEKs, FA2) (Bradley *et al.* 2004; Mahjoub *et al.* 2004; Bradley and Quarmby 2005; Vigneault *et al.* 2007), Aurora-like kinases (ALKs) (Pan *et al.* 2004), and a few other kinases known to be associated with flagella and basal bodies (GSK3 and CDKL1) (Wilson and Lefebvre 2004; Tam *et al.* 2007) (Supplementary Table 4).

**Fig. 8.**
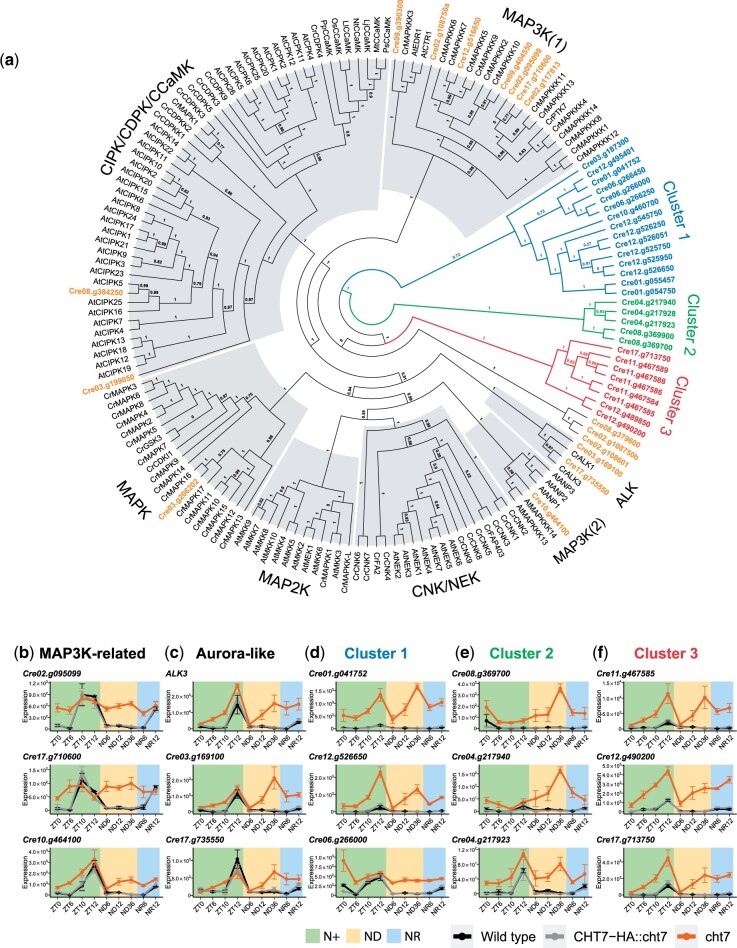
Analysis of protein kinase families misregulated in *cht7*. a) ML phylogeny of the kinase domain of 43 protein kinases encoded by genes that are differentially expressed (log_2_-fold change >1, *P-*adj < 0.05) in *cht7* at ≥5 timepoints along with kinase domains retrieved from 122 known protein kinases. Confidence of branches was tested by aLRT branch support. Refer to *Materials and methods* for details. Cr, *Chlamydomonas reinhardtii*; At, *Arabidopsis*; Lt, *Lotus japonicus*; Mt, *Medicago truncatula*; Ps, *Pisum sativum*; Nt, *Nicotiana tabacum*; Ll, *Lilium longiflorum*; Os, *Oryza sativa*; Pp, *Physcomitrella patience*; CCaMK, Ca2+/CaM-dependent protein kinase; CIPK, CBL-interacting protein kinase; CDK and CPK, calcium-dependent protein kinases; CNK, Chlamydomonas NIMA-related kinase; NEK, NIMA-related Kinase; GSK3, glycogen synthase kinase 3. b–f) Expression of genes encoding MAPK3K-related kinases (b), ALKs (c), and cluster 1–3 potential protein kinases (d–f) in *cht7* (orange), WT (black), and *CHT7-HA::cht7* (gray) across all timepoints. *Y*-axis: normalized read count. See also Supplementary Data 1, Tables 3 and 4.

The phylogenetic analysis in Fig. 8a indicated that some *cht7*-induced kinases were related to known kinase families such as MAPK, MAP3K, CIPK, and ALK (Fig. 8a; highlighted in orange). For instance, the Cre17.g10600- and Cre02.g095099-encoding kinases were closely related to Chlamydomonas MAPKKK11 and MAPKKK13 [Fig. 8a; MAP3K (1)], while the Cre10.g464100-encoding kinase was related to an *Arabidopsis*-specific MAP3K family [Fig. 8a; MAP3K (2)]. In WT and *CHT7-HA::cht7* cells, these MAP3K-related kinases-encoding genes were mainly expressed at ZT10 (Cre17.g10600), ZT12 (Cre02.g095099), or both (Cre10.g464100), but were strongly derepressed in *cht7* at other timepoints (Fig. 8b). We also identified 2 kinases encoded by Cre03.g169100 and Cre17.g735550 that were grouped with the ALK family of Chlamydomonas on the phylogenetic tree (Fig. 8a; ALK). Genes encoding these ALK-related proteins and ALK3 were expressed at ZT12 in all strains but greatly derepressed in *cht7* when the cells were deprived of N, and should have ceased division, especially at ND36 (Fig. 8c). Lastly, we identified a large group of CHT7-regulated kinase-encoding genes (*n* = 31) that did not show obvious similarities to any of the reference sequences except for the kinase domain, and 3 clusters were formed based on their sequence similarities (Fig. 8a; highlighted in blue, green, and red). In WT and *CHT7HA::cht7*, most of the genes in the 3 clusters were slightly increased in transcript levels at ZT12 when cells are in the S/M phase, suggesting a potential role of these kinases during the cell cycle (Fig. 8, d–f). The expression profiles of the genes for these kinases indicated that most cluster 1 (*n* = 17) and cluster 3 (*n* = 8) kinase genes were highly expressed in *cht7* at timepoints besides ZT0 and ND6 and peaked at ZT12 and ND36 (Fig. 8, d and f). Genes encoding cluster 2 kinases (*n* = 5) were induced in *cht7* at all timepoints and their expression particularly peaked at ND36 (Fig. 8e). In summary, the identification of these potential CHT7-regulated kinase families suggests an uncharacterized, kinase-mediated cell cycle-related pathway in addition to the known kinase-mediated signaling pathways in Chlamydomonas.

## Discussion

Based on our previous analyses, the CHT7 protein in the green alga Chlamydomonas has been proposed to be involved in the regulation/repression of transcriptional programs particularly relevant during G0 and the transition between G0 and G1 states. To further test this hypothesis, we performed a comprehensive RNA-seq experiment using photoautotrophic cell cultures grown under synchronizing conditions in bioreactors with turbidostatic control allowing us to examine transcriptomic changes caused by the absence of CHT7 at key stages of the cell division cycle and ND-induced quiescence (Fig. 2a). A key prerequisite was that, as previously observed (Takeuchi *et al.* 2020a), most *cht7* cells grown under N+ conditions proceed through the cell division cycle on the same time scale as the WT and *CHT7-HA::cht7*, and the overall growth and viability of the cell culture was not noticeably affected by the absence of CHT7 under N+ growth conditions. However, probing the DNA content of individual cells under these conditions, we observed a subgroup of *cht7* cells with ≥2C DNA content across all noncell division timepoints (Fig. 1, a and b; Supplementary Fig. 1). Moreover, light microscopy at ZT6 revealed that compared with controls, the *cht7* mutant had a more diverse cell population even under N+ growth conditions. For instance, type 2 cells, defined as round and nonflagellated cells, were nearly exclusively found in *cht7* (Fig. 1c and d). The phenotypes of the type 2 *cht7* cells resemble those of WT during prophase, with their DNA duplicated, chromosomes condensed, and flagella resorbed (Cross and Umen 2015). While we were unable to determine whether the type 2 cells of *cht7* were the ≥2C DNA cells described above, overall, these observations suggest that the loss of CHT7 in the mutant allows a fraction of *cht7* cells to escape prematurely the G1 phase of the cell division cycle. Therefore, based on DNA content as a criterion, the *cht7* cell cultures appear to be less synchronized compared with the control strains. In fact, genes encoding proteins involved in cell division, especially DNA replication and mitotic entry, were abnormally and overwhelmingly more highly expressed in *cht7* at different times throughout the cell cycle in all conditions compared with the WT. A plausible interpretation of these results is that CHT7 is generally needed to suppress expression of genes required for the entry into cell division (Figs. 4 and 5) and, therefore, its absence could affect the level of synchrony of the culture. It remains to be determined whether spindle formation is abnormally activated under N+ growth of *cht7* cells as well.

Although abnormal cell phenotypes of at least a subpopulation of *cht7* cells were observed under N+ growth conditions, the altered transcriptional profiles raise the question of why *cht7* cells do not exhibit more severe cell cycle defects under normal N+ growth as they do when they are in ND and NR conditions. One plausible reason is that the phenotypes observed for ND and NR *cht7* cells are the result of cumulative effects when cell growth slows under ND and thus are not seen in the rapidly dividing N+ cell cultures. The loss of CHT7, although resulting in a widely misregulated transcriptome in *cht7* even under N+ conditions, did not have an immediate impact on cell cycle progression as observed for known cell cycle disrupted mutants such as *cdkb1* (impaired spindle) and *cdka1* (delayed S/M initiation). This could be because changes in transcript levels in *cht7* are under a threshold that does not immediately affect actual protein abundance or activity, or due to the fact that only a subset of cell cycle genes was induced (Fig. 6; Supplementary Table 3). For example, APC is a conserved cell cycle regulatory complex in eukaryotes and is required for mitotic exit by promoting chromosome segregation and mediating the degradation of mitotic cyclins (Cross and Umen 2015). The Chlamydomonas mutant lacking APC3, *cdc27-6*, arrests at metaphase, indicating the essential role of APC in the completion of mitosis (Atkins and Cross 2018). The genes encoding the APC components were expressed normally in *cht7* at all ZT timepoints (Fig. 6), although some were misregulated during ND. Accordingly, although DNA replication and some mitotic events are abnormally activated in *cht7* cells during G0 and G1, critical genes for cell division in *cht7* may allow the S/M phase to proceed when the cells are not under metabolic stress. However, during an extended period following ND, the imbalanced expression of many cell cycle genes in *cht7* and the abnormally activated S/M phase processes may eventually lead to irreversible metabolic outcomes, abnormal number of nuclei and arrested cell division (Takeuchi *et al.* 2020b).

Another possible reason for the relatively mild phenotype of N+ *cht7* cells compared with ND and NR cells is that CHT7 might govern additional pathways during quiescence. For instance, the apparent increase in cell wall synthesis or cell wall remodeling in *cht7* at ND time points might be responsible for the cellular phenotypes exhibited in N-deprived *cht7* cells (Fig. 7). Of note, previous studies using the cell wall-less (cw−) and cell-walled (cw+) *cht7* mutant strains observed different phenotypes: the viability of cw+ *cht7* cells declined to 40–50% following an extended period of ND, while the cw− *cht7* cell did not exhibit a loss of viability (Tsai *et al.* 2014; Takeuchi *et al.* 2020b). The basis of the differential viability may be deduced from the data reported here: derepression of the cell wall pathways may contribute to the increased mortality of the cw+ *cht7* mutant during quiescence since the Chlamydomonas cell wall would be a major sink for cellular nitrogen and energy (Lang and Chrispeels 1976); continuously active cell wall remodeling might be harmful to the cell, especially under N-limited conditions. Based on the insights from our current analysis, a future study analyzing the cell wall structure and remodeling of *cht7* cells should provide answers to the question of differences in apparent viability between the cw+ and cw− *cht7* mutant strains.

In addition to the genes affected by CHT7 that have defined functions, we identified several previously uncharacterized genes that are misregulated in *cht7* and may encode kinases related to MAPK cascade kinases or ALKs (Fig. 8a). These genes are highly expressed at ZT10-12 (Fig. 8, b and c), suggesting they may function in cell division. Three uncharacterized kinase families were identified in our dataset (Fig. 8, a, d–f), whose genes were strongly derepressed in *cht7* compared with the 2 controls. These should provide promising candidates for further investigations of cell division regulation in Chlamydomonas.

Finally, this study aimed to provide insight into the possible role of CHT7 in cell cycle regulatory mechanisms of Chlamydomonas. We showed that CHT7 is required for the repression of specific groups of cell cycle genes not only during quiescence, but also in the normal cell division cycle (Fig. 3). As mentioned previously, CHT7 impacts the expression of S-phase and mitosis-related genes, however, the mechanism by which CHT7 represses the expression of these genes remains to be determined. As reported previously, CHT7-based ChIP-Seq analysis did not detect a direct interaction between CHT7 and chromosomal DNA (Takeuchi *et al.* 2020b). Subsequently, it has been shown that the presumed DNA-binding domain (CHC domain) of the CHT7 protein was not required for its function during N deprivation and following N resupply (Takeuchi *et al.* 2020b). Together, these results strongly suggested that CHT7 does not act as a canonical transcriptional repressor by binding directly to DNA through its CHC domain. However, one could postulate that CHT7 interacts with other transcriptional repressors that directly bind to the respective target genes. The identification of the primary targets of CHT7 in complex with other protein factors will be needed to clarify this point. The HA-CHT7 protein having the deletion of the disordered region would serve as a negative control for such protein–protein interaction assay since that directed deletion creates a null mutation (Takeuchi *et al.* 2020b). This next step in the analysis of CHT7 function would also help resolve uncertainties affecting the interpretation of the current data such as the possibility that the induced expression of cell cycle genes in *cht7* was a compensatory effect due to other factors such as compromised central metabolism or partial loss of viability, or that other genes disrupted in the *cht7* mutant contribute to the observed phenotypes.

Aside from these uncertainties, our current data support a hypothesis that assigns CHT7 a role in the regulation of the cell cycle opposite to mitotic CDKs in Chlamydomonas. It has been shown that CDKA1 participates in the initiation of S-phase and cell division, while CYCB1–CDKB1 plays a more important role in promoting the entry into mitosis (Atkins and Cross 2018). Both proteins are in the nucleus and function by protein phosphorylation affecting transcriptional activation of the respective genes. In addition, CDKA1 also induces the expression of *CYCB1* and *CDKB1* (Tulin and Cross 2015; Atkins and Cross 2018). In *cht7*, genes encoding CDKB1 and CYCB1 were derepressed in a similar pattern to other misregulated cell cycle genes, while the transcription of the CDKA1-encoding gene was unaffected (Fig. 6). This result raised the possibility that a CHT7 containing complex may affect the expression of similar genes as CDKA1, but negatively regulates their expression to inhibit cell division when not desirable. A double mutant analysis of *cht7* and *cdka1* asking whether *cht7* suppresses effects of *cdka1* would be helpful to clarify this point. The current work should inspire future efforts toward the identification of CHT7-associated transcription factors and functional characterization of previously unreported protein kinases to gain a more complete understanding of cell cycle regulation in Chlamydomonas.

## Data availability

The transcriptomic data including the raw and processed sequence reads are deposited to GEO (Gene Expression Omnibus; https://www.ncbi.nlm.nih.gov/geo/) under accession number GSE167405.

Supplemental material is available at *G3* online.

## Supplementary Material

jkac023_Figure_S1

jkac023_Figure_S2

jkac023_Supplemental_Data_S1

jkac023_Supplemental_Table_S1

jkac023_Supplemental_Table_S2

jkac023_Supplemental_Table_S3

jkac023_Supplemental_Table_S4

jkac023_Supplemental_Figure_Legends
