# Supplementary figures and images for: Chlamydomonas CHT7 is involved in repressing DNA replication and mitotic genes during synchronous growth
Source: G3 (Bethesda). 2022 Feb 7;12(3):jkac023. doi: 10.1093/g3journal/jkac023 (PMC8895990; doi:10.1093/g3journal/jkac023)

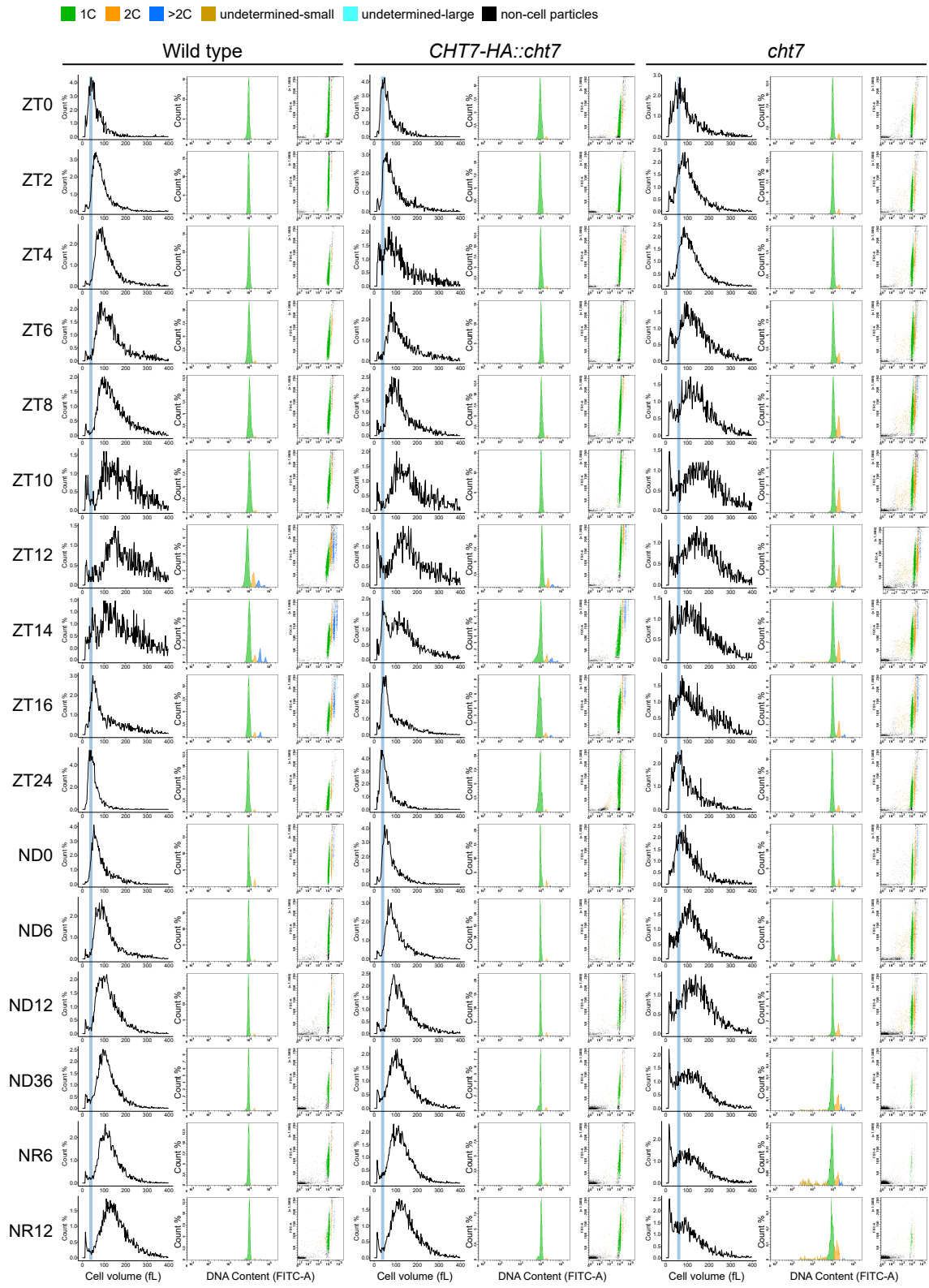

Supplement: jkac023_Figure_S1 [file jkac023_figure_s1.pdf]

GO Enrichment in up-regulated *cht7* DE genesp-value ■ < 0.01 ■ 0.01–0.05 ■ > 0.05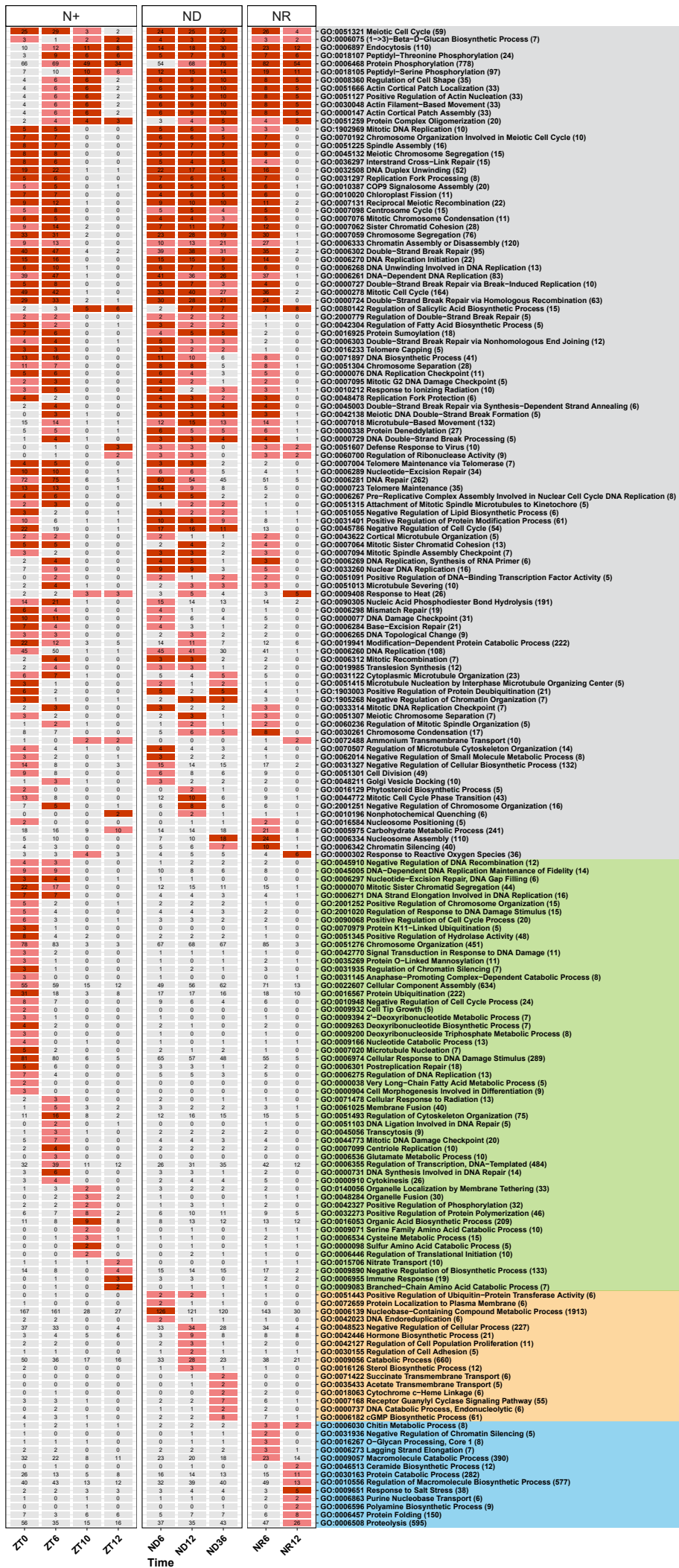

Supplement: jkac023_Figure_S2 [file jkac023_figure_s2.pdf]
