# Supplementary material for: Chlamydomonas CHT7 is involved in repressing DNA replication and mitotic genes during synchronous growth
Source: G3 (Bethesda). 2022 Feb 7;12(3):jkac023. doi: 10.1093/g3journal/jkac023 (PMC8895990; doi:10.1093/g3journal/jkac023)
Supplement: jkac023_Supplemental_Data_S1 [file jkac023_supplemental_data_s1.docx]

....|....| ....|....| ....|....| ....|....| ....|....| ....|....| ....|....| ....|....| ....|....| ....|....|

10 20 30 40 50 60 70 80 90 100

CrCDPK9 ---------- ---------- ---------- ---------- ---------- ---------- ---------- ---------- ---------Y RVDKQIGKGA

AtCPK25 ---------- ---------- ---------- ---------- ---------- ---------- ---------- ---------- ---------Y NLGSKLGHGQ

AtCPK26 ---------- ---------- ---------- ---------- -RPQEEATM- -------KHS GGNQACY--- -------VLG QKTPSIRDLY SLGHKLGQGQ

AtCPK6 ---------- ---------- ---------- ---------- ---------- ---------- ---------- ---------- ---------Y TLSRKLGQGQ

AtCPK5 ---------- ---------- ---------- ---------- ---------- ---------- ---------- ---------- ---------Y TLSRKLGQGQ

AtCPK20 ---------- ---------- ---------- ---------- ---------- ---------- ---------- ---------- ---------Y SVGRKLGQGQ

AtCPK2 ---------- ---------- ---------- ---------- ---------- ---------- ---------- ---------- ---------Y SLGRKLGQGQ

AtCPK1 ---------- ---------- ---------- ---------- ---------- ---------- ---------- ---------- ---------Y SLGRKLGQGQ

AtCPK12 ---------- ---------- ---------- ---------- ---------- ---------- ---------- ---------- ---------Y FLGQVLGQGQ

AtCPK11 ---------- ---------- ---------- ---------- ---------- ---------- ---------- ---------- ---------Y LLGKKLGQGQ

AtCPK4 ---------- ---------- ---------- ---------- ---------- ---------- ---------- ---------- ---------Y LLGKKLGQGQ

AtCIPK7 ---------- ---------- ---------- ---------- ---------- ---------- ---------- ---------- ---------Y ELGRRLGSGS

AtCIPK4 ---------- ---------- ---------- ---------- ---------- ---------- ---------- ---------- ---------Y ELGRRLGSGS

AtCIPK22 ---------- ---------- ---------- ---------D SNSSESIIV- -------NV- ---------- -------TGD DNKSALFGKY DLGKLLGSGA

AtCIPK11 ---------- ---------- ---------- ---------- ---------- ---------- ---------- ---------- ---------Y ELGKLLGCGA

AtCIPK14 ---------- ---------- ---------- ---------- ---------- ---------- ---------- ---------- ---------Y EVGKLVGCGA

AtCIPK12 ---------- ---------- ---------- ---------- ---------- ---------- ---------- ---------- ---------Y EMGKLLGHGT

AtCIPK19 ---------- ---------- ---------- ---------- ---------- ---------- ---------- ---------- ---------Y EMGRLLGHGT

AtCIPK18 ---------- ---------- ---------- ---------- ---------- ---------- ---------- ---------- ---------Y ELGKLLGHGT

AtCIPK13 ---------- ---------- ---------- ---------- ---------- ---------- ---------- ---------- ---------Y EIGKLLGHGS

AtCIPK16 ---------- ---------- ---------- ---------- ---------- ---------- ---------- ---------- ---------Y NIGRLLGTGN

AtCIPK5 ---------- ---------- ---------- ---------- ---------- ---------- ---------- ---------- ---------Y EMGRLLGKGT

AtCIPK25 ---------- ---------- ---------- ---------- ---------- ---------- ---------- ---------- ---------Y EMGRLLGKGT

AtCIPK2 ---------- ---------- ---------- ---------- ---------- ---------- ---------- ---------- ---------Y EVGRLLGQGT

AtCIPK10 ---------- ---------- ---------- ---------- ---------- ---------- ---------- ---------- ---------Y DVGRLLGQGT

AtCIPK15 ---------- ---------- ---------- ---------- ---------- ---------- ---------- ---------- ---------Y EVGKFLGQGT

AtCIPK6 ---------- ---------- ---------- ---------- ---------- ---------- ---------- ---------- ---------Y ELGRLLGHGT

AtCIPK20 ---------- ---------- ---------- ---------- ---------- ---------- ---------- ---------- ---------Y ELGRLLGQGT

AtCIPK21 ---------- ---------- ---------- ---------- ---------- ---------- ---------- ---------- ---------Y EIGRTIGEGN

AtCIPK17 ---------- ---------- ---------- ---------- ---------- ---------- ---------- ---------- ---------Y ELGRTLGEGN

AtCIPK1 ---------- ---------- ---------- ---------- ---------- ---------- ---------- ---------- ---------Y ELGRTLGEGN

AtCIPK3 ---------- ---------- ---------- ---------- ---------- ---------- ---------- ---------R QQVKRRVGKY EVGRTIGEGT

AtCIPK9 ---------- ---------- ---------- ---------- ---------- ---------- ---------- ---------- ---------Y EMGRTLGEGS

AtCIPK23 ---------- ---------- ---------- ---------- ---------- ---------- ---------- ---------- ---------Y ELGRTLGEGT

AtCIPK8 ---------- ---------- ---------- ---------- ---------- ---------- ---------- ---------- ---------Y ELGRTIGEGT

AtCIPK24 ---------- ---------- ---------- ---------- ---------- ---------- ---------- ---------- ---------Y EVGRTIGEGT

CrMAPK7 ---------- ---------- ---------- ---------- ---------- ---------- ---------- ---------- ---------Y KVVKQLGDGT

CrMAPK9 ---------- ---------- ---------- ---------- ---------- ---------- ---------- ---------- ---------Y EIISIVGEGA

CrMAPK13 ---------- ---------- ---------- ---------- ---------- ---------- ---------- ---------- ---------Y VYLRTIAEGS

Cre03.g206202 ---------- ---------- ---------- ---------- ---------- ---------- ---------- ---------- ---------Y EPIAQIGTGA

CrMAPK14 ---------- ---------- ---------- ---------- ---------- ---------- ---------- ---------- ---------Y EFLSRTGEGA

CrMAPK17 ---------- ---------- ---------- ---------- ---------- ---------- ---------- ---------- ---------- ----------

CrMAPK16 ---------- ---------- ---------- ---------- ---------- ---------- ---------- ---------- ---------Y TYIQLLGRGT

CrMAPK15 ---------- ---------- ---------- ---------- ---------- ---------- ---------- ---------- ---------M APFGNAGIGT

CrMAPK12 ---------- ---------- ---------- ---------- ---------- ---------- ---------- ---------- ---------Y TYVSTLGEGA

CrMAPK10 ---------- ---------- ---------- ---------- ---------- ---------- ---------- ---------- ---------- ----------

CrMAPK11 ---------- ---------- ---------- ---------- ---------- ---------- ---------- ---------- ---------- ----------

CrGSK3 ---------- ---------- ---------- ---------- ---------- ---------- ---------- ---------- ---------Y STDRVVGNGS

CrCDKI1 ---------- ---------- ---------- ---------- ---------- ---------- ---------- ---------- ---------Y RIIRRLGQGA

CrMAPK5 ---------- ---------- ---------- ---------- ---------- ---------- ---------- ---------- ---------Y EIQQKLGKGA

CrMAPK8 ---------- ---------- ---------- ---------- ---------- ---------- ---------- ---------- ---------Y LPIKPIGKGA

CrMAPK6 ---------- ---------- ---------- ---------- ---------- ---------- ---------- ---------- ---------Y VPIKAIGKGA

CrMAPK3 ---------- ---------- ---------- ---------- ---------- ---------- ---------- ---------- ---------Y VPIKAIGKGA

CrMAPK2 ---------- ---------- ---------- ---------- ---------- ---------- ---------- ---------- ---------Y TIKEVIGKGS

CrMAPK4 ---------- ---------- ---------- ---------- ---------- ---------- ---------- ---------- ---------Y TVHSLIGKGS

CrCDPK5 ---------- ---------- ---------- ---------- ---------- ---------- ---------- ---------- ---------F NLIKRLGRGG

CrCDPK4 ---------- ---------- ---------- ---------- ---------- ---------- ---------- ---------- ---------- ----------

CrCDPK3 ---------- ---------- ---------- ---------- ---------- ---------- ---------- ---------- ---------F KFGQQINKGQ

PpCCaMK ---------- ---------- ---------- ---------- ---------- ---------- ---------- ---------- ---------F HVGPVLGTGG

NtCCaMK ---------- ---------- ---------- ---------- ---------- ---------- ---------- ---------- ---------Y EVTDILGRGG

LjCCaMK ---------- ---------- ---------- ---------- ---------- ---------- ---------- ---------- ---------Y EISEILGRGG

MtCCaMK ---------- ---------- ---------- ---------- ---------- ---------- ---------- ---------- ---------Y EVSEILGRGG

PsCCaMK ---------- ---------- ---------- ---------- ---------- ---------- ---------- ---------- ---------Y EVSEILGRGG

LlCCaMK ---------- ---------- ---------- ---------- ---------- ---------- ---------- ---------- ---------Y EVVDVLGKGG

OsCCaMK ---------- ---------- ---------- ---------- ---------- ---------- ---------- ---------- ---------Y EVVDVLGRGG

Cre03.g199050 ---------- ---------- ---------- ---------- ---------- ---------- ---------- ---------- ---------A QAVAVLGAGG

CrCDPKK1 ---------- ---------- ---------- ---------- ---------- ---------- ---------- ---------- ---------Y LVVRFLGRGA

CrCDPKK2 ---------- ---------- ---------- ---------- ---------- ---------- ---------- ---------- ---------Y LIIKDLGKGA

AtMKK10 ---------- ---------- ---------- ---------- ---------- ---------- ---------- ---------- ---------L EKLSVLGQGS

AtMKK4 ---------- ---------- ---------- ---------- ---------- ---------- ---------- ---------- ---------L VRGNRIGSGA

AtMKK5 ---------- ---------- ---------- ---------- ---------- ---------- ---------- ---------- ---------L ERVNRIGSGA

AtMKK8 ---------- ---------- ---------- ---------- ---------- ---------- ---------- ---------- ---------L DRISVLGSGN

AtMKK7 ---------- ---------- ---------- ---------- ---------- ---------- ---------- ---------- ---------V EKLHVLGRGS

AtMKK9 ---------- ---------- ---------- ---------- ---------- ---------- ---------- ---------- ---------L EKLNVLGCGN

CrMAPKK1 ---------- ---------- ---------- ---------- ---------- ---------- ---------- ---------- ---------I RILKKLGQGA

AtMKK3 MAALEELKKK LSPLFDAEK- ----GFSSSS SLDPNDSYLL SDGGTVNLLS RSYGVYNFNE LGLQKCTSSH VDESESSETT YQCASH--EM RVFGAIGSGA

CrMAPKK-L ---------- ---------- ---------- ---------- ---------- ---------- ---------- ---------- --------DF EDLCVIGQGS

AtMKK6 ---------- ---------- ---------- ---------- ---------- ---------- ---------- ---------- ---------L ETVKVIGKGS

AtMEK1 ---------- ---------- ---------- ---------- ---------- ---------- ---------- ----PIEPLD NQLSLA--DL EVIKVIGKGS

AtMKK2 ---------- ---------- ---------- ---------- ---------- ---------- ---------- --------AD DQLSLS--DL DMVKVIGKGS

AtMAPKKK13 ---------- ---------- ---------- ---------- ---------- ---------- ----TCSSSL ML-------- -SSPSS--FW VRGACIGRGC

AtMAPKKK14 ---------- ---------- ---------- ---------- ---------- ---------- ---------- ---------- ---------W IRGSCVGRGC

Cre10.g464100 ---------- ---------- ---------- ---------- ---------- ---------- ---------- ---------- ---------W QRGRQIGQGA

AtANP2 ---------- ---------- ---------- ---------- ---------- ---------- ---------- ---------- ---------- ----------

AtANP1 ---------- ---------- ---------- ---------- ---------- ---------- ---------- ---------- ---------W RKGQLIGRGA

AtANP3 ---------- ---------- ---------- ---------- ---------- ---------- ---------- ---------- ---------W RKGELIGCGA

Cre03.g169100 ---------- ---------- ---------- ---------- ---------- ---------- ---------- ---------- ---------I KSRAELYKGS

Cre17.g735550 ---------- ---------- ---------- ---------- ---------- ---------- ---------- ---------- ---------Y RITNTLYSGY

CrALK3 ---------- ---------- ---------- ---------- ---------- ---------- ---------- ---------- ---------Y QLGDKLYTGY

CrALK1 ---------- ---------- ---------- ---------- ---------- ---------- ---------- ---------- ---------F HLLRKVGSGY

CrMAPKKK3 ---------- ---------- ---------- ---------- ---------- ---------- ---------- ---------- ---------L TLGQRIGIGS

AtEDR1 ---------- ---------- ---------- ---------- ---------- ---------- ---------- ---------- ---------L VIAERIGLGS

AtCTR1 ---------- ---------- ---------- ---------- ---------- ---------- ---------- ---------- ---------L NIKEKIGAGS

CrCNK9 ---------- ---------- ---------- ---------- ---------- ---------- ---------- ---------- ---------Y TQLRQIGKGA

AtNEK7 ---------- ---------- ---------- ---------- ---------- ---------- ---------- ---------- ---------Y HVVEQVRRGK

AtNEK5 ---------- ---------- ---------- ---------- ---------- ---------- ---------- ------KISE TASKMD--DY EVVEQIGRGA

AtNEK6 ---------- ---------- ---------- ---------- ---------- ---------- ---------- ---------- ---------Y ELMEQIGRGA

AtNEK4 ---------- ---------- ---------- ---------- ---------- ---------- ---------- ---------- ---------Y EVLEQIGKGS

AtNEK1 ---------- ---------- ---------- ---------- ---------- ---------- ---------- ---------- ---------Y EFLEQIGKGS

AtNEK2 ---------- ---------- ---------- ---------- ---------- ---------- ---------- ---------- ---------Y EVLEQIGKGS

AtNEK3 ---------- ---------- ---------- ---------- ---------- ---------- ---------- ---------- ---------Y EVLEQIGKGS

CrCNK7 ---------- ---------- ---------- ---------- ---------- ---------- ---------- ---------- ---------Y QLDRLLGRGK

CrCNK6 ---------- ---------- ---------- ---------- ---------- ---------- ---------- ---------- ---------Y DVQKPVGKGG

CrCNK4 ---------- ---------- ---------- ---------- ---------- ---------- ---------- ---------- ---------F IIKEKIGSGS

CrFA2 ---------- ---------- ---------- ---------- ---------- ---------- ---------- ---------- ---------D YELQYIDKGS

CrCNK1 ---------- ---------- ---------- ---------- ---------- ---------- ---------- ---------- ---------F KVHKLLGKGS

CrCNK2 ---------- ---------- ---------- ---------- ---------- ---------- ---------- ---------- ---------F KVLKFLGKGS

CrCNK8 ---------- ---------- ---------- ---------- ---------- ---------- ---------- ---------- ---------Y QELKLIGKGT

CrCNK5 ---------- ---------- ---------- ---------- ---------- ---------- ---------- ---------- ---------Y LDLTAIGQGQ

CrFAP403 ---------- ---------- ---------- ---------- ---------- ---------- ---------- ---------- ---------Y IRGKVLGKGS

CrCNK3 ---------- ---------- ---------- ---------- ---------- ---------- ---------- ---------- ---------- ----------

CrCDPKK3 ---------- ---------- ---------- ---------- ---------- ---------- ---------- ---------- ---------Y IVIKHLGSGT

CrMAPK1 ---------- ---------- ---------- ---------- ---------- ---------- ---------- ---------- ---------Y RLVAKKGEGT

Cre08.g379600 ---------- ---------- ---------- ---------- ---------- ---------- ---------- ---------- ---------L RFIQQIGAGG

Cre02.g108601 ---------- ---------- ---------- ---------- ---------- ---------- ---------- ---------- ---------- ----------

Cre02.g108750a ---------- ---------- ---------- ---------- ---------- ---------- ---------- ---------- ---------L RLKTLLGRGG

Cre17.g713750 ---------- ---------- ---------- -------WR- ---------- ---------- ---------- ---------- ---------- -SEGQIGRGG

Cre11.g467586 ---------- ---------- ---------- -------WR- ---------- ---------- ---------- ---------- ---------- -SEGKIGRGG

Cre11.g467588 ---------- ---------- ---------- -------WR- ---------- ---------- ---------- ---------- ---------- -SEGKIGRGG

Cre11.g467589 ---------- ---------- ---------- -------WR- ---------- ---------- ---------- ---------- ---------- -SEGKIGRGG

Cre11.g467584 ---------- ---------- ---------- -------WR- ---------- ---------- ---------- ---------- ---------- -SEGKIGRGG

Cre11.g467585 ---------- ---------- ---------- -------WR- ---------- ---------- ---------- ---------- ---------- -SEGKIGRGG

Cre12.g489850 ---------- ---------- ---------- -------LE- ---------- ---------- ---------- ---------- ---------- -ALVPFSKGG

Cre12.g490200 ---------- ---------- ---------- -------YE- ---------- ---------- ---------- ---------- ---------- -GGGLLGQGA

Cre08.g384250 ---------- ---------- ---------- ---------- ---------- ---------- ---------- ---------- ---------- ----------

Cre04.g217940 ---------- ---------- ---------- -------IR- ---------- ---------- ---------- ---------- ---------- -LKRHLGQGA

Cre04.g217923 ---------- ---------- ---------- -------IR- ---------- ---------- ---------- ---------- ---------- -LKRHLGQGA

Cre04.g217928 ---------- ---------- ---------- -------IR- ---------- ---------- ---------- ---------- ---------- -LKRHLGQGA

Cre08.g369700 ---------- ---------- ---------- -------WQ- ---------- ---------- ---------- ---------- ---------- -DPASMLHGG

Cre08.g369900 ---------- ---------- ---------- -------WQ- ---------- ---------- ---------- ---------- ---------- -DPASMLHGG

Cre03.g187300 ---------- ---------S SCLGGSGSGD GGSAGRAWAI EDGVEEMGE- --------GE HDGQAC---- ---------G RNLKISR-CA SFSSGFSAGD

Cre01.g041752 ---------- --------RS SC-DGSGSGT GSEVEEGPKV ESPVSPSLV- --------SS SDEVAPSRDA ---------A HQGPMPT-CR VVSKLSCTDP

Cre12.g495401 ---------- --------RS SC-DGSGSGT GSEVEEGPKV ESPAASSLV- --------SS ADEEAPSRDA ---------A HQGPMPT-CR VVSKLSCADP

Cre06.g266450 ---------- ---------- ---------- ---------- ---------- ---------- ---------- ---------- --------CE PVKE-LGKGG

Cre06.g266000 ---------- ---------- ---------- ---------- ---------- ---------- ---------- ---------- --------CE PVQE-LGRGG

Cre06.g266250 ---------- ---------- ---------- ---------- ---------- ---------- ---------- ---------- --------CE PVQE-LGRGG

Cre10.g460700 ---------- ---------- ---------- ---------- ---------- ---------- ---------- ---------- --------CR VVRD-LGQGA

Cre12.g545750 ---------- ---------- ---------- ---------- ---------- ---------- ---------- ---------- --------CV PGRA-LGKGG

Cre12.g525950 ---------- ---------- ---------- ---------- ---------- ---------- ---------- ---------- --------CI PQRE-LGKGA

Cre12.g525750 ---------- ---------- ---------- ---------- ---------- ---------- ---------- ---------- --------CI PERE-LGKGA

Cre12.g526650 ---------- ---------- ---------- ---------- ---------- ---------- ---------- ---------- --------CI PQRE-LGKGA

Cre12.g526051 ---------- ---------- ---------- ---------- ----TATFV- ---------- ----LARRGG L-------LR ILRRKSD-CK PKDK-SCSAK

Cre12.g526250 ---------- ---------- ---------- ---------- ---------- ---------- ---------- ---------- ---------- ----------

Cre01.g054750 ---------- ---------- ---------- ---------- ---------- ---------- ---------- ---------- --------AE FKGN-LGKGA

Cre01.g055457 ---------- ---------- ---------- ---------- ---------- ---------- ---------- ---------- ------R-AE FKGD-LGKGG

Cre09.g404550 ---------- ---------- ---------- RYNAAGQQL- ---------- ---------- ---------- --------TL AQATAQRGLA VRRQKFGEGA

Cre02.g108750b ---------- ---------- ---------- ---------- ---------- ---------- ---------- ---------- ---------- ----------

Cre02.g095099 ---------- ---------- ---------- ---------- ---------- ---------- ---------- ---------- -------LT- -HVERITCTS

Cre17.g710600 ---------- ---------- ---------- ---------- ---------- ---------- ---------- ---------- ---------- ----------

CrMAPKKK14 ---------- ---------- ---------- ---------- ---------- ---------- ---------- ---------- -------LQ- -LREMLGRGG

CrMAPKKK4 ---------- ---------- ---------- ---------- ---------- ---------- ---------- ---------- -------LE- -LVEVLGKGG

CrMAPKKK8 ---------- ---------- ---------- ---------- ---------- ---------- ---------- ---------- ---------- ----------

CrMAPKKK1 ---------- ---------- ---------- ---------- ---------- ---------- ---------- ---------- ---------- ----------

CrMAPKKK12 ---------- ---------- ---------- ---------- ---------- ---------- ---------- ---------- RLLPL----- ----VLGRGA

Cre09.g390300 ---------- ---------- ---------- ---------- ---------- ---------- ---------- ---------- -------LA- -IIKFLGSGG

CrMAPKKK11 ---------- ---------- ---------- ---------- ---------- ---------- ---------- ---------- ---------- ----------

CrMAPKKK13 ---------- ---------- ---------- ---------- ---------- ---------- ---------- ---------- -------FT- -LHKLIGRGG

CrPTK7 ---------- ---------- ---------- ---------- ---------- ---------- ---------- ---------- -------VQ- -ILEPIGQGS

Cre02.g117813 ---------- ---------- ---------- ---------- ---------- ---------- ---------- ---------- IILHNL---- -----LGAGS

CrMAPKKK10 --------IQ IQDIIDVGRP GPAFGSGSGS GLAAGGSSK- ---------- ---------- ---------- --------SL MLLPASRGS- -KSKSKSREP

CrMAPKKK2 ---------- ---------- ---------- ---------- ---------- ---------- ---------- ---------- LVLTSV---- -----LGSGS

CrMAPKKK6 ---------- ---------- ---------- ---------- ---------- ---------- ---------- ---------- LRILEV---- -----IGQGG

CrMAPKKK5 ---------- ---------- ---------- ---------- ---------- ---------- ---------- ---------- IVLEGV---- -----LGHGS

CrMAPKKK9 ---------- ---------- ---------- ---------- ---------- ---------- ---------- ---------- FTLEAV---- -----LGHGS

Cre12.g516650 ---------- ---------- ---------- ---------- ---------- ---------- ---------- ---------- LHVHGL---- -----IGKGA

CrMAPKKK7 ---------- ---------- ---------- ---------- ---------- ---------- ---------- ---------- LVVHGL---- -----IGKGA

....|....| ....|....| ....|....| ....|....| ....|....| ....|....| ....|....| ....|....| ....|....| ....|....|

110 120 130 140 150 160 170 180 190 200

CrCDPK9 FGVVRAGAKR ST-------- ---------- ---------- ---------- ---------- ---------- ---------- ---------- ----------

AtCPK25 FGTTFVCVEK GT-------- ---------- ---------- ---------- ---------- ---------- ---------- ---------- ----------

AtCPK26 FGTTYMCKEI ST-------- ---------- ---------- ---------- ---------- ---------- ---------- ---------- ----------

AtCPK6 FGTTYLCTDI AT-------- ---------- ---------- ---------- ---------- ---------- ---------- ---------- ----------

AtCPK5 FGTTYLCTEI AS-------- ---------- ---------- ---------- ---------- ---------- ---------- ---------- ----------

AtCPK20 FGTTFLCVDK KT-------- ---------- ---------- ---------- ---------- ---------- ---------- ---------- ----------

AtCPK2 FGTTFLCLEK GT-------- ---------- ---------- ---------- ---------- ---------- ---------- ---------- ----------

AtCPK1 FGTTFLCVEK TT-------- ---------- ---------- ---------- ---------- ---------- ---------- ---------- ----------

AtCPK12 FGTTFLCTHK QT-------- ---------- ---------- ---------- ---------- ---------- ---------- ---------- ----------

AtCPK11 FGTTYLCTEK ST-------- ---------- ---------- ---------- ---------- ---------- ---------- ---------- ----------

AtCPK4 FGTTYLCTEK SS-------- ---------- ---------- ---------- ---------- ---------- ---------- ---------- ----------

AtCIPK7 FAKVHLARSI ES-------- ---------- ---------- ---------- ---------- ---------- ---------- ---------- ----------

AtCIPK4 FAKVHVARSI ST-------- ---------- ---------- ---------- ---------- ---------- ---------- ---------- ----------

AtCIPK22 FAKVYQAEDL QN-------- ---------- ---------- ---------- ---------- ---------- ---------- ---------- ----------

AtCIPK11 FAKVFHARDR RT-------- ---------- ---------- ---------- ---------- ---------- ---------- ---------- ----------

AtCIPK14 FAKVYHGRST AT-------- ---------- ---------- ---------- ---------- ---------- ---------- ---------- ----------

AtCIPK12 FAKVYLARNV KT-------- ---------- ---------- ---------- ---------- ---------- ---------- ---------- ----------

AtCIPK19 FAKVYLARNA QS-------- ---------- ---------- ---------- ---------- ---------- ---------- ---------- ----------

AtCIPK18 FAKVYLAQNI KS-------- ---------- ---------- ---------- ---------- ---------- ---------- ---------- ----------

AtCIPK13 FAKVYLARNI HS-------- ---------- ---------- ---------- ---------- ---------- ---------- ---------- ----------

AtCIPK16 FAKVYHGTEI ST-------- ---------- ---------- ---------- ---------- ---------- ---------- ---------- ----------

AtCIPK5 FAKVYYGKEI IG-------- ---------- ---------- ---------- ---------- ---------- ---------- ---------- ----------

AtCIPK25 FGKVYYGKEI TT-------- ---------- ---------- ---------- ---------- ---------- ---------- ---------- ----------

AtCIPK2 FAKVYFGRSN HT-------- ---------- ---------- ---------- ---------- ---------- ---------- ---------- ----------

AtCIPK10 FAKVYYGRSI LT-------- ---------- ---------- ---------- ---------- ---------- ---------- ---------- ----------

AtCIPK15 FAKVYHARHL KT-------- ---------- ---------- ---------- ---------- ---------- ---------- ---------- ----------

AtCIPK6 FAKVYHARNI QT-------- ---------- ---------- ---------- ---------- ---------- ---------- ---------- ----------

AtCIPK20 FAKVYHARNI KT-------- ---------- ---------- ---------- ---------- ---------- ---------- ---------- ----------

AtCIPK21 FAKVKLGYDT TN-------- ---------- ---------- ---------- ---------- ---------- ---------- ---------- ----------

AtCIPK17 SAKVKFAIDT LT-------- ---------- ---------- ---------- ---------- ---------- ---------- ---------- ----------

AtCIPK1 FGKVKFAKDT VS-------- ---------- ---------- ---------- ---------- ---------- ---------- ---------- ----------

AtCIPK3 FAKVKFARNS ET-------- ---------- ---------- ---------- ---------- ---------- ---------- ---------- ----------

AtCIPK9 FAKVKYAKNT VT-------- ---------- ---------- ---------- ---------- ---------- ---------- ---------- ----------

AtCIPK23 FAKVKFARNV EN-------- ---------- ---------- ---------- ---------- ---------- ---------- ---------- ----------

AtCIPK8 FAKVKFAQNT ET-------- ---------- ---------- ---------- ---------- ---------- ---------- ---------- ----------

AtCIPK24 FAKVKFARNT DT-------- ---------- ---------- ---------- ---------- ---------- ---------- ---------- ----------

CrMAPK7 YGTVWKAINR ---------- ---------- -QTNE----- ---------V VAIKKMKRK- FY-SW----- ---------- ---------- ----------

CrMAPK9 YGVVLKCRNK ---------- ---------- -ETGE----- ---------I VAVKKFKES- DEDEI----- ---------- ---------- ----------

CrMAPK13 YGDVHACLRK ---------- ---------- -DTGE----- ---------L CAVKRMKDA- HIDGT----- ---------- ---------- ----------

Cre03.g206202 FGTVALAHN- ---------- ---------- -GAGL----- ---------L VAVKAT-KT- PDDPL----- ---------- ---------- ----------

CrMAPK14 YGSVWRARDK ---------- ---------- -NTGM----- ---------M VAVKKMKDV- PTTEE----- ---------- ---------- ----------

CrMAPK17 ---------- ---------- ---------- ---------- ---------- ---------- ---------- ---------- ---------- ----------

CrMAPK16 YGAVVKCAVR DAASASGAGG GAGSSTGGPA GDEEQ----- ---------Y VAIKAFHGA- YKEKQ----- ---------- ---------- ----------

CrMAPK15 QVASWYV--- ---------- ---------- ---------- ---------G RAIKGFKEA- HKDDV----- ---------- ---------- ----------

CrMAPK12 YGTVWRCTDN ---------- ---------- -ETGQ----- ---------E VAVKALKKA- HEDAL----- ---------- ---------- ----------

CrMAPK10 -----FRQDK ---------- ---------- -ITGR----- ---------V VAVKGFKAA- HEDKD----- ---------- ---------- ----------

CrMAPK11 ---------- ---------- ---------- ---------- ---------- -------MA- HEDKD----- ---------- ---------- ----------

CrGSK3 FGVVFQATCL ---------- ---------- -ETGE----- ---------T VAIKKVLQ-- ---------- ---------- ---------- ----------

CrCDKI1 FGEVSLAGVL ---------- ---------- -ETGE----- ---------V VALKRIHIR- NTG-G----- ---------- ---------- ----------

CrMAPK5 YGVVWKAIDR ---------- ---------- -KTRE----- ---------V VALKKIFDA- FQNAT----- ---------- ---------- ----------

CrMAPK8 YGVVCSAKNL ---------- ---------- -DNQE----- ---------K VAIKKIANA- FDNVI----- ---------- ---------- ----------

CrMAPK6 FGVVCSAKDT ---------- ---------- -KTGE----- ---------K VAIKKIGNA- FENLI----- ---------- ---------- ----------

CrMAPK3 YGVVASAKDS ---------- ---------- -VTGE----- ---------K VAIKKIGNA- FENLT----- ---------- ---------- ----------

CrMAPK2 YGVVCSAVDN ---------- ---------- -FTGE----- ---------K VAIKKITNV- FEHVS----- ---------- ---------- ----------

CrMAPK4 YGLVCAAKDN ---------- ---------- -LTGE----- ---------M VAIKKIQNV- FDNVA----- ---------- ---------- ----------

CrCDPK5 YSEVWHAVHK ---------- ---------- -GTGETR--- ---------- -ALKVVA--- ---------- ---------- ---------- ----------

CrCDPK4 -MVV----EK ---------- ---------- -STG------ ---------- ---------- ---------- ---------- ---------- ----------

CrCDPK3 FGVIHVVTDA ---------- ---------- -AGNKY---- ---------- -A-------- ---------- ---------- ---------- ----------

PpCCaMK FSVVRAGVRK ---------- ---------- -QDN------ --------LQ VAIKTLKKFG YGRGDH---- ---------- ---------- ----------

NtCCaMK FSVVRRGTRR ---------- ---------- -RTLH----- ---SGQHHEV VAIKTLRRFG PPPAPE---- ---------- ---------- ----------

LjCCaMK FSVVRKGTKK ---------- ---------- -SGNE----- -------KTQ VAIKTLRRLG SSP------- ---------- ---------- ----------

MtCCaMK FSVVRKGTKK ---------- ---------- -SSIEEEKS- -------QSQ VAIKTLRRLG ASNNPS---- ---------- ---------- ----------

PsCCaMK FSVVRKGTRK ---------- ---------- -SNNDDEKSQ SQSKSQSQSQ VAIKTLRRLG TSNN------ ---------- ---------- ----------

LlCCaMK FSVVRRGISK ---------- ---------- -SRGK----- -------NND VAIKTLRRYG YTLPGA---- ---------- ---------- ----------

OsCCaMK FSIVRRGVSK ---------- ---------- -SEEK----- --------TQ VAIKTLRRLG PAMAGM---- ---------- ---------- ----------

Cre03.g199050 FGQVLLV--R ---------- ---------- -YAAQ----- ---------Y WALKAISKA- FVK------- ---------- ---------- ----------

CrCDPKK1 CGKVFLCLNT ---------- ---------- -YDLR----- ---------L YAMKAVRKV- DLESSQ---- ---------- ---------- ----------

CrCDPKK2 HGTVKLVYNT ---------- ---------- -QDDM----- ---------L YAMKVIHKR- RMRRQS---- ---------- ---------- ----------

AtMKK10 GGTVYKTRHR ---------- ---------- -RTKT----- ---------L YALKVLRPN- LN-------- ---------- ---------- ----------

AtMKK4 GGTVYKVIHR ---------- ---------- -PSSR----- ---------L YALKVIYGN- HEETVR---- ---------- ---------- ----------

AtMKK5 GGTVYKVIHT ---------- ---------- -PTSR----- ---------P FALKVIYGN- HEDTVR---- ---------- ---------- ----------

AtMKK8 GGTVFKVKDK ---------- ---------- -TTSE----- ---------I YALKKVKEN- WD-------- ---------- ---------- ----------

AtMKK7 SGIVYKVHHK ---------- ---------- -TTGE----- ---------I YALKSVNGD- MSPAFT---- ---------- ---------- ----------

AtMKK9 GGIVYKVRHK ---------- ---------- -TTSE----- ---------I YALKTVNGD- MDPIFT---- ---------- ---------- ----------

CrMAPKK1 SSIVHKGFFI ---------- ---------- -RENK----- ---------F VAVKKINVF- ERDTRH---- ---------- ---------- ----------

AtMKK3 SSVVQRAIHI ---------- ---------- -PNHR----- ---------I LALKKINIF- EREKRQ---- ---------- ---------- ----------

CrMAPKK-L SGVAKKVRNK ---------- ---------- -RDGR----- ---------H MVLKVIQFD- VSSDTV---- ---------- ---------- ----------

AtMKK6 GGVVQLVRHK ---------- ---------- -WVGK----- ---------F FAMKVIQMN- IQEEIR---- ---------- ---------- ----------

AtMEK1 SGNVQLVKHK ---------- ---------- -LTQQ----- ---------F FALKVIQLN- TEESTC---- ---------- ---------- ----------

AtMKK2 SGVVQLVQHK ---------- ---------- -WTGQ----- ---------F FALKVIQLN- IDEAIR---- ---------- ---------- ----------

AtMAPKKK13 FGAVSTAISK ---------- ---------- -TNGE----- ---------V FAVKSVDLA- TSLPTQ---- ---------- ---------- ----------

AtMAPKKK14 FGTVSKALSK ---------- ---------- -IDGG----- ---------L FAVKSIDLA- TCLPSQ---- ---------- ---------- ----------

Cre10.g464100 FGTVYQGLVH ---------- ---------- -ATGQ----- ---------E IAVKQVQLP- RDNANS---- ---------- ---------- ----------

AtANP2 ---------- ---------- ---------- ---------- ---------- ---------- ---------- ---------- ---------- ----------

AtANP1 FGTVYMGMNL ---------- ---------- -DSGE----- ---------L LAVKQVLIA- ANFASK---- ---------- ---------- ----------

AtANP3 FGRVYMGMNL ---------- ---------- -DSGE----- ---------L LAIKQVLIA- PSSASK---- ---------- ---------- ----------

Cre03.g169100 VSTVFKCQL- ---------- ---------- -VGGS----- ---------A VVVKQYHKA- KMQDK----- ---------- ---------- ----------

Cre17.g735550 AAQVYTATCR ---------- ---------- -YSGM----- ---------E VVLKVYNDVN KAPDV----- ---------- ---------- ----------

CrALK3 ASTVYKAVCR ---------- ---------- -ASGE----- ---------V VVLKIYHLM- SVCDL----- ---------- ---------- ----------

CrALK1 ASTVYLGTCR ---------- ---------- -TSGN----- ---------Q VAVKLYHKQ- KLSEL----- ---------- ---------- ----------

CrMAPKKK3 YGEVYKGSWR ---------- ---------- ---GT----- ---------E VAVKRFLEQ- NLSPP----- ---------- ---------- ----------

AtEDR1 YGEVYHADWH ---------- ---------- ---GT----- ---------E VAVKKFLDQ- DFSGA----- ---------- ---------- ----------

AtCTR1 FGTVHRAEWH ---------- ---------- ---GS----- ---------D VAVKILMEQ- DFHAE----- ---------- ---------- ----------

CrCNK9 FGTAHIVEHK ---------- ---------- -VTGE----- ---------R SVLKRVRLA- RQSAK----- ---------- ---------- ----------

AtNEK7 SSSDFVVLHD ---------- ---------- -IEDK----- ---------K YAMKKICLA- KHTDK----- ---------- ---------- ----------

AtNEK5 FGSAFLVIHK ---------- ---------- -SERR----- ---------K YVVKKIRLA- KQTER----- ---------- ---------- ----------

AtNEK6 FGAAILVHHK ---------- ---------- -AERK----- ---------K YVLKKIRLA- RQTER----- ---------- ---------- ----------

AtNEK4 FGSALLVRHK ---------- ---------- -QERK----- ---------K YVLKKIRLA- RQSDR----- ---------- ---------- ----------

AtNEK1 FGSALLVRHK ---------- ---------- -HEKK----- ---------K YVLKKIRLA- RQTQR----- ---------- ---------- ----------

AtNEK2 FGSALLVRHK ---------- ---------- -HEKK----- ---------L YVLKKIRLA- RQTGR----- ---------- ---------- ----------

AtNEK3 FGSALLVRHK ---------- ---------- -HEKK----- ---------L YVLKKIRLA- RQTGR----- ---------- ---------- ----------

CrCNK7 YSQVYLARET ---------- ---------- -HTGQ----- ---------M VAIKRVEIF- DMMDP----- ---------- ---------- ----------

CrCNK6 YAVVYKGIRR ---------- ---------- -DDGR----- ---------V VAVKKVEIF- EMSA------ ---------- ---------- ----------

CrCNK4 YGVVFKVVRK ---------- ---------- -VDKH----- ---------V YAMKEIDLQ- GMSRK----- ---------- ---------- ----------

CrFA2 FGAVFKAVRK ---------- ---------- -SDGR----- ---------V YALKQVDLR- SADFKN---- ---------- ---------- ----------

CrCNK1 YGKVYKVERE ---------- ---------- -SDKQ----- ---------L YALKEADLG- SMSQA----- ---------- ---------- ----------

CrCNK2 YGSVFLVQRL ---------- ---------- -ADSQ----- ---------T YALKEMDVR- SMSQA----- ---------- ---------- ----------

CrCNK8 YGKVYQVRSK ---------- ---------- -VDDD----- ---------I CVVKKVQFD- GTPQS----- ---------- ---------- ----------

CrCNK5 YGTAYRAKDK ---------- ---------- -YDNQ----- ---------L YCIKRIPMS- AKDD------ ---------- ---------- ----------

CrFAP403 FGCAILVTNK ---------- ---------- -LDNK----- ---------N YVIKEIDIS- RMPKA----- ---------- ---------- ----------

CrCNK3 ---------- ---------- ---------- ----M----- ---------Q VVCKQIRLF- EMDDK----- ---------- ---------- ----------

CrCDPKK3 SGQVKLAFNL RSKK------ ---------- ---------- ------L--- VAIKAVRK-- ---------- ---------- ---------- ----------

CrMAPK1 FSEVLKAQCI KNGK------ ---------- ---------- ------Y--- VAIKCMKN-- ---------- ---------- ---------- ----------

Cre08.g379600 FAVVWLAECL GTRV------ ---------- ---------- ---------- -AVKIF---- ---------- ---------- ---------- ----------

Cre02.g108601 ---------- ---------- ---------- ---------- ---------- ---------- ---------- ---------- ---------- ----------

Cre02.g108750a FAAVWLAEYA GQEV------ ---------- ---------- ---------- -AVKVN---- ---------- ---------- ---------- ----------

Cre17.g713750 YGLVHVGFLQ LPNG------ ---------- ---------- ------TTRP VAAKLFMGGL TKAKEQLN-R EV-------- ---------- ----------

Cre11.g467586 YGLVHVGLLQ RPDG------ ---------- ---------- ------TTRP VAAKLFLGGL TKAKEQLN-R EV-------- ---------- ----------

Cre11.g467588 YGLVHVGLLQ RPDG------ ---------- ---------- ------TTRP VAAKLFLGGL TKAKEQLN-R EV-------- ---------- ----------

Cre11.g467589 YGLVHVGLLQ RPDG------ ---------- ---------- ------TTRP VAAKLFLGGL TKAKEQLN-R EV-------- ---------- ----------

Cre11.g467584 YGLVHVGLLQ RPDG------ ---------- ---------- ------TTRP VAAKLFLGGL TKAKEQLN-R EV-------- ---------- ----------

Cre11.g467585 YGLVHVGLLQ RPDG------ ---------- ---------- ------TTRP VAAKLFLGGL TKAKEQLN-R EV-------- ---------- ----------

Cre12.g489850 YGSVWFGQVY SSYG------ ---------- ---------- ------IFQA VSKTAN---- -----DPN-S ---------- ---------- ----------

Cre12.g490200 NGCVYSGTVV TEYG------ ---------- ---------- ------AVRV AIKTPI---- -----QTD-S D--------- ---------- ----------

Cre08.g384250 ---------- ---------- ---------- ---------- ---------- ---------- ---------- ---------- ---------- ----------

Cre04.g217940 FGCVDCWEVT ERQS------ ---------- ---------- ------ATAS SAASSS--GL AAAA------ ---------- ---------- ----------

Cre04.g217923 FGCVDCWEVT ERQS------ ---------- ---------- ------PTAS SAASSS--GL AAAA------ ---------- ---------- ----------

Cre04.g217928 FGCVDCWEVT ERQS------ ---------- ---------- ------ATAS SAASSS--GL AAAA------ ---------- ---------- ----------

Cre08.g369700 FGRVGFCSVL YGAM------ ---------- ---------- ------HQP- ---------- ---------- ---------- ---------- ----------

Cre08.g369900 FGRVGFCDVL YGAM------ ---------- ---------- ------HQP- ---------- ---------- ---------- ---------- ----------

Cre03.g187300 SAWA------ SAGG------ ---------- ---------- ---------- ---------- ---------- ---------- ---------- ----------

Cre01.g041752 YTCLEVVEVP QPDG------ ---------- ---------- ---------- ---------- ---------- ---------- ---------- ----------

Cre12.g495401 YTCLEVVEVP QPDG------ ---------- ---------- ---------- ---------- ---------- ---------- ---------- ----------

Cre06.g266450 CGRVDVVSIP LPGG------ ---------- ---------- ---------- ---------- ---------- ---------- ---------- ----------

Cre06.g266000 FGRVQSVSIP IPGG------ ---------- ---------- ---------- ---------- ---------- ---------- ---------- ----------

Cre06.g266250 FGRVQAVNIT LPGG------ ---------- ---------- ---------- ---------- ---------- ---------- ---------- ----------

Cre10.g460700 FGYTQEVDIA LPNG------ ---------- ---------- ---------- ---------- ---------- ---------- ---------- ----------

Cre12.g545750 AGHVDLVEVT LPDG------ ---------- ---------- ---------- ---------- ---------- ---------- ---------- ----------

Cre12.g525950 FGHVEMVIIT LPDG------ ---------- ---------- ---------- ---------- ---------- ---------- ---------- ----------

Cre12.g525750 FGHVEMVIIT LPDG------ ---------- ---------- ---------- ---------- ---------- ---------- ---------- ----------

Cre12.g526650 FGHVEMVIIT LPDG------ ---------- ---------- ---------- ---------- ---------- ---------- ---------- ----------

Cre12.g526051 SGIAT--VSS SPND------ ---------- ---------- ---------- ---------- ---------- ---------- ---------- ----------

Cre12.g526250 ---------- ---------- ---------- ---------- ---------- ---------- ---------- ---------- ---------- ----------

Cre01.g054750 FGNVELVAIS LPDG------ ---------- ---------- ---------- ---------- ---------- ---------- ---------- ----------

Cre01.g055457 FGNVELVAIS LPDG------ ---------- ---------- ---------- ---------- ---------- ---------- ---------- ----------

Cre09.g404550 ERAVFQA--- -TVV------ ---------- ---------- ---------- -VVSGSS--- ---------- ---------- ---------- ----------

Cre02.g108750b ---------- ---------- ---------- ---------- ---------- ---------- ---------- ---------- ---------- ----------

Cre02.g095099 YSTVSKACHQ GSPV------ ---------- ---------- ---------- -VVKIYDVAE RDKLANAF-- ---------- ---------- ----------

Cre17.g710600 ---------- ---------- ---------- ---------- ---------- ---------- ---------- ---------- KPPATARGGG AS--------

CrMAPKKK14 GGVVLRGRCG ATDV------ ---------- ---------- ---------- -AVKLMEMPV EADPAAAT-A PVPAASLLAT AAAATAPGGE QPAAREAAPG

CrMAPKKK4 GGIVYRGRMG TQEV------ ---------- ---------- ---------- -AVKVMELPD VDAGETAASG QQPQQQR--- -SPAASPGN- ---AGGNAAA

CrMAPKKK8 ---------- ---------- ---------- ---------- ---------- ---------- ---------- ---------- ---------- ----------

CrMAPKKK1 ---------- ---------- ---------- ---------- ---------- ---------- ---------- ---------- ---------- ----------

CrMAPKKK12 FGKVYAGVYR GRHV------ ---------- ---------- ---------- -AVKLLMGLG PHQLAP---- ---------- ---------- --------PV

Cre09.g390300 YGEVYLCRWH SCDV------ ---------- ---------- ---------- -AVKCLNPSL LMPDAA---- ---------- ---------- ---------G

CrMAPKKK11 ---------- ---------- ---------- ---------- ---------- ---------- ---------- ---------- ---------- ----------

CrMAPKKK13 FGNVYLGEWE GRKV------ ---------- ---------- ---------- -AVKVVTGNN NETASD---- ---------- ---------- --------QP

CrPTK7 FARVYKGIWQ GSVV------ ---------- ---------- ---------- -ALKVLLLPA S--------- ---------- ---------- ----------

Cre02.g117813 FGRVYYGTWQ GVGV------ ---------- ---------- ---------- -AVKIIPHSE QA-------- ---------- ---------- ----------

CrMAPKKK10 GRAVYRGLWR GQQV------ ---------- ---------- ---------- -AVKTRVEAL -A-------- ---------- ---------- ----------

CrMAPKKK2 FGVVFNGTWR GLRV------ ---------- ---------- ---------- -AVKTLVVHD SL-------- ---------- ---------- ----------

CrMAPKKK6 FGVVYRGQWK GLNV------ ---------- ---------- ---------- -AVKTITFQD RV-------- ---------- ---------- ----------

CrMAPKKK5 FGTVYKGTWQ GLSV------ ---------- ---------- ---------- -AIKTVVFSA NQ-------- ---------- ---------- ----------

CrMAPKKK9 FGTVYKGTWQ GLPV------ ---------- ---------- ---------- -AIKTVVFSA TQ-------- ---------- ---------- ----------

Cre12.g516650 HGTVYRGTWR GLSV------ ---------- ---------- ---------- -AIKSMVFGP DD-------- ---------- ---------- ----------

CrMAPKKK7 HGTVYRGTWR GLEV------ ---------- ---------- ---------- -AVKSMIFGP DN-------- ---------- ---------- ----------

....|....| ....|....| ....|....| ....|....| ....|....| ....|....| ....|....| ....|....| ....|....| ....|....|

210 220 230 240 250 260 270 280 290 300

CrCDPK9 ---------- ---------- ---------- ---------- ---------- ---------- ---------- ---------- ---------- ----------

AtCPK25 ---------- ---------- ---------- ---------- ---------- ---------- ---------- ---------- ---------- ----------

AtCPK26 ---------- ---------- ---------- ---------- ---------- ---------- ---------- ---------- ---------- ----------

AtCPK6 ---------- ---------- ---------- ---------- ---------- ---------- ---------- ---------- ---------- ----------

AtCPK5 ---------- ---------- ---------- ---------- ---------- ---------- ---------- ---------- ---------- ----------

AtCPK20 ---------- ---------- ---------- ---------- ---------- ---------- ---------- ---------- ---------- ----------

AtCPK2 ---------- ---------- ---------- ---------- ---------- ---------- ---------- ---------- ---------- ----------

AtCPK1 ---------- ---------- ---------- ---------- ---------- ---------- ---------- ---------- ---------- ----------

AtCPK12 ---------- ---------- ---------- ---------- ---------- ---------- ---------- ---------- ---------- ----------

AtCPK11 ---------- ---------- ---------- ---------- ---------- ---------- ---------- ---------- ---------- ----------

AtCPK4 ---------- ---------- ---------- ---------- ---------- ---------- ---------- ---------- ---------- ----------

AtCIPK7 ---------- ---------- ---------- ---------- ---------- ---------- ---------- ---------- ---------- ----------

AtCIPK4 ---------- ---------- ---------- ---------- ---------- ---------- ---------- ---------- ---------- ----------

AtCIPK22 ---------- ---------- ---------- ---------- ---------- ---------- ---------- ---------- ---------- ----------

AtCIPK11 ---------- ---------- ---------- ---------- ---------- ---------- ---------- ---------- ---------- ----------

AtCIPK14 ---------- ---------- ---------- ---------- ---------- ---------- ---------- ---------- ---------- ----------

AtCIPK12 ---------- ---------- ---------- ---------- ---------- ---------- ---------- ---------- ---------- ----------

AtCIPK19 ---------- ---------- ---------- ---------- ---------- ---------- ---------- ---------- ---------- ----------

AtCIPK18 ---------- ---------- ---------- ---------- ---------- ---------- ---------- ---------- ---------- ----------

AtCIPK13 ---------- ---------- ---------- ---------- ---------- ---------- ---------- ---------- ---------- ----------

AtCIPK16 ---------- ---------- ---------- ---------- ---------- ---------- ---------- ---------- ---------- ----------

AtCIPK5 ---------- ---------- ---------- ---------- ---------- ---------- ---------- ---------- ---------- ----------

AtCIPK25 ---------- ---------- ---------- ---------- ---------- ---------- ---------- ---------- ---------- ----------

AtCIPK2 ---------- ---------- ---------- ---------- ---------- ---------- ---------- ---------- ---------- ----------

AtCIPK10 ---------- ---------- ---------- ---------- ---------- ---------- ---------- ---------- ---------- ----------

AtCIPK15 ---------- ---------- ---------- ---------- ---------- ---------- ---------- ---------- ---------- ----------

AtCIPK6 ---------- ---------- ---------- ---------- ---------- ---------- ---------- ---------- ---------- ----------

AtCIPK20 ---------- ---------- ---------- ---------- ---------- ---------- ---------- ---------- ---------- ----------

AtCIPK21 ---------- ---------- ---------- ---------- ---------- ---------- ---------- ---------- ---------- ----------

AtCIPK17 ---------- ---------- ---------- ---------- ---------- ---------- ---------- ---------- ---------- ----------

AtCIPK1 ---------- ---------- ---------- ---------- ---------- ---------- ---------- ---------- ---------- ----------

AtCIPK3 ---------- ---------- ---------- ---------- ---------- ---------- ---------- ---------- ---------- ----------

AtCIPK9 ---------- ---------- ---------- ---------- ---------- ---------- ---------- ---------- ---------- ----------

AtCIPK23 ---------- ---------- ---------- ---------- ---------- ---------- ---------- ---------- ---------- ----------

AtCIPK8 ---------- ---------- ---------- ---------- ---------- ---------- ---------- ---------- ---------- ----------

AtCIPK24 ---------- ---------- ---------- ---------- ---------- ---------- ---------- ---------- ---------- ----------

CrMAPK7 ---------- ---------- ---------- ---------- ---------- ---------- ---------- ---------- ---------- ----------

CrMAPK9 ---------- ---------- ---------- ---------- ---------- ---------- ---------- ---------- ---------- ----------

CrMAPK13 ---------- ---------- ---------- ---------- ---------- ---------- ---------- ---------- ---------- ----------

Cre03.g206202 ---------- ---------- ---------- ---------- ---------- ---------- ---------- ---------- ---------- ----------

CrMAPK14 ---------- ---------- ---------- ---------- ---------- ---------- ---------- ---------- ---------- ----------

CrMAPK17 ---------- ---------- ---------- ---------- ---------- ---------- ---------- ---------- ---------- ----------

CrMAPK16 ---------- ---------- ---------- ---------- ---------- ---------- ---------- ---------- ---------- ----------

CrMAPK15 ---------- ---------- ---------- ---------- ---------- ---------- ---------- ---------- ---------- ----------

CrMAPK12 ---------- ---------- ---------- ---------- ---------- ---------- ---------- ---------- ---------- ----------

CrMAPK10 ---------- ---------- ---------- ---------- ---------- ---------- ---------- ---------- ---------- ----------

CrMAPK11 ---------- ---------- ---------- ---------- ---------- ---------- ---------- ---------- ---------- ----------

CrGSK3 ---------- ---------- ---------- ---------- ---------- ---------- ---------- ---------- ---------- ----------

CrCDKI1 ---------- ---------- ---------- ---------- ---------- ---------- ---------- ---------- ---------- ----------

CrMAPK5 ---------- ---------- ---------- ---------- ---------- ---------- ---------- ---------- ---------- ----------

CrMAPK8 ---------- ---------- ---------- ---------- ---------- ---------- ---------- ---------- ---------- ----------

CrMAPK6 ---------- ---------- ---------- ---------- ---------- ---------- ---------- ---------- ---------- ----------

CrMAPK3 ---------- ---------- ---------- ---------- ---------- ---------- ---------- ---------- ---------- ----------

CrMAPK2 ---------- ---------- ---------- ---------- ---------- ---------- ---------- ---------- ---------- ----------

CrMAPK4 ---------- ---------- ---------- ---------- ---------- ---------- ---------- ---------- ---------- ----------

CrCDPK5 ---------- ---------- ---------- ---------- ---------- ---------- ---------- ---------- ---------- ----------

CrCDPK4 ---------- ---------- ---------- ---------- ---------- ---------- ---------- ---------- KE-------- ----------

CrCDPK3 ---------- ---------- ---------- ---------- ---------- ---------- ---------- ---------- ---------- ----------

PpCCaMK ---------- ---------- ---------- G--------- ---------- ---------- ---------- ------RPG- ---------- ----------

NtCCaMK ---------- ---------- ---------- K--------- ---------- ---------- ---------- ------KSL- ---------- ----------

LjCCaMK ---------- ---------- ---------- ---------- ---------- ---------- ---------- --------SG TG-------- ----------

MtCCaMK ---------- ---------- ---------- G--------- ---------- ---------- ---------- ------LPRK KD-------- ----------

PsCCaMK ---------- ---------- ---------- ---------- ---------- ---------- ---------- ------LPRK KD-------- ----------

LlCCaMK ---------- ---------- ---------- Q--------- ---------- ---------- ---------- ------RSQ- ---------- ----------

OsCCaMK ---------- ---------- ---------- K--------- ---------- ---------- ---------- ------Q--- ---------- ----------

Cre03.g199050 ---------- ---------- ---------- ---------- ---------- ---------- ---------- ---------- ---------- ----------

CrCDPKK1 ---------- ---------- ---------- ---------- ---------- ---------- ---------- --------P- ---------- ----------

CrCDPKK2 ---------- ---------- ---------- YLAEPRAVAN MMRNVG---- ---------- ---------- ---MSPLSPG SQ-------- ----------

AtMKK10 ---------- ---------- ---------- ---------- ---------- ---------- ---------- ---------- ---------- ----------

AtMKK4 ---------- ---------- ---------- ---------- ---------- ---------- ---------- ---------- ---------- ----------

AtMKK5 ---------- ---------- ---------- ---------- ---------- ---------- ---------- ---------- ---------- ----------

AtMKK8 ---------- ---------- ---------- ---------- ---------- ---------- ---------- ---------- ---------- ----------

AtMKK7 ---------- ---------- ---------- ---------- ---------- ---------- ---------- ---------- ---------- ----------

AtMKK9 ---------- ---------- ---------- ---------- ---------- ---------- ---------- ---------- ---------- ----------

CrMAPKK1 ---------- ---------- ---------- ---------- ---------- ---------- ---------- ---------- ---------- ----------

AtMKK3 ---------- ---------- ---------- ---------- ---------- ---------- ---------- ---------- ---------- ----------

CrMAPKK-L ---------- ---------- ---------- ---------- ---------- ---------- ---------- ---------- ---------- ----------

AtMKK6 ---------- ---------- ---------- ---------- ---------- ---------- ---------- ---------- ---------- ----------

AtMEK1 ---------- ---------- ---------- ---------- ---------- ---------- ---------- ---------- ---------- ----------

AtMKK2 ---------- ---------- ---------- ---------- ---------- ---------- ---------- ---------- ---------- ----------

AtMAPKKK13 ---------- ---------- ---------- ---------- ---------- ---------- ---------- ---------- ---------- ----------

AtMAPKKK14 ---------- ---------- ---------- ---------- ---------- ---------- ---------- ---------- ---------- ----------

Cre10.g464100 ---------- ---------- ---------- G--------- ---------- ---------- ---------- ---------- ---------- ----------

AtANP2 ---------- ---------- ---------- ---------- ---------- ---------- ---------- ---------- ---------- ----------

AtANP1 ---------- ---------- ---------- E--------- ---------- ---------- ---------- ---------- ---------- ----------

AtANP3 ---------- ---------- ---------- E--------- ---------- ---------- ---------- ---------- ---------- ----------

Cre03.g169100 ---------- ---------- ---------- ---------- ---------- ---------- ---------- ---------- ---------- ----------

Cre17.g735550 ---------- ---------- ---------- ---------- ---------- ---------- ---------- ---------- ---------- ----------

CrALK3 ---------- ---------- ---------- ---------- ---------- ---------- ---------- ---------- ---------- ----------

CrALK1 ---------- ---------- ---------- ---------- ---------- ---------- ---------- ---------- ---------- ----------

CrMAPKKK3 ---------- ---------- ---------- ---------- ---------- ---------- ---------- ---------- ---------- ----------

AtEDR1 ---------- ---------- ---------- ---------- ---------- ---------- ---------- ---------- ---------- ----------

AtCTR1 ---------- ---------- ---------- ---------- ---------- ---------- ---------- ---------- ---------- ----------

CrCNK9 ---------- ---------- ---------- ---------- ---------- ---------- ---------- ---------- ---------- ----------

AtNEK7 ---------- ---------- ---------- ---------- ---------- ---------- ---------- ---------- ---------- ----------

AtNEK5 ---------- ---------- ---------- ---------- ---------- ---------- ---------- ---------- ---------- ----------

AtNEK6 ---------- ---------- ---------- ---------- ---------- ---------- ---------- ---------- ---------- ----------

AtNEK4 ---------- ---------- ---------- ---------- ---------- ---------- ---------- ---------- ---------- ----------

AtNEK1 ---------- ---------- ---------- ---------- ---------- ---------- ---------- ---------- ---------- ----------

AtNEK2 ---------- ---------- ---------- ---------- ---------- ---------- ---------- ---------- ---------- ----------

AtNEK3 ---------- ---------- ---------- ---------- ---------- ---------- ---------- ---------- ---------- ----------

CrCNK7 ---------- ---------- ---------- ---------- ---------- ---------- ---------- ---------- ---------- ----------

CrCNK6 ---------- ---------- ---------- ---------- ---------- ---------- ---------- ---------- ---------- ----------

CrCNK4 ---------- ---------- ---------- ---------- ---------- ---------- ---------- ---------- ---------- ----------

CrFA2 ---------- ---------- ---------- ---------- ---------- ---------- ---------- ---------- ---------- ----------

CrCNK1 ---------- ---------- ---------- ---------- ---------- ---------- ---------- ---------- ---------- ----------

CrCNK2 ---------- ---------- ---------- ---------- ---------- ---------- ---------- ---------- ---------- ----------

CrCNK8 ---------- ---------- ---------- ---------- ---------- ---------- ---------- ---------- ---------- ----------

CrCNK5 ---------- ---------- ---------- ---------- ---------- ---------- ---------- ---------- ---------- ----------

CrFAP403 ---------- ---------- ---------- ---------- ---------- ---------- ---------- ---------- ---------- ----------

CrCNK3 ---------- ---------- ---------- ---------- ---------- ---------- ---------- ---------- ---------- ----------

CrCDPKK3 ---------- ----SHCGAG G--------H PLSHASRDGA RSYDGRLG-- ---------- ---------- -SASLGLP-G TAAGGAGAG- ----------

CrMAPK1 ---------- ----HF---- ---------- ---------- ---------- ---------- ---------- ---------- ---------- ----------

Cre08.g379600 ---------- ---------- ---------- ---------- ---------- ---------- ---------- ---------C PA-------- ----------

Cre02.g108601 ---------- ---------- ---------- ---------- ---------- ---------- ---------- ---------- ---------- ----------

Cre02.g108750a ---------- ---------- ---------- ---------- ---------- ---------- ---------- ---------L PA-------- ----------

Cre17.g713750 ---G----AA MQLQQACATA SSSSS----- --SSPEQLPL LAAAER---- ---------- ---------- -SPFVRLL-C ---------- ----------

Cre11.g467586 ---G----AA MQLQQACATA SSSSS----S SSSPEQPLPL LAAAER---- ---------- ---------- -SPFVRLL-C ---------- ----------

Cre11.g467588 ---G----AA MQLQQACATA SSSSS----S SSSPEQPLPL LAAAER---- ---------- ---------- -SPFVRLL-C ---------- ----------

Cre11.g467589 ---G----AA MQLQQACATA SSSSS----S SSSPEQPLPL LAAAER---- ---------- ---------- -SPFVRLL-C ---------- ----------

Cre11.g467584 ---G----AA MKLKQACATA SSSSSSSSSS SSSHEQPLPL LAAAEQ---- ---------- ---------- -SPFVQLL-C ---------- ----------

Cre11.g467585 ---G----AA MKLKQACATA SSSSSSSSSS SSSHEQPLPL LAAAEQ---- ---------- ---------- -SPFVQLL-C ---------- ----------

Cre12.g489850 ---------- ---------- ---------- ----TTEVTN VI-KEG---- ---------- ---------- -QVMLGLQ-A ---------- ----------

Cre12.g490200 --------SD NDSDNDCDSD S--------- --DWDSANQL LR-AEG---- ---------- ---------- -EVMFQLQ-Y ---------- ----------

Cre08.g384250 ---------- ---------- ---------- ---------- ---------- ---------- ---------- ---------- ---------- ----------

Cre04.g217940 ---------- --SCT--ATA TSS------- --S-H----T FEAAVK---- ---------- ---------- -TCALDLE-G LA-------- ----------

Cre04.g217923 ---------- -ASGT--ATA TSS------- --S-R----T FEAAVK---- ---------- ---------- -TCALDLK-G LA-------- ----------

Cre04.g217928 ---------- -ASGT--ATA TSS------- --S-R----T FEAAVK---- ---------- ---------- -TCALDLE-G LA-------- ----------

Cre08.g369700 ---------- ---------- ---------- ---------- ---------- ---------- ---------- ------AA-E AK-------- ----------

Cre08.g369900 ---------- ---------- ---------- ---------- ---------- ---------- ---------- ------AA-E AK-------- ----------

Cre03.g187300 ---------- ---------- ---------- ---------E FA-------- ---------- ---------- ---------- ---------- ----------

Cre01.g041752 ---------- ---------- ---------- ---------T CSPALL---- ---------- ---------- -MTLLR---K HK-------- ----------

Cre12.g495401 ---------- ---------- ---------- ---------T CSPALL---- ---------- ---------- -MTLLR---K HK-------- ----------

Cre06.g266450 ---------- ---------- ---------- ---------G VMQAVR---- ---------- ---------- -KEIFR---R TD-------- ----------

Cre06.g266000 ---------- ---------- ---------- ---------G VMQAVR---- ---------- ---------- -KEIFR---R TD-------- ----------

Cre06.g266250 ---------- ---------- ---------- ---------G VIKAVR---- ---------- ---------- -KEIFR---R TD-------- ----------

Cre10.g460700 ---------- ---------- ---------- ---------T TIKAAR---- ---------- ---------- -KTLLP---Q KN-------- ----------

Cre12.g545750 ---------- ---------- ---------- ---------T ILKAAR---- ---------- ---------- -KTILL---G PG-------- ----------

Cre12.g525950 ---------- ---------- ---------- ---------T TVKAAR---- ---------- ---------- -KTLHA---C ET-------- ----------

Cre12.g525750 ---------- ---------- ---------- ---------T TVKAAR---- ---------- ---------- -KTLHA---C ET-------- ----------

Cre12.g526650 ---------- ---------- ---------- ---------T TVKAAR---- ---------- ---------- -KTLHA---C ET-------- ----------

Cre12.g526051 ---------- ---------- ---------- ---------A AAVPLR---- ---------- ---------- -DDLQP---T KA-------- ----------

Cre12.g526250 ---------- ---------- ---------- ---------- ---------- ---------- ---------- ---------- ---------- ----------

Cre01.g054750 ---------- ---------- ---------- ---------T VLEAAR---- ---------- ---------- -KTVLP---C TG-------- ----------

Cre01.g055457 ---------- ---------- ---------- ---------T VLEAAR---- ---------- ---------- -KVLLQ---R TN-------- ----------

Cre09.g404550 ---------- ---------- ---------A YTVGPRLVAK QPRFLE---- ---------- ---------- ---------- ---------- ----------

Cre02.g108750b ---------- ---------- ---------- ---------- ---------- ---------- ---------- ---------- ---------- ----------

Cre02.g095099 ---------- ------AEIG VLL------H LAGWAGAVRL LDYGRHE--- ---------- ---------- DKVM------ ---------- ----------

Cre17.g710600 -------SPT ---KTAAATT TAA------A ------LRQH QSYQVHSGAA ---------- ---AVATPRE H--------G AAAHSQTGLS GGGFTSSALA

CrMAPKKK14 EEAARLRARR TMLRNAMELW CGR------A -LNHPNVVQV YATYTSVVLK VRQLPAGGGR QFYLV--PVP EDCVPPPPPG PAAAAAAGGG GGAGISS---

CrMAPKKK4 LAKEQLRARR ALLRNAMEMA VQV------R -VSHPNVLQV YATHSNVLVE QRLRPDGSAY GCLVC--TG- ---------- ---------- ----------

CrMAPKKK8 ---------- ---------- ---------- ---------- ---------- ---------- ---------- ---------- ---------- ----------

CrMAPKKK1 ---------- ---------- ---------- ---------- ---------- ---------- ---------- ---------- ---------- ----------

CrMAPKKK12 LAAGLPAAPR KATAGPQQDT VAP------A -VGTPTAVRA SAVALLQHTA D-----GTGA DAANPAHTQQ QQQQLQQQ-- PVANSNA--- ----------

Cre09.g390300 GSSA--VSSD VVTELLQEAA MLG------G -LRHPNIVWV YGIVLPGG-V R--------- ----EIKAKR DRLRA----- ---------- ----------

CrMAPKKK11 ---------- ---MATD--V QIT------P -SQSRSTLPS LPSQPSED-G L-----GNPR -LIQH----- ---------- ---------- ----------

CrMAPKKK13 EDKE--WEAR KEKMAQMEAI LMA------S -VNHPNIVHT LKVIAHQG-D A-----MDPE -LAAI----- ---------- ---------- ----------

CrPTK7 --LS--QDER LQRVAVMEAA VSS------G -LSHPNLVQT YSYSFRAL-Y D-----STAT -SASVPHRRR DDLLLRDP-- PALMESAAGG GGAAAT-G--

Cre02.g117813 ---------- -NAKVQQEVA LCI------K -FNHPNVVRS LHCVTFEA-S E-----QHQQ -LAHMQ---Q DS--LRSA-- STLGGAAAGA GGG-------

CrMAPKKK10 --GG--REAR ARLRAVLESA TST------A -LSHPHIVAT YLYDLRVL-G ---------- ---PL----- ------EL-- PTGGGGGGGG GGGSASAAAS

CrMAPKKK2 --LG--TEAR RRHRAVLEAA ISK------S -LNHPNVVAT YETEVIPL-S ---------- ---VP----- ------GT-- ARNLSALP-- ----------

CrMAPKKK6 --AG---GEK AQHRAILEAA ISS------S -LAHPNVVTT YSYDIKPL-T ---------- ---VQ----- ------GV-- PSTDSPGS-- ----------

CrMAPKKK5 -------ESR K--HALKEAA LCQ------S -ISHPNVIAT YASELQPI-G ---------- ---VL----- ------PT-- SAASSDAP-- ----------

CrMAPKKK9 -------ESR R--QALKEAA LCH------S -IIHPNIIAT YATDLQPI-G ---------- ---VL----- ------PG-- SGFDTRSH-- ----------

Cre12.g516650 -------HAR HQQRPLMEAA ISS------N -LTHPNIVTT YSYELREV-Q ---------- ---HE----- ------LA-- S--------- ----------

CrMAPKKK7 -------STR HQQRPLMEAA ISS------N -LTHANIVTT YSYELREV-Q ---------- ---HE----- ------LA-- S--------- ----------

....|....| ....|....| ....|....| ....|....| ....|....| ....|....| ....|....| ....|....| ....|....| ....|....|

310 320 330 340 350 360 370 380 390 400

CrCDPK9 ---------- ---------- ---------- ---------- ---------- ---------- ---------- ---------- ---------- ----------

AtCPK25 ---------- ---------- ---------- ---------- ---------- ---------- ---------- ---------- ---------- ----------

AtCPK26 ---------- ---------- ---------- ---------- ---------- ---------- ---------- ---------- ---------- ----------

AtCPK6 ---------- ---------- ---------- ---------- ---------- ---------- ---------- ---------- ---------- ----------

AtCPK5 ---------- ---------- ---------- ---------- ---------- ---------- ---------- ---------- ---------- ----------

AtCPK20 ---------- ---------- ---------- ---------- ---------- ---------- ---------- ---------- ---------- ----------

AtCPK2 ---------- ---------- ---------- ---------- ---------- ---------- ---------- ---------- ---------- ----------

AtCPK1 ---------- ---------- ---------- ---------- ---------- ---------- ---------- ---------- ---------- ----------

AtCPK12 ---------- ---------- ---------- ---------- ---------- ---------- ---------- ---------- ---------- ----------

AtCPK11 ---------- ---------- ---------- ---------- ---------- ---------- ---------- ---------- ---------- ----------

AtCPK4 ---------- ---------- ---------- ---------- ---------- ---------- ---------- ---------- ---------- ----------

AtCIPK7 ---------- ---------- ---------- ---------- ---------- ---------- ---------- ---------- ---------- ----------

AtCIPK4 ---------- ---------- ---------- ---------- ---------- ---------- ---------- ---------- ---------- ----------

AtCIPK22 ---------- ---------- ---------- ---------- ---------- ---------- ---------- ---------- ---------- ----------

AtCIPK11 ---------- ---------- ---------- ---------- ---------- ---------- ---------- ---------- ---------- ----------

AtCIPK14 ---------- ---------- ---------- ---------- ---------- ---------- ---------- ---------- ---------- ----------

AtCIPK12 ---------- ---------- ---------- ---------- ---------- ---------- ---------- ---------- ---------- ----------

AtCIPK19 ---------- ---------- ---------- ---------- ---------- ---------- ---------- ---------- ---------- ----------

AtCIPK18 ---------- ---------- ---------- ---------- ---------- ---------- ---------- ---------- ---------- ----------

AtCIPK13 ---------- ---------- ---------- ---------- ---------- ---------- ---------- ---------- ---------- ----------

AtCIPK16 ---------- ---------- ---------- ---------- ---------- ---------- ---------- ---------- ---------- ----------

AtCIPK5 ---------- ---------- ---------- ---------- ---------- ---------- ---------- ---------- ---------- ----------

AtCIPK25 ---------- ---------- ---------- ---------- ---------- ---------- ---------- ---------- ---------- ----------

AtCIPK2 ---------- ---------- ---------- ---------- ---------- ---------- ---------- ---------- ---------- ----------

AtCIPK10 ---------- ---------- ---------- ---------- ---------- ---------- ---------- ---------- ---------- ----------

AtCIPK15 ---------- ---------- ---------- ---------- ---------- ---------- ---------- ---------- ---------- ----------

AtCIPK6 ---------- ---------- ---------- ---------- ---------- ---------- ---------- ---------- ---------- ----------

AtCIPK20 ---------- ---------- ---------- ---------- ---------- ---------- ---------- ---------- ---------- ----------

AtCIPK21 ---------- ---------- ---------- ---------- ---------- ---------- ---------- ---------- ---------- ----------

AtCIPK17 ---------- ---------- ---------- ---------- ---------- ---------- ---------- ---------- ---------- ----------

AtCIPK1 ---------- ---------- ---------- ---------- ---------- ---------- ---------- ---------- ---------- ----------

AtCIPK3 ---------- ---------- ---------- ---------- ---------- ---------- ---------- ---------- ---------- ----------

AtCIPK9 ---------- ---------- ---------- ---------- ---------- ---------- ---------- ---------- ---------- ----------

AtCIPK23 ---------- ---------- ---------- ---------- ---------- ---------- ---------- ---------- ---------- ----------

AtCIPK8 ---------- ---------- ---------- ---------- ---------- ---------- ---------- ---------- ---------- ----------

AtCIPK24 ---------- ---------- ---------- ---------- ---------- ---------- ---------- ---------- ---------- ----------

CrMAPK7 ---------- ---------- ---------- ---------- ---------- ---------- ---------- --EECMNLRE ---------- ----------

CrMAPK9 ---------- ---------- ---------- ---------- ---------- ---------- ---------- --VRKTTLRE ---------- ----------

CrMAPK13 ---------- ---------- ---------- ---------- ---------- ---------- ---------- --SKRLALRE ---------- ----------

Cre03.g206202 ---------- ---------- ---------- ---------- ---------- ---------- ---------- --SLRLAQRE ---------- ----------

CrMAPK14 ---------- ---------- ---------- ---------- ---------- ---------- ---------- --EGEIAMRE ---------- ----------

CrMAPK17 ---------- ---------- ---------- ---------- ---------- ---------- ---------- ---MRIALRE ---------- ----------

CrMAPK16 ---------- ---------- ---------- ---------- ---------- ---------- ---------- --GLKLALRE ---------- ----------

CrMAPK15 ---------- ---------- ---------- ---------- ---------- ---------- ---------- --VMRLATRE ---------- ----------

CrMAPK12 ---------- ---------- ---------- ---------- ---------- ---------- ---------- --IMRLAVRE ---------- ----------

CrMAPK10 ---------- ---------- ---------- ---------- ---------- ---------- ---------- --IMRLAMRE ---------- ----------

CrMAPK11 ---------- ---------- ---------- ---------- ---------- ---------- ---------- --IMRLAVRE ---------- ----------

CrGSK3 ---------- ---------- ---------- ---------- ---------- ---------- ---------- --DKRFKNRE ---------- ----------

CrCDKI1 ---------- ---------- ---------- ---------- ---------- ---------- ---------- --IPDVVVRE ---------- ----------

CrMAPK5 ---------- ---------- ---------- ---------- ---------- ---------- ---------- --DAQRTFRE ---------- ----------

CrMAPK8 ---------- ---------- ---------- ---------- ---------- ---------- ---------- --DAKRTLRE ---------- ----------

CrMAPK6 ---------- ---------- ---------- ---------- ---------- ---------- ---------- --DARRTLRE ---------- ----------

CrMAPK3 ---------- ---------- ---------- ---------- ---------- ---------- ---------- --DARRTLRE ---------- ----------

CrMAPK2 ---------- ---------- ---------- ---------- ---------- ---------- ---------- --DATRIVRE ---------- ----------

CrMAPK4 ---------- ---------- ---------- ---------- ---------- ---------- ---------- --DAHRILRE ---------- ----------

CrCDPK5 ---------- ---------- ---------- ---------- -----LDDP- ---------- ------ELL- PGDL-AILRA ---------- ----------

CrCDPK4 ---------- ---------- ---------- ----YAAKSI AK--RLSVPN I--------- SA-----NKQ KQHIENIKRE ---------- ----------

CrCDPK3 ---------- ---------- ---------- ---------- ---------- C--------- KQISKRKLTG PNSIRDIRRE ---------- ----------

PpCCaMK ---------- ---------- ---------- ---------- ---------- ---------- AQMSQAEAL- --VKNEIMVM ---------- ----------

NtCCaMK ---------- ---------- ---------- ---------- -N--KSRVP- ---------- QAALISETL- --LTNELLVM ---------- ----------

LjCCaMK ---------- ---------- ---------- ----GGQKST AT--VMGFPS L--------- RQVSVSDAL- --LTNEILVM ---------- ----------

MtCCaMK ---------- ---------- ---------- ----IGEK-- -S--TIGFPT M--------- RQVSVSDTL- --LTNEILVM ---------- ----------

PsCCaMK ---------- ---------- ---------- ----GGENST ET--MMKFPT M--------- RQVSVSDAL- --LTNEILVM ---------- ----------

LlCCaMK ---------- ---------- ---------- -----PGQRG LS--PLGMPT L--------- KQVSVSDAL- --LTNEILVM ---------- ----------

OsCCaMK ---------- ---------- ---------- ------GTKP VP--GSGLPM W--------- KQVSISDAL- --LTNEILVM ---------- ----------

Cre03.g199050 ---------- ---------- ---------- ---------- ---------- ---------- E--------- QGLVEHVKRE ---------- ----------

CrCDPKK1 ---------- ---------- ---------- ---------- ----PQQGAK ---------- K--------- RNPMEDLKRE ---------- ----------

CrCDPKK2 ---------- ---------- ---------- ----TEMQRG MS--PLASPR T--------- P--------- NGMTDEYSNE ---------- ----------

AtMKK10 ---------- ---------- ---------- ---------- ---------- ---------- ---------- ----TTVTVE ---------- ----------

AtMKK4 ---------- ---------- ---------- ---------- ---------- ---------- ---------- ----RQICRE ---------- ----------

AtMKK5 ---------- ---------- ---------- ---------- ---------- ---------- ---------- ----RQICRE ---------- ----------

AtMKK8 ---------- ---------- ---------- ---------- ---------- ---------- ---------- ----STSLRE ---------- ----------

AtMKK7 ---------- ---------- ---------- ---------- ---------- ---------- ---------- ----RQLARE ---------- ----------

AtMKK9 ---------- ---------- ---------- ---------- ---------- ---------- ---------- ----RQLMRE ---------- ----------

CrMAPKK1 ---------- ---------- ---------- ---------- ---------- ---------- ---------- ----QMLNDL ---------- ----------

AtMKK3 ---------- ---------- ---------- ---------- ---------- ---------- ---------- ----QLLTEI ---------- ----------

CrMAPKK-L ---------- ---------- ---------- ---------- ---------- ---------- ---------- ----RKQARA ---------- ----------

AtMKK6 ---------- ---------- ---------- ---------- ---------- ---------- ---------- ----KQIVQE ---------- ----------

AtMEK1 ---------- ---------- ---------- ---------- ---------- ---------- ---------- ----RAISQE ---------- ----------

AtMKK2 ---------- ---------- ---------- ---------- ---------- ---------- ---------- ----KAIAQE ---------- ----------

AtMAPKKK13 ---------- ---------- ---------- ---------- ---------- ---------- ---------- ---SESLENE ---------- ----------

AtMAPKKK14 ---------- ---------- ---------- ---------- ---------- ---------- ---------- ---AESLENE ---------- ----------

Cre10.g464100 ---------- ---------- ---------- ---------- ---------- K--------- V--------- SEHIRSLESE ---------- ----------

AtANP2 ---------- ---------- ---------- ---------- ---------- ---------- ---------- -AHIQELEEE ---------- ----------

AtANP1 ---------- ---------- ---------- ---------- ---------- K--------- T--------- QAHIQELEEE ---------- ----------

AtANP3 ---------- ---------- ---------- ---------- ---------- K--------- T--------- QGHIRELEEE ---------- ----------

Cre03.g169100 ---------- ---------- ---------- ---------- ---------- ---------- ---------- --HFHKLARE ---------- ----------

Cre17.g735550 ---------- ---------- ---------- ---------- ---------- ---------- ---------- --AQHEMYRE ---------- ----------

CrALK3 ---------- ---------- ---------- ---------- ---------- ---------- ---------- --YKYQIYRE ---------- ----------

CrALK1 ---------- ---------- ---------- ---------- ---------- ---------- ---------- --NHFQVARE ---------- ----------

CrMAPKKK3 ---------- ---------- ---------- ---------- ---------- ---------- ---------- --TIRDFRDE ---------- ----------

AtEDR1 ---------- ---------- ---------- ---------- ---------- ---------- ---------- --ALAEFRSE ---------- ----------

AtCTR1 ---------- ---------- ---------- ---------- ---------- ---------- ---------- --RVNEFLRE ---------- ----------

CrCNK9 ---------- ---------- ---------- ---------- ---------- ---------- ---------- --ERQCSVRE ---------- ----------

AtNEK7 ---------- ---------- ---------- ---------- ---------- ---------- ---------- --LKQTALQE ISRA--VINY ----------

AtNEK5 ---------- ---------- ---------- ---------- ---------- ---------- ---------- --CKLAAIQE ---------- ----------

AtNEK6 ---------- ---------- ---------- ---------- ---------- ---------- ---------- --CRRSAHQE ---------- ----------

AtNEK4 ---------- ---------- ---------- ---------- ---------- ---------- ---------- --ARRSAHQE ---------- ----------

AtNEK1 ---------- ---------- ---------- ---------- ---------- ---------- ---------- --TRRSAHQE ---------- ----------

AtNEK2 ---------- ---------- ---------- ---------- ---------- ---------- ---------- --TRRSAHQE ---------- ----------

AtNEK3 ---------- ---------- ---------- ---------- ---------- ---------- ---------- --TRRSAHQE ---------- ----------

CrCNK7 ---------- ---------- ---------- ---------- ---------- ---------- ---------- -VSRQACVKE ---------- ----------

CrCNK6 ---------- ---------- ---------- ---------- ---------- ---------- ---------- -KKRDRCLQE ---------- ----------

CrCNK4 ---------- ---------- ---------- ---------- ---------- ---------- ---------- --EQEECIRE ---------- ----------

CrFA2 ---------- ---------- ---------- ---------- ---------- ---------- P--------- TLDRAAAIDE ---------- ----------

CrCNK1 ---------- ---------- ---------- ---------- ---------- ---------- ---------- --ERADAVNE ---------- ----------

CrCNK2 ---------- ---------- ---------- ---------- ---------- ---------- ---------- --EREDSINE ---------- ----------

CrCNK8 ---------- ---------- ---------- ---------- ---------- ---------- ---------- --EAEAALRE ---------- ----------

CrCNK5 ---------- ---------- ---------- ---------- ---------- ---------- ---------- ---HAGALRE ---------- ----------

CrFAP403 ---------- ---------- ---------- ---------- ---------- ---------- ---------- --ERDASEQE ---------- ----------

CrCNK3 ---------- ---------- ---------- ---------- ---------- ---------- ---------- --ARADTLTE ---------- ----------

CrCDPKK3 ---------- ---------- ---------- ---------- ---------- ---------- ---------- ---------- ---------- ----------

CrMAPK1 ---------- ---------- ---------- ---------- ---------- ---------- ---------- ---------- ---------- ----------

Cre08.g379600 ---------- ---------- ---------- ---PRYGRS- ----APFFEA MFLR------ ----EAALCA KQSHSNLVAY R--GLARLRP ----------

Cre02.g108601 ---------- ---------- ---------- ---------- ----MLEYER MFLR------ ----EAACTA SQEHRSIIQC L--GVLQLPA ----------

Cre02.g108750a ---------- ---------- ---------- ---GAGGTMA --VVPQEVTQ MFLR------ ----EAEVTG SLAHRHIIRC L--GTLELPA ----------

Cre17.g713750 ---------- ---------- ---------- ----HGGRPG ------QLPM MVM------- ---------- ---------- ------ELAK ----------

Cre11.g467586 ---------- ---------- ---------- ----HGGRPG ------QLPM MVM------- ---------- ---------- ------ELAK ----------

Cre11.g467588 ---------- ---------- ---------- ----HGGRPG ------QLPM MVM------- ---------- ---------- ------ELAK ----------

Cre11.g467589 ---------- ---------- ---------- ----HGGRPG ------QLPM MVM------- ---------- ---------- ------ELAK ----------

Cre11.g467584 ---------- ---------- ---------- ----HGGRPG ------QLPM MVM------- ---------- ---------- ------ELAK ----------

Cre11.g467585 ---------- ---------- ---------- ----HGGRPG ------QLPM MVM------- ---------- ---------- ------ELAK ----------

Cre12.g489850 ---------- ---------- ---------- ----AG---- ---------- ---------- ---------- ---------- ------LVKD ----------

Cre12.g490200 ---------- ---------- ---------- ----KG---- ---------- ---------- ---------- ---------- ------LVDD ----------

Cre08.g384250 ---------- ---------- ---------- ---------- ---------- ---------- ---------- ---------- ---------- ----------

Cre04.g217940 ---------- ---------- ---------- ---AGGETPH SVEMHAKEAA AVLAVQ---A LDSRHL---- VKVLGAYVDV LTPGEDVPPR ----------

Cre04.g217923 ---------- ---------- ---------- ---AGGATPH SVEMHAKEAA AVLAVQ---A LDSRHL---- VKVLGAYVDV LTPGEDVPPR ----------

Cre04.g217928 ---------- ---------- ---------- ---AGGETPH TVEMHAKEAA AVLAVQ---A LDSRHL---- VKVLGAYVDV LTPGEDVPPR ----------

Cre08.g369700 ---------- ---------- ---------- ---AGGRRNH RGNKKQPRPA LVTSVT---A AAFKVLPLPD PGAHDAFMAE VSAHIRATNT ----------

Cre08.g369900 ---------- ---------- ---------- ---AGGRRNH RGNKKQPRPA LVTSVT---A AAFKVLPLQD PGAHDAFMAE VSTHIRATNT ----------

Cre03.g187300 ---------- ---------- ---------- --------PP --PPPPPPPA LVL------- ---------- ---------- -------RPA ----------

Cre01.g041752 ---------- ---------- ---------- ---VTGK-PR --RKPDNRTP LLL------- ---------- ----QHLERQ A--AAHKALG ----------

Cre12.g495401 ---------- ---------- ---------- ---VTGK-PR --RKPDNRTP LLL------- ---------- ----QHLERQ A--AAHKALG ----------

Cre06.g266450 ---------- ---------- ---------- ---GV----- ------SRTE LL-------- ---------- -------QQE V--AGTRAAA ----------

Cre06.g266000 ---------- ---------- ---------- ---GV----- ------SRTE LL-------- ---------- -------RQE V--AGTLSAR ----------

Cre06.g266250 ---------- ---------- ---------- ---GL----- ------SRTE LL-------- ---------- -------QQE V--AGTRAAA ----------

Cre10.g460700 ---------- ---------- ---------- ---GH----- ------VVPA LL-------- ---------- -------QQE V--AGLRAAA ----------

Cre12.g545750 ---------- ---------- ---------- ---RN----- ---GKAIMRS IL-------- ---------- -------YQE L--AGLTAAA ----------

Cre12.g525950 ---------- ---------- ---------- ---KG----- ---AVAALRR IL-------- ---------- -------NQE L--AGLAAAA ----------

Cre12.g525750 ---------- ---------- ---------- ---KG----- ---AVAALRR IL-------- ---------- -------NQE L--AGLAAAA ----------

Cre12.g526650 ---------- ---------- ---------- ---KG----- ---AVAALRR IL-------- ---------- -------NQE L--AGLAAAA ----------

Cre12.g526051 ---------- ---------- ---------- ---AD----- ---EAADATS SS-------- ---------- -------GGE L--AGLAAAA ----------

Cre12.g526250 ---------- ---------- ---------- ---------- ---------- ---------- ---------- ---------- ---------- ----------

Cre01.g054750 ---------- ---------- ---------- ----G----- ----TAELCC LL-------- ---------- -------QQE L--DGLAAAE ----------

Cre01.g055457 ---------- ---------- ---------- ---SS----- ----MDELCD AL-------- ---------- -------QRE L--DGLAAAE ----------

Cre09.g404550 ---------- ---------- ---------- ---------- ---------- -----QLTGA MRFQQETFCR VQAEAEALAQ KF-------- ----------

Cre02.g108750b ---------- ---------- ---------- ---------- ---------- ---------- ---------- ---------- ---------- ----------

Cre02.g095099 ---------- LMLESC---- DHSLKDWCD- ---------- --------SQ RFEALTGAEY IMECLRLWCT LAELVAELHE RFHV------ ----------

Cre17.g710600 TTAVPAPALA GLLDACASGG DASSAALTT- ---AGPDAAI TTT-TIDNNN INSANTAAPS ETWLVMELCD GGSLAAAAAR GEFVRRPAVA P---------

CrMAPKKK14 ---------- -VASSAAGGP A----AANGP NGPNGPGSAP PPP-PPAAAA AGGIEPGDSV CTALLVEWCD LGSLAAAVAS RAFPRYLPPP G---------

CrMAPKKK4 ---------- ---------- ---------- -----QGSAA DPP---SAN- --LLGPAGPT HCAIVTELAD GGSLAALLTA RLFPRVVVAR G---------

CrMAPKKK8 ---------- ---------- ---------- ---------- ---------- ---------- ---------- ---------- ---------- ----------

CrMAPKKK1 ---------- ---------- ---------- ---------- ---------- ---------- ---------- ---------- ---------- ----------

CrMAPKKK12 ---------- -VTQTTTATP DAAAAAWLSP VKAGGSSSPA KAV-PQ-FGT QVQQVLPDGW DEKQQQPTDP TG-------- ---------- -LLAVTAAQD

Cre09.g390300 ---------- -----E-GDL D--AVRLTAA L--TG-GLP- ---------- ---ILPGVIR PPALVAEFLG AGSLRAALSR GADFL----- ----------

CrMAPKKK11 ER-------- Q--------- ---------- ---------- ----GWREVL ARVGAAPNKH LLMLVQELCD AGSLGAAIRQ GMFRPKPGVR -TPA------

CrMAPKKK13 ER-------- E--------- ---------- ---------- ----LFTE-- -KEDQHPPSF EWHIIMEYCD RGSLSRALAA MRFHEFVETQ GYVR------

CrPTK7 ---------- ---------- ---------- E--GGEGLE- ------GEGA GGVEAQPHGY ELQLVLEYCD LGTLRQALDR GVFHRNMQLQ ----------

Cre02.g117813 ---------- ---------- ---------- ----GVGAAG AGV-GAGAAH TAHGGNVLSM ETWIVLEYCN AGSLSSRLNM KAAVLGTTGL GLLSGGSLAL

CrMAPKKK10 RR-------- SLMHSE-SGA SASAAAATAA V--NGGGAPG ASV-SA--AA AAAAAAREVS KLYVVQELCS GGSLRAALQE GVAGCVLAGG -LFRLLALRL

CrMAPKKK2 -D-------- PL-------- ---------- A--AGGDPAG N---AS--EA VVLDATTDTY KLLLVMEYCD AGSLTRALEM GAVGSVAAG- ----------

CrMAPKKK6 ---------- ---------- ---------- ----GNGANG T---PG--MK IVDKRPVLDW KLYLVQEFCD GGSLRMAILK RKFYDAKKDE -P--------

CrMAPKKK5 ---------- ---------- ---------- ----SVSGHT P---RA--SG TGSHLNIMDW RLYIIQEFAD GGPLANLYGH RALWLSPGVV ----------

CrMAPKKK9 ---------- ---------- ---------- ----SSST-- ----TP--TS SARLYQITDW RLYIIQEFAD GGPLGSLYGH PMLWLAPGVV ----------

Cre12.g516650 ---------- ---------- ---------- ---------- --------LS PELSQQGGGW RLLIIQEFCD AGPLRSLVDC GFFLTPPRPA -HAAAP----

CrMAPKKK7 ---------- ---------- ---------- ---------- --------LS PELSQQGGGW RLLIIQEFCD AGPLRSLVDC GFFLTPPKQA -S---P----

....|....| ....|....| ....|....| ....|....| ....|....| ....|....| ....|....| ....|....| ....|....| ....|....|

410 420 430 440 450 460 470 480 490 500

CrCDPK9 ---------- ---------- ---------- ---------- ---------- ---------- ---------- ---------- ---------- ----------

AtCPK25 ---------- ---------- ---------- ---------- ---------- ---------- ---------- ---------- ---------- ----------

AtCPK26 ---------- ---------- ---------- ---------- ---------- ---------- ---------- ---------- ---------- ----------

AtCPK6 ---------- ---------- ---------- ---------- ---------- ---------- ---------- ---------- ---------- ----------

AtCPK5 ---------- ---------- ---------- ---------- ---------- ---------- ---------- ---------- ---------- ----------

AtCPK20 ---------- ---------- ---------- ---------- ---------- ---------- ---------- ---------- ---------- ----------

AtCPK2 ---------- ---------- ---------- ---------- ---------- ---------- ---------- ---------- ---------- ----------

AtCPK1 ---------- ---------- ---------- ---------- ---------- ---------- ---------- ---------- ---------- ----------

AtCPK12 ---------- ---------- ---------- ---------- ---------- ---------- ---------- ---------- ---------- ----------

AtCPK11 ---------- ---------- ---------- ---------- ---------- ---------- ---------- ---------- ---------- ----------

AtCPK4 ---------- ---------- ---------- ---------- ---------- ---------- ---------- ---------- ---------- ----------

AtCIPK7 ---------- ---------- ---------- ---------- ---------- ---------- ---------- ---------- ---------- ----------

AtCIPK4 ---------- ---------- ---------- ---------- ---------- ---------- ---------- ---------- ---------- ----------

AtCIPK22 ---------- ---------- ---------- ---------- ---------- ---------- ---------- ---------- ---------- ----------

AtCIPK11 ---------- ---------- ---------- ---------- ---------- ---------- ---------- ---------- ---------- ----------

AtCIPK14 ---------- ---------- ---------- ---------- ---------- ---------- ---------- ---------- ---------- ----------

AtCIPK12 ---------- ---------- ---------- ---------- ---------- ---------- ---------- ---------- ---------- ----------

AtCIPK19 ---------- ---------- ---------- ---------- ---------- ---------- ---------- ---------- ---------- ----------

AtCIPK18 ---------- ---------- ---------- ---------- ---------- ---------- ---------- ---------- ---------- ----------

AtCIPK13 ---------- ---------- ---------- ---------- ---------- ---------- ---------- ---------- ---------- ----------

AtCIPK16 ---------- ---------- ---------- ---------- ---------- ---------- ---------- ---------- ---------- ----------

AtCIPK5 ---------- ---------- ---------- ---------- ---------- ---------- ---------- ---------- ---------- ----------

AtCIPK25 ---------- ---------- ---------- ---------- ---------- ---------- ---------- ---------- ---------- ----------

AtCIPK2 ---------- ---------- ---------- ---------- ---------- ---------- ---------- ---------- ---------- ----------

AtCIPK10 ---------- ---------- ---------- ---------- ---------- ---------- ---------- ---------- ---------- ----------

AtCIPK15 ---------- ---------- ---------- ---------- ---------- ---------- ---------- ---------- ---------- ----------

AtCIPK6 ---------- ---------- ---------- ---------- ---------- ---------- ---------- ---------- ---------- ----------

AtCIPK20 ---------- ---------- ---------- ---------- ---------- ---------- ---------- ---------- ---------- ----------

AtCIPK21 ---------- ---------- ---------- ---------- ---------- ---------- ---------- ---------- ---------- ----------

AtCIPK17 ---------- ---------- ---------- ---------- ---------- ---------- ---------- ---------- ---------- ----------

AtCIPK1 ---------- ---------- ---------- ---------- ---------- ---------- ---------- ---------- ---------- ----------

AtCIPK3 ---------- ---------- ---------- ---------- ---------- ---------- ---------- ---------- ---------- ----------

AtCIPK9 ---------- ---------- ---------- ---------- ---------- ---------- ---------- ---------- ---------- ----------

AtCIPK23 ---------- ---------- ---------- ---------- ---------- ---------- ---------- ---------- ---------- ----------

AtCIPK8 ---------- ---------- ---------- ---------- ---------- ---------- ---------- ---------- ---------- ----------

AtCIPK24 ---------- ---------- ---------- ---------- ---------- ---------- ---------- ---------- ---------- ----------

CrMAPK7 ----VKSLR- ---------- ---------- ---------- ---------- ---------- ---------- ---------- ---------- ----------

CrMAPK9 ----VKMLR- ---------- ---------- ---------- ---------- ---------- ---------- ---------- ---------- ----------

CrMAPK13 ----AQIHR- ---------- ---------- ---------- ---------- ---------- ---------- ---------- ---------- ----------

Cre03.g206202 ----ARALR- ---------- ---------- ---------- ---------- ---------- ---------- ---------- ---------- ----------

CrMAPK14 ----VRVLQ- ---------- ---------- ---------- ---------- ---------- ---------- ---------- ---------- ----------

CrMAPK17 ----VTVLN- ---------- ---------- ---------- ---------- ---------- ---------- ---------- ---------- ----------

CrMAPK16 ----ARVLR- ---------- ---------- ---------- ---------- ---------- ---------- ---------- ---------- ----------

CrMAPK15 ----AKLLQ- ---------- ---------- ---------- ---------- ---------- ---------- ---------- ---------- ----------

CrMAPK12 ----VKVLR- ---------- ---------- ---------- ---------- ---------- ---------- ---------- ---------- ----------

CrMAPK10 ----AKMLE- ---------- ---------- ---------- ---------- ---------- ---------- ---------- ---------- ----------

CrMAPK11 ----VKVLQ- ---------- ---------- ---------- ---------- ---------- ---------- ---------- ---------- ----------

CrGSK3 ----LQIMK- ---------- ---------- ---------- ---------- ---------- ---------- ---------- ---------- ----------

CrCDKI1 ----IKALQ- ---------- ---------- ---------- ---------- ---------- ---------- ---------- ---------- ----------

CrMAPK5 ----VMFLQ- ---------- ---------- ---------- ---------- ---------- ---------- ---------- ---------- ----------

CrMAPK8 ----IKLLR- ---------- ---------- ---------- ---------- ---------- ---------- ---------- ---------- ----------

CrMAPK6 ----IKLLR- ---------- ---------- ---------- ---------- ---------- ---------- ---------- ---------- ----------

CrMAPK3 ----IKLLR- ---------- ---------- ---------- ---------- ---------- ---------- ---------- ---------- ----------

CrMAPK2 ----IKLLR- ---------- ---------- ---------- ---------- ---------- ---------- ---------- ---------- ----------

CrMAPK4 ----ITLLR- ---------- ---------- ---------- ---------- ---------- ---------- ---------- ---------- ----------

CrCDPK5 ----EAKFL- ---------- ---------- ---------- ---------- ---------- ---------- ---------- ---------- ----------

CrCDPK4 ----ALILF- ---------- ---------- ---------- ---------- ---------- ---------- ---------- ---------- ----------

CrCDPK3 ----IEIMH- ---------- ---------- ---------- ---------- ---------- ---------- ---------- ---------- ----------

PpCCaMK ----MRIVD- ---------- ---------- ---------- ---------- ---------- ---------- ---------- ---------- ----------

NtCCaMK ----IKIVE- ---------- ---------- ---------- ---------- ---------- ---------- ---------- ---------- ----------

LjCCaMK ----RRIVE- ---------- ---------- ---------- ---------- ---------- ---------- ---------- ---------- ----------

MtCCaMK ----RRIVE- ---------- ---------- ---------- ---------- ---------- ---------- ---------- ---------- ----------

PsCCaMK ----RRIVE- ---------- ---------- ---------- ---------- ---------- ---------- ---------- ---------- ----------

LlCCaMK ----RRIVE- ---------- ---------- ---------- ---------- ---------- ---------- ---------- ---------- ----------

OsCCaMK ----RRIVE- ---------- ---------- ---------- ---------- ---------- ---------- ---------- ---------- ----------

Cre03.g199050 ----KDLM-- ---------- ---------- ---------- ---------- ---------- ---------- ---------- ---------- ----------

CrCDPKK1 ----IMIM-- ---------- ---------- ---------- ---------- ---------- ---------- ---------- ---------- ----------

CrCDPKK2 ----IAVM-- ---------- ---------- ---------- ---------- ---------- ---------- ---------- ---------- ----------

AtMKK10 ----ADILK- ---------- ---------- ---------- ---------- ---------- ---------- ---------- ---------- ----------

AtMKK4 ----IEILR- ---------- ---------- ---------- ---------- ---------- ---------- ---------- ---------- ----------

AtMKK5 ----IEILR- ---------- ---------- ---------- ---------- ---------- ---------- ---------- ---------- ----------

AtMKK8 ----IEILR- ---------- ---------- ---------- ---------- ---------- ---------- ---------- ---------- ----------

AtMKK7 ----MEILR- ---------- ---------- ---------- ---------- ---------- ---------- ---------- ---------- ----------

AtMKK9 ----MEILR- ---------- ---------- ---------- ---------- ---------- ---------- ---------- ---------- ----------

CrMAPKK1 ----KALCD- ---------- ---------- ---------- ---------- ---------- ---------- ---------- ---------- ----------

AtMKK3 ----RTLCE- ---------- ---------- ---------- ---------- ---------- ---------- ---------- ---------- ----------

CrMAPKK-L ----KQYQQ- ---------- ---------- ---------- ---------- ---------- ---------- ---------- ---------- ----------

AtMKK6 ----LKINQ- ---------- ---------- ---------- ---------- ---------- ---------- ---------- ---------- ----------

AtMEK1 ----LRINL- ---------- ---------- ---------- ---------- ---------- ---------- ---------- ---------- ----------

AtMKK2 ----LKINQ- ---------- ---------- ---------- ---------- ---------- ---------- ---------- ---------- ----------

AtMAPKKK13 ----ISVFR- ---------- ---------- ---------- ---------- ---------- ---------- ---------- ---------- ----------

AtMAPKKK14 ----IVILR- ---------- ---------- ---------- ---------- ---------- ---------- ---------- ---------- ----------

Cre10.g464100 ----VAVLR- ---------- ---------- ---------- ---------- ---------- ---------- ---------- ---------- ----------

AtANP2 ----VKLLK- ---------- ---------- ---------- ---------- ---------- ---------- ---------- ---------- ----------

AtANP1 ----VKLLK- ---------- ---------- ---------- ---------- ---------- ---------- ---------- ---------- ----------

AtANP3 ----VQLLK- ---------- ---------- ---------- ---------- ---------- ---------- ---------- ---------- ----------

Cre03.g169100 ----VEAMRA L--------- ---------- ---------- ---------- ---------- ---------- ---------- ---------- ----------

Cre17.g735550 ----VAIQS- ---------- ---------- ---------- ---------- ---------- ---------- ---------- ---------- ----------

CrALK3 ----VRVHS- ---------- ---------- ---------- ---------- ---------- ---------- ---------- ---------- ----------

CrALK1 ----ITIHS- ---------- ---------- ---------- ---------- ---------- ---------- ---------- ---------- ----------

CrMAPKKK3 ----VLIMS- ---------- ---------- ---------- ---------- ---------- ---------- ---------- ---------- ----------

AtEDR1 ----VRIMR- ---------- ---------- ---------- ---------- ---------- ---------- ---------- ---------- ----------

AtCTR1 ----VAIMK- ---------- ---------- ---------- ---------- ---------- ---------- ---------- ---------- ----------

CrCNK9 ----LLLLS- ---------- ---------- ---------- ---------- ---------- ---------- ---------- ---------- ----------

AtNEK7 --DLMKLLS- ---------- ---------- ---------- ---------- ---------- ---------- ---------- ---------- ----------

AtNEK5 ----MSLIS- ---------- ---------- ---------- ---------- ---------- ---------- ---------- ---------- ----------

AtNEK6 ----MSLIA- ---------- ---------- ---------- ---------- ---------- ---------- ---------- ---------- ----------

AtNEK4 ----MELIS- ---------- ---------- ---------- ---------- ---------- ---------- ---------- ---------- ----------

AtNEK1 ----MELIS- ---------- ---------- ---------- ---------- ---------- ---------- ---------- ---------- ----------

AtNEK2 ----MELIS- ---------- ---------- ---------- ---------- ---------- ---------- ---------- ---------- ----------

AtNEK3 ----MELIS- ---------- ---------- ---------- ---------- ---------- ---------- ---------- ---------- ----------

CrCNK7 ----VKILQ- ---------- ---------- ---------- ---------- ---------- ---------- ---------- ---------- ----------

CrCNK6 ----VTLLQ- ---------- ---------- ---------- ---------- ---------- ---------- ---------- ---------- ----------

CrCNK4 ----TRVLS- ---------- ---------- ---------- ---------- ---------- ---------- ---------- ---------- ----------

CrFA2 ----ARMLA- ---------- ---------- ---------- ---------- ---------- ---------- ---------- ---------- ----------

CrCNK1 ----VRLLV- ---------- ---------- ---------- ---------- ---------- ---------- ---------- ---------- ----------

CrCNK2 ----IRLLA- ---------- ---------- ---------- ---------- ---------- ---------- ---------- ---------- ----------

CrCNK8 ----GQVLS- ---------- ---------- ---------- ---------- ---------- ---------- ---------- ---------- ----------

CrCNK5 ----AQLLD- ---------- ---------- ---------- ---------- ---------- ---------- ---------- ---------- ----------

CrFAP403 ----AKLLM- ---------- ---------- ---------- ---------- ---------- ---------- ---------- ---------- ----------

CrCNK3 ----AKVLA- ---------- ---------- ---------- ---------- ---------- ---------- ---------- ---------- ----------

CrCDPKK3 ---------- ---------- ---------- ---------- ---------- ---------- -PGAGALGNC GSVNTAPSGS VHSAGGGVAR GWRAMTPSAF

CrMAPK1 ---------- ---------- ---------- ---------- ---------- ---------- ---------- ---------- ---------- ----------

Cre08.g379600 ---------- --G------- ---------- ---------- ---------- ---------- ---------- ---------- ---------- ----------

Cre02.g108601 ---------- --AAVNSSAL ---------- ---------- ---------- ---------- ---------- ---------- ---------- ----------

Cre02.g108750a ---------- --SFHLPGLE ---------- ---------- ---------- ---------- ---------- ---------- ---------- ----------

Cre17.g713750 ---------- --GCVASELE D--------- ---------- ---------- ---------- ---------- ATGKRDEIWA QMEA--TQQQ PA------DA

Cre11.g467586 ---------- --GCVASVLE D--------- ---------- ---------- ---------- ---------- ATEKRDEIWA QMEA--TQQQ AD------AA

Cre11.g467588 ---------- --GCVASVLE D--------- ---------- ---------- ---------- ---------- ATEKRDEIWA QMEA--TQQQ AD------AA

Cre11.g467589 ---------- --GCVASELE D--------- ---------- ---------- ---------- ---------- ATEKRDEIWA QMEA--TQQQ AD------AA

Cre11.g467584 ---------- --GCVACELE D--------- ---------- ---------- ---------- ---------- ATEKRDEIWA QMEA--TQQQ AD------AA

Cre11.g467585 ---------- --GCVACELE D--------- ---------- ---------- ---------- ---------- ATEKRDEIWA QMEA--TQQQ AD------AA

Cre12.g489850 ---------- --GCTIRALA L--------- ---------- ---------- ---------- ---------- CTQ------- ---------- ----------

Cre12.g490200 ---------- --GCTVRALG L--------- ---------- ---------- ---------- ---------- TGK------- ---------- ----------

Cre08.g384250 ---------- ---------- ---------- ---------- ---------- ---------- ---------- ---------- ---------- ----------

Cre04.g217940 ---------- --GCGIANLH ---------- ---------- ---------- ---------- ---------- -------VGR IVME--VARE SL------T-

Cre04.g217923 ---------- --GCGIANLR ---------- ---------- ---------- ---------- ---------- -------VGR IVME--VARE SL------T-

Cre04.g217928 ---------- --GCGIANLR ---------- ---------- ---------- ---------- ---------- -------VGR IVME--VARE SL------T-

Cre08.g369700 ---------- --GCGHICRV ---------- ---------- ---------- ---------- ---------- -------LGY R-------TG SM------G-

Cre08.g369900 ---------- --GCDHICRV ---------- ---------- ---------- ---------- ---------- -------LGY R-------TG SM------G-

Cre03.g187300 ---------- --GCTGPAAT ---------- ---------- ---------- ---------- ---------- -------QPP VVPT--GGMA SA------A-

Cre01.g041752 ---------- --GSPHGVRL ---------- ---------- ---------- ---------- ---------- -------LWQ SRPG--RESD AH------M-

Cre12.g495401 ---------- --GSPHGVRL ---------- ---------- ---------- ---------- ---------- -------LWQ SRPG--RESD AH------V-

Cre06.g266450 ---------- --GCASAVQL ---------- ---------- ---------- ---------- ---------- -------LGY TEPQ--TDDD PH------E-

Cre06.g266000 ---------- --GCASAVQL ---------- ---------- ---------- ---------- ---------- -------LGW TEPQ--TDDD PH------E-

Cre06.g266250 ---------- --GCASAVQL ---------- ---------- ---------- ---------- ---------- -------LGY TEPQ--TDDD PH------E-

Cre10.g460700 ---------- --GCAFAIQL ---------- ---------- ---------- ---------- ---------- -------LGY SCPA--TETE PV------E-

Cre12.g545750 ---------- --GCEHAVQC ---------- ---------- ---------- ---------- ---------- -------LGY RLPT--DDDE TA------E-

Cre12.g525950 ---------- --GCEHAVQC ---------- ---------- ---------- ---------- ---------- -------LGY RLPT--DDDK RA------E-

Cre12.g525750 ---------- --GCEHAVQC ---------- ---------- ---------- ---------- ---------- -------LGY RLPT--DDDE RA------E-

Cre12.g526650 ---------- --GCEHAVQC ---------- ---------- ---------- ---------- ---------- -------LGY RLPT--DDDE RA------E-

Cre12.g526051 ---------- --GCEHAVQC ---------- ---------- ---------- ---------- ---------- -------LGY RLPT--DDDE RA------E-

Cre12.g526250 ---------- ---------- ---------- ---------- ---------- ---------- ---------- ---------- ---------- ----------

Cre01.g054750 ---------- --GCQYAVQC ---------- ---------- ---------- ---------- ---------- -------LGF RMPT--AEGE PA------E-

Cre01.g055457 ---------- --GCQYAVQC ---------- ---------- ---------- ---------- ---------- -------LGF RMPT--AEGE PA------E-

Cre09.g404550 ---------- ---------- ---------- ---------- ---------- ---------- --------NR QPAVREAAWQ QVAF--LQVV VWRVLDTEGV

Cre02.g108750b ---------- ---------- ---------- ---------- ---------- ---------- ---------- ---------- ---------- ----------

Cre02.g095099 ---------- ------AHCD LKPGNVLLSS GRLKLADFSE SMLFNGAPLL MDQARGTVVY QPPEMVHGHC VDARKADVWA MGCI------ LYEIITGELL

Cre17.g710600 ---------- ---------- ---------- ---------- ---------- ---------- ---------- ---------- ---------- ----------

CrMAPKKK14 ---------- ---------- ---------- ---------- ---------- ---------- ---------- ---------- ---------- ----------

CrMAPKKK4 ---------- ---------- ---------- ---------- ---------- ---------- ---------- ---------- ---------- ----------

CrMAPKKK8 ---------- ---------- ---------- ---------- ---------- ---------- ---------- ---------- ---------- --------VV

CrMAPKKK1 ---------- ---------- ---------- ---------- ---------- ---------- ---------- --V------- ---------- --------KL

CrMAPKKK12 EIAREQDMEQ QHGQEGYQQG QQPV------ ---------- ---------- ---------- --AAVVSEHH NHM------- ---------- --------AL

Cre09.g390300 ---------- ---------- ---------- ---------- ---------- ---------- ---------- ---------- ---------- ----------

CrMAPKKK11 ---------- ---------- ---------- ---------- ---------- ---------- ---------- ---------- ---------- ----------

CrMAPKKK13 ---------- ---------- ---------- ---------- ---------- ---------- ---------- ---------- ---------- ----------

CrPTK7 ---------- ---------- ---------- ---------- ---------- ---------- ---------- ---------- ---------- ----------

Cre02.g117813 AHD------- ---------- ---------- ---------- ---------- ---------- ---------- ---------- ---------- ----------

CrMAPKKK10 ALDAALGMRQ LHACRLVHGD LRPENVLLVA GPRAG----- ---------- ------TPGG PSAVGPHGHH TPA------- ---------- -------PLL

CrMAPKKK2 ---------- ---------- ---------- ---------- ---------- ---------- ---------- ---------- ---------- ----------

CrMAPKKK6 ---------- ---------- ---------- ---------- ---------- ---------- ---------- ---------- ---------- ----------

CrMAPKKK5 ---------- ---------- ---------- ---------- ---------- ---------- ---------- ---------- ---------- ----------

CrMAPKKK9 ---------- ---------- ---------- ---------- ---------- ---------- ---------- ---------- ---------- ----------

Cre12.g516650 -SPPTSSLRS RF-------R LPNVSSILRR DAHAN----- ---------- ------APVS PATL------ ---------- ---------- ----------

CrMAPKKK7 -PPAPAPARA KR-------K LPGMSSLLRK EASSG----- ---------- ------ASAH ADNA------ ---------- ---------- ----------

....|....| ....|....| ....|....| ....|....| ....|....| ....|....| ....|....| ....|....| ....|....| ....|....|

510 520 530 540 550 560 570 580 590 600

CrCDPK9 ---------- ---------- ---------- ---------- ---------- ---------- ---------- ---------- --G------D PVAVKSIS--

AtCPK25 ---------- ---------- ---------- ---------- ---------- ---------- ---------- ---------- --G------E EYACKSIP--

AtCPK26 ---------- ---------- ---------- ---------- ---------- ---------- ---------- ---------- --G------R EYACKSIT--

AtCPK6 ---------- ---------- ---------- ---------- ---------- ---------- ---------- ---------- --G------V DYACKSIS--

AtCPK5 ---------- ---------- ---------- ---------- ---------- ---------- ---------- ---------- --G------V DYACKSIS--

AtCPK20 ---------- ---------- ---------- ---------- ---------- ---------- ---------- ---------- --G------K EFACKTIA--

AtCPK2 ---------- ---------- ---------- ---------- ---------- ---------- ---------- ---------- --G------N EYACKSIS--

AtCPK1 ---------- ---------- ---------- ---------- ---------- ---------- ---------- ---------- --G------K EFACKSIA--

AtCPK12 ---------- ---------- ---------- ---------- ---------- ---------- ---------- ---------- --G------Q KLACKSIP--

AtCPK11 ---------- ---------- ---------- ---------- ---------- ---------- ---------- ---------- --S------A NYACKSIP--

AtCPK4 ---------- ---------- ---------- ---------- ---------- ---------- ---------- ---------- --S------A NYACKSIP--

AtCIPK7 ---------- ---------- ---------- ---------- ---------- ---------- ---------- ---------- --D------E LVAVKIIE--

AtCIPK4 ---------- ---------- ---------- ---------- ---------- ---------- ---------- ---------- --G------E LVAIKIID--

AtCIPK22 ---------- ---------- ---------- ---------- ---------- ---------- ---------- ---------- -GG------E SVAIKVVQ--

AtCIPK11 ---------- ---------- ---------- ---------- ---------- ---------- ---------- ---------- --G------Q SVAVKILN--

AtCIPK14 ---------- ---------- ---------- ---------- ---------- ---------- ---------- ---------- --G------Q SVAIKVVS--

AtCIPK12 ---------- ---------- ---------- ---------- ---------- ---------- ---------- ---------- --N------E SVAIKVID--

AtCIPK19 ---------- ---------- ---------- ---------- ---------- ---------- ---------- ---------- --G------E SVAIKVID--

AtCIPK18 ---------- ---------- ---------- ---------- ---------- ---------- ---------- ---------- --G------D KVAIKVID--

AtCIPK13 ---------- ---------- ---------- ---------- ---------- ---------- ---------- ---------- --G------E DVAIKVID--

AtCIPK16 ---------- ---------- ---------- ---------- ---------- ---------- ---------- ---------- --G------D DVAIKVIK--

AtCIPK5 ---------- ---------- ---------- ---------- ---------- ---------- ---------- ---------- --G------E CVAIKVIN--

AtCIPK25 ---------- ---------- ---------- ---------- ---------- ---------- ---------- ---------- --G------E SVAIKIIN--

AtCIPK2 ---------- ---------- ---------- ---------- ---------- ---------- ---------- ---------- --N------E SVAIKMID--

AtCIPK10 ---------- ---------- ---------- ---------- ---------- ---------- ---------- ---------- --N------Q SVAIKMID--

AtCIPK15 ---------- ---------- ---------- ---------- ---------- ---------- ---------- ---------- --G------D SVAIKVID--

AtCIPK6 ---------- ---------- ---------- ---------- ---------- ---------- ---------- ---------- --G------K SVAMKVVG--

AtCIPK20 ---------- ---------- ---------- ---------- ---------- ---------- ---------- ---------- --G------E SVAIKVID--

AtCIPK21 ---------- ---------- ---------- ---------- ---------- ---------- ---------- ---------- --G------T YVAVKIID--

AtCIPK17 ---------- ---------- ---------- ---------- ---------- ---------- ---------- ---------- --G------E SFAIKIIE--

AtCIPK1 ---------- ---------- ---------- ---------- ---------- ---------- ---------- ---------- --G------H SFAVKIID--

AtCIPK3 ---------- ---------- ---------- ---------- ---------- ---------- ---------- ---------- --G------E PVALKILD--

AtCIPK9 ---------- ---------- ---------- ---------- ---------- ---------- ---------- ---------- --G------D QAAIKILD--

AtCIPK23 ---------- ---------- ---------- ---------- ---------- ---------- ---------- ---------- --G------D NVAIKVID--

AtCIPK8 ---------- ---------- ---------- ---------- ---------- ---------- ---------- ---------- --G------E SVAMKIVD--

AtCIPK24 ---------- ---------- ---------- ---------- ---------- ---------- ---------- ---------- --G------D NVAIKIMA--

CrMAPK7 ---------- ---------- ---------- ---------- ---------- ---------- ---------- ---------K L-------NH PCIIKLKEVI

CrMAPK9 ---------- ---------- ---------- ---------- ---------- ---------- ---------- ---------A L-------RQ ENIVNLKEAF

CrMAPK13 ---------- ---------- ---------- ---------- ---------- ---------- ---------- ---------A L------PPH VNVTRLMDAF

Cre03.g206202 ---------- ---------- ---------- ---------- ---------- ---------- ---------- ---------L C-------NH PCIIKLLDCF

CrMAPK14 ---------- ---------- ---------- ---------- ---------- ---------- ---------- ---------L A-------KH VNIVNLLEAY

CrMAPK17 ---------- ---------- ---------- ---------- ---------- ---------- ---------- ---------G L-------SH CNIIKLRRAF

CrMAPK16 ---------- ---------- ---------- ---------- ---------- ---------- ---------- ---------V L-------DH PGVVKLLDAF

CrMAPK15 ---------- ---------- ---------- ---------- ---------- ---------- ---------- ---------S I-------AH PNVVTLLKAF

CrMAPK12 ---------- ---------- ---------- ---------- ---------- ---------- ---------- ---------K L-------AH PNCVKLLDAF

CrMAPK10 ---------- ---------- ---------- ---------- ---------- ---------- ---------- ---------A V-------SH ENVVRLITAF

CrMAPK11 ---------- ---------- ---------- ---------- ---------- ---------- ---------- ---------A V-------SH ENLVRLINAF

CrGSK3 ---------- ---------- ---------- ---------- ---------- ---------- ---------- ---------L V------D-H PNIVKLKHCF

CrCDKI1 ---------- ---------- ---------- ---------- ---------- ---------- ---------- ---------S V------S-H PNVVALLDVF

CrMAPK5 ---------- ---------- ---------- ---------- ---------- ---------- ---------- ---------D L------NNH DNIVRLLNVL

CrMAPK8 ---------- ---------- ---------- ---------- ---------- ---------- ---------- ---------H L------Q-H ENIVQIKDII

CrMAPK6 ---------- ---------- ---------- ---------- ---------- ---------- ---------- ---------H L------R-H ENIIQVKDVL

CrMAPK3 ---------- ---------- ---------- ---------- ---------- ---------- ---------- ---------H L------K-H DNIIAVKDIL

CrMAPK2 ---------- ---------- ---------- ---------- ---------- ---------- ---------- ---------L L------K-H PDIVDIKHIM

CrMAPK4 ---------- ---------- ---------- ---------- ---------- ---------- ---------- ---------V L------R-H PDIVEIKHIM

CrCDPK5 ---------- ---------- ---------- ---------- ---------- ---------- -------L-- ---------T L-------DC PYLIKCYETT

CrCDPK4 ---------- ---------- ---------- ---------- ---------- ---------- -------R-- ---------L R-------GT LNVVHLEDCF

CrCDPK3 ---------- ---------- ---------- ---------- ---------- ---------- -------H-- ---------L R-------GH PSVITFHGVY

PpCCaMK ---------- ---------- ---------- ---------- ---------- ---------- -------E-- ---------V S-------PH PNVIHLIDVY

NtCCaMK ---------- ---------- ---------- ---------- ---------- ---------- -------D-- ---------V S-------PH PNVIHLYDVC

LjCCaMK ---------- ---------- ---------- ---------- ---------- ---------- -------N-- ---------V S-------PH PNVIDLYDVC

MtCCaMK ---------- ---------- ---------- ---------- ---------- ---------- -------N-- ---------V S-------PH PNVIDLYDVY

PsCCaMK ---------- ---------- ---------- ---------- ---------- ---------- -------N-- ---------V S-------PH PNVIDLYDVY

LlCCaMK ---------- ---------- ---------- ---------- ---------- ---------- -------D-- ---------V S-------PH PNVIHLHDVY

OsCCaMK ---------- ---------- ---------- ---------- ---------- ---------- -------S-- ---------V A-------PH PNVINLHDVY

Cre03.g199050 ---------- ---------- ---------- ---------- ---------- ---------- -------A-- ---------E C-------DS PFLVRLEGTA

CrCDPKK1 ---------- ---------- ---------- ---------- ---------- ---------- -------K-- ---------K M-------KH TNIVTLSEVI

CrCDPKK2 ---------- ---------- ---------- ---------- ---------- ---------- -------K-- ---------E L-------DH PNVVKLYEVI

AtMKK10 ---------- ---------- ---------- ---------- ---------- ---------- ---------- ---------R I-------ES SFIIKCYAVF

AtMKK4 ---------- ---------- ---------- ---------- ---------- ---------- ---------- ---------D V-------NH PNVVKCHEMF

AtMKK5 ---------- ---------- ---------- ---------- ---------- ---------- ---------- ---------S V-------DH PNVVKCHDMF

AtMKK8 ---------- ---------- ---------- ---------- ---------- ---------- ---------- ---------M V-------NS PYVAKCHDIF

AtMKK7 ---------- ---------- ---------- ---------- ---------- ---------- ---------- ---------R T-------DS PYVVRCQGIF

AtMKK9 ---------- ---------- ---------- ---------- ---------- ---------- ---------- ---------R T-------DS PYVVKCHGIF

CrMAPKK1 ---------- ---------- ---------- ---------- ---------- ---------- -------A-- ---------P N-------NV PGLVSFYGAY

AtMKK3 ---------- ---------- ---------- ---------- ---------- ---------- -------A-- ---------P C-------H- EGLVDFHGAF

CrMAPKK-L ---------- ---------- ---------- ---------- ---------- ---------- -------H-- ---------Q H-------QQ HRLASCSKAA

AtMKK6 ---------- ---------- ---------- ---------- ---------- ---------- -------A-- ---------S S-------QC PHVVVCYHSF

AtMEK1 ---------- ---------- ---------- ---------- ---------- ---------- ---------- ---------S S-------QC PYLVSCYQSF

AtMKK2 ---------- ---------- ---------- ---------- ---------- ---------- ---------- ---------S S-------QC PNLVTSYQSF

AtMAPKKK13 ---------- ---------- ---------- ---------- ---------- ---------- -------S-- ---------L K-------PH PYIVKFLGDG

AtMAPKKK14 ---------- ---------- ---------- ---------- ---------- ---------- -------S-- ---------M K-------SH PNIVRFLGDD

Cre10.g464100 ---------- ---------- ---------- ---------- ---------- ---------- -------S-- ---------L --------RH ENIVRYLGTE

AtANP2 ---------- ---------- ---------- ---------- ---------- ---------- -------N-- ---------L --------SH PNIVRYLGTV

AtANP1 ---------- ---------- ---------- ---------- ---------- ---------- -------N-- ---------L --------SH PNIVRYLGTV

AtANP3 ---------- ---------- ---------- ---------- ---------- ---------- -------N-- ---------L --------SH PNIVRYLGTV

Cre03.g169100 ---------- ------LQQA QHQKQQQQQQ HRGSGSGAAE SSSTSSSVGG SGSGAGAVES RPQTPSSA-- ---------A C-------VC PGVVQLNDTF

Cre17.g735550 ---------- ---------- ---------- ---------- ---------- ---------- ---------- ---------S L-------QH HNIVHLFAAF

CrALK3 ---------- ---------- ---------- ---------- ---------- ---------- ---------- ---------N L-------CH ENIVHLYAAF

CrALK1 ---------- ---------- ---------- ---------- ---------- ---------- ---------- ---------S L-------DH KNIIQLWAAF

CrMAPKKK3 ---------- ---------- ---------- ---------- ---------- ---------- ---------- ---------K L-------RH PNIVLFMGAV

AtEDR1 ---------- ---------- ---------- ---------- ---------- ---------- ---------- ---------R L-------RH PNVVFFLGAV

AtCTR1 ---------- ---------- ---------- ---------- ---------- ---------- ---------- ---------R L-------RH PNIVLFMGAV

CrCNK9 ---------- ---------- ---------- ---------- ---------- ---------- ---------- ---------N L-------RH RNVLDFKGCW

AtNEK7 ---------- ---------- ---------- ---------- ---------- ---------- ---------- ---------S L-------KN PYIVHYEDSW

AtNEK5 ---------- ---------- ---------- ---------- ---------- ---------- ---------- ---------K L-------KS PYIVEYKDSW

AtNEK6 ---------- ---------- ---------- ---------- ---------- ---------- ---------- ---------R V-------QH PYIVEFKEAW

AtNEK4 ---------- ---------- ---------- ---------- ---------- ---------- ---------- ---------T V-------RN PFVVEYKDSW

AtNEK1 ---------- ---------- ---------- ---------- ---------- ---------- ---------- ---------K M-------RH PFIVEYKDSW

AtNEK2 ---------- ---------- ---------- ---------- ---------- ---------- ---------- ---------K I-------HN PFIVEYKDSW

AtNEK3 ---------- ---------- ---------- ---------- ---------- ---------- ---------- ---------K I-------RN PFIVEYKDSW

CrCNK7 ---------- ---------- ---------- ---------- ---------- ---------- ---------- ---------N V-------EH PNIVKCFRSF

CrCNK6 ---------- ---------- ---------- ---------- ---------- ---------- ---------- ---------Q L-------DH PNIIQMLDAF

CrCNK4 ---------- ---------- ---------- ---------- ---------- ---------- ---------- ---------S L-------DS DFIIRYYDSF

CrFA2 ---------- ---------- ---------- ---------- ---------- ---------- ---------- ---------Q L-------NH PHVIRHFESF

CrCNK1 ---------- ---------- ---------- ---------- ---------- ---------- ---------- ---------S I-------TH HNVIRYNEAF

CrCNK2 ---------- ---------- ---------- ---------- ---------- ---------- ---------- ---------S V-------NH PNVVCYNEAF

CrCNK8 ---------- ---------- ---------- ---------- ---------- ---------- ---------- ---------L L-------RH PHVVPYKEFF

CrCNK5 ---------- ---------- ---------- ---------- ---------- ---------- ---------- ---------S L-------DH PNIIRYRESF

CrFAP403 ---------- ---------- ---------- ---------- ---------- ---------- ---------- ---------A L-------NH PNIVRCIECF

CrCNK3 ---------- ---------- ---------- ---------- ---------- ---------- ---------- ---------Q F-------NH VNIVHYYECV

CrCDPKK3 ---------- ---------L RRASS-STHA AWGRASGCNP GNGGLASGGA VPGGVGA--- ---------- ---------G CGNS------ ----------

CrMAPK1 ---------- ---------- ---------- ---------- ---------- ---------- ---------- ---------- ---------- ----------

Cre08.g379600 ---------- ---------- ---------- ---------- ---------- ----TLP--- GLASSSWAIL SD-------F CDGGSLRD-- ----------

Cre02.g108601 ---------- ---------- ---------- ---------- ---------- -GG---K--- LDQPPMWCMV LE-------L CEPYTTKT-- ----------

Cre02.g108750a ---------- ---------- ---------- ---------- ---------- -REGLAT--- RRPEPSAAMV LE-------L CEPTSAKH-- ----------

Cre17.g713750 ITANANVASM ---------P DNVTR-ARPP P--PPP---- ---------- -GSGVLP-AS RPPPPPPGFD PLP-EASPRP RPPPPGFHAL PAA-------

Cre11.g467586 AAANTDVASM ---------S DNLTR-ARPA ---APP---- ---------- -GSGVLP-AP RPPPPPPGFE PLP-VASPRP CPPPPGFHAM PAA-------

Cre11.g467588 AAANTDVASM ---------P DNVTR-ARPA ---APP---- ---------- -GSGVLP-AP RPPPPPPGFE PLL-AASPRP CPPPPGFHAL PAA-------

Cre11.g467589 AAANTDVASM ---------S DNVTR-ARPA ---APP---- ---------- -GSGVLP-AP RPPPPPPGFE PLP-AASPRP CPPPPGFHAL PAA-------

Cre11.g467584 AAANTDVASM ---------S DNLTR-ARPA ---APP---- ---------- -GSGVLP-AP RPPPPPPGFE PLL-AASPRP CPPLPGFHAL PAA-------

Cre11.g467585 AAANTDVASM ---------P DNVTR-ARPA ---APP---- ---------- -GSGVLP-AP RPPPPPPGFE PLP-VASPRP CPPPPGFHAL PAA-------

Cre12.g489850 ---------- ---------- ---------- ---------- ---------- ---------- --PDP----- ---------- ---------- ----------

Cre12.g490200 ---------- ---------- ---------- ---------- ---------- ---------- --K------- ---------- ---------- ----------

Cre08.g384250 ---------- ---------- ---------- ---------- ---------- ---------- --------ML -DV-DLLP-- ---------- ----------

Cre04.g217940 ---------- ---------- ---------- ---------- ---------- ---------- DVVTGHR--V -LA-HCAA-- ---------- ----------

Cre04.g217923 ---------- ---------- ---------- ---------- ---------- ---------- DVVTGHR--V -LA-HCAV-- ---------- ----------

Cre04.g217928 ---------- ---------- ---------- ---------- ---------- ---------- DVVTGHR--V -LA-HCAA-- ---------- ----------

Cre08.g369700 ---------- ---------- ---------- ---------- ---------- ---------- PGVGGSWGFL -YM-QAAA-- ---------- ----------

Cre08.g369900 ---------- ---------- ---------- ---------- ---------- ---------- PGVGGSWGFL -YM-QAAA-- ---------- ----------

Cre03.g187300 ---------- ---------- ---------- ---------- ---------- ----AAT--- APQPGHP-PE NQQ-HHQH-- ---------- ----------

Cre01.g041752 ---------- ---------- ---------- ---------- ---------- ----LLL--- SYESGAVGLD SFM-DDLA-- ---------- ----------

Cre12.g495401 ---------- ---------- ---------- ---------- ---------- ----LLL--- SYESGAVGME SFM-DDLA-- ---------- ----------

Cre06.g266450 ---------- ---------- ---------- ---------- ---------- ----LLL--- SYVPGMP-LS DYL-MLVH-- ---------- ----------

Cre06.g266000 ---------- ---------- ---------- ---------- ---------- ----LLL--- SYVPGVP-LS TYL-NEVH-- ---------- ----------

Cre06.g266250 ---------- ---------- ---------- ---------- ---------- ----LLL--- SYVPGMT-LS AYL-DLVH-- ---------- ----------

Cre10.g460700 ---------- ---------- ---------- ---------- ---------- ----LLL--- TYVPGGT-LA EYL-DDLM-- ---------- ----------

Cre12.g545750 ---------- ---------- ---------- ---------- ---------- ----LLL--- SFADGGS-VE DLL-RALT-- ---------- ----------

Cre12.g525950 ---------- ---------- ---------- ---------- ---------- ----LLL--- SCAEGGS-VE NLL-NALA-- ---------- ----------

Cre12.g525750 ---------- ---------- ---------- ---------- ---------- ----LLL--- SCAEGGS-VE NLL-NALA-- ---------- ----------

Cre12.g526650 ---------- ---------- ---------- ---------- ---------- ----LLL--- SCAEGGS-VE NLL-NALA-- ---------- ----------

Cre12.g526051 ---------- ---------- ---------- ---------- ---------- ----LLL--- SCAEGGS-VE NLL-NALA-- ---------- ----------

Cre12.g526250 ---------- ---------- ---------- ---------- ---------- ---------- ---------- -MQ-NALA-- ---------- ----------

Cre01.g054750 ---------- ---------- ---------- ---------- ---------- ----LLL--- TYAKGGS-ME DFL-AKTC-- ---------- ----------

Cre01.g055457 ---------- ---------- ---------- ---------- ---------- ----LLL--- SYADGGS-VE DFL-TKMG-- ---------- ----------

Cre09.g404550 RGTGSRVVDM ---------L VEELE-GQLI KWNNNNGGVA RSMGGITEGG GGSRAAP-DP A-MVVIADDD GEEYEEEGYE HYGDDGAEVR PEHVPQAFSH

Cre02.g108750b ---------- ---------- ---------- ---------- ---------- ---------- ---------- ---------- ---------S PSSERAGDAT

Cre02.g095099 FKGNSDCMRA VAAGAAARAL RHHHH-HSRR RGLSGSRGGS GSGAGARGGS RGGSESG-AG A-GAGASGRS SGGEGGDSVS CGGA--DADV PVVPPVMPA-

Cre17.g710600 ---------- ---------- ---------- ---------- ---------- ---------- ---------- ---------- ---------- ----------

CrMAPKKK14 ---------- ---------- ---------- ---------- ---------- ---GRV---- ---------- ---------- ---------- ----------

CrMAPKKK4 ---------- ---------- ---------- ---------- ---------- ---GPV---- ---------- ---------- ---------- ----------

CrMAPKKK8 L--------- ---------- --------QR R--LGSGA-- ---------- --HGTVY-Q- ---------- --------GL WVGR--PV-A VKVVP----L

CrMAPKKK1 L--------- ---------- --------PV I--RGKGS-- ---------- --YGRVV-E- ---------- --------GL YGGQ--RV-A VKLVV----D

CrMAPKKK12 FG-------- ---K------ --------PA A--AASQA-- ---------- --PGALL-SK A-AASAPDAA AAVAASTAAA ATAA--AA-A ASAAA----P

Cre09.g390300 ---------- ---------- ---------- ---------- ---------- ---------- ---------- ---------- ---------- ----------

CrMAPKKK11 ---------- ---------- ---------- ---------- ---------- ---------- ---------- ---------- ---------- ----------

CrMAPKKK13 ---------- ---------- ---------- ---------- ---------- ---------- ---------- ---------- ---------- ----------

CrPTK7 ---------- ---------- ----R-QQQQ A--GGSGGEG GGGGGVGGGG AGGGAGG-NG G---GGVGAG GAGAGGSAAA VEGR--RAVQ PVVVPTADRH

Cre02.g117813 ---------- ---------- ------S--- ---ALS---- --------GT GGTGTGG-AN A--LISPGDS VALST----A ---------- ----------

CrMAPKKK10 LP-------- ---TPAMQRP SHFLE-SPLT A--TASGGGG GGGGG--GGG GGGGDGG-DS N--MGSPLMH AASARNFG-- --GD--RP-S PFGLRQMNSP

CrMAPKKK2 ---------- ---------- ---------- ---------- ---------- ---------- ---------- ---------- ---------- ----------

CrMAPKKK6 ---------- ---------- ---------- ---------- ---------- ---------- ---------- ---------- ---------- ----------

CrMAPKKK5 ---------- ---------- ---------- ---------- ---------- ---------- ---------- ---------- ---------- ----------

CrMAPKKK9 ---------- ---------- ---------- ---------- ---------- ---------- ---------- ---------- ---------- ----------

Cre12.g516650 ---------- ---------- ----A---TA A--PPRSRAS --------GS SSDKDVG-ES S--RGSRDTS KSARGGVGTA VEGD--ARAD RDQAGRGAAS

CrMAPKKK7 ---------- ---------- ----S-GQVA M--TPSRGSS --------GS SGKGRAV-ES G--RTSPGSD RS-SG----- ---------- GNKAGAEASP

....|....| ....|....| ....|....| ....|....| ....|....| ....|....| ....|....| ....|....| ....|....| ....|....|

610 620 630 640 650 660 670 680 690 700

CrCDPK9 ---------- ---------- ---------- ---------- ---------- --KAKLV-CK EDVKDVQAEV AIMNLVAGHP YVVTLRSTHE --DKEFVHIA

AtCPK25 ---------- ---------- ---------- ---------- ---------- --KRKLE-NE EDVEDVRREI EIMKHLLGQP NVISIKGAYE --DSVAVHMV

AtCPK26 ---------- ---------- ---------- ---------- ---------- --KRKLI-SK EDVEDVRREI QIMHHLAGYK NIVTIKGAYE --DPLYVHIV

AtCPK6 ---------- ---------- ---------- ---------- ---------- --KRKLI-SK EDVEDVRREI QIMHHLAGHK NIVTIKGAYE --DPLYVHIV

AtCPK5 ---------- ---------- ---------- ---------- ---------- --KRKLI-SK EDVEDVRREI QIMHHLAGHG SIVTIKGAYE --DSLYVHIV

AtCPK20 ---------- ---------- ---------- ---------- ---------- --KRKLT-TP EDVEDVRREI QIMHHLSGHP NVIQIVGAYE --DAVAVHVV

AtCPK2 ---------- ---------- ---------- ---------- ---------- --KRKLL-TD EDVEDVRREI QIMHHLAGHP NVISIKGAYE --DVVAVHLV

AtCPK1 ---------- ---------- ---------- ---------- ---------- --KRKLL-TD EDVEDVRREI QIMHHLAGHP NVISIKGAYE --DVVAVHLV

AtCPK12 ---------- ---------- ---------- ---------- ---------- --KRKLL-CQ EDYDDVLREI QIMHHLSEYP NVVRIESAYE --DTKNVHLV

AtCPK11 ---------- ---------- ---------- ---------- ---------- --KRKLV-CR EDYEDVWREI QIMHHLSEHP NVVRIKGTYE --DSVFVHIV

AtCPK4 ---------- ---------- ---------- ---------- ---------- --KRKLV-CR EDYEDVWREI QIMHHLSEHP NVVRIKGTYE --DSVFVHIV

AtCIPK7 ---------- ---------- ---------- ---------- ---------- --KKKTI-ES GMEPRIIREI DAMRRLRHHP NILKIHEVMA --TKSKIYLV

AtCIPK4 ---------- ---------- ---------- ---------- ---------- --KQKTI-DS GMEPRIIREI EAMRRLHNHP NVLKIHEVMA --TKSKIYLV

AtCIPK22 ---------- ---------- ---------- ---------- ---------- --KKRL--KD GLTAHVKREI SVMRRLR-HP HIVLLSEVLA --TKTKIYFV

AtCIPK11 ---------- ---------- ---------- ---------- ---------- --KKKLLTNP ALANNIKREI SIMRRLS-HP NIVKLHEVMA --TKSKIFFA

AtCIPK14 ---------- ---------- ---------- ---------- ---------- --KQRL-QKG GLNGNIQREI AIMHRLR-HP SIVRLFEVLA --TKSKIFFV

AtCIPK12 ---------- ---------- ---------- ---------- ---------- --KEKVL-KG GLIAHIKREI SILRRVR-HP NIVQLFEVMA --TKAKIYFV

AtCIPK19 ---------- ---------- ---------- ---------- ---------- --KEKVL-KS GLIAHIKREI SILRRVR-HP NIVQLFEVMA --TKSKIYFV

AtCIPK18 ---------- ---------- ---------- ---------- ---------- --KEKIM-KS GLVAHIKREI SILRRVR-HP YIVHLFEVMA --TKSKIYFV

AtCIPK13 ---------- ---------- ---------- ---------- ---------- --KEKIV-KS GLAGHIKREI SILRRVR-HP YIVHLLEVMA --TKTKIYIV

AtCIPK16 ---------- ---------- ---------- ---------- ---------- --KDHVFKRR GMMEQIEREI AVMRLLR-HP NVVELREVMA --TKKKIFFV

AtCIPK5 ---------- ---------- ---------- ---------- ---------- --KDQVMKRP GMMEQIKREI SIMKLVR-HP NIVELKEVMA --TKTKIFFV

AtCIPK25 ---------- ---------- ---------- ---------- ---------- --KDQV-KRE GMMEQIKREI SIMRLVR-HP NIVELKEVMA --TKTKIFFI

AtCIPK2 ---------- ---------- ---------- ---------- ---------- --KDKVM-RV GLSQQIKREI SVMRIAK-HP NVVELYEVMA --TKSRIYFV

AtCIPK10 ---------- ---------- ---------- ---------- ---------- --KEKVM-KV GLIEQIKREI SVMRIAR-HP NVVELYEVMA --TKTRIYFV

AtCIPK15 ---------- ---------- ---------- ---------- ---------- --KERIL-KV GMTEQIKREI SAMRLLR-HP NIVELHEVMA --TKSKIYFV

AtCIPK6 ---------- ---------- ---------- ---------- ---------- --KEKVV-KV GMVDQIKREI SVMRMVK-HP NIVELHEVMA --SKSKIYFA

AtCIPK20 ---------- ---------- ---------- ---------- ---------- --KQKVA-KV GLIDQIKREI SVMRLVR-HP HVVFLHEVMA --SKTKIYFA

AtCIPK21 ---------- ---------- ---------- ---------- ---------- --KALVI-QK GLESQVKREI RTMKLLN-HP NIVQIHEVIG --TKTKICIV

AtCIPK17 ---------- ---------- ---------- ---------- ---------- --KSCIT-RL NVSFQIKREI RTLKVLK-HP NIVRLHEVLA --SKTKIYMV

AtCIPK1 ---------- ---------- ---------- ---------- ---------- --KSRIA-DL NFSLQIKREI RTLKMLK-HP HIVRLHEVLA --SKTKINMV

AtCIPK3 ---------- ---------- ---------- ---------- ---------- --KEKVL-KH KMAEQIRREI ATMKLIK-HP NVVQLYEVMA --SKTKIFII

AtCIPK9 ---------- ---------- ---------- ---------- ---------- --REKVF-RH KMVEQLKREI STMKLIK-HP NVVEIIEVMA --SKTKIYIV

AtCIPK23 ---------- ---------- ---------- ---------- ---------- --KEKVL-KN KMIAQIKREI STMKLIK-HP NVIRMFEVMA --SKTKIYFV

AtCIPK8 ---------- ---------- ---------- ---------- ---------- --RSTII-KR KMVDQIKREI SIMKLVR-HP CVVRLYEVLA --SRTKIYII

AtCIPK24 ---------- ---------- ---------- ---------- ---------- --KSTIL-KN RMVDQIKREI SIMKIVR-HP NIVRLYEVLA --SPSKIYIV

CrMAPK7 REN------- ---------- ---------- ---------- ---------- ---------- ---------- ---------- ---------- ----DELFFV

CrMAPK9 RRK------- ---------- ---------- ---------- ---------- ---------- ---------- ---------- ---------- ----QKLYLV

CrMAPK13 RGTS------ ---------- ---------- ---------- ---------- ---------- ---------- ---------- ---------- ----GRLYLV

Cre03.g206202 RSPS------ ---------- ---------- ---------- ---------- ---------- ---------- ---------- ---------- ----GRAYLV

CrMAPK14 KSQS------ ---------- ---------- ---------- ---------- ---------- ---------- ---------- ---------- ----GRLYLV

CrMAPK17 RTPS------ ---------- ---------- ---------- ---------- ---------- ---------- ---------- ---------- ----GRVCMV

CrMAPK16 KSPS------ ---------- ---------- ---------- ---------- ---------- ---------- ---------- ---------- ----GRPYLV

CrMAPK15 RSQH------ ---------- ---------- ---------- ---------- ---------- ---------- ---------- ---------- ----KHIYMV

CrMAPK12 KSKT------ ---------- ---------- ---------- ---------- ---------- ---------- ---------- ---------- ----GRVYMV

CrMAPK10 KSKS------ ---------- ---------- ---------- ---------- ---------- ---------- ---------- ---------- ----GRVYMV

CrMAPK11 KSKS------ ---------- ---------- ---------- ---------- ---------- ---------- ---------- ---------- ----GRVYMV

CrGSK3 YSHT------ ------D--- ---------- ---------- ---------- ---------- ---------- ---------- ---------- -KDETYLHLV

CrCDKI1 PK-------- ---------- ---------- ---------- ---------- ---------- ---------- ---------- ---------- ---GQAIYLV

CrMAPK5 KA-------- ---------- ---------- ---------- ---------- ---------- ---------- ---------- ---------- -ENDRDLYLV

CrMAPK8 PPTN------ ------R--- ---------- ---------- ---------- ---------- ---------- ---------- ---------- -DAFKDLYVV

CrMAPK6 KPPS------ ------R--- ---------- ---------- ---------- ---------- ---------- ---------- ---------- -DNFNDVYII

CrMAPK3 KPPA------ ------K--- ---------- ---------- ---------- ---------- ---------- ---------- ---------- -DKFNDVYLV

CrMAPK2 LPPS------ ------P--- ---------- ---------- ---------- ---------- ---------- ---------- ---------- -KDFKDIYVV

CrMAPK4 LPSD------ ------P--- ---------- ---------- ---------- ---------- ---------- ---------- ---------- -NTFKDLYVC

CrCDPK5 HD-------- ---------- ---------- ---------- ---------- ---------- ---------- ---------- ---------- ---GVHLFLV

CrCDPK4 EC-------- ---------- ---------- ---------- ---------- ---------- ---------- ---------- ---------- ---EDSVHLI

CrCDPK3 ED-------- ---------- ---------- ---------- ---------- ---------- ---------- ---------- ---------- ---VSDVYMV

PpCCaMK ED-------- ---------- ---------- ---------- ---------- ---------- ---------- ---------- ---------- ---DGAVHLV

NtCCaMK ED-------- ---------- ---------- ---------- ---------- ---------- ---------- ---------- ---------- ---PSGVHLI

LjCCaMK ED-------- ---------- ---------- ---------- ---------- ---------- ---------- ---------- ---------- ---SNGVHLV

MtCCaMK ED-------- ---------- ---------- ---------- ---------- ---------- ---------- ---------- ---------- ---TNGVHLV

PsCCaMK ED-------- ---------- ---------- ---------- ---------- ---------- ---------- ---------- ---------- ---TNGVHLV

LlCCaMK ED-------- ---------- ---------- ---------- ---------- ---------- ---------- ---------- ---------- ---ANGVHLV

OsCCaMK ED-------- ---------- ---------- ---------- ---------- ---------- ---------- ---------- ---------- ---VHGVHLV

Cre03.g199050 AD-------- ---------- ---------- ---------- ---------- ---------- ---------- ---------- ---------- ---DTTLYMM

CrCDPKK1 DDP------- ---------- ---------- ---------- ---------- ---------- ---------- ---------- ---------- --AGSKLLLV

CrCDPKK2 HDP------- ---------- ---------- ---------- ---------- ---------- ---------- ---------- ---------- --SNNKLLMT

AtMKK10 VS-------- ---------- ---------- ---------- ---------- ---------- ---------- ---------- ---------- ---LYDLCFV

AtMKK4 DQ-------- ---------- ---------- ---------- ---------- ---------- ---------- ---------- ---------- ---NGEIQVL

AtMKK5 DH-------- ---------- ---------- ---------- ---------- ---------- ---------- ---------- ---------- ---NGEIQVL

AtMKK8 QNP------- ---------- ---------- ---------- ---------- ---------- ---------- ---------- ---------- ---SGEVSIL

AtMKK7 EKP------- ---------- ---------- ---------- ---------- ---------- ---------- ---------- ---------- --IVGEVSIL

AtMKK9 EKP------- ---------- ---------- ---------- ---------- ---------- ---------- ---------- ---------- --VVGEVSIL

CrMAPKK1 HVP------- ---------- ---------- ---------- ---------- ---------- ---------- ---------- ---------- --ESGQISIV

AtMKK3 YSP------- ---------- ---------- ---------- ---------- ---------- ---------- ---------- ---------- --DSGQISIA

CrMAPKK-L PSSS------ ------AAAA GPVAAPGSAA AAAAGTSNGG SSGAA----- ---------- ---------- ---------- -------AAA AAAAGPSSHV

AtMKK6 YHN------- ---------- ---------- --------GA F--------- ---------- ---------- ---------- ---------- -------SLV

AtMEK1 YHN------- ---------- ---------- --------GL V--------- ---------- ---------- ---------- ---------- -------SII

AtMKK2 YDN------- ---------- ---------- --------GA I--------- ---------- ---------- ---------- ---------- -------SLI

AtMAPKKK13 VSKE------ ---------- ---------- ---------- ---------- ---------- ---------- ---------- ---------- -GTTTFRNLY

AtMAPKKK14 VSKE------ ---------- ---------- ---------- ---------- ---------- ---------- ---------- ---------- -GTASFRNLH

Cre10.g464100 RTS------- ---------- ---------- ---------- ---------- ---------- ---------- ---------- ---------- ----EHLNIF

AtANP2 RED------- ---------- ---------- ---------- ---------- ---------- ---------- ---------- ---------- ----ETLNIL

AtANP1 RED------- ---------- ---------- ---------- ---------- ---------- ---------- ---------- ---------- ----DTLNIL

AtANP3 RES------- ---------- ---------- ---------- ---------- ---------- ---------- ---------- ---------- ----DSLNIL

Cre03.g169100 ED-------- ---------- ---------- ---------- ---------- ---------- ---------- ---------- ---------- ---SASIYLV

Cre17.g735550 QE-------- ---------- ---------- ---------- ---------- ---------- ---------- ---------- ---------- ---GQTLVLV

CrALK3 QE-------- ---------- ---------- ---------- ---------- ---------- ---------- ---------- ---------- ---GDKVILV

CrALK1 ED-------- ---------- ---------- ---------- ---------- ---------- ---------- ---------- ---------- ---QYGIYLV

CrMAPKKK3 TQ-------- ---------- ---------- ---------- ---------- ---------- ---------- ---------- ---------- ---SNQLAIV

AtEDR1 TR-------- ---------- ---------- ---------- ---------- ---------- ---------- ---------- ---------- ---PPNLSIV

AtCTR1 TQ-------- ---------- ---------- ---------- ---------- ---------- ---------- ---------- ---------- ---PPNLSIV

CrCNK9 VEG------- ---------- ---------- ---------- ---------- ---------- ---------- ---------- ---------- ---GCNLCLL

AtNEK7 IDN------- ---------- ---------- ---------- ---------- ---------- ---------- ---------- ---------- ---DNNACIF

AtNEK5 VEK------- ---------- ---------- ---------- ---------- ---------- ---------- ---------- ---------- ---DC-VCIV

AtNEK6 VEK------- ---------- ---------- ---------- ---------- ---------- ---------- ---------- ---------- ---GCYVCIV

AtNEK4 VEK------- ---------- ---------- ---------- ---------- ---------- ---------- ---------- ---------- ---GCYVCIV

AtNEK1 VEK------- ---------- ---------- ---------- ---------- ---------- ---------- ---------- ---------- ---ACYVCIV

AtNEK2 VEK------- ---------- ---------- ---------- ---------- ---------- ---------- ---------- ---------- ---GCYVCII

AtNEK3 VEK------- ---------- ---------- ---------- ---------- ---------- ---------- ---------- ---------- ---GCYVCIV

CrCNK7 LSE------- ---------- ---------- ---------- ---------- ---------- ---------- ---------- ---------- --ADNELVIV

CrCNK6 IDE------- ---------- ---------- ---------- ---------- ---------- ---------- ---------- ---------- ----NMLIII

CrCNK4 LE-------- ---------- ---------- ---------- ---------- ---------- ---------- ---------- ---------- ---KGKLYII

CrFA2 VDG------- ---------- ---------- ---------- ---------- ---------- ---------- ---------- ---------- ---EGKLNIL

CrCNK1 LL-------- ---------- ---------- ---------- ---------- ---------- ---------- ---------- ---------- ---GNKLCTV

CrCNK2 LD-------- ---------- ---------- ---------- ---------- ---------- ---------- ---------- ---------- ---GNRLCII

CrCNK8 KHT------- ---------- ---------- ---------- ---------- ---------- ---------- ---------- ---------- ---DGDLCLV

CrCNK5 VDK------- ---------- ---------- ---------- ---------- ---------- ---------- ---------- ---------- ---DGSLCIV

CrFAP403 TH-------- ---------- ---------- ---------- ---------- ---------- ---------- ---------- ---------- ---MNKLCIV

CrCNK3 LE-------- ---------- ---------- ---------- ---------- ---------- ---------- ---------- ---------- ---SGVLNIV

CrCDPKK3 ---------- ---------- GPGGAPGG-- -----PSESA ---------- ---------- ---EDFVREI AILKRL-SHP NVVQLLEVID DPASDCLLLV

CrMAPK1 ---------- ---------- ---------- -----DSLDQ ---------- ---------- ---VNNLREI QALRRLSPHA NIIKLLEVLY DQPTGRLALV

Cre08.g379600 ---------- ---------- ---------- ---------- ---------- ---------- ---------- ---------- ----LLVGRV PATLLP----

Cre02.g108601 ---------- ---------- ---------- ---------- ---------- ---------- ---------- ---------- ----LITQG- ----------

Cre02.g108750a ---------- ---------- ---------- ---------- ---------- ---------- ---------- ---------- ----LIVRA- ----------

Cre17.g713750 ---------- ---------- ---PRAPL-- -----PPPGF AP-L-A---- ---------- SPPL------ -------PAS APAAAA-VTA EDPLLP--SV

Cre11.g467586 ---------- ---------- ---PRPPL-- -----PPPGF AP-LLA---- ---------- SPPV------ -------PAS APASAATSAA EDPLLP--SV

Cre11.g467588 ---------- ---------- ---PRPPL-- -----PPPGF AP-LLA---- ---------- SPPV------ -------PAS APASAATSAA EDPLLP--SV

Cre11.g467589 ---------- ---------- ---PRPPL-- -----PPPGF AP-LLA---- ---------- SPPV------ -------PAS APASAATSAA EDPLLP--SV

Cre11.g467584 ---------- ---------- ---PRPPL-- -----PPPGF AP-LLA---- ---------- SPPV------ -------PAS APASAATSAA EDPLLP--SV

Cre11.g467585 ---------- ---------- ---PRPPL-- -----PPPGF AP-LLA---- ---------- SPPV------ -------PAS APASAATSAA EDPLLP--SV

Cre12.g489850 ---------- ---------- ---------- ------T--- ---------- ---------- ---------- --------TG HVVTLLMERA QRSLMA--DV

Cre12.g490200 ---------- ---------- ---------- ---------- ---------- ---------- ---------- --------KS HPVQLLMELA QCSLWD--KL

Cre08.g384250 ---------- ---------- -----GGL-- -----ALIME ---------- ---------- ---------- ---------- ---------- ----------

Cre04.g217940 ---------- ---------- -----GGA-- -----A---- ---------- ---------- ---------- ---------- ---------- ----------

Cre04.g217923 ---------- ---------- -----GGA-- -----A---- ---------- ---------- ---------- ---------- ---------- ----------

Cre04.g217928 ---------- ---------- -----GGA-- -----A---- ---------- ---------- ---------- ---------- ---------- ----------

Cre08.g369700 ---------- ---------- -----GGA-- -----AGTLW ---------- ---------- ---------- ---------- ---------- ----------

Cre08.g369900 ---------- ---------- -----GGA-- -----AGTLW ---------- ---------- ---------- ---------- ---------- ----------

Cre03.g187300 ---------- ---------- -----G-Q-- -----PQQQA AP-AV----- ---------- ---------- ---------- ---------- ----------

Cre01.g041752 ---------- ---------- -----MDE-- -----TQREF DA-ML----- ---------- ---------- ---------- ---------- ----------

Cre12.g495401 ---------- ---------- -----MDE-- -----INRDF DA-ML----- ---------- ---------- ---------- ---------- ----------

Cre06.g266450 ---------- ---------- -----CVD-- -----VDRRS GV-------- ---------- ---------- ---------- ---------- ----------

Cre06.g266000 ---------- ---------- -----RAD-- -----VVKRC VA-------- ---------- ---------- ---------- ---------- ----------

Cre06.g266250 ---------- ---------- -----RAD-- -----VVKRF VA-------- ---------- ---------- ---------- ---------- ----------

Cre10.g460700 ---------- ---------- -----RAN-- -----QMRIF HG-P------ ---------- ---------- ---------- ---------- ----------

Cre12.g545750 ---------- ---------- -----WAH-- -----RARIA NA-------- ---------- ---------- ---------- ---------- ----------

Cre12.g525950 ---------- ---------- -----KEH-- -----FDRID NA-------- ---------- ---------- ---------- ---------- ----------

Cre12.g525750 ---------- ---------- -----KEH-- -----FDRID NA-------- ---------- ---------- ---------- ---------- ----------

Cre12.g526650 ---------- ---------- -----KEH-- -----FDRID NA-------- ---------- ---------- ---------- ---------- ----------

Cre12.g526051 ---------- ---------- -----KEH-- -----FDRID NA-------- ---------- ---------- ---------- ---------- ----------

Cre12.g526250 ---------- ---------- -----KEH-- -----FDRID NA-------- ---------- ---------- ---------- ---------- ----------

Cre01.g054750 ---------- ---------- -----QEL-- -----FKRLV A--------- ---------- ---------- ---------- ---------- ----------

Cre01.g055457 ---------- ---------- -----SGL-- -----YQDIY E--------- ---------- ---------- ---------- ---------- ----------

Cre09.g404550 FT-------- ---------- -----WQ--- ------ATGG TKPVYNLQGT WTEADGFTLT DPVIHHARGH KLNGATDEGL EGVRSFFRTH TCN-------

Cre02.g108750b TAGVNS---- -------AQH SPTAAAAT-- -----PAAGA AAGALS---- ---------- ---------- ---------- ---------- --------EA

Cre02.g095099 ---------- ---------- ---------- ---------- ---------- ---------- ---------- ---------- ---------- ---------V

Cre17.g710600 ---------- ---------- ---------- ---------- ---------- ---------- ---------- ---------- ---------- ---------A

CrMAPKKK14 ---------- ---------- ---------- ---------- ---------- ---------- ---------- ---------- ---------- ----------

CrMAPKKK4 ---------- ---------- ---------- ---------- ---------- ---------- ---------- ---------- ---------- ----------

CrMAPKKK8 LDGD------ ---------- -----GGA-- -----VCP-- --QALE---- --------SI RQE------V QVLSRLSH-P HIVQFFGACL APPHVC--IV

CrMAPKKK1 VDE------- ---------- -----WSG-- -----PTD-- --SLVS---- --------SF AQE------V EVLGRCQH-P NIVRLLAACL KPPRLC--LV

CrMAPKKK12 AAV------- ---------- -----WYD-- -----PEE-- ----LE---- --------LF KRE------V QVLGRLSH-P NIVRLIAVCM APPRLS--LV

Cre09.g390300 ---------- ---------- ---------- ---------- ---------- ---------- ---------- ---------- ---------- ----------

CrMAPKKK11 ---------- ---------- ---------- ---------- ---------- ---------- ---------- ---------- ---------- ----------

CrMAPKKK13 ---------- ---------- ---------- ---------- ---------- ---------- ---------- ---------- ---------- ----------

CrPTK7 QAGAGAVRSA GGVG-----E GSSRGPGG-- -----PGPGA SAGAAAVR-- ---SAGSTIA AHM------L QVMGGAGGGG GGMR------ --GALQ--QL

Cre02.g117813 ---------- ---------- ---------- ---------- ---------- ---------- ---------L QRE------- ---------- ----------

CrMAPKKK10 VAGA--PRNL DSPGQLAIRN --LDSPGL-- -----PGSGA ASPAPE---- ---------- RAS------L QRGGEQADGP AAAA--AAAV AATAAA--TV

CrMAPKKK2 ---------- ---------- ---------- ---------- ---------- ---------- ---------- ---------- ---------- ----------

CrMAPKKK6 ---------- ---------- ---------- ---------- ---------- ---------- ---------- ---------- ---------- ----------

CrMAPKKK5 ---------- ---------- ---------- ---------- ---------- ---------- ---------- ---------- ---------- ----------

CrMAPKKK9 ---------- ---------- ---------- ---------- ---------- ---------- ---------- ---------- ---------- ----------

Cre12.g516650 TAGA--PP-- ---DQLQVRA PLEDMTGH-- -----HQHGG TRPLLE---- ---------- DV-------- ---------- ---------- ----------

CrMAPKKK7 QAGV--TV-- ---PS-APGN APTDATGH-- -----HQHGG TRPLLE---- ---------- DM-------- ---------- ---------- ----------

....|....| ....|....| ....|....| ....|....| ....|....| ....|....| ....|....| ....|....| ....|....| ....|....|

710 720 730 740 750 760 770 780 790 800

CrCDPK9 ME-------- --LCAGGELF DSIV------ ---------- ---------E AGNFTEKKAA L-YFRKMVEV VNHCHE---L GV---MHRDL KPENFLLTSK

AtCPK25 ME-------- --LCRGGELF DRIV------ ---------- ---------E RGHYSERKAA H-LAKVILGV VQTCHS---L GV---MHRDL KPENFLFVND

AtCPK26 ME-------- --LCSGGELF DRII------ ---------- ---------Q RGHYSERKAA E-LIKIIVGV VEACHS---L GV---MHRDL KPENFLLVNK

AtCPK6 ME-------- --LCAGGELF DRII------ ---------- ---------H RGHYSERKAA E-LTKIIVGV VEACHS---L GV---MHRDL KPENFLLVNK

AtCPK5 ME-------- --LCAGGELF DRII------ ---------- ---------Q RGHYSERKAA E-LTKIIVGV VEACHS---L GV---MHRDL KPENFLLVNK

AtCPK20 ME-------- --ICAGGELF DRII------ ---------- ---------Q RGHYTEKKAA E-LARIIVGV IEACHS---L GV---MHRDL KPENFLFVSG

AtCPK2 ME-------- --LCSGGELF DRII------ ---------- ---------Q RGHYTERKAA E-LARTIVGV LEACHS---L GV---MHRDL KPENFLFVSR

AtCPK1 ME-------- --CCAGGELF DRII------ ---------- ---------Q RGHYTERKAA E-LTRTIVGV VEACHS---L GV---MHRDL KPENFLFVSK

AtCPK12 ME-------- --LCEGGELF DRIV------ ---------- ---------K RGHYSEREAA K-LIKTIVGV VEACHS---L GV---VHRDL KPENFLFSSS

AtCPK11 ME-------- --VCEGGELF DRIV------ ---------- ---------S KGHFSEREAV K-LIKTILGV VEACHS---L GV---MHRDL KPENFLFDSP

AtCPK4 ME-------- --VCEGGELF DRIV------ ---------- ---------S KGCFSEREAA K-LIKTILGV VEACHS---L GV---MHRDL KPENFLFDSP

AtCIPK7 ME-------- --LASGGELF SKVL------ ---------- ---------R RGRLPESTAR R-YFQQLASA LRFSHQ---D GV---AHRDV KPQNLLLD--

AtCIPK4 VE-------- --YAAGGELF TKLI------ ---------- ---------R FGRLNESAAR R-YFQQLASA LSFCHR---D GI---AHRDV KPQNLLLD--

AtCIPK22 ME-------- --LAKGGELF SRVT------ ---------- ---------- SNRFTESLSR K-YFRQLISA VRYCHA---R GV---FHRDL KPENLLLD--

AtCIPK11 ME-------- --FVKGGELF NKIS------ ---------- ---------K HGRLSEDLSR R-YFQQLISA VGYCHA---R GV---YHRDL KPENLLID--

AtCIPK14 ME-------- --FAKGGELF AKVS------ ---------- ---------- KGRFCEDLSR R-YFQQLISA VGYCHS---R GI---FHRDL KPENLLLD--

AtCIPK12 ME-------- --YVRGGELF NKVA------ ---------- ---------- KGRLKEEVAR K-YFQQLISA VTFCHA---R GV---YHRDL KPENLLLD--

AtCIPK19 ME-------- --YVKGGELF NKVA------ ---------- ---------- KGRLKEEMAR K-YFQQLISA VSFCHF---R GV---YHRDL KPENLLLD--

AtCIPK18 ME-------- --YVGGGELF NTVA------ ---------- ---------- KGRLPEETAR R-YFQQLISS VSFCHG---R GV---YHRDL KPENLLLD--

AtCIPK13 ME-------- --YVRGGELY NTVA------ ---------- ---------- RGRLREGTAR R-YFQQLISS VAFCHS---R GV---YHRDL KLENLLLD--

AtCIPK16 ME-------- --YVNGGELF EMID------ ---------- ---------R DGKLPEDLAR K-YFQQLISA VDFCHS---R GV---FHRDI KPENLLLD--

AtCIPK5 ME-------- --FVKGGELF CKIS------ ---------- ---------- KGKLHEDAAR R-YFQQLISA VDYCHS---R GV---SHRDL KPENLLLD--

AtCIPK25 ME-------- --YVKGGELF SKIV------ ---------- ---------- KGKLKEDSAR K-YFQQLISA VDFCHS---R GV---SHRDL KPENLLVD--

AtCIPK2 IE-------- --YCKGGELF NKVA------ ---------- ---------- KGKLKEDVAW K-YFYQLISA VDFCHS---R GV---YHRDI KPENLLLD--

AtCIPK10 ME-------- --YCKGGELF NKVA------ ---------- ---------- KGKLRDDVAW K-YFYQLINA VDFCHS---R EV---YHRDI KPENLLLD--

AtCIPK15 ME-------- --HVKGGELF NKVS------ ---------- ---------- TGKLREDVAR K-YFQQLVRA VDFCHS---R GV---CHRDL KPENLLLD--

AtCIPK6 ME-------- --LVRGGELF AKVA------ ---------- ---------- KGRLREDVAR V-YFQQLISA VDFCHS---R GV---YHRDL KPENLLLD--

AtCIPK20 ME-------- --YVKGGELF DKVS------ ---------- ---------- KGKLKENIAR K-YFQQLIGA IDYCHS---R GV---YHRDL KPENLLLD--

AtCIPK21 ME-------- --YVSGGQLS DRLG------ ---------- ---------R -QKMKESDAR K-LFQQLIDA VDYCHN---R GV---YHRDL KPQNLLLD--

AtCIPK17 LE-------- --CVTGGDLF DRIV------ ---------- ---------S KGKLSETQGR K-MFQQLIDG VSYCHN---K GV---FHRDL KLENVLLD--

AtCIPK1 ME-------- --LVTGGELF DRIV------ ---------- ---------S NGKLTETDGR K-MFQQLIDG ISYCHS---K GV---FHRDL KLENVLLD--

AtCIPK3 LE-------- --YVTGGELF DKIV------ ---------- ---------N DGRMKEDEAR R-YFQQLIHA VDYCHS---R GV---YHRDL KPENLLLD--

AtCIPK9 LE-------- --LVNGGELF DKIA------ ---------- ---------Q QGRLKEDEAR R-YFQQLINA VDYCHS---R GV---YHRDL KPENLILD--

AtCIPK23 LE-------- --FVTGGELF DKIS------ ---------- ---------S NGRLKEDEAR K-YFQQLINA VDYCHS---R GV---YHRDL KPENLLLD--

AtCIPK8 LE-------- --YITGGELF DKIV------ ---------- ---------R NGRLSESEAR K-YFHQLIDG VDYCHS---K GV---YHRDL KPENLLLD--

AtCIPK24 LE-------- --FVTGGELF DRIV------ ---------- ---------H KGRLEESESR K-YFQQLVDA VAHCHC---K GV---YHRDL KPENLLLD--

CrMAPK7 FE-------- --YLEC-NVY ---------- ---------- -QLT-K--DR DKFLPESRIR N-WCYQIFQG LAYIHK---H GF---FHRDM KPENLLASK-

CrMAPK9 FE-------- --YVER-NLL ---------- ---------- -EIL-E--EH PGGLEGEQVR N-YIYQLIKA VGWCHQ---H NI---VHRDI KPENLLISPS

CrMAPK13 FD-------- --LANR-SLL ---------- ---------- -HEMRK--HP RGVVPSGMAK V-VAWQLLRA LEHCHK---Q GV---VHRDV KPANVLIGQP

Cre03.g206202 LE-------- --YVNG-CAS ---------- ---------- -TLL-E--SC PRGLSPVALK L-LAWQMCLA LSHMHT-GQT QL---LHRDI KPSNILVST-

CrMAPK14 FE-------- --YVER-TLL ---------- ---------- -QEL-K--AN RGGMPPAAVK S-LTWQLLQS LSYLHR---K QI---IHRDV KPSNILITE-

CrMAPK17 FD-------- --YGGS-SAQ ---------- ---------- -QVLED--RY PLGFPGPQLK R-LAFQLLQA LKYLHS---R KI---LHRDV KPANVLLDS-

CrMAPK16 LE-------- --YLPR-CVA ---------- ---------- -TEL-D--AR PSGLSRRDVK L-LSWQLASV IRYLHA---C RV---VHRDV KPANILLTD-

CrMAPK15 FE-------- --YMRT-TVH ---------- ---------- -AML-D--CM PCGLPAPITK V-IAWQLLRA TTYLHE---R KI---VHRDI KPANVLVDPC

CrMAPK12 FE-------- --FVGP-SAH ---------- ---------- -DML-D--AH PEGLPRDALK L-VCWQLLRA AAYLHQ---E KL---LHRDI KPANVLVDTR

CrMAPK10 FD-------- --YAGS-SVQ ---------- ---------- -SVM-E--RA GGGLGGAATK L-LSWQLLQA AAHLHG---N KV---LHRDI KPANILVDS-

CrMAPK11 FE-------- --FAGS-SVH ---------- ---------- -DQL-D--RY PTGLGAAATK L-LSWQLLQA AAYLHG---N KV---LHRDI KPANILVDS-

CrGSK3 LE-------- --FVPD-TVY ---------- ---------- -RISKHYAKN NQRMPNLFVK L-YAYQMCRA LNSIHK---M GI---CHRDI KPQNLLVNTE

CrCDKI1 QE-------- --YCTT-DLA ---------- ---------- -ALLR---RL PAPPPERIAK G-LMLQLCRG LEALHA---E GI---MHRDV KPSNTLLSAA

CrMAPK5 FE-------- --YMET-DLH ---------- ---------- -AVIR---AN -ILE-EVHKQ Y-IMYQLFKS LKYMHS---G EL---LHRDI KPSNLLLNS-

CrMAPK8 YE-------- --LMDT-DLH ---------- ---------- -QIIR---SP QALS-NDHSQ Y-FLYQLLRG LKYIHS---A NI---LHRDL KPSNLLVNA-

CrMAPK6 YE-------- --LMDT-DLH ---------- ---------- -QIIR---SS QTLT-NEHFQ Y-FVYQVLRG LKYVHT---A NV---LHRDL KPSNLLLNA-

CrMAPK3 YE-------- --LMDT-DLH ---------- ---------- -QIIR---SS QPLT-NEHFQ Y-FVYQVLRG LKYVHT---A NV---LHRDL KPSNLLLNA-

CrMAPK2 FE-------- --LLET-DLH ---------- ---------- -QVIK---AN DDLT-PEHHQ F-FLYQMLRG IKYIHS---A KV---FHRDL KPKNILANS-

CrMAPK4 FE-------- --LMES-DLH ---------- ---------- -TVIG---AN DDLT-ADHHK V-FLFQLLRG LNFMHS---N GV---LHRDL KPKNILANS-

CrCDPK5 LE-------- --MLTGGELL ---------- ---------- -DHIHKV--- -HNYTERQAA N-LFAQVVSA ISYLHN-LN- -L---MHRDI KPENVMFTAP

CrCDPK4 ME-------- --NCKGGELL ---------- ---------- -HRINT---- -RHYSERTAA S-YMRAVLRT LAQCHH-LR- -I---LHRDI KPGNFMLLHD

CrCDPK3 ME-------- --LCTGGDLF ---------- ---------- -EKIEKM--- -STVTERAAA A-LFRELVEC VAYCHT-LG- -V---MHRDL KPENFLVTDR

PpCCaMK LE-------- --LCRGGELF ---------- ---------- -DRIVQH--- -ERYSERDAA K-VVRQIASG LAALHQ-AQ- -I---VHRDL KPENCLYVNP

NtCCaMK LE-------- --LCSGGELF ---------- ---------- -DRIAGQ--- -ARYNEAGAA A-VVRQIAKG LEALHG-AS- -I---VHRDL KPENCLFLNK

LjCCaMK LE-------- --LCSGGELF ---------- ---------- -DRIVAQ--- -DKYAETEAA A-VVRQIAAG LEAVHK-AD- -I---VHRDL KPENCLFLDS

MtCCaMK LE-------- --LCSGGELF ---------- ---------- -DRIVAQ--- -DKYSETEAA T-VVHQIASG LEAVHR-AN- -I---VHRDL KPENCLFLDV

PsCCaMK LE-------- --LCSGGELF ---------- ---------- -DRIVAQ--- -DKYSETEAS T-VVHQIVAG LEAIHR-AN- -I---IHRDL KPENCLFLDV

LlCCaMK LE-------- --LCSGGELF ---------- ---------- -DRIVAQ--- -DRYSESEAA E-VVQQIASG LAALHK-ST- -I---IHRDL KPENCLFLNQ

OsCCaMK LE-------- --LCSGGELF ---------- ---------- -DRIVGR--- -DRYSEFDAA C-VIRQIASG LEALHK-AS- -I---VHRDL KPENCLFSDK

Cre03.g199050 ME-------- --AVMGGELF ---------- ---------- -SY-LQT--R SRPLEESHAR F-YAASVVLA LEYLHD-RD- -L---VYRDL KPENLLIDLQ

CrCDPKK1 ME-------- --YMEGGPVL ---------- ---------- -TREALE--K RERLPESLAL Q-YFRDMIKA LDYLHG-NK- -V---VHGDL KPENVLMAAS

CrCDPKK2 ME-------- --YVEGGCVL ---------- ---------- -AGSSPT--Q KIPIPEATAV K-YFRDVVKG LEYLHF-NR- -I---VHGDL KPDNLLMSSS

AtMKK10 ME-------- --LMEKGSLH ---------- ---------- -DALLAQ--- -QVFSEPMVS S-LANRILQG LRYLQK-MG- -I---VHGDI KPSNLLINKK

AtMKK4 LE-------- --FMDKGSLE ---------- ---------- -GAHV----- ---WKEQQLA D-LSRQILSG LAYLHS-RH- -I---VHRDI KPSNLLINSA

AtMKK5 LE-------- --FMDQGSLE ---------- ---------- -GAHI----- ---WQEQELA D-LSRQILSG LAYLHR-RH- -I---VHRDI KPSNLLINSA

AtMKK8 MD-------- --YMDLGSLE ---------- ---------- -SLRG----- ---VTEKQLA L-MSRQVLEG KNYLHE-HK- -I---VHRDI KPANLLRSSK

AtMKK7 ME-------- --YMDGGNLE ---------- ---------- -SLRG----- --AVTEKQLA G-FSRQILKG LSYLHS-LK- -I---VHRDI KPANLLLNSR

AtMKK9 ME-------- --YMDGGTLE ---------- ---------- -SLRG----- --GVTEQKLA G-FAKQILKG LSYLHA-LK- -I---VHRDI KPANLLLNSK

CrMAPKK1 LE-------- --YVDGGSLA ---------- ---------- -DVQAKV--- -GKIPENVLS K-MTAKILRA LAYLHREKH- -M---VHRDI KPANILMTIS

AtMKK3 LE-------- --YMNGGSLA ---------- ---------- -DILKVT--- -KKIPEPVLS S-LFHKLLQG LSYLHGVRH- -L---VHRDI KPANLLINLK

CrMAPKK-L GENGTSTGAG GAGAGSGAGA ---------- ---------- -AAGRSG--- -PGLPEPVIA H-IARQLVAG LTYLHRELK- -V---VHRDI KPSNLLLNGA

AtMKK6 LE-------- --YMDRGSLA ---------- ---------- -DVIRQV--- -KTILEPYLA V-VCKQVLLG LVYLHNERH- -V---IHRDI KPSNLLVNHK

AtMEK1 LE-------- --FMDGGSLA ---------- ---------- -DLLKKV--- -GKVPENMLS A-ICKRVLRG LCYIHHERR- -I---IHRDL KPSNLLINHR

AtMKK2 LE-------- --YMDGGSLA ---------- ---------- -DFLKSV--- -KAIPDSYLS A-IFRQVLQG LIYLHHDRH- -I---IHRDL KPSNLLINHR

AtMAPKKK13 LE-------- --YLPNGDVA ---------- ---------- -SHRAGG--- -KIEDETLLQ R-YTACLVSA LRHVHS-QG- -F---VHCDV KARNILVS-Q

AtMAPKKK14 LE-------- --YSPEGDVA ---------- ---------- -----NG--- -GIVNETLLR R-YVWCLVSA LSHVHS-NG- -I---VHCDV KSKNVLVFNG

Cre10.g464100 LE-------- --YVAGGPIS ---------- ---------- -SKLAQF--- -GPLREETVR V-YTKQILRG LEYLHK-QK- -V---MHRDI KGANILVDSN

AtANP2 LE-------- --FVPGGSIS ---------- ---------- -SLLEKF--- -GAFPESVVR T-YTNQLLLG LEYLHN-HA- -I---MHRDI KGANILVDNQ

AtANP1 LE-------- --FVPGGSIS ---------- ---------- -SLLEKF--- -GPFPESVVR T-YTRQLLLG LEYLHN-HA- -I---MHRDI KGANILVDNK

AtANP3 ME-------- --FVPGGSIS ---------- ---------- -SLLEKF--- -GSFPEPVII M-YTKQLLLG LEYLHN-NG- -I---MHRDI KGANILVDNK

Cre03.g169100 ME-------- --CCEGGDLF ---------- ---------- -KKLMLH--- GGRLPEGWVA AEVIAPLLQV LHRMHA-LR- -I---MHRDI KPENIFLTAE

Cre17.g735550 QE-------- --YAPGGDLL ---------- ---------- -GLMAAH--- GGRADEATVV KTVLRPLLEA LLYLHR-WG- -I---VHRDV KPENVLFAAD

CrALK3 QE-------- --YADGSDLF ---------- ---------- -TLLHKY--- GGRLSERLAV QLVLEPFLRV LQYLHS-RA- -I---IHRDI KPENILFNKA

CrALK1 FE-------- --YASKGDVF ---------- ---------- -TEVERR--- GGQLDEAETV RAVVYPFLSA LDYLHS-RC- -I---IHRDI KPENLLFTAT

CrMAPKKK3 TQ-------- --FVARGSLF ---------- ---------- -RLLHRT--- KEVLDPRRRL N-MSLDIAKG MEYLHN-CKP VL---VHRDL KSPNLLVDRD

AtEDR1 TE-------- --FLPRGSLY ---------- ---------- -RILHRP--- KSHIDERRRI K-MALDVAMG MNCLHT-STP TI---VHRDL KTPNLLVDNN

AtCTR1 TE-------- --YLSRGSLY ---------- ---------- -RLLHKS-GA REQLDERRRL S-MAYDVAKG MNYLHN-RNP PI---VHRDL KSPNLLVDKK

CrCNK9 VE-------- --LCESGDLF ---------- ---------- -TQIKLRAQS GVHFSEEHLQ E-MAVQLLSA VAYLHR-SS- -I---AHRDL KSSNVLITGE

AtNEK7 TA-------- --YYEGGNMA ---------- ---------- -NAIK--KAR GKLFPEERIF K-WLAQLLLA VNYLHS-NR- -V---VHMDL TCSNIFLPKD

AtNEK5 TS-------- --YCEGGDMT ---------- ---------- -QMIK--KSR GVFASEEKLC R-WMVQLLLA IDYLHN-NR- -V---LHRDL KCSNIFLTKE

AtNEK6 TG-------- --YCEGGDMA ---------- ---------- -ELMK--KSN GVYFPEEKLC K-WFTQLLLA VEYLHS-NY- -V---LHRDL KCSNIFLTKD

AtNEK4 IG-------- --YCQGGDMT ---------- ---------- -DTIK--RAC GVHFPEEKLC Q-WLVQLLMA LDYLHS-NH- -I---LHRDV KCSNIFLTKE

AtNEK1 IG-------- --YCEGGDMA ---------- ---------- -QAIK--KSN GVHFQEEKLC K-WLVQLLMG LEYLHS-NH- -I---LHRDV KCSNIFLTKE

AtNEK2 IG-------- --YCKGGDMA ---------- ---------- -EAIK--KTN GVHFTEEKLC K-WLVQILLA LEYLHA-NH- -I---LHRDV KCSNIFLTKD

AtNEK3 IG-------- --YCKGGDMA ---------- ---------- -EAIK--KAN GVEFSEEKLC K-WLVQLLMA LEYLHA-SH- -I---LHRDV KCSNIFLTKD

CrCNK7 LE-------- --WAEAGDLG ---------- ---------- -QLIKQRAEA GQPFSEEQVW R-QFQQVCNA LKHMHD-RR- -M---MHRDL KPSNIFVTAS

CrCNK6 FE-------- --WAPAGDLK ---------- ---------- -RLIKKTAEQ GKTLDEPSIW T-LFYQVTDG LRYMHQ-HR- -I---MHRDI KPANVLVGAN

CrCNK4 TE-------- --YAANGNLH ---------- ---------- -DYIKKQKS- --RLTEDLIW K-LYIQILLG LNHMHS-KK- -I---LHRDI KTLNVFLDED

CrFA2 ME-------- --YASKGSVR ---------- ---------- -QLVKSYRG- -RPLPEEGVW R-IFIQTLIG LSYLHS-KK- -I---IHRDI KSANLFIDAY

CrCNK1 ME-------- --YAPFGDLR ---------- ---------- -YYISKGAKL RTPFPEEAVW R-IFLQLCKG LQALHS-QN- -I---IHRDI KPANIFLCAN

CrCNK2 ME-------- --YAADGDLA ---------- ---------- -KVIKKQQMM KRPLPEDMIW K-YFIQVVMG LQALHS-MK- -I---LHRDI KPGNIMVFDN

CrCNK8 MA-------- --FCEGGDLF ---------- ---------- -KYIRELRDK GQTVPEPQVW A-WLVQLLLS LSYIHS-KR- -I---LHRDV KTQNIFLSGG

CrCNK5 TS-------- --FCEEGDLF ---------- ---------- -NRIRKKAAA KEYFTEDEVM N-MFVQIASA ISYIHS-KR- -V---LHRDL KTQNIFIAKG

CrFAP403 MD-------- --WCSEGDLY ---------- ---------- -GLIQKRRG- -QQLSEDTIL D-WLVQMCLG LKHVHD-RK- -I---LHRDI KTQNVFMSSG

CrCNK3 ME-------- --YANGGDLA ---------- ---------- -AAIQRRQAE KKPYSEDEIM F-WFVQIVLA LYHVHG-RN- -V---LHRDL KSQNIFIAEG

CrCDPKK3 MG-------- --YV------ ---------- -EGATL---- -QPPQVAPGR WKPVPEQVVW R-HCRDVLCG LEYLHC-HS- -V---VHGDL KPANFIQDSL

CrMAPK1 FE-------- --LM------ ---------- -DMN------ ---------- ---------- ---------- ---------- ---------- ----------

Cre08.g379600 ---------- ------PPQQ QQQQ------ -----QQQQQ QQQGQQVQGP FQAYTEQQAL T-WLLDVAAA LAFLHG-SSP TV---IHRDV KAENVLLQSE

Cre02.g108601 ---------- ---------- ---------- ---------- -----MTD-N RRTYTNLQAY T-WLTQLASA LAHLHS-HRP PI---IHRDI KQENLLLKKV

Cre02.g108750a ---------- ---------- ---------- ---------- -----IISGR TKAYSTAQAL Q-WCEQVAEA LAYLHR-QKP CI---IHRDV KQENILFQR-

Cre17.g713750 RM-------- --ARAPPGFP VPGT------ ----AS---- APRCRDVWED VALLPLQQVL A-ILVSLAAA IKAMHR-V-- GL---VHNDV KPANMLHRA-

Cre11.g467586 RV-------- --ARAPPGFP VPGT------ ----AS---- APRCRDVWED VALLPLKQVL A-ILVSLAAA IKAMHR-V-- GL---VHNDV KPANMLHRA-

Cre11.g467588 RV-------- --ARAPPGFP VPGT------ ----AS---- APRCRDVWED VALLPLKQVL A-ILVSLAAA IKAMHR-V-- GL---VHNDV KPANMLHRA-

Cre11.g467589 RV-------- --ARAPPGFP VPGT------ ----AS---- APRCRDVWED VALLPLKQVL A-ILVSLAAA IKAMHR-V-- GL---VHNDV KPANMLHRA-

Cre11.g467584 HV-------- --ARAPPGFP VPGT------ ----AS---- APRCRDVWED VALLPLKQVL A-ILVFLAAA IKAMHR-V-- GL---VHNDV KPANMLHRA-

Cre11.g467585 RV-------- --ARAPPGFP VPGT------ ----AS---- APRCRDVWED VALLPLKQVL A-ILVSLAAA IKAMHR-V-- GL---VHNDV KPANMLHRA-

Cre12.g489850 MD-------- --Q------- ---E------ ----AF---- RQRQQLSCDG MSVLPLPTVF K-VLGTVATA LAALHK-H-- GY---AHQDV NPNNILVRA-

Cre12.g490200 DE-------- --A------- ---------- ---------- QARPDFCRGQ TTVLPMRDVF R-VLRAVTSA LAATHR-L-- GF---VHQDV KTDNVLCRD-

Cre08.g384250 ---------- ---------- ---------- -----YQEGV QTLDKLQETH GGRLPQSVAR E-LFLQLMIA IEFCHR---I GL---SNRDC KITDLLVRVC

Cre04.g217940 ---------- ---------- ---------- -----A---- AFDADSEDTS GYRLPETAVR V-VLASVLLG LRDLHG-R-A RL---AHRDL KLDNLLVGT-

Cre04.g217923 ---------- ---------- ---------- -----A---- AFDADSEDTS GYRLPETAVR V-VLASVLLG LRDLHG-R-A RL---AHRDL KLDNLLVGT-

Cre04.g217928 ---------- ---------- ---------- -----A---- AFDADSEDTS GYRLPETAVR V-VLASVLLG LRDLHG-R-A RL---AHRDL KLDNLLVGT-

Cre08.g369700 ---------- ---------- ---------- -----D---- LWGANLAQHP DQGLPTAALQ V-VAASVLLA LRALHA-I-- GM---AHLDL KPENIILGL-

Cre08.g369900 ---------- ---------- ---------- -----D---- LWGANLAQHP DQGLPTAALQ V-VAASVLLA LRALHA-I-- GM---AHLDL KPENIILGS-

Cre03.g187300 ---------- --TSNKPAVP VTAAPAA--- ---AAAA-PA AVLPPRMPPS EMLMAESRLR V-VAASLLHA LYGLHV-Q-- GF---VHLHI TPANVLCSG-

Cre01.g041752 ---------- --AKPKPEKP RRQNPSSSNK TTGKTATPPP APPPALVLTG RTLVPEPLLK R-IAVALLSA LAPLHE-A-- GI---CHGAI APDCVSVVT-

Cre12.g495401 ---------- --AKPKPEKP RRQNPSSSNK TTGKTATPPP APPPALVLTG RTLVPEPLLK R-IAVALLSA LVPLHE-A-- GI---CHGAI APDCVSVVT-

Cre06.g266450 ---------- ---------- LKKV------ ----KKS--- -KRAKAPK-P HTILSTPLLK Q-LAVSVLTA VDAMHN-N-- NM---CHGDI KGDNVFVDL-

Cre06.g266000 ---------- ---------- KKG------- -----KK--- -GKTLTPR-T STILTTPLLK Q-LAISVLTA VDVMHA-N-- NM---AHGDI KSDNVYVNT-

Cre06.g266250 ---------- ---------- KKGK------ ---KGKK--- -GKTLIPR-T STILSTPLLK Q-LAISVLTA VNVMHN-N-- NM---CHGDI KSDNVYVNT-

Cre10.g460700 ---------- ------PPTA KRTG------ ---QNKD--- -KYERLPYTG ATLMSEPELR A-LAIALLQV VKVMHA-R-- GY---GHCDI KPANVLVDH-

Cre12.g545750 ---------- ------PEGR RRTG------ ---KNKD--- -KYEMLPYPG NTLMDEADLK G-MLRAMALF IKHLNA-R-- GY---MHFDL KPANLLYDI-

Cre12.g525950 ---------- ------PE-R RRTG------ ---KNKD--- -KYEMLPYPG NTLMDEADLK G-MLRAMAPF IKHLNA-R-- GY---MHFDL KPANLLYDI-

Cre12.g525750 ---------- ------PE-R RRTG------ ---KNKD--- -KYEMLPYPG NTLMDEADLK G-MLRAMALF IKHLNA-R-- GY---MHFDL KPANLLYDI-

Cre12.g526650 ---------- ------PE-R RRTG------ ---KNKD--- -KYEMLPYPG NTLMDEADLK G-MLRAMALF IKHLNA-R-- GY---MHFDL KPANLLYDI-

Cre12.g526051 ---------- ------PE-R RRTG------ ---KNKD--- -KYEMLPYPG NTLMDEADLK G-MLRAMALF IKHLNA-R-- GY---MHFDL KPANLLYDI-

Cre12.g526250 ---------- ------PE-R RRTG------ ---KNKD--- -KYEMLPYPG NTLMDEADLK G-MLRAMALF IKHLNA-R-- GY---MHFDL KPANLLYDI-

Cre01.g054750 ---------- ------PGRP RRTG------ ---KNKD--- -KCELLPYPG STLTDEADMR G-MLLAMMHT VKHMNS-R-- GY---AHFDL KPANLLFDT-

Cre01.g055457 ---------- ------RSRP RRTG------ ---KNKD--- -KYELLPYPG STLTDEADMR G-MLLAMMHT IKHMNS-R-- GY---VHFDL KPANLLFDT-

Cre09.g404550 ---------- ---------- ---------- ---------- ---------- ---------- ---------- ---------- ---------- -----ALCRR

Cre02.g108750b R--------- ---------- ---------- ---------- ---------- --------SG S-LQQPMAAA LHKSRS-LKQ RASGGPGGGR ASADVALVTP

Cre02.g095099 SA-------- --AA------ ---------- ---------- -AAAPPQPPR VP--RLPLGL A-ARPPIAPV LSQSTG---- -P---SHQPM APP--PLPLP

Cre17.g710600 RQ-------- --QQ------ ---------- ---------- PEAAGVTGTD GPQLDMVGVL A-VARDIARG LSHLHA-KG- -I---VHGDM KMENVMLCRN

CrMAPKKK14 ---------- ---------A A--------- ---------- -AAAAGAQQP PPAVDMMGVY L-TLLDVAMA LRYLHG-LNP PV---AHRDV KAANVLLRSS

CrMAPKKK4 ---------- ---------Q ---------- ---------- ------PDRH PFTLDLRGVY M-VLLDVALA LRHLHS-MN- -L---VHRDL KPANLLLKSN

CrMAPKKK8 EE-------- --LAAAGSLH HRLH------ ----AR---- RR-GS-SVKL HPAMSYGEVL Q-LGLDVSSA MCYLHP--H- -V---VHRDL KPQNVLLDGS

CrMAPKKK1 ME-------- --LMDTS-LE RLVH------ ----G----- --------KP GELLPLPTVL H-VAADIARG LAYLHP--T- -I---VHRDL KPGNVLLSNT

CrMAPKKK12 ME-------- --AAETS-LD KVVY------ ----GG---- GG-GAPGGGG GELLPLPKVL H-IAVQIAQG LAYLHP--T- -V---CHGDL KPANVLLNGA

Cre09.g390300 ---------- ---------- ---------- ---------- ---------- ---RSDVVRV K-LALDAARG MEYLHL-KR- -I---VHFDL KTGNLLVGFR

CrMAPKKK11 ---------- ---------- ---------- ---------- ---------- ---LARRVLL R-TATELCRG MVHIHT-AN- -V---LHGDL KPANVLLARS

CrMAPKKK13 ---------- ---------- ---------- ---------- ---------- ---WDAWACL Q-TLKEVTKA LMFLHE-NR- -I---LHGDL KAANVLLSSS

CrPTK7 RQ-------- --QLQPP--R RSLS------ ----AGR--- VPPISPDKPP APAYRYGLML S-VACDVASA LLHLHS-HN- -I---VHGDV KVRTAAVPWR

Cre02.g117813 ---------- ---------- ---------- ---------- ------QQKD AALAHMRGVL S-VARDIAAG MAYLHS-LN- -V---CHGDL KCENVLLCYK

CrMAPKKK10 RA-------- --AAARA-VA ARAA------ ----AEH--- EDPVPGGPPP P--------- ----PPPGAG LAEVVA-KA- -V---IPGRR NPDSPTLGLP

CrMAPKKK2 ---------- ---------- ---------- ---------- ---------- --GAAGLCAL T-LALDVACG MRHIHA-RN- -I---VHGDL SAGNILLCST

CrMAPKKK6 ---------- ---------- ---------- ---------- ---------- ----RMEMIL D-TSVELCGG LCHLHE-RN- -I---VHGDL NPNNVLLKRD

CrMAPKKK5 ---------- ---------- ---------- ---------- ---------- ----NLAAVV P-LALGIARA LAHLHS-KR- -I---VHGDL NPNNVLLKRD

CrMAPKKK9 ---------- ---------- ---------- ---------- ---------- ----ELAAVV P-LALGIARA LAHLHS-KR- -I---VHGDL NPNNVLLRQD

Cre12.g516650 ---------- ---------- ---------- ---------- PADVSGGRPA SSLQAALRYV E-AALQIARG LQHIHE-KN- -I---VHGDL NPNNVLLVRA

CrMAPKKK7 ---------- ---------- ---------- ---------- PADVSGGRPA SSLQAALRYV E-AALQIARG LQHIHD-KN- -I---VHGDL NPNNVLLVRA

....|....| ....|....| ....|....| ....|....| ....|....| ....|....| ....|....| ....|....| ....|....| ....|....|

810 820 830 840 850 860 870 880 890 900

CrCDPK9 GPE------- ---------- ---------- ---------- ---------- ---------- ---------- ---------- ---------- ----------

AtCPK25 DED------- ---------- ---------- ---------- ---------- ---------- ---------- ---------- ---------- ----------

AtCPK26 DDD------- ---------- ---------- ---------- ---------- ---------- ---------- ---------- ---------- ----------

AtCPK6 DDD------- ---------- ---------- ---------- ---------- ---------- ---------- ---------- ---------- ----------

AtCPK5 DDD------- ---------- ---------- ---------- ---------- ---------- ---------- ---------- ---------- ----------

AtCPK20 DEE------- ---------- ---------- ---------- ---------- ---------- ---------- ---------- ---------- ----------

AtCPK2 EED------- ---------- ---------- ---------- ---------- ---------- ---------- ---------- ---------- ----------

AtCPK1 HED------- ---------- ---------- ---------- ---------- ---------- ---------- ---------- ---------- ----------

AtCPK12 DED------- ---------- ---------- ---------- ---------- ---------- ---------- ---------- ---------- ----------

AtCPK11 KDD------- ---------- ---------- ---------- ---------- ---------- ---------- ---------- ---------- ----------

AtCPK4 SDD------- ---------- ---------- ---------- ---------- ---------- ---------- ---------- ---------- ----------

AtCIPK7 -EQ------- ---------- ---------- ---------- ---------- ---------- ---------- ---------- ---------- ----------

AtCIPK4 -KQ------- ---------- ---------- ---------- ---------- ---------- ---------- ---------- ---------- ----------

AtCIPK22 -EN------- ---------- ---------- ---------- ---------- ---------- ---------- ---------- ---------- ----------

AtCIPK11 -EN------- ---------- ---------- ---------- ---------- ---------- ---------- ---------- ---------- ----------

AtCIPK14 -EK------- ---------- ---------- ---------- ---------- ---------- ---------- ---------- ---------- ----------

AtCIPK12 -EN------- ---------- ---------- ---------- ---------- ---------- ---------- ---------- ---------- ----------

AtCIPK19 -EN------- ---------- ---------- ---------- ---------- ---------- ---------- ---------- ---------- ----------

AtCIPK18 -NK------- ---------- ---------- ---------- ---------- ---------- ---------- ---------- ---------- ----------

AtCIPK13 -DK------- ---------- ---------- ---------- ---------- ---------- ---------- ---------- ---------- ----------

AtCIPK16 -GE------- ---------- ---------- ---------- ---------- ---------- ---------- ---------- ---------- ----------

AtCIPK5 -EN------- ---------- ---------- ---------- ---------- ---------- ---------- ---------- ---------- ----------

AtCIPK25 -EN------- ---------- ---------- ---------- ---------- ---------- ---------- ---------- ---------- ----------

AtCIPK2 -DN------- ---------- ---------- ---------- ---------- ---------- ---------- ---------- ---------- ----------

AtCIPK10 -DN------- ---------- ---------- ---------- ---------- ---------- ---------- ---------- ---------- ----------

AtCIPK15 -EH------- ---------- ---------- ---------- ---------- ---------- ---------- ---------- ---------- ----------

AtCIPK6 -EE------- ---------- ---------- ---------- ---------- ---------- ---------- ---------- ---------- ----------

AtCIPK20 -EN------- ---------- ---------- ---------- ---------- ---------- ---------- ---------- ---------- ----------

AtCIPK21 -SK------- ---------- ---------- ---------- ---------- ---------- ---------- ---------- ---------- ----------

AtCIPK17 -AK------- ---------- ---------- ---------- ---------- ---------- ---------- ---------- ---------- ----------

AtCIPK1 -AK------- ---------- ---------- ---------- ---------- ---------- ---------- ---------- ---------- ----------

AtCIPK3 -SY------- ---------- ---------- ---------- ---------- ---------- ---------- ---------- ---------- ----------

AtCIPK9 -AN------- ---------- ---------- ---------- ---------- ---------- ---------- ---------- ---------- ----------

AtCIPK23 -AN------- ---------- ---------- ---------- ---------- ---------- ---------- ---------- ---------- ----------

AtCIPK8 -SQ------- ---------- ---------- ---------- ---------- ---------- ---------- ---------- ---------- ----------

AtCIPK24 -TN------- ---------- ---------- ---------- ---------- ---------- ---------- ---------- ---------- ----------

CrMAPK7 ---------- ---------- ---------- ---------- ---------- ---------- ---------- ---------- ---------- ----------

CrMAPK9 ---------- ---------- ---------- ---------- ---------- ---------- ---------- ---------- ---------- ----------

CrMAPK13 QADPA----- -G---RGPP- ---------- ---------- ---------- ---------- -----SPAAA AGAAA----- ---------- ----------

Cre03.g206202 ---------- ---------- ---------- ---------- ---------- ---------- ---------- ---------- ---------- ----------

CrMAPK14 ---------- ---------- ---------- ---------- ---------- ---------- ---------- ---------- ---------- ----------

CrMAPK17 ---------- ---------- ---------- ---------- ---------- ---------- ---------- ---------- ---------- ----------

CrMAPK16 ---------- ---------- ---------- ---------- ---------- ---------- ---------- ---------- ---------- ----------

CrMAPK15 ---------- ---------- ---------- ---------- ---------- ---------- ---------- ---------- ---------- ----------

CrMAPK12 ---------- ---------- ---------- ---------- ---------- ---------- ---------- ---------- ---------- ----------

CrMAPK10 ---------- ---------- ---------- ---------- ---------- ---------- ---------- ---------- ---------- ----------

CrMAPK11 ---------- ---------- ---------- ---------- ---------- ---------- ---------- ---------- ---------- ----------

CrGSK3 ---------- ---------- ---------- ---------- ---------- ---------- ---------- ---------- ---------- ----------

CrCDKI1 ---------- ---------- ---------- ---------- ---------- ---------- ---------- ---------- ---------- ----------

CrMAPK5 ---------- ---------- ---------- ---------- ---------- ---------- ---------- ---------- ---------- ----------

CrMAPK8 ---------- ---------- ---------- ---------- ---------- ---------- ---------- ---------- ---------- ----------

CrMAPK6 ---------- ---------- ---------- ---------- ---------- ---------- ---------- ---------- ---------- ----------

CrMAPK3 ---------- ---------- ---------- ---------- ---------- ---------- ---------- ---------- ---------- ----------

CrMAPK2 ---------- ---------- ---------- ---------- ---------- ---------- ---------- ---------- ---------- ----------

CrMAPK4 ---------- ---------- ---------- ---------- ---------- ---------- ---------- ---------- ---------- ----------

CrCDPK5 VND------- ---------- ---------- ---------- ---------- ---------- ---------- ---------- ---------- ----------

CrCDPK4 ADN------- ---------- ---------- ---------- ---------- ---------- ---------- ---------- ---------- ----------

CrCDPK3 TGD------- ---------- ---------- ---------- ---------- ---------- ---------- ---------- ---------- ----------

PpCCaMK LAE------- ---------- ---------- ---------- ---------- ---------- ---------- ---------- ---------- ----------

NtCCaMK DEN------- ---------- ---------- ---------- ---------- ---------- ---------- ---------- ---------- ----------

LjCCaMK RKD------- ---------- ---------- ---------- ---------- ---------- ---------- ---------- ---------- ----------

MtCCaMK RKD------- ---------- ---------- ---------- ---------- ---------- ---------- ---------- ---------- ----------

PsCCaMK GKD------- ---------- ---------- ---------- ---------- ---------- ---------- ---------- ---------- ----------

LlCCaMK EKR------- ---------- ---------- ---------- ---------- ---------- ---------- ---------- ---------- ----------

OsCCaMK DEK------- ---------- ---------- ---------- ---------- ---------- ---------- ---------- ---------- ----------

Cre03.g199050 G-Y------- ---------- ---------- ---------- ---------- ---------- ---------- ---------- ---------- ----------

CrCDPKK1 G-E------- ---------- ---------- ---------- ---------- ---------- ---------- ---------- ---------- ----------

CrCDPKK2 G-K------- ---------- ---------- ---------- ---------- ---------- ---------- ---------- ---------- ----------

AtMKK10 G-E------- ---------- ---------- ---------- ---------- ---------- ---------- ---------- ---------- ----------

AtMKK4 K-N------- ---------- ---------- ---------- ---------- ---------- ---------- ---------- ---------- ----------

AtMKK5 K-N------- ---------- ---------- ---------- ---------- ---------- ---------- ---------- ---------- ----------

AtMKK8 E-E------- ---------- ---------- ---------- ---------- ---------- ---------- ---------- ---------- ----------

AtMKK7 N-E------- ---------- ---------- ---------- ---------- ---------- ---------- ---------- ---------- ----------

AtMKK9 N-E------- ---------- ---------- ---------- ---------- ---------- ---------- ---------- ---------- ----------

CrMAPKK1 G-E------- ---------- ---------- ---------- ---------- ---------- ---------- ---------- ---------- ----------

AtMKK3 G-E------- ---------- ---------- ---------- ---------- ---------- ---------- ---------- ---------- ----------

CrMAPKK-L G-E------- ---------- ---------- ---------- ---------- ---------- ---------- ---------- ---------- ----------

AtMKK6 G-E------- ---------- ---------- ---------- ---------- ---------- ---------- ---------- ---------- ----------

AtMEK1 G-E------- ---------- ---------- ---------- ---------- ---------- ---------- ---------- ---------- ----------

AtMKK2 G-E------- ---------- ---------- ---------- ---------- ---------- ---------- ---------- ---------- ----------

AtMAPKKK13 SSM------- ---------- ---------- ---------- ---------- ---------- ---------- ---------- ---------- ----------

AtMAPKKK14 GSS------- ---------- ---------- ---------- ---------- ---------- ---------- ---------- ---------- ----------

Cre10.g464100 G-V------- ---------- ---------- ---------- ---------- ---------- ---------- ---------- ---------- ----------

AtANP2 G-C------- ---------- ---------- ---------- ---------- ---------- ---------- ---------- ---------- ----------

AtANP1 G-C------- ---------- ---------- ---------- ---------- ---------- ---------- ---------- ---------- ----------

AtANP3 G-C------- ---------- ---------- ---------- ---------- ---------- ---------- ---------- ---------- ----------

Cre03.g169100 G-D------- ---------- ---------- ---------- ---------- ---------- ---------- ---------- ---------- ----------

Cre17.g735550 G-T------- ---------- ---------- ---------- ---------- ---------- ---------- ---------- ---------- ----------

CrALK3 M-C------- ---------- ---------- ---------- ---------- ---------- ---------- ---------- ---------- ----------

CrALK1 G-V------- ---------- ---------- ---------- ---------- ---------- ---------- ---------- ---------- ----------

CrMAPKKK3 W-T------- ---------- ---------- ---------- ---------- ---------- ---------- ---------- ---------- ----------

AtEDR1 W-N------- ---------- ---------- ---------- ---------- ---------- ---------- ---------- ---------- ----------

AtCTR1 Y-T------- ---------- ---------- ---------- ---------- ---------- ---------- ---------- ---------- ----------

CrCNK9 G-C------- ---------- ---------- ---------- ---------- ---------- ---------- ---------- ---------- ----------

AtNEK7 D-H------- ---------- ---------- ---------- ---------- ---------- ---------- ---------- ---------- ----------

AtNEK5 N-E------- ---------- ---------- ---------- ---------- ---------- ---------- ---------- ---------- ----------

AtNEK6 Q-D------- ---------- ---------- ---------- ---------- ---------- ---------- ---------- ---------- ----------

AtNEK4 Q-D------- ---------- ---------- ---------- ---------- ---------- ---------- ---------- ---------- ----------

AtNEK1 Q-D------- ---------- ---------- ---------- ---------- ---------- ---------- ---------- ---------- ----------

AtNEK2 Q-D------- ---------- ---------- ---------- ---------- ---------- ---------- ---------- ---------- ----------

AtNEK3 Q-D------- ---------- ---------- ---------- ---------- ---------- ---------- ---------- ---------- ----------

CrCNK7 G-D------- ---------- ---------- ---------- ---------- ---------- ---------- ---------- ---------- ----------

CrCNK6 G-A------- ---------- ---------- ---------- ---------- ---------- ---------- ---------- ---------- ----------

CrCNK4 L-N------- ---------- ---------- ---------- ---------- ---------- ---------- ---------- ---------- ----------

CrFA2 D-N------- ---------- ---------- ---------- ---------- ---------- ---------- ---------- ---------- ----------

CrCNK1 D-L------- ---------- ---------- ---------- ---------- ---------- ---------- ---------- ---------- ----------

CrCNK2 G-V------- ---------- ---------- ---------- ---------- ---------- ---------- ---------- ---------- ----------

CrCNK8 --K------- ---------- ---------- ---------- ---------- ---------- ---------- ---------- ---------- ----------

CrCNK5 G-I------- ---------- ---------- ---------- ---------- ---------- ---------- ---------- ---------- ----------

CrFAP403 G-L------- ---------- ---------- ---------- ---------- ---------- ---------- ---------- ---------- ----------

CrCNK3 N-L------- ---------- ---------- ---------- ---------- ---------- ---------- ---------- ---------- ----------

CrCDPKK3 TGVV------ ---------- ---------- ---------- ---------- ---------- ---------- ---------- ---------- ----------

CrMAPK1 ---------- ---------- ---------- ---------- ---------- ---------- ---------- ---------- ---------- ----------

Cre08.g379600 AAAG------ ---------- ---------- ---------- ---------- -----PAGGA G------AAA GGGG----GA GGGGRLVAKL ADLGLHVQVD

Cre02.g108601 TRPP------ ---------- ---------- ---------- ---------- -----PAAAA AAGGGVPAGA QRLP----GG GGGTQLEAKL ADLGLHVVVE

Cre02.g108750a ---------- ---------- ---------- ---------- ---------- ---------- ---------- ---------R GGGTQLEAKL ADLGLHVAIE

Cre17.g713750 ---------- ---------- ---------- ---------- ---------- ---------- ---------- ---------- ----DDTHCL ADFGLAERLS

Cre11.g467586 ---------- ---------- ---------- ---------- ---------- ---------- ---------- ---------- ----DDTYCL ADFGLAEQLS

Cre11.g467588 ---------- ---------- ---------- ---------- ---------- ---------- ---------- ---------- ----DDTYCL ADFGLAEQLS

Cre11.g467589 ---------- ---------- ---------- ---------- ---------- ---------- ---------- ---------- ----DDTYCL ADFGLAEQLS

Cre11.g467584 ---------- ---------- ---------- ---------- ---------- ---------- ---------- ---------- ----DDTYCL ADFGLAEQLS

Cre11.g467585 ---------- ---------- ---------- ---------- ---------- ---------- ---------- ---------- ----DDTYCL ADFGLAEQLS

Cre12.g489850 ---------- ---------- ---------- ---------- ---------- ---------- ---------- ---------- ----DGSVAL SDFGLAVSIS

Cre12.g490200 ---------- ---------- ---------- ---------- ---------- ---------- ---------- ---------- ----DGSYCL GDFGCVAKIP

Cre08.g384250 PASG------ ---------- ---------- ---------- ---------- ---------- ---------- ---------- ----DVRLQL A---------

Cre04.g217940 ---------- ---------- ---------- ---------- ---------- ---------- ---------- ---------- ----DRLVKI T---------

Cre04.g217923 ---------- ---------- ---------- ---------- ---------- ---------- ---------- ---------- ----DRLVKI T---------

Cre04.g217928 ---------- ---------- ---------- ---------- ---------- ---------- ---------- ---------- ----DRLIKI T---------

Cre08.g369700 ---------- ---------- ---------- ---------- ---------- ---------- ---------- ---------- ----DGLLKL I---------

Cre08.g369900 ---------- ---------- ---------- ---------- ---------- ---------- ---------- ---------- ----DGLLKL I---------

Cre03.g187300 ---------- ---------- ---------- ---------- ---------- ---------- ---------- ---------- ----IGRVLL ADFGTARCLI

Cre01.g041752 ---------- ---------- ---------- ---------- ---------- ---------- ---------- ---------- ----ASSTE- ----------

Cre12.g495401 ---------- ---------- ---------- ---------- ---------- ---------- ---------- ---------- ----ASSTE- ----------

Cre06.g266450 ---------- ---------- ---------- ---------- ---------- ---------- ---------- ---------- ----SA---- ----------

Cre06.g266000 ---------- ---------- ---------- ---------- ---------- ---------- ---------- ---------- ----GD---- ----------

Cre06.g266250 ---------- ---------- ---------- ---------- ---------- ---------- ---------- ---------- ----GD---- ----------

Cre10.g460700 ---------- ---------- ---------- ---------- ---------- ---------- ---------- ---------- -----A---- ----------

Cre12.g545750 ---------- ---------- ---------- ---------- ---------- ---------- ---------- ---------- -----A---- ----------

Cre12.g525950 ---------- ---------- ---------- ---------- ---------- ---------- ---------- ---------- -----A---- ----------

Cre12.g525750 ---------- ---------- ---------- ---------- ---------- ---------- ---------- ---------- -----A---- ----------

Cre12.g526650 ---------- ---------- ---------- ---------- ---------- ---------- ---------- ---------- -----A---- ----------

Cre12.g526051 ---------- ---------- ---------- ---------- ---------- ---------- ---------- ---------- -----A---- ----------

Cre12.g526250 ---------- ---------- ---------- ---------- ---------- ---------- ---------- ---------- -----A---- ----------

Cre01.g054750 ---------- ---------- ---------- ---------- ---------- ---------- ---------- ---------- -----A---- ----------

Cre01.g055457 ---------- ---------- ---------- ---------- ---------- ---------- ---------- ---------- -----A---- ----------

Cre09.g404550 LG-------- LRAA------ ---------- ---------- ---------- ---------- ---------- ---------- ---------- ----------

Cre02.g108750b F--------- ---------- ---------- ---------- ------ASAV PIATPPGSVG GATPTPGSSA GGASNGNGNG NGNGNGSGEQ GPLPLHTSES

Cre02.g095099 PH--LFAQPG SARGLPFPPA TGR------- ---------- ---------- SLELIAEGCG TEDGGASSRA DSESCWGG-G VGGASGAATT APTTA--RSL

Cre17.g710600 AAAAGRSRT- ---AARTPSA SA-------- ---------- ---------Y AFKAAA--A- --GGGESAAA GGNSWWPF-A VGGGAGVASV YANPVAAVGT

CrMAPKKK14 TS-------- ---------- ---------- ---------- ---------- ---------- ---------- ---------- ---------- ----------

CrMAPKKK4 LR-------- ---------- ---------- ---------- ---------- ---------- ---------- ---------- ---------- ----------

CrMAPKKK8 G--------- ---------- ---------- ---------- ---------- ---------- ---------- ---------- ---------- ----------

CrMAPKKK1 EC-------- ---------- ---------- ---------- ---------- ---------- ---------- ---------- ---------- ----------

CrMAPKKK12 SG-------- ---------- ---------- ---------- ---------- ---------- ---------- ---------- ---------- ----------

Cre09.g390300 EK-------- ---------- ---------- ---------- ---------- ---------- ---------- ---------- ---------- ----------

CrMAPKKK11 RK-------- ---------- ---------- ---------- ---------- ---------- ---------- ---------- ---------- ----------

CrMAPKKK13 ET-------- ---------- ---------- ---------- ---------- ---------- ---------- ---------- ---------- ----------

CrPTK7 RASNVLLKSG SG-------- --------GS RALAACGSSS SGAASAIPAL LLHSPPGGGG GGGAGTPAGS ---------- --------SV GPGPTPAAAA

Cre02.g117813 AGMDRASTGG MGAS------ ---------- ---------- ---------- ---------- ---------- ---------- -----GSVR- ----------

CrMAPKKK10 PAAGLT--GG LATPLRSPPA AIPAAQLDGS DGIATPDA-- AAAAAAAAAA ALGSPGGRGG GGGGGAAATT GGGADGVG-G GGGGGGAARL GGGPSFRASA

CrMAPKKK2 ADAQ------ ---------- ---------- ---------- ---------- ---------- ---------- ---------- ---------- ----------

CrMAPKKK6 PTKK------ ---------- ---------- ---------- ---------- ---------- ---------- ---------- ---------- ----------

CrMAPKKK5 PAEP------ ---------- ---------- ---------- ---------- ---------- ---------- ---------- ---------- ----------

CrMAPKKK9 PTQP------ ---------- ---------- ---------- ---------- ---------- ---------- ---------- ---------- ----------

Cre12.g516650 PGTP------ ---------- ---------- ---------- ---------- ---------- ---------- ---------- ---------- ----------

CrMAPKKK7 PGTP------ ---------- ---------- ---------- ---------- ---------- ---------- ---------- ---------- ----------

....|....| ....|....| ....|....| ....|....| ....|....| ....|....| ....|....| ....|....| ....|....| ....|....|

910 920 930 940 950 960 970 980 990 1000

CrCDPK9 ---------- ---------- ---------- ---------- ---------- ---------- -----GELKL TDFGLGVFFK P--------- ----------

AtCPK25 ---------- ---------- ---------- ---------- ---------- ---------- -----SPLKA IDFGLSMFLK P--------- ----------

AtCPK26 ---------- ---------- ---------- ---------- ---------- ---------- -----FSLKA IDFGLSVFFK P--------- ----------

AtCPK6 ---------- ---------- ---------- ---------- ---------- ---------- -----FSLKA IDFGLSVFFK P--------- ----------

AtCPK5 ---------- ---------- ---------- ---------- ---------- ---------- -----FSLKA IDFGLSVFFK P--------- ----------

AtCPK20 ---------- ---------- ---------- ---------- ---------- ---------- -----AALKT IDFGLSVFFK P--------- ----------

AtCPK2 ---------- ---------- ---------- ---------- ---------- ---------- -----SLLKT IDFGLSMFFK P--------- ----------

AtCPK1 ---------- ---------- ---------- ---------- ---------- ---------- -----SLLKT IDFGLSMFFK P--------- ----------

AtCPK12 ---------- ---------- ---------- ---------- ---------- ---------- -----ASLKS TDFGLSVFCT P--------- ----------

AtCPK11 ---------- ---------- ---------- ---------- ---------- ---------- -----AKLKA TDFGLSVFYK P--------- ----------

AtCPK4 ---------- ---------- ---------- ---------- ---------- ---------- -----AKLKA TDFGLSVFYK P--------- ----------

AtCIPK7 ---------- ---------- ---------- ---------- ---------- ---------- -----GNLKV SDFGLSALPE H--------- ----------

AtCIPK4 ---------- ---------- ---------- ---------- ---------- ---------- -----GNLKV SDFGLSALPE H--------- ----------

AtCIPK22 ---------- ---------- ---------- ---------- ---------- ---------- -----RDLKV SDFGLSAMKE Q--------- ----------

AtCIPK11 ---------- ---------- ---------- ---------- ---------- ---------- -----GNLKV SDFGLSALTD Q--------- ----------

AtCIPK14 ---------- ---------- ---------- ---------- ---------- ---------- -----LDLKI SDFGLSALTD Q--------- ----------

AtCIPK12 ---------- ---------- ---------- ---------- ---------- ---------- -----GNLKV SDFGLSAVSD Q--------- ----------

AtCIPK19 ---------- ---------- ---------- ---------- ---------- ---------- -----GNLKV SDFGLSAVSD Q--------- ----------

AtCIPK18 ---------- ---------- ---------- ---------- ---------- ---------- -----GNLKV SDFGLSAVAE Q--------- ----------

AtCIPK13 ---------- ---------- ---------- ---------- ---------- ---------- -----GNVKV SDFGLSVVSE Q--------- ----------

AtCIPK16 ---------- ---------- ---------- ---------- ---------- ---------- -----GDLKV TDFGLSALMM PEGLGGRR-- ----------

AtCIPK5 ---------- ---------- ---------- ---------- ---------- ---------- -----GDLKI SDFGLSALPE Q--------- ----------

AtCIPK25 ---------- ---------- ---------- ---------- ---------- ---------- -----GDLKV SDFGLSALPE Q--------- ----------

AtCIPK2 ---------- ---------- ---------- ---------- ---------- ---------- -----DNLKV SDFGLSALAD C--------- ----------

AtCIPK10 ---------- ---------- ---------- ---------- ---------- ---------- -----ENLKV SDFGLSALAD C--------- ----------

AtCIPK15 ---------- ---------- ---------- ---------- ---------- ---------- -----GNLKI SDFGLSALSD S--------- ----------

AtCIPK6 ---------- ---------- ---------- ---------- ---------- ---------- -----GNLKV TDFGLSAFTE H--------- ----------

AtCIPK20 ---------- ---------- ---------- ---------- ---------- ---------- -----GDLKI SDFGLSALRE S--------- ----------

AtCIPK21 ---------- ---------- ---------- ---------- ---------- ---------- -----GNLKV SDFGLSAVPK S--------- ----------

AtCIPK17 ---------- ---------- ---------- ---------- ---------- ---------- -----GHIKI TDFGLSALSQ H--------- ----------

AtCIPK1 ---------- ---------- ---------- ---------- ---------- ---------- -----GHIKI TDFGLSALPQ H--------- ----------

AtCIPK3 ---------- ---------- ---------- ---------- ---------- ---------- -----GNLKI SDFGLSALSQ Q--------- ----------

AtCIPK9 ---------- ---------- ---------- ---------- ---------- ---------- -----GVLKV SDFGLSAFSR Q--------- ----------

AtCIPK23 ---------- ---------- ---------- ---------- ---------- ---------- -----GALKV SDFGLSALPQ Q--------- ----------

AtCIPK8 ---------- ---------- ---------- ---------- ---------- ---------- -----GNLKI SDFGLSALPE Q--------- ----------

AtCIPK24 ---------- ---------- ---------- ---------- ---------- ---------- -----GNLKV SDFGLSALPQ E--------- ----------

CrMAPK7 ---------- ---------- ---------- ---------- ---------- ---------- -----DSIKI ADFGLAREIR SR-------- ----------

CrMAPK9 ---------- ---------- ---------- ---------- ---------- ---------- AAGGVGKLKL CDFGFARQLP PA-------- ----------

CrMAPK13 ---------- ---------- ---------- ---------- AVHPGGQSCS --------ST PHGAGMVVKL CDFGLARYLP SKRSTTAAAR PRR-------

Cre03.g206202 ---------- ---------- ---------- ---------- ---------- ---------- ----EGFVKL CDFGLARALP RAGAPTSSAR DSG-------

CrMAPK14 ---------- ---------- ---------- ---------- ---------- ---------- ----SGVLKI CDFGFARAMT SS-------- ----------

CrMAPK17 ---------- ---------- ---------- ---------- ---------- ---------- ----AGVLRL CDFGFARFTC EEAAIAAKA- ----------

CrMAPK16 ---------- ---------- ---------- ---------- ---------- ---------- ----EGVLKL CDFGFARFTS CDD------- ----------

CrMAPK15 ---------- ---------- ---------- ---------- ---------- ---------- ----TLTAKL CDFGFAKATN CD-------- ----------

CrMAPK12 ---------- ---------- ---------- ---------- ---------- ---------- ----TGVVRL CDFGFARPCN SS-------- ----------

CrMAPK10 ---------- ---------- ---------- ---------- ---------- ---------- ----SGVAKL CDFGFARAVR CG-------- ----------

CrMAPK11 ---------- ---------- ---------- ---------- ---------- ---------- ----SGVAKL CDFGFARAVR CG-------- ----------

CrGSK3 ---------- ---------- ---------- ---------- ---------- ---------- ----THQLKL CDFGSAKVLV KG-------- ----------

CrCDKI1 ---------- ---------- ---------- ---------- ---------- ---------- ----SGTAKL ADCGLARPLD GG-------- ----------

CrMAPK5 ---------- ---------- ---------- ---------- ---------- ---------- ----DCMVKL ADFGLARSVS QLN------- ----------

CrMAPK8 ---------- ---------- ---------- ---------- ---------- ---------- ----NCDLKI CDFGLARTST SN-------- ----------

CrMAPK6 ---------- ---------- ---------- ---------- ---------- ---------- ----SCDLKI ADFGLARTGT -E-------- ----------

CrMAPK3 ---------- ---------- ---------- ---------- ---------- ---------- ----SCDLKI CDFGLARTGS -E-------- ----------

CrMAPK2 ---------- ---------- ---------- ---------- ---------- ---------- ----DCKLKI CDFGLARPAF NDM------- ----------

CrMAPK4 ---------- ---------- ---------- ---------- ---------- ---------- ----NCKLKI CDFGLARPHL LDG------- ----------

CrCDPK5 ---------- ---------- ---------- ---------- ---------- --------CE AAGAPLRVKV IDLGMSAQFD PKK------- ----------

CrCDPK4 ---------- ---------- ---------- ---------- ---------- ---------- -----APVKA IDFGLAVFFD PKK------- ----------

CrCDPK3 ---------- ---------- ---------- ---------- ---------- ---------- -----ARIKL SDFGLSTFFR EGQ------- ----------

PpCCaMK ---------- ---------- ---------- ---------- ---------- ---------- -----APLKI MDFGLSYIHH -NT------- ----------

NtCCaMK ---------- ---------- ---------- ---------- ---------- ---------- -----SPLKI MDFGLSSIED -FA------- ----------

LjCCaMK ---------- ---------- ---------- ---------- ---------- ---------- -----SPLKI MDFGLSSVEE -FT------- ----------

MtCCaMK ---------- ---------- ---------- ---------- ---------- ---------- -----SPLKI MDFGLSSVEE -FT------- ----------

PsCCaMK ---------- ---------- ---------- ---------- ---------- ---------- -----SSLKI MDFGLSSVEE -FT------- ----------

LlCCaMK ---------- ---------- ---------- ---------- ---------- ---------- -----STLKI MDFGLSSVED -FT------- ----------

OsCCaMK ---------- ---------- ---------- ---------- ---------- ---------- -----STLKI MDFGLSSVED -FS------- ----------

Cre03.g199050 ---------- ---------- ---------- ---------- ---------- ---------- -------VKV TDFGFVKRLR KGT------- ----------

CrCDPKK1 ---------- ---------- ---------- ---------- ---------- ---------- -------VKL SDFGCSKVFA TGN------- ----------

CrCDPKK2 ---------- ---------- ---------- ---------- ---------- ---------- -------VKI SDFGSARFCE -KS------- ----------

AtMKK10 ---------- ---------- ---------- ---------- ---------- ---------- -------VKI ADFGASRIVA GGD------- ----------

AtMKK4 ---------- ---------- ---------- ---------- ---------- ---------- -------VKI ADFGVSRILA QTM------- ----------

AtMKK5 ---------- ---------- ---------- ---------- ---------- ---------- -------VKI ADFGVSRILA QTM------- ----------

AtMKK8 ---------- ---------- ---------- ---------- ---------- ---------- -------VKI ADFGVSKIVV RSL------- ----------

AtMKK7 ---------- ---------- ---------- ---------- ---------- ---------- -------VKI ADFGVSKIIT RSL------- ----------

AtMKK9 ---------- ---------- ---------- ---------- ---------- ---------- -------VKI ADFGVSKILV RSL------- ----------

CrMAPKK1 ---------- ---------- ---------- ---------- ---------- ---------- -------PKI TDFGISAFID STL------- ----------

AtMKK3 ---------- ---------- ---------- ---------- ---------- ---------- -------PKI TDFGISAGLE NSM------- ----------

CrMAPKK-L ---------- ---------- ---------- ---------- ---------- ---------- -------VKI SDFGVSGQLA SSV------- ----------

AtMKK6 ---------- ---------- ---------- ---------- ---------- ---------- -------VKI SDFGVSASLA SSM------- ----------

AtMEK1 ---------- ---------- ---------- ---------- ---------- ---------- -------VKI TDFGVSKILT STS------- ----------

AtMKK2 ---------- ---------- ---------- ---------- ---------- ---------- -------VKI TDFGVSTVMT NTA------- ----------

AtMAPKKK13 ---------- ---------- ---------- ---------- ---------- ---------- -------VKL ADFGSAFRIH TPR------- ----------

AtMAPKKK14 ---------- ---------- ---------- ---------- ---------- ---------- -------VKL ADFGSAVEFE KST------- ----------

Cre10.g464100 ---------- ---------- ---------- ---------- ---------- ---------- -------VKL ADFGASKKIE DLA------- ----------

AtANP2 ---------- ---------- ---------- ---------- ---------- ---------- -------IKL ADFGASKQVA ELA------- ----------

AtANP1 ---------- ---------- ---------- ---------- ---------- ---------- -------IKL ADFGASKQVA ELA------- ----------

AtANP3 ---------- ---------- ---------- ---------- ---------- ---------- -------IRL ADFGASKKVV ELA------- ----------

Cre03.g169100 ---------- ---------- ---------- ---------- ---------- ---------- -------VKL GDFGLAIDWS HE-------- ----------

Cre17.g735550 ---------- ---------- ---------- ---------- ---------- ---------- -------VKL ADFGLAINMV EE-------- ----------

CrALK3 ---------- ---------- ---------- ---------- ---------- ---------- -------LKL GDFGLAIDLR EE-------- ----------

CrALK1 ---------- ---------- ---------- ---------- ---------- ---------- -------LKV ADFGLSINFS QE-------- ----------

CrMAPKKK3 ---------- ---------- ---------- ---------- ---------- ---------- -------VKV CDFGLSKVKM DTF------- ----------

AtEDR1 ---------- ---------- ---------- ---------- ---------- ---------- -------VKV GDFGLSRLKH NTF------- ----------

AtCTR1 ---------- ---------- ---------- ---------- ---------- ---------- -------VKV CDFGLSRLKA STF------- ----------

CrCNK9 ---------- ---------- ---------- ---------- ---------- ---------- -------LKI ADFGLSTVLH DDG------- ----------

AtNEK7 ---------- ---------- ---------- ---------- ---------- ---------- -------VQL GNYGLAKLIN PEK------- ----------

AtNEK5 ---------- ---------- ---------- ---------- ---------- ---------- -------VRL GDFGLAKLLG KDD------- ----------

AtNEK6 ---------- ---------- ---------- ---------- ---------- ---------- -------VRL GDFGLAKTLK ADD------- ----------

AtNEK4 ---------- ---------- ---------- ---------- ---------- ---------- -------IRL GDFGLAKILT SDD------- ----------

AtNEK1 ---------- ---------- ---------- ---------- ---------- ---------- -------IRL GDFGLAKILT SDD------- ----------

AtNEK2 ---------- ---------- ---------- ---------- ---------- ---------- -------IRL GDFGLAKVLT SDD------- ----------

AtNEK3 ---------- ---------- ---------- ---------- ---------- ---------- -------IRL GDFGLAKILT SDD------- ----------

CrCNK7 ---------- ---------- ---------- ---------- ---------- ---------- -------LKL GDLGLSRYFS SRT------- ----------

CrCNK6 ---------- ---------- ---------- ---------- ---------- ---------- -------LKL GDLGLGRQLS EQT------- ----------

CrCNK4 ---------- ---------- ---------- ---------- ---------- ---------- -------VKL GDMGVAKILS TNT------- ----------

CrFA2 ---------- ---------- ---------- ---------- ---------- ---------- -------IKI GDFGIARSLG ASS------- ----------

CrCNK1 ---------- ---------- ---------- ---------- ---------- ---------- -------LKI GDLGIAKALT SM-------- ----------

CrCNK2 ---------- ---------- ---------- ---------- ---------- ---------- -------AKI GDLGIAKLLT KT-------- ----------

CrCNK8 ---------- ---------- ---------- ---------- ---------- ---------- -------VLL GDFGLAKQLQ RTF------- ----------

CrCNK5 ---------- ---------- ---------- ---------- ---------- ---------- -------IKL GDFGISKVLE RTD------- ----------

CrFAP403 ---------- ---------- ---------- ---------- ---------- ---------- -------LKL GDFGVSKVLN STF------- ----------

CrCNK3 ---------- ---------- ---------- ---------- ---------- ---------- -------LKL GDFGIARVLN SDT------- ----------

CrCDPKK3 ---------- ---------- ---------- ---------- ---------- ---------- --------RI LDFGSAVFHA RVM------- ----------

CrMAPK1 ---------- ---------- ---------- ---------- ---------- ---------- ---------- ---------- ---------- ----------

Cre08.g379600 P--SRPVMLR AKQSA----- ---------- ---------- ---------- ---------V ALPSEEAGTP EVAGLAAAAG HGGECQCD-- ----------

Cre02.g108601 A--ERSVMLR RRASS----- ---------- ---------- ---------- ---------L GTRSIHSRLS ---------R RNSTAQPE-- ----------

Cre02.g108750a M--DRKVMLR RRSVT----- ---------- ---------- ---------- ---------A GSSMSGTAAP TSGG-----G NDSGVLPS-- ----------

Cre17.g713750 P--ADP---T GTFCF----- ---------- ---------E IA-------- ---------- ---------- ---------- ---------- ----------

Cre11.g467586 P--ADP---T GTFVF----- ---------- ---------E MA-------- ---------- ---------- ---------- ---------- ----------

Cre11.g467588 P--ADP---T GTFFF----- ---------- ---------E MA-------- ---------- ---------- ---------- ---------- ----------

Cre11.g467589 P--ADP---T GTFFF----- ---------- ---------E MA-------- ---------- ---------- ---------- ---------- ----------

Cre11.g467584 P--ADP---T GTFCF----- ---------- ---------E IA-------- ---------- ---------- ---------- ---------- ----------

Cre11.g467585 P--ADP---T GTFVF----- ---------- ---------E MA-------- ---------- ---------- ---------- ---------- ----------

Cre12.g489850 P--D------ GEYVG----- ---------- ---------D KC-------- ---------- ---------- ---------- ---------- ----------

Cre12.g490200 K--NRKIL-A DTYHK----- ---------- ---------D DC-------- ---------- ---------- ---------- ---------- ----------

Cre08.g384250 ---------- ---------- ---------- ---------- ---------- ---------- ---------- -DFSFAKDAD KDEDS----- -NP-------

Cre04.g217940 ---------- ---------- ---------- ---------- ---------- ---------- ---------- -DFGLVTPLD SQGRLVSE-- ------V---

Cre04.g217923 ---------- ---------- ---------- ---------- ---------- ---------- ---------- -DFGLVTPLD SQGRLVSE-- ------V---

Cre04.g217928 ---------- ---------- ---------- ---------- ---------- ---------- ---------- -DFGLVTPLD SQGRLVSE-- ------V---

Cre08.g369700 ---------- ---------- ---------- ---------- ---------- ---------- ---------- -DFGLARRIT LPSDFSAGKA KSPLTRVDGG

Cre08.g369900 ---------- ---------- ---------- ---------- ---------- ---------- ---------- -DFGLARRIT LPSDLAGE-- AKSLTGVDGG

Cre03.g187300 A--APHAHTD GLPCY----- ---------- ---------H HVCPAPPPCP TGLMMAAASA GARAAAA-SA AACALTADHG GRGRVAAGAV LERLRSWAAA

Cre01.g041752 P--TPSIGGS SSTQF----- ---------- ---------- LVT------- ---------- ---------- -SCGVAAPVD AAGVVEQRRM RVK-------

Cre12.g495401 P--TPSIGGS SSTQF----- ---------- ---------- LVT------- ---------- ---------- -SCGLAAPVD AAGVVEQRRM RVK-------

Cre06.g266450 --------SD GAIHF----- ---------- ---------- VLG------- ---------- ---------- -DMGTAEPTD SEGMLIRG-- -AI-------

Cre06.g266000 --------AD GVIHF----- ---------- ---------- VLG------- ---------- ---------- -DFGSAEPTD SEGMLISG-- -AV-------

Cre06.g266250 --------AD GVIHF----- ---------- ---------- VLG------- ---------- ---------- -DFGSAEPTD SEGMLISG-- -AV-------

Cre10.g460700 --------PD GSKVF----- ---------- ---------- RVC------- ---------- ---------- -DMGIASKAD KSGRLLQ--- -CP-------

Cre12.g545750 --------PD GSKVF----- ---------- ---------- RVC------- ---------- ---------- -DFNLAVKTD SNGCVER--- -AP-------

Cre12.g525950 --------PD GSKVF----- ---------- ---------- RVC------- ---------- ---------- -DFNLAVKTD SNGRVAR--- -AQ-------

Cre12.g525750 --------PD GSKVF----- ---------- ---------- RVC------- ---------- ---------- -DFNLAVKTD SNGRVAR--- -AQ-------

Cre12.g526650 --------PD GSKVF----- ---------- ---------- RVC------- ---------- ---------- -DFNLAVKTD SNGRVAR--- -AQ-------

Cre12.g526051 --------PD GSKVF----- ---------- ---------- RVC------- ---------- ---------- -DFNLAVKTD SNGRVAR--- -AQ-------

Cre12.g526250 --------PD GSKVF----- ---------- ---------- RVC------- ---------- ---------- -DFNLAVKTD SNGRVAR--- -AQ-------

Cre01.g054750 --------PD GSKVF----- ---------- ---------- RVC------- ---------- ---------- -DFNLATKLD SNGHVER--- -AP-------

Cre01.g055457 --------PD GSKVF----- ---------- ---------- RVC------- ---------- ---------- -DFNFATKLD SNGHVGS--- -AP-------

Cre09.g404550 ---------- ---------- ---------- ---------- ---------- ---------- ---------- ---------- ---------- ----------

Cre02.g108750b LGT------- ---------- MLARRP---- ---------S LVTPVDSSAA RSG------- L--RAVRSLL QQLGKREL-R PLRKGD---- ----------

Cre02.g095099 LPTAR----- D--------- ---------- ---------- ---------- ---------- ------TARS CHTAL----- ---------- ----------

Cre17.g710600 P--------- AASSPAASGA LFGGAGASSI ---------- ---------N GDG------G IDAFRYTAKV GDFGLSRALD SGRSHL---- ----------

CrMAPKKK14 ---------- ---------- ---------- ---------- ---------- ---------- -DPRGFSAKL ADYGFALPLT KTAEDGTR-- ----------

CrMAPKKK4 ---------- ---------- ---------- ---------- ---------- ---------- -DYRGFTVKL ADFGFVLHLS EVAEDGTR-- ----------

CrMAPKKK8 ---------- ---------- ---------- ---------- ---------- ---------- ------RAKV CDFGIAKIKD RT-------- ----------

CrMAPKKK1 ---------- ---------- ---------- ---------- ---------- ---------- ---MRPIAKL TDFGLSRLRS TV-------- ----------

CrMAPKKK12 ---------- ---------- ---------- ---------- ---------- ---------- ---DTPEVKL TDFGQAHIRM STTFGSSR-- ----------

Cre09.g390300 ---------- ---------- ---------- ---------- ---------- ---------- ----SPTCKV ADFGLSKQRQ QTYV------ ----------

CrMAPKKK11 ---------- ---------- ---------- ---------- ---------- ---------- -DRRGFTVKV ADFGLSKLLH TDNS------ ----------

CrMAPKKK13 ---------- ---------- ---------- ---------- ---------- ---------- -DRRGFVAKV SDFGLSRVLA GNRK------ ----------

CrPTK7 APASVPRTVS DTSDRAAAGS GAASGPQPRV GGSGGQQQQQ AQQPGPTSS- TFE------R LLTSGIVAKV GDFGLATHMD DERQET---- ----------

Cre02.g117813 ---------- -----GLAGA AFTSGP---- --QASAAAAA LAGPSSASLL GGG------Q RDRVPFVAKV ADFGLSKTFS ETST------ ----------

CrMAPKKK10 N--------- GRLNSSVLGS AFGGGG---- --AAAAAAKT MASPSPLGDP -SA------- --QLHVTAKI ADFGLAAPPV PAAAAA---- ----------

CrMAPKKK2 ---------- ---------- -CRGGC---- --D------- -LADAPLRSP -LA------G LWAPPLVAKV ADFGLSVHMG EHQT------ ----------

CrMAPKKK6 ---------- ---------- ---------- ---------- ---------- ---------- ---YGAVCKI ADFGLSIKMN ADQS------ ----------

CrMAPKKK5 ---------- ---------- ---------- ---------- ---------- ---------- ---SGYGVKV GDFGLSVMLP HDRT------ ----------

CrMAPKKK9 ---------- ---------- ---------- ---------- ---------- ---------- ---SGYAVKI GDFGLSVMLP QGRT------ ----------

Cre12.g516650 ---------- ---------- ---------- ---------- ---------- ---------- ---LGFCLKV SDFGLSVRVG EGQS------ ----------

CrMAPKKK7 ---------- ---------- ---------- ---------- ---------- ---------- ---LGFCLKV ADFGLSIRMA EDES------ ----------

....|....| ....|....| ....|....| ....|....| ....|....| ....|....| ....|....| ....|....| ....|....| ....|....|

1010 1020 1030 1040 1050 1060 1070 1080 1090 1100

CrCDPK9 ---------- ---------- ---------- ---------- ---------- GERFRDLVGS PYYVAPEVLR K--------- ---------- ----------

AtCPK25 ---------- ---------- ---------- ---------- ---------- GENFTDVVGS PYYIAPEVLN K--------- ---------- ----------

AtCPK26 ---------- ---------- ---------- ---------- ---------- GQIFEDVVGS PYYVAPEVLL K--------- ---------- ----------

AtCPK6 ---------- ---------- ---------- ---------- ---------- GQIFKDVVGS PYYVAPEVLL K--------- ---------- ----------

AtCPK5 ---------- ---------- ---------- ---------- ---------- GQIFTDVVGS PYYVAPEVLL K--------- ---------- ----------

AtCPK20 ---------- ---------- ---------- ---------- ---------- GETFTDVVGS PYYVAPEVLR K--------- ---------- ----------

AtCPK2 ---------- ---------- ---------- ---------- ---------- DEVFTDVVGS PYYVAPEVLR K--------- ---------- ----------

AtCPK1 ---------- ---------- ---------- ---------- ---------- DDVFTDVVGS PYYVAPEVLR K--------- ---------- ----------

AtCPK12 ---------- ---------- ---------- ---------- ---------- GEAFSELVGS AYYVAPEVLH K--------- ---------- ----------

AtCPK11 ---------- ---------- ---------- ---------- ---------- GQYLYDVVGS PYYVAPEVLK K--------- ---------- ----------

AtCPK4 ---------- ---------- ---------- ---------- ---------- GQYLYDVVGS PYYVAPEVLK K--------- ---------- ----------

AtCIPK7 ---------- ---------- ---------- ---------- -------L-Q NGLLHTACGT PAYTAPEVIS R--------- ---------- ----------

AtCIPK4 ---------- ---------- ---------- ---------- -------RSN NGLLHTACGT PAYTAPEVIA Q--------- ---------- ----------

AtCIPK22 ---------- ---------- ---------- ---------- -------IHP DGMLHTLCGT PAYVAPELLL K--------- ---------- ----------

AtCIPK11 ---------- ---------- ---------- ---------- -------IRP DGLLHTLCGT PAYVAPEILS K--------- ---------- ----------

AtCIPK14 ---------- ---------- ---------- ---------- -------IRP DGLLHTLCGT PAYVAPEVLA K--------- ---------- ----------

AtCIPK12 ---------- ---------- ---------- ---------- -------IRQ DGLFHTFCGT PAYVAPEVLA R--------- ---------- ----------

AtCIPK19 ---------- ---------- ---------- ---------- -------IRQ DGLFHTFCGT PAYVAPEVLA R--------- ---------- ----------

AtCIPK18 ---------- ---------- ---------- ---------- -------LRQ DGLCHTFCGT PAYIAPEVLT R--------- ---------- ----------

AtCIPK13 ---------- ---------- ---------- ---------- -------LKQ EGICQTFCGT PAYLAPEVLT R--------- ---------- ----------

AtCIPK16 ---------- ---------- ---------- ---------- -------GSS DDLLHTRCGT PAYVAPEVLR N--------- ---------- ----------

AtCIPK5 ---------- ---------- ---------- ---------- -------ILQ DGLLHTQCGT PAYVAPEVLK K--------- ---------- ----------

AtCIPK25 ---------- ---------- ---------- ---------- -------ILQ DGLLHTQCGT PAYVAPEVLR K--------- ---------- ----------

AtCIPK2 ---------- ---------- ---------- ---------- -------KRQ DGLLHTTCGT PAYVAPEVIN R--------- ---------- ----------

AtCIPK10 ---------- ---------- ---------- ---------- -------KRQ DGLLHTTCGT PAYVAPEVIN R--------- ---------- ----------

AtCIPK15 ---------- ---------- ---------- ---------- -------RRQ DGLLHTTCGT PAYVAPEVIS R--------- ---------- ----------

AtCIPK6 ---------- ---------- ---------- ---------- -------LKQ DGLLHTTCGT PAYVAPEVIL K--------- ---------- ----------

AtCIPK20 ---------- ---------- ---------- ---------- -------KQQ DGLLHTTCGT PAYVAPEVIG K--------- ---------- ----------

AtCIPK21 ---------- ---------- ---------- ---------- -------G-- -DMLSTACGS PCYIAPELIM N--------- ---------- ----------

AtCIPK17 ---------- ---------- ---------- ---------- -------YRE DGLLHTTCGS PNYVAPEVLA N--------- ---------- ----------

AtCIPK1 ---------- ---------- ---------- ---------- -------FRD DGLLHTTCGS PNYVAPEVLA N--------- ---------- ----------

AtCIPK3 ---------- ---------- ---------- ---------- -------VRD DGLLHTSCGT PNYVAPEVLN D--------- ---------- ----------

AtCIPK9 ---------- ---------- ---------- ---------- -------VRE DGLLHTACGT PNYVAPEVLS D--------- ---------- ----------

AtCIPK23 ---------- ---------- ---------- ---------- -------VRE DGLLHTTCGT PNYVAPEVIN N--------- ---------- ----------

AtCIPK8 ---------- ---------- ---------- ---------- -------G-- VTILKTTCGT PNYVAPEVLS H--------- ---------- ----------

AtCIPK24 ---------- ---------- ---------- ---------- -------G-- VELLRTTCGT PNYVAPEVLS G--------- ---------- ----------

CrMAPK7 ---------- ---------- ---------- ---------- ---------- -PPYTDYVST RWYRAPEVLL R--------- ---------- ----------

CrMAPK9 ---------- ---------- ---------- ---------- ---------- DVSITDYVST RWYRAPELLL G--------- ---------- ----------

CrMAPK13 GGE------- ---------- ---------- ---------A GPLDDR-KQR LGGLTDYVVT RWYRAPEVII S--------- ---------- ----------

Cre03.g206202 SSPEASS--A NSCSQSCSDS R--------- ------PYVA GSAGAQ-QSQ QQLLSSYVVT RWYRPPEILL S--------- ---------- ----------

CrMAPK14 ---------- ---------- ---------- ---------- --------SA DPAYTTYVVT RWYRAPEVLV G--------- ---------- ----------

CrMAPK17 ---------- ---------- ---------- ---------- --DLRA-AAS SDRLTPYVVT RWYRAPEVLL G--------- ---------- ----------

CrMAPK16 ---------- ---------- ---------- ---------- -------PRD GEALTSYVVT RWYRAPEVLL G--------- ---------- ----------

CrMAPK15 ---------- ---------- ---------- ---------- -------PRE ARRGTSYVVT RWYRAPEILV R--------- ---------- ----------

CrMAPK12 ---------- ---------- ---------- ---------- -------AKE AEQCTSYVVT RWYRAPEVLV A--------- ---------- ----------

CrMAPK10 ---------- ---------- ---------- ---------- -------PRE AQRCTSYVVT RWYRAPEVLV S--------- ---------- ----------

CrMAPK11 ---------- ---------- ---------- ---------- -------PRE AQRCTSYVVT RWYRAPEVLV S--------- ---------- ----------

CrGSK3 ---------- ---------- ---------- ---------- ---------- -EPNISYICS RYYRAPELIF G--------- ---------- ----------

CrCDKI1 ---------- ---------- ---------- ---------- ---------E RPAYTHAVAT RWYRAPELLY G--------- ---------- ----------

CrMAPK5 ---------- ---------- ---------- ---------- ----AS-DGQ NPILTDYVAT RWYRAPEILL G--------- ---------- ----------

CrMAPK8 ---------- ---------- ---------- ---------- ---------E KEFMTEYVVT RWYRAPELLL S--------- ---------- ----------

CrMAPK6 ---------- ---------- ---------- ---------- ---------K QNFMTEYVVT RWYRAPELLL S--------- ---------- ----------

CrMAPK3 ---------- ---------- ---------- ---------- ---------- RNFMTEYVVT RWYRAPELLL S--------- ---------- ----------

CrMAPK2 ---------- ---------- ---------- ---------- --------PT TVFWTDYVAT RWYRAPELCG S--------- ---------- ----------

CrMAPK4 ---------- ---------- ---------- ---------- --AGAA-PLT PVLWTDYVAT RWYRAPELCG C--------- ---------- ----------

CrCDPK5 ---------- ---------- ---------- ---------- --------L- V---REATGT PGFAAPELFN G--------- ---------- ----------

CrCDPK4 ---------- ---------- ---------- ---------- --------L- PRSDLGLEGT PHFMAPEQLN S--------- ---------- ----------

CrCDPK3 ---------- ---------- ---------- ---------- ---------- -ADFADVLGS AYYMAPEVLE R--------- ---------- ----------

PpCCaMK ---------- ---------- ---------- ---------- ---------- -NSIVGIFGS IDYMAPEQLS L--------- ---------- ----------

NtCCaMK ---------- ---------- ---------- ---------- ---------- -NPVVGLFGS IDYVSPEALS R--------- ---------- ----------

LjCCaMK ---------- ---------- ---------- ---------- ---------- -DPVVGLFGS IDYVSPEALS Q--------- ---------- ----------

MtCCaMK ---------- ---------- ---------- ---------- ---------- -DPVVGLFGS IDYVSPEALS Q--------- ---------- ----------

PsCCaMK ---------- ---------- ---------- ---------- ---------- -DPVVGLFGS IDYVSPEALS Q--------- ---------- ----------

LlCCaMK ---------- ---------- ---------- ---------- ---------- -DPIVALFGS IDYVSPEALS Q--------- ---------- ----------

OsCCaMK ---------- ---------- ---------- ---------- ---------- -DPIVALFGS IDYVSPEALS R--------- ---------- ----------

Cre03.g199050 ---------- ---------- ---------- ---------- --------K- ---TYTLCGT PEYLAPEIIM N--------- ---------- ----------

CrCDPKK1 ---------- ---------- ---------- ---------- --------E- --YLERCNGT PAFLAPEMMK P--------- ---------- ----------

CrCDPKK2 ---------- ---------- ---------- ---------- --------D- --MIFATAGT PTFMAPEMCQ ---------- ---------- ----------

AtMKK10 ---------- ---------- ---------- ---------- ---------- ----YGSNGT CAYMSPERVD L--------- ---------- ----------

AtMKK4 ---------- ---------- ---------- ---------- --------D- --PCNSSVGT IAYMSPERIN T--------- ---------- ----------

AtMKK5 ---------- ---------- ---------- ---------- --------D- --PCNSSVGT IAYMSPERIN T--------- ---------- ----------

AtMKK8 ---------- ---------- ---------- ---------- --------N- --KCNSFVGT FAYMSPERLD S--------- ---------- ----------

AtMKK7 ---------- ---------- ---------- ---------- --------D- --YCNSYVGT CAYMSPERFD S--------- ---------- ----------

AtMKK9 ---------- ---------- ---------- ---------- --------D- --SCNSYVGT CAYMSPERFD S--------- ---------- ----------

CrMAPKK1 ---------- ---------- ---------- ---------- --------A- --QCNTFLGT VTYMSPERIN N--------- ---------- ----------

AtMKK3 ---------- ---------- ---------- ---------- --------A- --MCATFVGT VTYMSPERIR N--------- ---------- ----------

CrMAPKK-L ---------- ---------- ---------- ---------- --------S- --NCLSWVGT VTYMSPERIK G--------- ---------- ----------

AtMKK6 ---------- ---------- ---------- ---------- --------G- --QRDTFVGT YNYMSPERIS G--------- ---------- ----------

AtMEK1 ---------- ---------- ---------- ---------- --------S- --LANSFVGT YPYMSPERIS G--------- ---------- ----------

AtMKK2 ---------- ---------- ---------- ---------- --------G- --LANTFVGT YNYMSPERIV G--------- ---------- ----------

AtMAPKKK13 ---------- ---------- ---------- ---------- --------A- ---LITPRGS PLWMAPEVIR R--------- ---------- ----------

AtMAPKKK14 ---------- ---------- ---------- ---------- --------I- ---HVSPRGS PLWMAPEVVR R--------- ---------- ----------

Cre10.g464100 ---------- ---------- ---------- ---------- --------TI GGGSRSIRGT ANWMAPEVIK Q--------- ---------- ----------

AtANP2 ---------- ---------- ---------- ---------- --------TI SG-AKSMKGT PYWMAPEVIL Q--------- ---------- ----------

AtANP1 ---------- ---------- ---------- ---------- --------TM TG-AKSMKGT PYWMAPEVIL Q--------- ---------- ----------

AtANP3 ---------- ---------- ---------- ---------- --------TV NG-AKSMKGT PYWMAPEVIL Q--------- ---------- ----------

Cre03.g169100 ---------- ---------- ---------- ---------- ---------- --LAFSRSGT LDYMAPEVLI NPATHLQESA A--------- V---------

Cre17.g735550 ---------- ---------- ---------- ---------- ---------- --RPVSRVGT LDYMAPEVLR CPIKQHMGMA P--------- G---------

CrALK3 ---------- ---------- ---------- ---------- ---------- --RAVTRAGT LDYMAPEVLR CPFKSRPEEN ---------- K---------

CrALK1 ---------- ---------- ---------- ---------- ---------- --RPVTRVGT LDYMAPEVVV CPDKHRPQDH ---------- K---------

CrMAPKKK3 ---------- ---------- ---------- ---------- ---------- -LTAKTQGGS PAWMAPEILR S--------- ---------- ----------

AtEDR1 ---------- ---------- ---------- ---------- ---------- -LSSKSTAGT PEWMAPEVLR N--------- ---------- ----------

AtCTR1 ---------- ---------- ---------- ---------- ---------- -LSSKSAAGT PEWMAPEVLR D--------- ---------- ----------

CrCNK9 ---------- ---------- ---------- ---------- --------AG HLLTKTVVGT PNYMSPCVLQ E--------- ---------- ----------

AtNEK7 ---------- ---------- ---------- ---------- ---------- --PVSMVSGI SNSMCPEVLE D--------- ---------- ----------

AtNEK5 ---------- ---------- ---------- ---------- ---------- --LASSMVGT PNYMCPELLA D--------- ---------- ----------

AtNEK6 ---------- ---------- ---------- ---------- ---------- --LTSSVVGT PNYMCPELLA D--------- ---------- ----------

AtNEK4 ---------- ---------- ---------- ---------- ---------- --LTSSVVGT PSYMCPELLA D--------- ---------- ----------

AtNEK1 ---------- ---------- ---------- ---------- ---------- --LTSSVVGT PSYMCPELLA D--------- ---------- ----------

AtNEK2 ---------- ---------- ---------- ---------- ---------- --LASSVVGT PSYMCPELLA D--------- ---------- ----------

AtNEK3 ---------- ---------- ---------- ---------- ---------- --LASSVVGT PSYMCPELLA D--------- ---------- ----------

CrCNK7 ---------- ---------- ---------- ---------- ---------- -LQANTTVGT PYYMSPEVVR G--------- ---------- ----------

CrCNK6 ---------- ---------- ---------- ---------- ---------- -MEAFSKVGT PYYVSPEVVR G--------- ---------- ----------

CrCNK4 ---------- ---------- ---------- ---------- ---------- -VFAKTIVGT PYYLSPELCE D--------- ---------- ----------

CrFA2 ---------- ---------- ---------- ---------- ---------- -NLAQTILGT PYYMAPELCQ D--------- ---------- ----------

CrCNK1 ---------- ---------- ---------- ---------- ---------- -NFARTQIGT PCYMAPEVWS G--------- ---------- ----------

CrCNK2 ---------- ---------- ---------- ---------- ---------- -AAAKTQIGT PHYMGPEIWK N--------- ---------- ----------

CrCNK8 ---------- ---------- ---------- ---------- ---------- -EMARTPIGT PYYMAPEIYE E--------- ---------- ----------

CrCNK5 ---------- ---------- ---------- ---------- ---------- -SFATTVTGT PYYMAPEICT N--------- ---------- ----------

CrFAP403 ---------- ---------- ---------- ---------- ---------- -QLATTAVGT PYYLSPEICQ N--------- ---------- ----------

CrCNK3 ---------- ---------- ---------- ---------- ---------- -ELARTVIGS PYYLSPEICE D--------- ---------- ----------

CrCDPKK3 ---------- ---------- ---------- ---------- GEEGRGAH-- ----VPMSCT PGFRSPESLV SG-------- ---------- ----------

CrMAPK1 ---------- ---------- ---------- ---------- ---------- ---------- ---------- ---------- ---------- ----------

Cre08.g379600 ---------- ----SSPDPH GGVGA----- ---------- GVQQRGERGS Q-----QHPS QPCQQPHHHN HHHHDRQSH- ------GSRP AS---VAFIG

Cre02.g108601 ---------- ----R--SAH GGGGG----- ---------- GGAG------ --------GG TPLLSPHPRL S--KCGSSA- ---------- ----------

Cre02.g108750a ---------- ----RGPSAN GGGGA----- ---------- GATG------ --------PS TP---PSALA SGVKGGPSA- -----SQLLP GAAGIITMIA

Cre17.g713750 ---------- ---------- ---------- ---------- ---------- -------CGT RHYMAPEVQ- ---------- ---------- ----------

Cre11.g467586 ---------- ---------- ---------- ---------- ---------- -------CGT RHYMAPEVQ- ---------- ---------- ----------

Cre11.g467588 ---------- ---------- ---------- ---------- ---------- -------CGT RHYMAPEVQ- ---------- ---------- ----------

Cre11.g467589 ---------- ---------- ---------- ---------- ---------- -------CGT RHYMAPEVQ- ---------- ---------- ----------

Cre11.g467584 ---------- ---------- ---------- ---------- ---------- -------CGT RHYMAPEVQ- ---------- ---------- ----------

Cre11.g467585 ---------- ---------- ---------- ---------- ---------- -------CGT RHYMAPEVQ- ---------- ---------- ----------

Cre12.g489850 ---------- ---------- ---------- ---------- ---------- -------GGT MHFGAPEVAM RNEYIGGSIP WPFVE----- DYR-------

Cre12.g490200 ---------- ---------- ---------- ---------- ---------- -------CGS PDFCAPEAAL ANRDLRPKTP WLFLK----- NCI-------

Cre08.g384250 ---------- ---------- ---------- ---------- ---------- ----MAQHGS ALFAAPEVI- ---------- -TASHA---- ----------

Cre04.g217940 ---------- ---------- ---------- ---------- ---------- ----GGRRGT KGYQAPETLA RRTFAANERD LPSWP----- ----------

Cre04.g217923 ---------- ---------- ---------- ---------- ---------- ----GGRRGT KGYQAPETLA RRTFAANERD LPSWP----- ----------

Cre04.g217928 ---------- ---------- ---------- ---------- ---------- ----GGRRGT KGYQAPETLA RRTFAANERE LPSWP----- ----------

Cre08.g369700 -------HH- ---------- ---------- LGDAADANIF ---------G LLVQPFIGGT DGYKAPELYE RATFVKFPS- --AWLQ---- ----------

Cre08.g369900 -------HH- ---------- ---------- LGNAARANIF ---------G LLVQPFIGGT DGYKAPEICD RTTFVKFPS- --AWLQ---- ----------

Cre03.g187300 AGDAAARHHF VPRTRACAAA AGSDAGAAGT IDDGAAAPAA GLVGRGPNSS ASVGAAEAAL MVFTAPELRK AAVAAASSAA TAVLSAGSLP HAE-----VA

Cre01.g041752 ---------- ---------- ---------- ---------- ---------- -------GSA LEYAAAEVRA HLAAAR---- ---------S GCD-------

Cre12.g495401 ---------- ---------- ---------- ---------- ---------- -------GSA LEYAAPEVRA HLAAAR---- ---------S GCD-------

Cre06.g266450 ---------- ---------- ---------- ---------- ---------- -------GGS WLTAAPEQRV HLAGGT---- ---------P ACG-------

Cre06.g266000 ---------- ---------- ---------- ---------- ---------- -------GGS RATAAPEQRV HLAGGT---- ---------P ACG-------

Cre06.g266250 ---------- ---------- ---------- ---------- ---------- -------GGS RATAAPEQRV HLAGGT---- ---------P ACG-------

Cre10.g460700 ---------- ---------- ---------- ---------- ---------- -------GGT PAFAAGEVLQ KIRLGK---- ---------S QYP-------

Cre12.g545750 ---------- ---------- ---------- ---------- ---------- -------GGS RGFWAWETYC QLMGLE---- ---------Q KHP-------

Cre12.g525950 ---------- ---------- ---------- ---------- ---------- -------GGT RGFWAWETYC QLEGLE---- ---------V KHP-------

Cre12.g525750 ---------- ---------- ---------- ---------- ---------- -------GGT RGFWAWETYC QLEGLE---- ---------V KHP-------

Cre12.g526650 ---------- ---------- ---------- ---------- ---------- -------GGT RGFWAWETYC QLEGLE---- ---------V KHP-------

Cre12.g526051 ---------- ---------- ---------- ---------- ---------- -------GGT RGFWAWETYC QLEGLE---- ---------V KHP-------

Cre12.g526250 ---------- ---------- ---------- ---------- ---------- -------GGT RGFWAWETYC QLEGLE---- ---------V KHP-------

Cre01.g054750 ---------- ---------- ---------- ---------- ---------- -------GGT PGFWAPEVYF QLQGDT---- ---------P KYP-------

Cre01.g055457 ---------- ---------- ---------- ---------- ---------- -------GGT RAFCAWEVYC QLQGDT---- ---------P KYP-------

Cre09.g404550 ---------- ---------- ---------- ---------- ---------- ---------- ---------- ---------- ---------- ----------

Cre02.g108750b ---------- ---------- ---------- ---------- -------LEW VYGLTGRAGS YMYMAPEVWN R--------- ---------- ----------

Cre02.g095099 ---------- ---------- ---------- ---------- ---------- ---------- ---------- ---------- ---------- ----------

Cre17.g710600 ---------- ---------- ---------- ---------- ---------- QRTAGCVAGS LSHLAPEALL R--------- ---------- ----------

CrMAPKKK14 ---------- ---------- ---------- ---------- -------Y-- -ALVDQACGT VTHLPPEALN KV-------- ---------- ----------

CrMAPKKK4 ---------- ---------- ---------- ---------- -------Y-- -VMVDQACGT VTHMAPELLP GK-------- ---------- ----------

CrMAPKKK8 ---------- ---------- ---------- ---------- ---------L LSTRNAHAGT PAYMAPEQFE GR-------- ---------- ----------

CrMAPKKK1 ---------- ---------- ---------- ---------- ---------- LVTRHPEAGT PAYMAPEAFD AS-------- ---------- ----------

CrMAPKKK12 ---------- ---------- ---------- ---------- -------ATL GALDAPAGGT AAFCAPECFD QAGG------ ---------- ----------

Cre09.g390300 ---------- ---------- ---------- ---------- ---------- -TGVTSLRGT LPWTAPEIIR TP-------- ---------- ----------

CrMAPKKK11 ---------- ---------- ---------- ---------- ---------- -RIDSSAGGT LAYMSPEAF- D--------- ---------- ----------

CrMAPKKK13 ---------- ---------- ---------- ---------- ---------- -EIKTQTFGT VTHMPPELLS K--------- ---------- ----------

CrPTK7 ---------- ---------- ---------- ---------- -------H-- -ISALTAQGT LSHMAPELLL H--------- ---------- ----------

Cre02.g117813 ---------- ---------- ---------- ---------- ---------- -HMSTATVGT VTHMCPVLLL T--------- ---------- ----------

CrMAPKKK10 ---------- ---------- ---------- ---------- -------MSE AAKRAAAAAI TSYTAPEVAA AASSSSSH-- ---------- ----------

CrMAPKKK2 ---------- ---------- ---------- ---------- ---------- -HASNKFQGT PAYTAPEVLS R--------- ---------- ----------

CrMAPKKK6 ---------- ---------- ---------- ---------- ---------- -HISNMRRGT PFYTCPQILA R--------- ---------- ----------

CrMAPKKK5 ---------- ---------- ---------- ---------- ---------- -HLSNIRMGT MFYICPAVAC K--------- ---------- ----------

CrMAPKKK9 ---------- ---------- ---------- ---------- ---------- -HVSNLRMGT MFYICPAVVC Q--------- ---------- ----------

Cre12.g516650 ---------- ---------- ---------- ---------- ---------- -HLSNLFQGT PYYCAPEVML S--------- ---------- ----------

CrMAPKKK7 ---------- ---------- ---------- ---------- ---------- -HLSNLFQGT PYYCAPEVML S--------- ---------- ----------

....|....| ....|....| ....|....| ....|....| ....|....| ....|....| ....|....| ....|....| ....|....| ....|....|

1110 1120 1130 1140 1150 1160 1170 1180 1190 1200

CrCDPK9 ---------- ---------- N--YSHEADM WSLGVILYIL LSGLP-PFWG DTEDQI---- ---FKMVLKG HID------- ---------- ----------

AtCPK25 ---------- ---------- N--YGPEADI WSAGVMIYVL LSGSA-PFWG ETEEEI---- ---FNEVLEG ELD------- ---------- ----------

AtCPK26 ---------- ---------- H--YGPEADV WTAGVILYIL VSGVP-PFWA ETQQGI---- ---FDAVLKG HID------- ---------- ----------

AtCPK6 ---------- ---------- H--YGPEADV WTAGVILYIL LSGVP-PFWA ETQQGI---- ---FDAVLKG YID------- ---------- ----------

AtCPK5 ---------- ---------- R--YGPEADV WTAGVILYIL LSGVP-PFWA ETQQGI---- ---FDAVLKG YID------- ---------- ----------

AtCPK20 ---------- ---------- H--YSHECDV WSAGVIIYIL LSGVP-PFWD ETEQGI---- ---FEQVLKG DLD------- ---------- ----------

AtCPK2 ---------- ---------- R--YGPESDV WSAGVIVYIL LSGVP-PFWA ETEQGI---- ---FEQVLHG DLD------- ---------- ----------

AtCPK1 ---------- ---------- R--YGPEADV WSAGVIVYIL LSGVP-PFWA ETEQGI---- ---FEQVLHG DLD------- ---------- ----------

AtCPK12 ---------- ---------- H--YGPECDV WSAGVILYIL LCGFP-PFWA ESEIGI---- ---FRKILQG KLE------- ---------- ----------

AtCPK11 ---------- ---------- C--YGPEIDV WSAGVILYIL LSGVP-PFWA ETESGI---- ---FRQILQG KLD------- ---------- ----------

AtCPK4 ---------- ---------- C--YGPEIDV WSAGVILYIL LSGVP-PFWA ETESGI---- ---FRQILQG KID------- ---------- ----------

AtCIPK7 ---------- ---------- RGYDGAKADA WSCGVILFVL LVGDV-PFDD SNIAAM---- ---YRKIHRR DY-------- ---------- ----------

AtCIPK4 ---------- ---------- RGYDGAKADA WSCGVFLFVL LAGYV-PFDD ANIVAM---- ---YRKIHKR DY-------- ---------- ----------

AtCIPK22 ---------- ---------- KGYDGSKADI WSCGVVLFLL NAGYL-PFRD PNIMGL---- ---YRKIHKA QY-------- ---------- ----------

AtCIPK11 ---------- ---------- KGYEGAKVDV WSCGIVLFVL VAGYL-PFND PNVMNM---- ---YKKIYKG EY-------- ---------- ----------

AtCIPK14 ---------- ---------- KGYDGAKIDI WSCGIILFVL NAGYL-PFND HNLMVM---- ---YRKIYKG EF-------- ---------- ----------

AtCIPK12 ---------- ---------- KGYDAAKVDI WSCGVILFVL MAGYL-PFHD RNVMAM---- ---YKKIYRG EF-------- ---------- ----------

AtCIPK19 ---------- ---------- KGYDGAKVDI WSCGVILFVL MAGFL-PFHD RNVMAM---- ---YKKIYRG DF-------- ---------- ----------

AtCIPK18 ---------- ---------- KGYDAAKADV WSCGVILFVL MAGHI-PFYD KNIMVM---- ---YKKIYKG EF-------- ---------- ----------

AtCIPK13 ---------- ---------- KGYEGAKADI WSCGVILFVL MAGYL-PFDD KNILVM---- ---YTKIYKG QF-------- ---------- ----------

AtCIPK16 ---------- ---------- KGYDGAMADI WSCGIVLYAL LAGFL-PFID ENVMTL---- ---YTKIFKA EC-------- ---------- ----------

AtCIPK5 ---------- ---------- KGYDGAKADI WSCGVVLYVL LAGCL-PFQD ENLMNM---- ---YRKIFRA DF-------- ---------- ----------

AtCIPK25 ---------- ---------- KGYDGAKGDI WSCGIILYVL LAGFL-PFQD ENLMKM---- ---YRKIFKS EF-------- ---------- ----------

AtCIPK2 ---------- ---------- KGYEGTKADI WSCGVVLFVL LAGYL-PFHD TNLMEM---- ---YRKIGKA DF-------- ---------- ----------

AtCIPK10 ---------- ---------- KGYDGTKADI WSCGVVLFVL LAGYL-PFHD SNLMEM---- ---YRKIGKA DF-------- ---------- ----------

AtCIPK15 ---------- ---------- NGYDGFKADV WSCGVILFVL LAGYL-PFRD SNLMEL---- ---YKKIGKA EV-------- ---------- ----------

AtCIPK6 ---------- ---------- KGYDGAKADL WSCGVILFVL LAGYL-PFQD DNLVNM---- ---YRKIYRG DF-------- ---------- ----------

AtCIPK20 ---------- ---------- KGYDGAKADV WSCGVVLYVL LAGFL-PFHE QNLVEM---- ---YRKITKG EF-------- ---------- ----------

AtCIPK21 ---------- ---------- KGYSGAAVDV WSCGVILFEL LAGYP-PFDD HTLPVL---- ---YKKILRA DY-------- ---------- ----------

AtCIPK17 ---------- ---------- EGYDGAASDI WSCGVILYVI LTGCL-PFDD ANLAVI---- ---CRKIFKG DP-------- ---------- ----------

AtCIPK1 ---------- ---------- RGYDGAASDI WSCGVILYVI LTGCL-PFDD RNLAVL---- ---YQKICKG DP-------- ---------- ----------

AtCIPK3 ---------- ---------- RGYDGATADM WSCGVVLYVL LAGYL-PFDD SNLMNL---- ---YKKISSG EF-------- ---------- ----------

AtCIPK9 ---------- ---------- KGYDGAAADV WSCGVILFVL MAGYL-PFDE PNLMTL---- ---YKRICKA EF-------- ---------- ----------

AtCIPK23 ---------- ---------- KGYDGAKADL WSCGVILFVL MAGYL-PFED SNLTSL---- ---YKKIFKA EF-------- ---------- ----------

AtCIPK8 ---------- ---------- KGYNGAVADI WSCGVILYVL MAGYL-PFDE MDLPTL---- ---YSKIDKA EF-------- ---------- ----------

AtCIPK24 ---------- ---------- QGYDGSAADI WSCGVILFVI LAGYL-PFSE TDLPGL---- ---YRKINAA EF-------- ---------- ----------

CrMAPK7 ---------- S-PYYN---- -----APIDL FAMGAIMAEL YMLRP-LFPG TSEADE---- ---IYKICSI MGTPTQQVWP EGLKLAATM- ----------

CrMAPK9 ---------- S-THYG---- -----KEVDL WAIGCIMAEL LDGQP-LFPG ESDIDQ---- ---LYILQRL LGPLTREQHD LFLRNPRFNG LKFPD-----

CrMAPK13 ---------- C-EPYD---- -----TAVDI WSFGCTLAEL MSGTP-LFPG SSSPDQ---- ---LWRIMRC CGALADRHAA YLKFYPRLAA LAE-------

Cre03.g206202 ---------- --QAYG---- -----PPADI FSLGTTIAEL AVGIP-LFPG ASTTDQ---- ---LNRLCRC FGPLPQHMTA ALSAMAARSA TPAVAAELSV

CrMAPK14 ---------- --DNYG---- -----PPVDV WALGCIFAEM LAGRP-LFPG KNHHDQ---- ---LWLILKC LGTMTERQLE LLDSDPQFAC FRL-PTQSE-

CrMAPK17 ---------- --MPYG---- -----PGADV WSFGATLAEL ATGEP-LLPG DSSLDQ---- ---LARIMRA TGPLNPHMAT CLSRDLRTPL TSL-LANHAA

CrMAPK16 ---------- --QEYG---- -----PPADI WSFGCTVAEM ATGVP-LLPG TSTADQ---- ---LWRIIRM FGPLPPDMAL CLAHDPLLI- ------HMTA

CrMAPK15 ---------- --DHYG---- -----ASADI WSLGCTIAEM ATGRP-LFQG TSSLDQ---- ---LWHIVRC LGPLSPQQAA CVAANPHLQ- ------PLAA

CrMAPK12 ---------- --APYG---- -----PASDV WSIGCTMAEL ATGRP-LFVG SSTADQ---- ---LRRIMRC LGPLSPPHMS RIASDPRLA- ------NVQ-

CrMAPK10 ---------- --DEYG---- -----AASDI WSVGCSIAEF AVGRA-LFPG SSTADQ---- ---LWRIMRC FGPLPSGLSM RMATDPRLR- ------SLC-

CrMAPK11 ---------- --DNYG---- -----AAADI WSIGCTIAEI ATGRA-LFPG NSTSDQ---- ---LWRIMRC LGPLPPAHTA RLLSDPRLR- ------GAT-

CrGSK3 ---------- A-TDYT---- -----SAIDV WSVGCVLAEL LLGQP-LFPG ESGVDQ---- ---LVEIIKV LGTPTREEIN AMNPNYTE-- ----------

CrCDKI1 ---------- A-RAYG---- -----PAVDV WALGLVFAEL LGLSP-LVPG DNDIDQ---- ---LGRVIAT FGSMEPVWLG VRALPDWG-- ----------

CrMAPK5 ---------- S-TKYT---- -----FGVDM WSSGCILGEL LMGKP-VFPG TSTMNQ---- ---LDRIVEF CGRPSPSDVE AIDSPFAATM ----------

CrMAPK8 ---------- C-SGYT---- -----TAIDV WSVGCIFAEL LGRKP-LFPG KDYVHQ---- ---LSLITKV IGSPSEEELG FITSEKAKRY ----------

CrMAPK6 ---------- C-DTYT---- -----TAIDV WSVGCILAEL LGRKP-LLPG KDYVDQ---- ---LKLIIKT LGPPSEEDLG FITSSKARAY ----------

CrMAPK3 ---------- C-EHYT---- -----SAIDM WSVGCIMAEL LGRKP-LLPG KDYVDQ---- ---LKLIIKS LGPPSEDDLT FINSQKARAY ----------

CrMAPK2 ---------- FFAKYS---- -----PAIDI WSVGCIFAEV LLGKP-LFPG RNVVHQ---- ---LELITDL LGTPSPEVIA KVRNEKARRF ----------

CrMAPK4 ---------- FYGRYS---- -----SAVDM WSIGCIFAEV LLGKP-LFPG RDAVHQ---- ---LQLITDL LGKPPPHVVD AIGNVKARAF ----------

CrCDPK5 ---------- --GAQT---- -----PACDV YSLGVVLFIM LIGRP-PHSG TDIRYLT--- --YSDKSIKD APGL------ ---------- ----------

CrCDPK4 ---------- --K-TE---- -----PASDI WSAGIMAYQL LSGAV-PFDD VKNPKSP-SL SLVWRAILTQ EPKF------ ---------- ----------

CrCDPK3 ---------- --S-YG---- -----KSCDV WSLGVILHVL LSGAP-PFAG ATDAEI---- ---IKAVRSR DVDL------ ---------- ----------

PpCCaMK ---------- --SGIM---- -----PANDM WSLGVILYIL LCGYP-PFRA RSSRDK---- ---QMQILTG SYSM------ ---------- ----------

NtCCaMK ---------- --ENIT---- -----TKSDI WSLGVILYIL LSGYP-PFIA PSNRKK---- ---QQMILNG QFSF------ ---------- ----------

LjCCaMK ---------- --GKIT---- -----AKSDM WSLGVILYIL LSGYP-PFIA QNNRQK---- ---QQMIING NFSF------ ---------- ----------

MtCCaMK ---------- --GKIT---- -----TKSDM WSLGVILYIL LSGYP-PFIA QNNRQK---- ---QQMIMNG NFSF------ ---------- ----------

PsCCaMK ---------- --GKIT---- -----TKSDM WSLGVILYIL LSGYP-PFIA QNNRQK---- ---QQMILNG NFSF------ ---------- ----------

LlCCaMK ---------- --RQVS---- -----SASDM WSLGVILYIL LSGCP-PFHA PSNREK---- ---QQRILAG DFSF------ ---------- ----------

OsCCaMK ---------- --QEVS---- -----AASDM WSVGVILYIL LSGCP-PFHA ATNREK---- ---QQRILQG EFSF------ ---------- ----------

Cre03.g199050 ---------- ---------- KGHN-GAADW WALGVLVFEL CNGLP-PFMD DD-------R LAMFRKICAR ELSF------ ---------- ----------

CrCDPKK1 ---------- ---------N TRYRGRPTDV YALGACLYTL LFGRI-PFSA PN-------L YKLFQVVQNE PVKY------ ---------- ----------

CrCDPKK2 ---------- ---------G KQFNGFPGDI WALGICLFMF VFGKP-PFVG AT-------T YQIYEAIQRA ELAF------ ---------- ----------

AtMKK10 ---------- --EKWGF--- GGEVGFAGDV WSLGVVVLEC YIGRY-PLTK VGDKPDWAT- --LFCAICCN EKVD------ ---------- ----------

AtMKK4 ---------- --DLNQ---- GKYDGYAGDI WSLGVSILEF YLGRF-PFP- VSRQGDWAS- --LMCAICMS QPPE------ ---------- ----------

AtMKK5 ---------- --DLNH---- GRYDGYAGDV WSLGVSILEF YLGRF-PFA- VSRQGDWAS- --LMCAICMS QPPE------ ---------- ----------

AtMKK8 ---------- --EADGVTEE DKSNVYAGDI WSFGLTMLEI LVGYY-PMLP -----DQAA- --IVCAVCFG EPPK------ ---------- ----------

AtMKK7 ---------- --A-AG---- ENSDVYAGDI WSFGVMILEL FVGHF-PLLP QGQRPDWAT- --LMCVVCFG EPPR------ ---------- ----------

AtMKK9 ---------- --ESSG---- GSSDIYAGDI WSFGLMMLEL LVGHF-PLLP PGQRPDWAT- --LMCAVCFG EPPR------ ---------- ----------

CrMAPKK1 ---------- --QAYS---- -----FPADI WSLGLALVEL ATGRY-PYDA GE------GP LQLMIHVLQE DAPL------ ---------- ----------

AtMKK3 ---------- --DSYS---- -----YPADI WSLGLALFEC GTGEF-PYIA NE------GP VNLMLQILDD PSPT------ ---------- ----------

CrMAPKK-L ---------- --DSYS---- -----FDSDL WSLGLTLLEC ALGRF-PYPP PGETGVNLGF WELLEYIVME PPPA------ ---------- ----------

AtMKK6 ---------- --STYD---- -----YSSDI WSLGMSVLEC AIGRF-PYLE SEDQQNPPSF YELLAAIVEN PPPT------ ---------- ----------

AtMEK1 ---------- --SLYS---- -----NKSDI WSLGLVLLEC ATGKF-PYTP PEHKKGWSSV YELVDAIVEN PPPC------ ---------- ----------

AtMKK2 ---------- --NKYG---- -----NKSDI WSLGLVVLEC ATGKF-PYAP PNQEETWTSV FELMEAIVDQ PPPA------ ---------- ----------

AtMAPKKK13 ---------- --EYQG---- -----PESDV WSLGCTIIEM FTGKP-AWED H--G-----I DSLSRISFSD ELPV------ ---------- ----------

AtMAPKKK14 ---------- --EYQG---- -----PESDV WSLGCTVIEM LTGKP-AWED H--G-----F DSLSRIGFSN DLPF------ ---------- ----------

Cre10.g464100 ---------- --SGHG---- -----RAADI WSLGCVVIEM ATGRA-PWAN FSDP-----Y AVMYHVAATK ELPA------ ---------- ----------

AtANP2 ---------- --TGHS---- -----FSADI WSVGCTVIEM VTGKA-PWSQ QYKE-----I AAIFHIGTTK SHPP------ ---------- ----------

AtANP1 ---------- --TGHS---- -----FSADI WSVGCTVIEM VTGKA-PWSQ QYKE-----V AAIFFIGTTK SHPP------ ---------- ----------

AtANP3 ---------- --TGHS---- -----FSADI WSVGCTVIEM ATGKP-PWSE QYQQ-----F AAVLHIGRTK AHPP------ ---------- ----------

Cre03.g169100 --TAPQLAAK NIRPYT---- -----AAVDV WAVGCLAYEL VSGRP-PFEV ED-------E KQTASNIIYS NNIR------ ---------- ----------

Cre17.g735550 --------PT TPARYG---- -----FATDC WAVGVVTFEL LTGLP-PYAS DC-------R YQAEARIIAA APPP------ ---------- ----------

CrALK3 --------DN ERLHYS---- -----ARVDA WAVGVLTYEL LVGFP-PFFD QS-------R TSTEERINH- SMPA------ ---------- ----------

CrALK1 --------EL KHLHYT---- -----PLVDA WAVGVLAYEL IVGRP-PFDK GQ-------K KATIAEILN- SEPF------ ---------- ----------

CrMAPKKK3 ---------- --ERCD---- -----EKSDV FSFGVILYEL VTGRE-PWEE LNP------M QVVGVVGFNG QRMD------ ---------- ----------

AtEDR1 ---------- --EPSN---- -----EKCDV YSFGVILWEL ATLRL-PWRG MNP------M QVVGAVGFQN RRLE------ ---------- ----------

AtCTR1 ---------- --EPSN---- -----EKSDV YSFGVILWEL ATLQQ-PWGN LNP------A QVVAAVGFKC KRLE------ ---------- ----------

CrCNK9 ---------- --KPYG---- -----MPNDI WGLGCVLYEV SALKP-AFQA FN-------M AGLIKRVTTG AAPA------ ---------- ----------

AtNEK7 ---------- --QPYG---- -----YKSDI WSLGCCMYEI TAHQP-AFKA PD-------M AGLINKINRS LMSP------ ---------- ----------

AtNEK5 ---------- --IPYG---- -----YKSDI WSLGCCMFEV AAHQP-AFKA PD-------M AALINKINRS SLSP------ ---------- ----------

AtNEK6 ---------- --IPYG---- -----FKSDI WSLGCCIYEM AAYRP-AFKA FD-------M AGLISKVNRS SIGP------ ---------- ----------

AtNEK4 ---------- --IPYG---- -----SKSDI WSLGCCMYEM AAHKP-PFKA SD-------V QTLITKIHKL IMDP------ ---------- ----------

AtNEK1 ---------- --IPYG---- -----SKSDI WSLGCCIYEM AYLKP-AFKA FD-------M QALINKINKT IVSP------ ---------- ----------

AtNEK2 ---------- --IPYG---- -----SKSDI WSLGCCMYEM TAMKP-AFKA FD-------M QGLINRINRS IVPP------ ---------- ----------

AtNEK3 ---------- --IPYG---- -----SKSDI WSLGCCMYEM TALKP-AFKA FD-------M QGLINRINRS IVAP------ ---------- ----------

CrCNK7 ---------- --QPYD---- -----FSSDI WSLGCLLYEL VALRN-PFYK ENQS-----L YVLGKNIQNC AYEP------ ---------- ----------

CrCNK6 ---------- --AGYD---- -----WKSDV WSMGCLLYEL ACLRS-PFEM EGAN-----L YDVFQKISKG EYSP------ ---------- ----------

CrCNK4 ---------- --KPYN---- -----EKSDV WALGVVLYEC CTQRH-PFDA DN-------Q GALILKILRG KFPP------ ---------- ----------

CrFA2 ---------- --KPYD---- -----AKSDV WALGVVMYEC CMGHY-PFDV ENNN-----Q VALIRKIARG VFKP------ ---------- ----------

CrCNK1 ---------- --RPYS---- -----YSSDM WSLGAVLYEM MTFRT-PMEG RT-------M ADLRNRIMGG RYTP------ ---------- ----------

CrCNK2 ---------- --RPYS---- -----YTSDT WAIGCLLYEL AALAV-PFEA RS-------M SELRYKVLRG TYPP------ ---------- ----------

CrCNK8 ---------- --QPYS---- -----FKSDV WALGCVMYEM MTGRA-AFAA DN-------L SRVVLRVIRG QYDP------ ---------- ----------

CrCNK5 ---------- --QPYT---- -----YKSDI WSLGCVLYEL CTLRH-AFAA DS-------L LSLVYQIVRG NFPP------ ---------- ----------

CrFAP403 ---------- --RKYN---- -----QKSDI WSLGCVLYEL ATLKH-AFEA PN-------M RALIQKIIKG SYPP------ ---------- ----------

CrCNK3 ---------- --RPYN---- -----RKSDV WSLGCVLYEL ATLRR-AFDG QS-------L PALVVKILRG KYPP------ ---------- ----------

CrCDPKK3 ---------- --YRSS---- -----FELDM WALGVCLYMW VFGEL-PFKG SAPFVV---- YE--N----- ---------- ---------- ----------

CrMAPK1 ---------- ---------- ---------- ---------- ---------- -----I---- YE--L----- ---------- ---------- ----------

Cre08.g379600 IGHHGQDSSC SGSSSS---- -----GDAGD AVEGLAG--- -MGEGEEVG- ---------- MA-GW--YD- ---------- ---------- ----------

Cre02.g108601 -------AAF GAAAAI---- -----GDDDD --TPFLA--- -VSSVAPSGQ AS-SRP---- PS--W--SLA TAG------- ------G--G ----------

Cre02.g108750a TAPGGRISAT GAASGS---- -----SKVAA --SPFLT--- -ISPGASPAQ ---SRT---- PS-AN--ALG SRP------- ------G--S ----------

Cre17.g713750 ---------- SNVPLT---- -----AAVDV YSFGITALCV AV-TGSNSAA ADWFAR---- ES-WW--PAG DAP------- ------A--A ----------

Cre11.g467586 ---------- SNVPLT---- -----AAVDV YSFGITALCV AV-TGSNSAA ADWFAQ---- QS-WW--PAG DAP------- ------A--A ----------

Cre11.g467588 ---------- SNVPLT---- -----AAVDV YSFGITALCV AV-TGSNSAA ADWFAQ---- QS-WW--PAG DAP------- ------A--A ----------

Cre11.g467589 ---------- SNVPLT---- -----AAVDV YSFGITALCV AV-TGSNSAA ADWFAQ---- QS-WW--PAG DAP------- ------A--A ----------

Cre11.g467584 ---------- SNVPLM---- -----AAVDV YSFGITALCV AV-TGSNSAA AEWFAQ---- QS-WW--PAG DAP------- ------A--A ----------

Cre11.g467585 ---------- SNVPLT---- -----AAVDV YSFGITALCV AV-TGSNSAA ADWFAQ---- QS-WW--PAG DAP------- ------A--A ----------

Cre12.g489850 -RASGAEPPT PLPPLT---- -----RAVDI WSLGALAVCL MV-TGTDWNL QDEYVR---- ---------- ---------- ---------- ----------

Cre12.g490200 -RMR------ TKLPLT---- -----RAVDI YSLGVLAVCL LV-TGTFQES AARYAH---- ---------- ---------- ---------- ----------

Cre08.g384250 ---------- -ARYSG---- -----PKADI WSCGVVLCTL LFGC-HPHLA AA-------- -------DLD AAP------- ------G--D ----------

Cre04.g217940 ---------- -----A---- -----AKSDI YAVGVIGAAL VCGT-ESGPE M--------- ---------- ---------- ---------- ----------

Cre04.g217923 ---------- -----A---- -----AKSDV FAVGVIGAAL VCGT-ESGPE M--------- ---------- ---------- ---------- ----------

Cre04.g217928 ---------- -----A---- -----AKSDV FAVGVIGAAL VCGT-ESGPE M--------- ---------- ---------- ---------- ----------

Cre08.g369700 ---------- -GRISG---- -----RKADS YSLGRMLTYL AAKP-SEMDS VL-------- -------P-- ---------- ---------- ----------

Cre08.g369900 ---------- -GRISG---- -----PKADC YSLGRMLTYL AAKP-SEMDS VL-------- -------S-- ---------- ---------- ----------

Cre03.g187300 AAVHAAVAAA VHAAAS---- -----PAADM YSLGVLLTAC VVWHGDVGR- MR-------- ---------- ---------- ---------- ----------

Cre01.g041752 -----VDSEA AVPVVT---- -----VACDV ASVGLLLLDA AAFRATAGG- LR-------- ---------- ---------- ---------- ----------

Cre12.g495401 -----IDSEA AVPVVT---- -----VACDV ASVGLLLLDA AAFRATAGG- LR-------- ---------- ---------- ---------- ----------

Cre06.g266450 ---------- ----LS---- -----RMIDM ASVGLLLADA AQFCVEAGR- LV-------- ---------- ---------- ---------- ----------

Cre06.g266000 ---------- ----LS---- -----RMIDM ASVGVLLAEA AQFRPMPTR- LW-------- ---------- ---------- ---------- ----------

Cre06.g266250 ---------- ----LS---- -----RMIDM ASVGLLLAEA AQFRPMPTR- LW-------- ---------- ---------- ---------- ----------

Cre10.g460700 ---------- ----VC---- -----LQSDM AAIGMVMAHA AAFHGLPGG- LQ-------- ---------- ---------- ---------- ----------

Cre12.g545750 ---------- ----IT---- -----MAADV ASMGLVLADA AGLHGLAGG- TQ-------- ---------- ---------- ---------- ----------

Cre12.g525950 ---------- ----IT---- -----RAADV ASMGLVLADA AGLHCLGGG- TQ-------- ---------- ---------- ---------- ----------

Cre12.g525750 ---------- ----IT---- -----RAADV ASMGLVLADA AGLHCLGGG- TQ-------- ---------- ---------- ---------- ----------

Cre12.g526650 ---------- ----IT---- -----RAADV ASMGLVLADA AGLHCLGGG- TQ-------- ---------- ---------- ---------- ----------

Cre12.g526051 ---------- ----IT---- -----RAADV ASMGLVLADA AGLHCLGGG- TQ-------- ---------- ---------- ---------- ----------

Cre12.g526250 ---------- ----IT---- -----RAADV ASMGLVLADA AGLHCLGGG- TQ-------- ---------- ---------- ---------- ----------

Cre01.g054750 ---------- ----IT---- -----KAADI ASAGLVLADA AGLHCLGVCS TQ-------- ---------- ---------- ---------- ----------

Cre01.g055457 ---------- ----IT---- -----KAADI ASAGLVLADA AGLHCLGGG- TT-------- ---------- ---------- ---------- ----------

Cre09.g404550 ---------- ---------- ---------- ---------- ---------- ---------- ---------- ---------- ---------- ----------

Cre02.g108750b ---------- --MPYN---- -----EKVDV FSFGVVMYEL LSRDLLLISH MNTSRA---- AQLGI----- ---------- ---------- ----------

Cre02.g095099 ---------- ---------- ---------- ---------- ---------- ---------- ---------- ---------- ---------- ----------

Cre17.g710600 ---------- --GEMR---- -----REGDV YAFGVLLWEL CSGVR-AYRG LSSGEV---- VQGVL--LRG LRP---HTAA AAATAAALAS ----------

CrMAPKKK14 ---------- --PRLD---- -----ASADI YSLGITMWEL LAGGQRPHAA VPVDRI---- P--------- ---------- ---------- ----------

CrMAPKKK4 ---------- --ARVE---- -----ASADV YSFGILCWEL VAGGIRPFPH LHPDLI---- P--------- ---------- ---------- ----------

CrMAPKKK8 ---------- ---PVS---- -----EKVDV YAFAMTLYEC LTGEQ-PWRD LQNPMQ---- VIFVV--GV- ---------- ---------- ----------

CrMAPKKK1 ---------- -NYVIT---- -----HKADI YALGVILWEM LTGSV-PWEG CSMVAI---- AY-SI--TV- ---------- ---------- ----------

CrMAPKKK12 ---------- RAVAVT---- -----YKADI YSFAVVLWCM LTGQE-PWQG LGAVSI---- AV-QV--AL- ---------- ---------- ----------

Cre09.g390300 ---------- --KAVT---- -----DKADV YSFGVVMWEL WTFRE-PFDG LNYHAL---- LHAIS--MSK ---------- -DPVRPAMPG ----------

CrMAPKKK11 ---------- --GMFS---- -----RASDV YAFGMVLYEM ATGER-PYEH LNPAQV---- ---------- ---------- ---------- ----------

CrMAPKKK13 ---------- --GILS---- -----PSADV YAMGVLLWEA YSGDR-VFKQ LSDSEV---- ---------- ---------- ---------- ----------

CrPTK7 ---------- --GHIS---- -----KHCDT YAFGILLFEL FSGER-AHRG VPRALL---- ---------- ---------- ---------- ----------

Cre02.g117813 ---------- --GQMR---- -----PSCDV FSFGIMLWQM ATGGV-PFAG MRYAEI---- VY-K------ ---------- ---------- ----------

CrMAPKKK10 --------SA ASPALT---- -----QRGDV WSFGILLLEL YYGVG-LDDC RRLYNS---- LQLEA----- ---------- ---------- ----------

CrMAPKKK2 ---------- --GRLS---- -----SAADV WSFGCILLEL THGER-ISRI RARAAT---- AAAAA--PAA ---------- -AAPAPAAAG ----------

CrMAPKKK6 ---------- --GNMT---- -----KAADV YSMGVMMWEM YHSCM-SYRS ----LP---- ---SG--FA- ---------- ---------- ----------

CrMAPKKK5 ---------- --GRVG---- -----PAADV FSLGVILWEL YHGRR-A--- -GVRTQ---- ---EG--PR- ---------- ---------- ----------

CrMAPKKK9 ---------- --GQVG---- -----ASSDV FSLGVLLWEL FHGRR-A--- -GVLTK---- ---EG--PR- ---------- ---------- ----------

Cre12.g516650 ---------- --GKVG---- -----KSADL YSLGIMLWEL QNGTR-PPWR MGVRLR---- ---TY--PS- ---------- ---------- ----------

CrMAPKKK7 ---------- --GKIG---- -----KSADL YSLGIMLWEL QNGTR-PPWR MGVRLR---- ---TY--PS- ---------- ---------- ----------

....|....| ....|....| ....|....| ....|....| ....|....| ....|....| ....|....| ....|....| ....|....| ....|....|

1210 1220 1230 1240 1250 1260 1270 1280 1290 1300

CrCDPK9 ---------- ---------- ---------- ---------- -------FKT DPWPKISEAA KDCVKRLLDQ DVT---K--- --RATAAQI- LKHEWL----

AtCPK25 ---------- ---------- ---------- ---------- -------LTS DPWPQVSESA KDLIRKMLER NPI---Q--- --RLTAQQV- LCHPWI----

AtCPK26 ---------- ---------- ---------- ---------- -------FDS DPWPLISDSA KNLIRGMLCS RPS---E--- --RLTAHQV- LRHPWI----

AtCPK6 ---------- ---------- ---------- ---------- -------FDT DPWPVISDSA KDLIRKMLCS SPS---E--- --RLTAHEV- LRHPWI----

AtCPK5 ---------- ---------- ---------- ---------- -------FES DPWPVISDSA KDLIRRMLSS KPA---E--- --RLTAHEV- LRHPWI----

AtCPK20 ---------- ---------- ---------- ---------- -------FIS EPWPSVSESA KDLVRRMLIR DPK---K--- --RMTTHEV- LCHPWA----

AtCPK2 ---------- ---------- ---------- ---------- -------FSS DPWPSISESA KDLVRKMLVR DPK---R--- --RLTAHQV- LCHPWV----

AtCPK1 ---------- ---------- ---------- ---------- -------FSS DPWPSISESA KDLVRKMLVR DPK---K--- --RLTAHQV- LCHPWV----

AtCPK12 ---------- ---------- ---------- ---------- -------FEI NPWPSISESA KDLIKKMLES NPK---K--- --RLTAHQV- LCHPWI----

AtCPK11 ---------- ---------- ---------- ---------- -------FKS DPWPTISEAA KDLIYKMLER SPK---K--- --RISAHEA- LCHPWI----

AtCPK4 ---------- ---------- ---------- ---------- -------FKS DPWPTISEGA KDLIYKMLDR SPK---K--- --RISAHEA- LCHPWI----

AtCIPK7 ---------- ---------- ---------- ---------- --------RF PSW--ISKQA KSIIYQMLDP NPV---T--- --RMSIETV- MKTNWF----

AtCIPK4 ---------- ---------- ---------- ---------- --------RF PSW--ISKPA RSIIYKLLDP NPE---T--- --RMSIEAV- MGTVWF----

AtCIPK22 ---------- ---------- ---------- ---------- --------KL PDW--TSSDL RKL------- ---------- ---------- ----------

AtCIPK11 ---------- ---------- ---------- ---------- --------RF PRW--MSPDL KRFVSRLLDI NPE---T--- --RITIDEI- LKDPWF----

AtCIPK14 ---------- ---------- ---------- ---------- --------RI PKW--TSPDL RRLLTRLLDT NPQ---T--- --RITIEEI- IHDPWF----

AtCIPK12 ---------- ---------- ---------- ---------- --------RC PRW--FSTEL TRLLSKLLET NPE---K--- --RFTFPEI- MENSWF----

AtCIPK19 ---------- ---------- ---------- ---------- --------RC PRW--FPVEI NRLLIRMLET KPE---R--- --RFTMPDI- METSWF----

AtCIPK18 ---------- ---------- ---------- ---------- --------RC PRW--FSSDL VRLLTRLLDT NPD---T--- --RITIPEI- MKNRWF----

AtCIPK13 ---------- ---------- ---------- ---------- --------KC PKW--FSPEL ARLVTRMLDT NPD---T--- --RITIPEI- MKHRWF----

AtCIPK16 ---------- ---------- ---------- ---------- --------EF PPW--FSLES KELLSRLLVP DPE---Q--- --RISMSEI- KMIPWF----

AtCIPK5 ---------- ---------- ---------- ---------- --------EF PPW--FSPEA RRLISKLLVV DPD---R--- --RISIPAI- MRTPWL----

AtCIPK25 ---------- ---------- ---------- ---------- --------EY PPW--FSPES KRLISKLLVV DPN---K--- --RISIPAI- MRTPWF----

AtCIPK2 ---------- ---------- ---------- ---------- --------KC PSW--FAPEV KRLLCKMLDP NHE---T--- --RITIAKI- KESSWF----

AtCIPK10 ---------- ---------- ---------- ---------- --------KA PSW--FAPEV RRLLCKMLDP NPE---T--- --RITIARI- RESSWF----

AtCIPK15 ---------- ---------- ---------- ---------- --------KF PNW--LAPGA KRLLKRILDP NPN---T--- --RVSTEKI- MKSSWF----

AtCIPK6 ---------- ---------- ---------- ---------- --------KC PGW--LSSDA RRLVTKLLDP NPN---T--- --RITIEKV- MDSPWF----

AtCIPK20 ---------- ---------- ---------- ---------- --------KC PNW--FPPEV KKLLSRILDP NPN---S--- --RIKIEKI- MENSWF----

AtCIPK21 ---------- ---------- ---------- ---------- --------TF PPG--FTGEQ KRLIFNILDP NPL---S--- --RITLAEII IKDSWF----

AtCIPK17 ---------- ---------- ---------- ---------- --------PI PRW--ISLGA KTMIKRMLDP NPV---T--- --RVTIAGI- KAHDWF----

AtCIPK1 ---------- ---------- ---------- ---------- --------PI PRW--LSPGA RTMIKRMLDP NPV---T--- --RITVVGI- KASEWF----

AtCIPK3 ---------- ---------- ---------- ---------- --------NC PPW--LSLGA MKLITRILDP NPM---T--- --RVTPQEV- FEDEWF----

AtCIPK9 ---------- ---------- ---------- ---------- --------SC PPW--FSQGA KRVIKRILEP NPI---T--- --RISIAEL- LEDEWFKK--

AtCIPK23 ---------- ---------- ---------- ---------- --------TC PPW--FSASA KKLIKRILDP NPA---T--- --RITFAEV- IENEWF----

AtCIPK8 ---------- ---------- ---------- ---------- --------SC PSY--FALGA KSLINRILDP NPE---T--- --RITIAEI- RKDEWF----

AtCIPK24 ---------- ---------- ---------- ---------- --------SC PPW--FSAEV KFLIHRILDP NPK---T--- --RIQIQGI- KKDPWF----

CrMAPK7 ---------- ---------- ---------- ---NFRFPQF A---PTPLNK IIT-NACPEA IDLMTQLCHW DPN---K--- --RPTAVQA- LQHPYF----

CrMAPK9 --MRNPETL- ---------- ---------- ---------- -------DRK YAG-KMPHDA LAFMKALLAV DPS---A--- --RLTCSQA- LSHPYL----

CrMAPK13 -TPAPGRTL- ---------- ---------- ---------- -------AER -VP-AACPQM LDLINACLNM DPL---K--- --RPTATQL- LQHPFF----

Cre03.g206202 VGAQRGRSLK QRLYGCCDGT GGRTTYATGG GGGGMSGPAA A---PSPRRS ARA-EPDPQL LEFLQACMEL DPA---M--- --RLTADQL- LDLPYF----

CrMAPK14 -----IEPL- ---------- ---------- ---------- -------EHR -LS-ALSGPA MQFLRACLNP DPK---Q--- --RATADEL- LAMPYF----

CrMAPK17 QKGPNG-LL- ---------- ---------- ---------- -------RQT LGA-RLSPAL LDLIEACLRP DPK---D--- --RPTAEQL- LRLPYF----

CrMAPK16 --PPPGMAL- ---------- ---------- ---------- -------RQR -LA-KLGPRL LELLEVCLRP DPR---D--- --RPTAEEL- MHLPYF----

CrMAPK15 LKPPVSKTL- ---------- ---------- ---------- -------RQR -LP-HLEPRL FQVVEACLRT DPR---Q--- --RPTAREL- LQLPYF----

CrMAPK12 LPDP-HLTL- ---------- ---------- ---------- -------RQR -LP-EVEPRL LALIYCCLQP DPQ---L--- --RPTAAEL- LQMPYF----

CrMAPK10 APQPLSRNL- ---------- ---------- ---------- -------RQR -LP-EVEPRL LELVAACLRL DPA---A--- --RPTAAEL- LQMPYFWDVP

CrMAPK11 I-PPLHKDL- ---------- ---------- ---------- -------RQR -LP-EVEPRL LELVAACLRL DPA---A--- --RPTAAEL- LQMPYF----

CrGSK3 ---------- ---------- ---------- ----FKFPQI K---AHPWTK VFSKRMPPDA VDLVSKLLQY APQ---K--- --RMTAVQA- MTHPFF----

CrCDKI1 ---------- ---------- ---------- ---KIAFPHC E---PLPLGD LLP-GAPAAA LEFLAAFLRY DPA---K--- --RITAACA- VRSTYL----

CrMAPK5 ---------- ---------- ---------- ---MESCSVS Q---ARRLQD VFP-HASPDA ADLLRKLLVF NPH---K--- --RLTAEQA- LRHPYV----

CrMAPK8 ---------- ---------- ---------- ---IRSLPRS E---RVDFGQ LWP-HVTKTA LDLIDKMLVF DPT---K--- --RITVEQA- LEHPYL----

CrMAPK6 ---------- ---------- ---------- ---IRALPPS E---KINFRK LFP-DADPLA IDLMEKMLQF DPR---K--- --RIDVIQA- LKHPWL----

CrMAPK3 ---------- ---------- ---------- ---IRALPPA E---KVNFRK KFP-EADPLA IDLMEKMLQF DPR---K--- --RIDVHGA- LKHPWL----

CrMAPK2 ---------- ---------- ---------- ---LLNMRPK P---GIPFEQ HFP-RADRGA LRLLRRLLAF DPA---E--- --RPTAEEA- LADPYF----

CrMAPK4 ---------- ---------- ---------- ---LNALEPK R---PRQLSS KFP-AADPLA LDLLGHLLAF DPA---D--- --RPTAAEA- LAHPYF----

CrCDPK5 ---------- ---------- ---------- ---------- -------QD- ARYRALSADA KDLLLKMLAD DPK---A--- --RPTCLEV- LKHPFM----

CrCDPK4 ---------- ---------- ---------- ---------- -------TG- RAWQGVSDTA KDFVRSLLAK DPK---E--- --RPTAKQA- LAHPWL----

CrCDPK3 ---------- ---------- ---------- ---------- -------SA- KPWPAISRHA RHLVSRMLEK DPE---Q--- --RIQLDQV- LTHPWL----

PpCCaMK ---------- ---------- ---------- ---------- -------EE- ESWRGISHEA KQLIRRLLSV DPF---A--- --RPTAREL- LSHPWV----

NtCCaMK ---------- ---------- ---------- ---------- -------DE- KTWKNISSSA KQLISSLLKV DPN---M--- --RPTAQEI- LEHPWV----

LjCCaMK ---------- ---------- ---------- ---------- -------YE- KTWKGITQSA KQLISSLLTV DPS---K--- --RPSAQEL- LSHPWV----

MtCCaMK ---------- ---------- ---------- ---------- -------YE- KTWKGISQPA KNLISSLLTV DPS---K--- --RPSALEL- LSDPWV----

PsCCaMK ---------- ---------- ---------- ---------- -------YE- KTWKGISQSA KNLISSLLTV DPA---K--- --RPSAQEL- LSDPWV----

LlCCaMK ---------- ---------- ---------- ---------- -------EE- HTWKTITSSA KDLISSLLSV DPY---K--- --RPTANDL- LKHPWV----

OsCCaMK ---------- ---------- ---------- ---------- -------QD- HTWKTISSSA KDLISRLLSV QPY---K--- --RPTASDL- LRHPWV----

Cre03.g199050 ---------- ---------- ---------- ---------- -------PK- --HF--SKDL RHLVERLLDP NPL---FRAG AGREGAAEI- KAHPWF----

CrCDPKK1 ---------- ---------- ---------- ---------- -------PP- --DVPITEEL KDLLARMLTK NPR---E--- --RIGMSEL- MRHPWL----

CrCDPKK2 ---------- ---------- ---------- ---------- -------PH- --EIPVSGEL KDLLGRLLQK DPA---E--- --RISLEEI- PAHPWV----

AtMKK10 ---------- ---------- ---------- ---------- -------IP- --VS-CSLEF RDFVGRCLEK DWR---K--- --RDTVEEL- LRHSFV----

AtMKK4 ---------- ---------- ---------- ---------- -------AP- --AT-ASPEF RHFISCCLQR EPG---K--- --RRSAMQL- LQHPFI----

AtMKK5 ---------- ---------- ---------- ---------- -------AP- --AT-ASQEF RHFVSCCLQS DPP---K--- --RWSAQQL- LQHPFI----

AtMKK8 ---------- ---------- ---------- ---------- -------AP- --EE-CSDDL KSFMDCCLRK KAS---E--- --R------- ----------

AtMKK7 ---------- ---------- ---------- ---------- -------AP- --EG-CSDEF RSFVDCCLRK ESS---E--- --RWTASQL- LGHPFL----

AtMKK9 ---------- ---------- ---------- ---------- -------AP- --EG-CSEEF RSFVECCLRK DSS---K--- --RWTAPQL- LAHPFL----

CrMAPKK1 ---------- ---------- ---------- ---------- -------PP- --AGEFSEEF RDFVRVSLQK DPH---K--- --RPMAEQL- LTHPFI----

AtMKK3 ---------- ---------- ---------- ---------- -------PP- --KQEFSPEF CSFIDACLQK DPD---A--- --RPTADQL- LSHPFI----

CrMAPKK-L ---------- ---------- ---------- ---------- -------LP- --ADQFSPAL CDFVGQCLQK DAA---A--- --RPSVTAL- AAHPFL----

AtMKK6 ---------- ---------- ---------- ---------- -------AP- --SDQFSPEF CSFVSACIQK DPP---A--- --RASSLDL- LSHPFI----

AtMEK1 ---------- ---------- ---------- ---------- -------AP- --SNLFSPEF CSFISQCVQK DPR---D--- --RKSAKEL- LEHKFV----

AtMKK2 ---------- ---------- ---------- ---------- -------LP- --SGNFSPEL SSFISTCLQK DPN---S--- --RSSA---- ----------

AtMAPKKK13 ---------- ---------- ---------- ---------- -------FP- --SK-LSEIG RDFLEKCLKR DPN---Q--- --RWSCDQL- LQHPFL----

AtMAPKKK14 ---------- ---------- ---------- ---------- -------IP- --VG-LSELG RDFLEKCLKR DRS---Q--- --RWSCDQL- LQHPFL----

Cre10.g464100 ---------- ---------- ---------- ---------- -------MP- --DS-LSHAA KDFITLCFNR VPR---E--- --RPNATRL- LQHPWL----

AtANP2 ---------- ---------- ---------- ---------- -------IP- --DN-ISSDA NDFLLKCLQQ EPN---L--- --RPTASEL- LKHPFVTGKQ

AtANP1 ---------- ---------- ---------- ---------- -------IP- --DT-LSSDA KDFLLKCLQE VPN---L--- --RPTASEL- LKHPFV----

AtANP3 ---------- ---------- ---------- ---------- -------IP- --ED-LSPEA KDFLMKCLHK EPS---L--- --RLSATEL- LQHPFV----

Cre03.g169100 ---------- ---------- ---------- ---------- -------FD- --PA-YSAAW ADFVAQALIK DPR---R--- --RPTAAAL- LAHPWL----

Cre17.g735550 ---------- ---------- ---------- ---------- -------FP- --HG-MSHPA RDFIRRLLAP IPG---D--- --RPTVRQL- LAHPWI----

CrALK3 ---------- ---------- ---------- ---------- -------FP- --AG-MSEEA KAFVSSALSK HAA---E--- --RPTILEM- LHHPWI----

CrALK1 ---------- ---------- ---------- ---------- -------IP- --PW-LSEGA THFIRWSLTK EVG---K--- --RPSVQQL- AEHPWI----

CrMAPKKK3 ---------- ---------- ---------- ---------- -------LP- --PD-LDPGV TALITACWAD KPA---D--- --RPSFSQI- LAT--LTTWS

AtEDR1 ---------- ---------- ---------- ---------- -------IP- --KE-LDPVV GRIILECWQT DPN---L--- --RPSFAQL- TEV--LKPLN

AtCTR1 ---------- ---------- ---------- ---------- -------IP- --RN-LNPQV AAIIEGCWTN EPW---K--- --RPSFATI- MDL--LRPLI

CrCNK9 ---------- ---------- ---------- ---------- -------MP- --SC-YSENW RSLIKSMLTK EPE---L--- --RPSAAEL- LELDWL----

AtNEK7 ---------- ---------- ---------- ---------- -------LP- --IV-YSSTL KQMIKLMLRK KPE---Y--- --RPTACEL- LRNPSLQPYL

AtNEK5 ---------- ---------- ---------- ---------- -------LP- --VM-YSSSL KRLIKSMLRK NPE---H--- --RP------ ----------

AtNEK6 ---------- ---------- ---------- ---------- -------LP- --PC-YSPSL KALIKGMLRK NPE---Y--- --RPNASEI- LKHPYL----

AtNEK4 ---------- ---------- ---------- ---------- -------IP- --AM-YSGSF RGLIKSMLRK NPE---L--- --RPSANEL- LNHPHLQPYI

AtNEK1 ---------- ---------- ---------- ---------- -------LP- --AK-YSGPF RGLVKSMLRK NPE---V--- --RPSASDL- LRHPHL----

AtNEK2 ---------- ---------- ---------- ---------- -------LP- --AQ-YSAAF RGLVKSMLRK NPE---L--- --RPSAAEL- LRQPLL----

AtNEK3 ---------- ---------- ---------- ---------- -------LP- --AQ-YSTAF RSLVKSMLRK NPE---L--- --RPSASDL- LRQPLL----

CrCNK7 ---------- ---------- ---------- ---------- -------LP- --PS-VPDEL RTLVTSMLQP QPS---S--- --RPSISHV- ADQVDLYV--

CrCNK6 ---------- ---------- ---------- ---------- -------LP- --ADQFSAPL RSLVGRMLQI DPA---K--- --RPELEEV- WTITSGAV--

CrCNK4 ---------- ---------- ---------- ---------- -------VS- --G--YSPDI CDLIKRCLTQ NAN---R--- --RPNTYKL- LLLPSI----

CrFA2 ---------- ---------- ---------- ---------- -------VS- --GP-YTQQL IQLITSCLTL DPR---Q--- --RPDTTAL- LRNPSL----

CrCNK1 ---------- ---------- ---------- ---------- -------IP- --AGRYSAEL TNICHSLLAT DPA---K--- --RPSPTSI- LGSSAAAKWM

CrCNK2 ---------- ---------- ---------- ---------- -------IP- --N-TFSRDL QQMVRECLDP NPD---K--- --RPTMDQI- LASAAV----

CrCNK8 ---------- ---------- ---------- ---------- -------LP- --DS-YSSAL RMVVTSMLCK EVK---A--- --RPDANQL- LTVPAVVPHV

CrCNK5 ---------- ---------- ---------- ---------- -------IP- --TDQFSNGL SDLVNRLLWR DPA---T--- --RPSLSEV- FKLQFV----

CrFAP403 ---------- ---------- ---------- ---------- -------MP- --AT-RSKEL RDLCDRMLTL DWQ---K--- --RPSINDI- LATPVM----

CrCNK3 ---------- ---------- ---------- ---------- -------VP- --TR-YSTPL RGLIESMLKQ NPK---D--- --RPSADAI- LKSDFV----

CrCDPKK3 ---------- ---------- ---------- -------I-R SAQLALPA-- --QPRISAQM TDFISRLLDK DPR---T--- --RMTVHQA- VNHPWT----

CrMAPK1 ---------- ---------- ---------- -------I-R GRRH-YVA-- --EERIKN-- --YMYQLMKS M--------- --DHMHRNG- IFHRWGWAGG

Cre08.g379600 ---------- ---------- ---------- -------CSA DDFPVEPQS- --------NL AAT------- ---------- -------KA- VAAPQA----

Cre02.g108601 -----GGG-- ---------V ---------- -P-----MSR PLLPSGHSG- --------NR --------RR RPASA----- ---QAVILG- LN--------

Cre02.g108750a -----SST-- ---------A ---------- -P-----PQA QAVPAGSGK- --------AL LAK-ASQVHR QEQQEQQ--- --KQQGKAG- MAVPESPWA-

Cre17.g713750 ---------- ---------- ---------- ---------A RAFPAGAF-- --PSYLPLSL ELLLLDCVAW EPQ---E--- --RPTAEQV- VQRLAAIWEE

Cre11.g467586 ---------- ---------- ---------- ---------A RAFPAGIF-- --PSYLPLSL ELLLLDCVAW DPQ---E--- --RPTAEQV- VQRLAAI---

Cre11.g467588 ---------- ---------- ---------- ---------A RAFPAGIF-- --PSYLPLSL ELLLLDCVAW DPQ---E--- --RPTAEQV- VQRLAAI---

Cre11.g467589 ---------- ---------- ---------- ---------A RAFPAGIF-- --PSYLPLSL ELLLLDCVAW DPQ---E--- --RPTAEQV- VQRLAAI---

Cre11.g467584 ---------- ---------- ---------- ---------A RAFPAGIF-- --PSYLPLSL ELLLLDCVAW DPQ---E--- --RPTAEQV- VQRLAAI---

Cre11.g467585 ---------- ---------- ---------- ---------A RAFPAGIF-- --PSYLPLSL ELLLLDCVAW DPQ---E--- --RPTAEQV- VQRLAAIW--

Cre12.g489850 ---------- ---------- ---------- ---------- ---CGRAL-- --PAYVPEPM VELIRECTAD VAA---L--- --RPSAQQV- LARLAAMQIH

Cre12.g490200 ---------- ---------- ---------- ---------- ---RELPL-- --PPHVPSAL AQLVLDCTED KSH---Q--- --RPSAEQV- LQRLDAMEAE

Cre08.g384250 -----QIL-- ---------L ---------- -I-----LQN AVAGRIMVP- REEAAAQPEA AALMSAMLTV DPA---A--- --RPDATAV- LSHPWL----

Cre04.g217940 ---------- ---------- ---------- ---------E AFRASGELP- -PHRHASPAL RQLLKGMAAA DPA---Q--- --RLGVEEA- LAHPAL----

Cre04.g217923 ---------- ---------- ---------- ---------E AFRGSGELP- -PHRHASPAL RQLLKGMAAA DPA---Q--- --RLGVEEA- LAHPAL----

Cre04.g217928 ---------- ---------- ---------- ---------E AFRASGELP- -PHRHASPAL RQLLKGMAAA DPA---R--- --RLGVEEA- LAHPAL----

Cre08.g369700 ---------- ---------L ---------- -P-----AEG SVVGSAALAH LQQSRGGRRL ASLISGLTAV DPA---A--- --RLGVEEA- LAHPAL----

Cre08.g369900 ---------- ---------L ---------- -P-----AEG SVVGSAALAH LQQSRGGRRL ASLISGLTAV DPA---A--- --RLGVEEA- LAHPAL----

Cre03.g187300 ---------- ---------- ---------- ---------A YQRYETDM-- --PGWVPAGL RRLVEGLLDE RPC---R--- --RPSAAAA- LCDPYL----

Cre01.g041752 ---------- ---------- ---------- ---------A YTEGRMGL-- --PARLPQGL RDLVARLTAA DAS---Q--- --RPSAREA- LAHPYL----

Cre12.g495401 ---------- ---------- ---------- ---------A YTEGRMGL-- --PARLPQGL RDLVARLTAA DAS---Q--- --RPSAREA- LAHPYL----

Cre06.g266450 ---------- ---------- ---------- ---------A YSQFEDDL-- --DEWVPEGC RDLCAQLLAP SPS---D--- --RPSAKEA- LAHWWL----

Cre06.g266000 ---------- ---------- ---------- ---------A YSLFEDDL-- --DEWVPEGC RDLCAQLLAP SPS---D--- --RPSAKEA- LAHWWL----

Cre06.g266250 ---------- ---------- ---------- ---------A YSLFEDDL-- --DEWVPEGC RDLCAQLLAP SPS---D--- --RPSAKEA- LAHWWL----

Cre10.g460700 ---------- ---------- ---------- ---------Q YALWARDL-- --PHRTPPAL KDFIEQLMSV DPA---E--- --RLTPDQA- LAHPWL----

Cre12.g545750 ---------- ---------- ---------- ---------A YLEYERDL-- --PRRMPPAL KELIEWMVAE DPA---A--- --RPTPDQI- LAHPWL----

Cre12.g525950 ---------- ---------- ---------- ---------A YLEYLRDL-- --PRRTPPAL KELIEWMVAE DPA---A--- --RPTPDQI- LAHRWL----

Cre12.g525750 ---------- ---------- ---------- ---------A YLEYLRDL-- --PRRTPPAL KELIEWMVAE DPA---A--- --RPTPDQI- LAHRWL----

Cre12.g526650 ---------- ---------- ---------- ---------A YLEYLRDL-- --PRRTPPAL KELIEWMVAE DPA---A--- --RPTPDQI- LAHRWL----

Cre12.g526051 ---------- ---------- ---------- ---------A YLEYLRDL-- --PRRTPPAL KELIEWMVAE DPA---A--- --RPTPDQI- LAHRWL----

Cre12.g526250 ---------- ---------- ---------- ---------A YLEYLRDL-- --PRRTPPAL KELIEWMVAE DPA---A--- --RPTPDQI- LAHRWL----

Cre01.g054750 ---------- ---------- ---------- ---------A YLRYERDL-- --PRRTPPAL KELIEWMVAE DPA---A--- --RPTPDQV- LAHRWM----

Cre01.g055457 ---------- ---------- ---------- ---------A YLRMERDL-- --PRRTPPAL KELIEWMVAE DPA---A--- --RPTPDQV- LAHRWM----

Cre09.g404550 ---------- ---------- ---------- ---------- ---------- ---------- ---------- ---------- ---------- ----------

Cre02.g108750b ---------- --------KT ---------- -PADYVAQ-V SKGYRPPR-- -PARLTSDAA WALISDCWQD DPV---Q--- --RPDMDTV- LVRLRELT--

Cre02.g095099 ---------- ---------- ---------- ---------- ---------- ---------- ---------- ---------- ---------- ----------

Cre17.g710600 -----SGTA- ----GAAAKT ---------- -LCRLSASRT TAATAAPV-- -P-VSVPEPL MELMELCWAA EPE---E--- --RPTFEDV- LYHIDC----

CrMAPKKK14 ---------- ---------- ---------- -------GHV TRGGRPVF-- -T-EAVPLPY RRLAAACWSA VPS---R--- --RPRAAEV- VAALTAELA-

CrMAPKKK4 ---------- ---------- ---------- -------RHI YKGARPTF-- -S-DSIPFTY RGLAQACWAA DPS---R--- --RPKASDL- VAFI------

CrMAPKKK8 ---------- ---------- ---------- -Q----CQ-- ----RPPL-- -P-PACPAGL ADLVCACWAD SPE---E--- --RPTFAAV- VERLRRL---

CrMAPKKK1 ---------- ---------- ---------- -R----HHRL P--LSKLD-- -A-NRCPPKL RKLIHQCWDP DPQ---R--- --RPAAAEL- VKQLTLVLQL

CrMAPKKK12 ---------- ---------- ---------- -R----GLSL LSVPQALA-- -E-ERCPPKL KRLLADCFEP DPR---R--- --RPAAAEI- VKQLLLVEEQ

Cre09.g390300 -----SPD-- ---------- ---------- ---------- ---WEGPE-- -P-DEPAPGW TALMTRCWSE AAE---A--- --RPPFPQV- VAELEGML--

CrMAPKKK11 ---------- ---------- ---------- -M----MGVS LGELRPDW-- -S-PADWPEL AALGARCLAQ QPE---D--- --RPSFREL- AEELVRLEEG

CrMAPKKK13 ---------- ---------- ---------- -I----LAVV TRKARPQF-- -P-PDTPHRL KYLAERCWAE LAE---V--- --RPSLQQL- YNELENLQLQ

CrPTK7 ---------- ---------- ---------- -P----HSVA LRGLRPVL-- -P-PHTPPSY RRLAERCWQA EPR---Q--- --RPTFHEI- LDELQRM---

Cre02.g117813 ---------- ---------- ---------- --------VA VAGLRPEF-- -P-AHTPPRF VALAEACWAA DPT---A--- --RPSFEAL- VGRLGELL--

CrMAPKKK10 ---------- ----GAR--T ---------- -G----KPLE ALLIEDML-- -S-RSETRPY AQLAAECLSR DPR---R--- --RPDFVQV- VARLQAIY--

CrMAPKKK2 -----PGDL- ----HGT--S ---------- -G----KPVW --VPWTAP-- -P-SGSSPRL AQLLAACLAM EPS---A--- --RPSFDQV- AEALVAILVE

CrMAPKKK6 ---------- -----AR--E ---------- -S-------- ----FPKF-- -P-RKAPHEF ARVVGLCLDP EPS---Q--- --RPAFVHL- KELLEAQL--

CrMAPKKK5 ---------- -----YC--S ---------- -N-------- ----FPAF-- -P-PTCPEVY KAVTLQCLQR QPQ---N--- --RPPATVI- VGALEHLLA-

CrMAPKKK9 ---------- -----YC--T ---------- -S-------- ----FPAF-- -P-PSCPEGY KAVALQCLQR QPS---N--- --RPTAPTI- VTALEQLL--

Cre12.g516650 ---------- -----LN--T ---------- -G-------- ----ELEF-- -G-PETPPRY ARLARECFHA SSA---A--- --RPSVGVV- VAALERI---

CrMAPKKK7 ---------- -----LN--T ---------- -G-------- ----ELEF-- -G-PDTPPRY ARLARECFSS SKE---A--- --RPIITVV- VTTLNSIKTE

....|....| ....|....| ....|....| ....|....| ....|....| ....|..

1310 1320 1330 1340 1350

CrCDPK9 ---------- ---------- ---------- ---------- ---------- -------

AtCPK25 ---------- ---------- ---------- ---------- ---------- -------

AtCPK26 ---------- ---------- ---------- ---------- ---------- -------

AtCPK6 ---------- ---------- ---------- ---------- ---------- -------

AtCPK5 ---------- ---------- ---------- ---------- ---------- -------

AtCPK20 ---------- ---------- ---------- ---------- ---------- -------

AtCPK2 ---------- ---------- ---------- ---------- ---------- -------

AtCPK1 ---------- ---------- ---------- ---------- ---------- -------

AtCPK12 ---------- ---------- ---------- ---------- ---------- -------

AtCPK11 ---------- ---------- ---------- ---------- ---------- -------

AtCPK4 ---------- ---------- ---------- ---------- ---------- -------

AtCIPK7 ---------- ---------- ---------- ---------- ---------- -------

AtCIPK4 ---------- ---------- ---------- ---------- ---------- -------

AtCIPK22 ---------- ---------- ---------- ---------- ---------- -------

AtCIPK11 ---------- ---------- ---------- ---------- ---------- -------

AtCIPK14 ---------- ---------- ---------- ---------- ---------- -------

AtCIPK12 ---------- ---------- ---------- ---------- ---------- -------

AtCIPK19 ---------- ---------- ---------- ---------- ---------- -------

AtCIPK18 ---------- ---------- ---------- ---------- ---------- -------

AtCIPK13 ---------- ---------- ---------- ---------- ---------- -------

AtCIPK16 ---------- ---------- ---------- ---------- ---------- -------

AtCIPK5 ---------- ---------- ---------- ---------- ---------- -------

AtCIPK25 ---------- ---------- ---------- ---------- ---------- -------

AtCIPK2 ---------- ---------- ---------- ---------- ---------- -------

AtCIPK10 ---------- ---------- ---------- ---------- ---------- -------

AtCIPK15 ---------- ---------- ---------- ---------- ---------- -------

AtCIPK6 ---------- ---------- ---------- ---------- ---------- -------

AtCIPK20 ---------- ---------- ---------- ---------- ---------- -------

AtCIPK21 ---------- ---------- ---------- ---------- ---------- -------

AtCIPK17 ---------- ---------- ---------- ---------- ---------- -------

AtCIPK1 ---------- ---------- ---------- ---------- ---------- -------

AtCIPK3 ---------- ---------- ---------- ---------- ---------- -------

AtCIPK9 ---------- ---------- ---------- ---------- ---------- -------

AtCIPK23 ---------- ---------- ---------- ---------- ---------- -------

AtCIPK8 ---------- ---------- ---------- ---------- ---------- -------

AtCIPK24 ---------- ---------- ---------- ---------- ---------- -------

CrMAPK7 ---------- ---------- ---------- ---------- ---------- -------

CrMAPK9 ---------- ---------- ---------- ---------- ---------- -------

CrMAPK13 ---------- ---------- ---------- ---------- ---------- -------

Cre03.g206202 ---------- ---------- ---------- ---------- ---------- -------

CrMAPK14 ---------- ---------- ---------- ---------- ---------- -------

CrMAPK17 ---------- ---------- ---------- ---------- ---------- -------

CrMAPK16 ---------- ---------- ---------- ---------- ---------- -------

CrMAPK15 ---------- ---------- ---------- ---------- ---------- -------

CrMAPK12 ---------- ---------- ---------- ---------- ---------- -------

CrMAPK10 KLIAGTQLAA AL-------- ---------- ---------- ---------- -------

CrMAPK11 ---------- ---------- ---------- ---------- ---------- -------

CrGSK3 ---------- ---------- ---------- ---------- ---------- -------

CrCDKI1 ---------- ---------- ---------- ---------- ---------- -------

CrMAPK5 ---------- ---------- ---------- ---------- ---------- -------

CrMAPK8 ---------- ---------- ---------- ---------- ---------- -------

CrMAPK6 ---------- ---------- ---------- ---------- ---------- -------

CrMAPK3 ---------- ---------- ---------- ---------- ---------- -------

CrMAPK2 ---------- ---------- ---------- ---------- ---------- -------

CrMAPK4 ---------- ---------- ---------- ---------- ---------- -------

CrCDPK5 ---------- ---------- ---------- ---------- ---------- -------

CrCDPK4 ---------- ---------- ---------- ---------- ---------- -------

CrCDPK3 ---------- ---------- ---------- ---------- ---------- -------

PpCCaMK ---------- ---------- ---------- ---------- ---------- -------

NtCCaMK ---------- ---------- ---------- ---------- ---------- -------

LjCCaMK ---------- ---------- ---------- ---------- ---------- -------

MtCCaMK ---------- ---------- ---------- ---------- ---------- -------

PsCCaMK ---------- ---------- ---------- ---------- ---------- -------

LlCCaMK ---------- ---------- ---------- ---------- ---------- -------

OsCCaMK ---------- ---------- ---------- ---------- ---------- -------

Cre03.g199050 ---------- ---------- ---------- ---------- ---------- -------

CrCDPKK1 ---------- ---------- ---------- ---------- ---------- -------

CrCDPKK2 ---------- ---------- ---------- ---------- ---------- -------

AtMKK10 ---------- ---------- ---------- ---------- ---------- -------

AtMKK4 ---------- ---------- ---------- ---------- ---------- -------

AtMKK5 ---------- ---------- ---------- ---------- ---------- -------

AtMKK8 ---------- ---------- ---------- ---------- ---------- -------

AtMKK7 ---------- ---------- ---------- ---------- ---------- -------

AtMKK9 ---------- ---------- ---------- ---------- ---------- -------

CrMAPKK1 ---------- ---------- ---------- ---------- ---------- -------

AtMKK3 ---------- ---------- ---------- ---------- ---------- -------

CrMAPKK-L ---------- ---------- ---------- ---------- ---------- -------

AtMKK6 ---------- ---------- ---------- ---------- ---------- -------

AtMEK1 ---------- ---------- ---------- ---------- ---------- -------

AtMKK2 ---------- ---------- ---------- ---------- ---------- -------

AtMAPKKK13 ---------- ---------- ---------- ---------- ---------- -------

AtMAPKKK14 ---------- ---------- ---------- ---------- ---------- -------

Cre10.g464100 ---------- ---------- ---------- ---------- ---------- -------

AtANP2 KESASKDLTS FMDNSCSPLP SELTNI---- ---------- -TSYQTSTSD -DVGDIC

AtANP1 ---------- ---------- ---------- ---------- ---------- -------

AtANP3 ---------- ---------- ---------- ---------- ---------- -------

Cre03.g169100 ---------- ---------- ---------- ---------- ---------- -------

Cre17.g735550 ---------- ---------- ---------- ---------- ---------- -------

CrALK3 ---------- ---------- ---------- ---------- ---------- -------

CrALK1 ---------- ---------- ---------- ---------- ---------- -------

CrMAPKKK3 ---------- ---------- ---------- ---------- ---------- -------

AtEDR1 ---------- ---------- ---------- ---------- ---------- -------

AtCTR1 ---------- ---------- ---------- ---------- ---------- -------

CrCNK9 ---------- ---------- ---------- ---------- ---------- -------

AtNEK7 ---------- ---------- ---------- ---------- ---------- -------

AtNEK5 ---------- ---------- ---------- ---------- ---------- -------

AtNEK6 ---------- ---------- ---------- ---------- ---------- -------

AtNEK4 SMVYMKLE-- --SPRRSTFP LQFSERDATL KERRRSSFSN DRRLNPSVSD TEAG---

AtNEK1 ---------- ---------- ---------- ---------- ---------- -------

AtNEK2 ---------- ---------- ---------- ---------- ---------- -------

AtNEK3 ---------- ---------- ---------- ---------- ---------- -------

CrCNK7 ---------- ---------- ---------- ---------- ---------- -------

CrCNK6 ---------- ---------- ---------- ---------- ---------- -------

CrCNK4 ---------- ---------- ---------- ---------- ---------- -------

CrFA2 ---------- ---------- ---------- ---------- ---------- -------

CrCNK1 ---------- ---------- ---------- ---------- ---------- -------

CrCNK2 ---------- ---------- ---------- ---------- ---------- -------

CrCNK8 QSYL------ ---------- ---------- ---------- ---------- -------

CrCNK5 ---------- ---------- ---------- ---------- ---------- -------

CrFAP403 ---------- ---------- ---------- ---------- ---------- -------

CrCNK3 ---------- ---------- ---------- ---------- ---------- -------

CrCDPKK3 ---------- ---------- ---------- ---------- ---------- -------

CrMAPK1 SVDGVGWAGG WVGG-CW--- ---------- ---------- ---------- -------

Cre08.g379600 ---------- ---------- ---------- ---------- ---------- -------

Cre02.g108601 ---------- ---------- ---------- ---------- ---------- -------

Cre02.g108750a ---------- ---------- ---------- ---------- ---------- -------

Cre17.g713750 VI-------- ---------- ---------- ---------- ---------- -------

Cre11.g467586 ---------- ---------- ---------- ---------- ---------- -------

Cre11.g467588 ---------- ---------- ---------- ---------- ---------- -------

Cre11.g467589 ---------- ---------- ---------- ---------- ---------- -------

Cre11.g467584 ---------- ---------- ---------- ---------- ---------- -------

Cre11.g467585 ---------- ---------- ---------- ---------- ---------- -------

Cre12.g489850 MPQF------ ---------- ---------- ---------- ---------- -------

Cre12.g490200 VL-------- ---------- ---------- ---------- ---------- -------

Cre08.g384250 ---------- ---------- ---------- ---------- ---------- -------

Cre04.g217940 ---------- ---------- ---------- ---------- ---------- -------

Cre04.g217923 ---------- ---------- ---------- ---------- ---------- -------

Cre04.g217928 ---------- ---------- ---------- ---------- ---------- -------

Cre08.g369700 ---------- ---------- ---------- ---------- ---------- -------

Cre08.g369900 ---------- ---------- ---------- ---------- ---------- -------

Cre03.g187300 ---------- ---------- ---------- ---------- ---------- -------

Cre01.g041752 ---------- ---------- ---------- ---------- ---------- -------

Cre12.g495401 ---------- ---------- ---------- ---------- ---------- -------

Cre06.g266450 ---------- ---------- ---------- ---------- ---------- -------

Cre06.g266000 ---------- ---------- ---------- ---------- ---------- -------

Cre06.g266250 ---------- ---------- ---------- ---------- ---------- -------

Cre10.g460700 ---------- ---------- ---------- ---------- ---------- -------

Cre12.g545750 ---------- ---------- ---------- ---------- ---------- -------

Cre12.g525950 ---------- ---------- ---------- ---------- ---------- -------

Cre12.g525750 ---------- ---------- ---------- ---------- ---------- -------

Cre12.g526650 ---------- ---------- ---------- ---------- ---------- -------

Cre12.g526051 ---------- ---------- ---------- ---------- ---------- -------

Cre12.g526250 ---------- ---------- ---------- ---------- ---------- -------

Cre01.g054750 ---------- ---------- ---------- ---------- ---------- -------

Cre01.g055457 ---------- ---------- ---------- ---------- ---------- -------

Cre09.g404550 ---------- ---------- ---------- ---------- ---------- -------

Cre02.g108750b ---------- ---------- ---------- ---------- ---------- -------

Cre02.g095099 ---------- ---------- ---------- ---------- ---------- -------

Cre17.g710600 ---------- ---------- ---------- ---------- ---------- -------

CrMAPKKK14 ---------- ---------- ---------- ---------- ---------- -------

CrMAPKKK4 ---------- ---------- ---------- ---------- ---------- -------

CrMAPKKK8 ---------- ---------- ---------- ---------- ---------- -------

CrMAPKKK1 V--------- ---------- ---------- ---------- ---------- -------

CrMAPKKK12 WV-------- ---------- ---------- ---------- ---------- -------

Cre09.g390300 ---------- ---------- ---------- ---------- ---------- -------

CrMAPKKK11 L--------- ---------- ---------- ---------- ---------- -------

CrMAPKKK13 LC-------- ---------- ---------- ---------- ---------- -------

CrPTK7 ---------- ---------- ---------- ---------- ---------- -------

Cre02.g117813 ---------- ---------- ---------- ---------- ---------- -------

CrMAPKKK10 ---------- ---------- ---------- ---------- ---------- -------

CrMAPKKK2 SHRNAQHNV- -L-------- ---------- ---------- ---------- -------

CrMAPKKK6 ---------- ---------- ---------- ---------- ---------- -------

CrMAPKKK5 ---------- ---------- ---------- ---------- ---------- -------

CrMAPKKK9 ---------- ---------- ---------- ---------- ---------- -------

Cre12.g516650 ---------- ---------- ---------- ---------- ---------- -------

CrMAPKKK7 LEALPHS--- ---------- ---------- ---------- ---------- -------
