# Supplementary material for: Chlamydomonas CHT7 is involved in repressing DNA replication and mitotic genes during synchronous growth
Source: G3 (Bethesda). 2022 Feb 7;12(3):jkac023. doi: 10.1093/g3journal/jkac023 (PMC8895990; doi:10.1093/g3journal/jkac023)
Supplement: jkac023_Supplemental_Figure_Legends [file jkac023_supplemental_figure_legends.docx]

**Supplemental Information**

**Figure S1.** Cell cycle analysis of cell cultures grown under synchronizing conditions.

Cell size distribution (left column) and flow cytometry DNA analysis (middle and right columns) of synchronized cells of WT, *CHT7-HA::cht7*, and *cht7* grown under N-replete, N-deprived, and N-resupplied conditions. For cell size measurement (left column), cells sampled at different timepoints were fixed with 0.2% of Glutaraldehyde prior to analysis. *X*-axis: cell volume (fL). *Y*-axis: population (%) of cells. The most abundant population of the ZT0 sample is labeled in blue and serves as a reference for other samples. For DNA measurement (middle and right column), cells were fixed with ethanol: acetic acid fixation buffer and stained with DNA dye SYTOX Green, as described in Materials and Method. Middle column: histogram of cells stained with SYTOX Green. *X*-axis: DNA content determined by the intensity of FITC-A. *Y*-axis: population (%) of cells. Right column: scatter plot of cells stained with SYTOX Green. *X*-axis: DNA content determined by the intensity of FITC-A. *Y*-axis: Particle size determined by FSC-A (Forward scatter). Supplemental information to Figure 1.

**Figure S2.** GO enrichment analysis of up-regulated *cht7*-DEGs across all timepoints.

A total of 184 GO terms of biological processes were identified to be significantly (*P* < 0.05) enriched in the up-regulated *cht7*-DEGs at ≥ 1 timepoints. GO terms of biological processes that were explicitly enriched in N+ (light green), ND (light orange), NR (light blue) conditions are highlighted, and others (cross-conditions) are in grey. The number of up-regulated DEGs is labeled in the boxes. The number of total annotated genes is indicated after GO terms. Supplemental information to Figure 3.

**Data S1.** Multiple sequence alignment of the kinase domain of 166 kinases. Supplemental information to Figure 7.

**Table S1.** GO enrichment analysis of the 14 gene clusters shown in Figure 2.

**Table S2.** GO enrichment analysis of downregulated DEGs of *cht7* and *CHT7-HA::cht7* compared to the wild type at ZT0.

**Table S3.** Reference tables of genes involved in cell cycle, cell wall, and protein phosphorylation.

**Table S4.** Kinase sequences for phylogenetic analysis in Figure 8.
